# Oral abstracts of the 21st International AIDS Conference

**DOI:** 10.7448/IAS.20.6.22253

**Published:** 2017-07-27

## MOAA0101

### Assessing individual viral reactivations of the latent reservoir using a novel barcoded virus

C Fennessey ^1^; M Pinkevych^2^; T Immonen^1^; C Camus^1^; G Del Prete^1^; J Estes^1^; J Lifson^1^; M Davenport^2^ and B Keele^1^

^1^Leidos Biomedical Research Inc., Frederick National Laboratory, AIDS and Cancer Virus Program, Frederick, USA. ^2^Kirby Institute for Infection and Immunity, University of New South Wales, Sydney, Australia

Presenting author email: christine.fennessey@nih.gov

**Background**: An overwhelming obstacle to HIV cure is the presence of long-lived viral reservoirs that can reignite viral infection after removal of combination antiretroviral therapy (cART). Yet, our knowledge of the mechanisms of reservoir maintenance and persistence remains incompletely understood. Therefore, in this study, we generated a novel virus model for evaluating individual reactivation events following cART interruption to better understand key aspects of reservoir biology *in vivo*.

**Methods**: A genetically tagged virus (SIVmac239M) was generated with a 34-base molecular barcode stably inserted between *vpx* and *vpr* of SIVmac239. Rhesus macaques were infected intravenously and cART was administered, beginning either on day 6 for 82 days (*n* = 4) (study 1) or on day 4 for 305, 374 or 482 days (*n* = 6) (study 2). Next-generation sequencing was used to evaluate the number of genetic variants in the stock and in plasma before and after treatment.

**Results**: In the viral stock, 9336 individual barcodes, or clonotypes, were identified. During acute infection, an average of 1247 barcodes were identified in each of the 10 animals. Plasma viraemia was reduced to 15 vRNA copies/ml during treatment, and virus rapidly rebounded following treatment interruption. Between 87 and 136 distinct clonotypes were detected in plasma at peak rebound viraemia in animals from study 1 and between 4 and 7 clonotypes in animals from study 2. Because the growth rate of each clonotype is equivalent once the virus reaches systemic infection, the measured relative proportions of each clonotype at rebound reflect the time between when each clonotype achieved systemic infection. The viral growth rate and the relative abundance of each clonotype in plasma during acute recrudescence were used to estimate a reactivation rate of 22.7 and 0.54 events per day in studies 1 and 2, respectively.

**Conclusions**: We conclude that identifying rebounding clonotypes may be used as a direct measurement of the latent reservoir that can successfully contribute to rebound viraemia. Furthermore, the results confirm that the size of the post-cART recrudescence-competent viral reservoir is influenced by the timing of cART initiation and duration of treatment, enabling manipulation of these parameters to establish reservoirs of desired size for specific experimental purposes.

## MOAA0102

### Accumulation and persistence of deleted HIV proviruses following prolonged ART

E Anderson ^1^; S Hill^1^; J Bell^2^; F Simonetti^1^; C Rehm^3^; S Jones^4^; R Gorelick^2^; J Coffin^5^ and F Maldarelli^1^

^1^National Cancer Institute, HIV Dynamics and Replication Program, Frederick, USA. ^2^Leidos, Biomedical Research Inc, Frederick, USA. ^3^National Institutes of Allergy and Infectious Diseases, Laboratory of Immunoregulation, Bethesda, USA. ^4^Leidos, Biomedical Research Inc, Clinical Monitoring Research Program, Frederick, USA. ^5^Department of Molecular Biology and Microbiology, Tufts University, Boston, USA

Presenting author email: elizabeth.anderson@nih.gov

**Background**: HIV persistence during antiretroviral therapy (ART) is a substantial obstacle to HIV cure. HIV-infected cells can undergo clonal expansion, and specific clones may be highly expanded. We previously reported one provirus integrated in the *HORMAD2* gene that accounted for 20–40% of all proviruses in one patient. Mechanisms by which clones emerge and persist over time are uncertain. To investigate the dynamics of HIV clonal expansion, we developed multiplexed droplet digital approaches (ddPCR) to quantify HIV proviruses, including specific integrants, prior to and following prolonged ART.

**Methods**: HIV-infected ART-naive individuals (*N* = 11) underwent ART and were followed for a median of 13.7 years (range 4.3–16.4). Cell-associated DNA (CA-DNA) from peripheral blood lymphocytes pre-ART, during first- and second-phase viral decay and after prolonged ART was quantified using multiplexed ddPCR assays targeting HIV *gag*, LTR and *tat/rev* regions, as well as a host gene (*CCR5*). We designed a specific ddPCR primer set overlapping the host–HIV junction to quantify the HIV integrant in *HORMAD2.*

**Results**: All patients had successful suppression of HIV RNA on ART to <50 copies/ml plasma within five months; HIV DNA copies/million CD4+ cells decreased for *gag* (average 15-fold), LTR (average 9.3-fold) and *tat/rev* (average 20-fold) regions. In 10/11 patients, the LTR:*gag* and LTR:*tat/rev* ratios increased progressively after second-phase decay (average 6-fold and 6.4-fold, respectively, *p* < 0. 01, paired *T*-test), demonstrating loss of full-length proviruses; in 1/11 patients, LTR:*gag* and LTR:*tat/rev* ratios remained stable. The *HORMAD2* integrant was undetectable at pre-ART and one month and two months on ART (<1 copy in 500,000 infected cells). After one year on suppressive ART, however, the *HORMAD2* integrant was present at a frequency of 30% of all infected cells and persisted for six years on ART.

**Conclusions**: Progressive appearance of deleted proviruses is detectable in most but not all patients undergoing ART. Substantial deletion did not appear during first- or second-phase viral decay, but only after one to four years during suppressive therapy. Clonal expansion of HIV-infected cells can be rapid and sustained at stable levels during prolonged ART, suggesting that both antigen-induced clonal expansion and homeostatic proliferation maintain HIV populations.

## MOAA0103

### A subset of extreme HIV controllers is characterized by a small HIV blood reservoir and a weak T-cell activation level

E Canoui^1,2^; C Lecuroux^2^; V Avettand-Fenoël^3^; M Gousset^3^; C Rouzioux^3^; A Saez-Cirion^4^; L Meyer^5^; F Boufassa^5^; O Lambotte^1,2^; N Noel ^1,2,4^ and ANRS CO21 CODEX Study Group

^1^AP-HP, Hopital BIcêtre, Service de Médecine Interne et Immunologie Clinique, Le Kremlin Bicêtre, France. ^2^UMR INSERM/CEA U1184, Le Kremlin Bicêtre, France. ^3^AP-HP, Hôpital Necker-Enfants-Malades, Service de Virologie, Paris, France. ^4^Institut Pasteur, Unité HIV, Inflammation et Persistance, Paris, France. ^5^INSERM U1018, CESP, Le Kremlin Bicêtre, France

Presenting author email: nicolas.noel@aphp.fr

**Background**: HIV controllers (HICs) form a heterogeneous group of patients with regard to formal definitions, immunologic characteristics and changes over time in viral load.

**Methods**: HICs with undetectable viral load (uHICs, i.e. for whom a viral load had never been detected with routine assays, *n* = 52) were compared with 178 HICs with blips during the follow-up (bHICs). Clinical characteristics, ultrasensitive HIV-RNA and HIV-DNA loads, HIV-1-Western blot profiles and immune parameters were analysed.

**Results**: Relative to bHICs, uHICs had significantly lower ultrasensitive plasma HIV-RNA loads (median <4 copies/ml (IQR <2 to <4) vs. 21 copies/ml (7–84), *p *< 0.0001) and HIV-DNA levels in PBMC (<10 copies per million PBMCs (<10–11) vs. 21 (<10–52), *p *= 0.0004), higher CD4+ T cell count (790 (638–1038) vs. 711 (520–920), *p *= 0.04) at enrolment and lower T-cell activation levels. Half the uHICs were characterized by having a protective HLA allele (-B57/58/B27), a weak CD8+ T cell response and very small HIV-DNA reservoir. Between diagnosis and inclusion in the cohort, the CD4+ T cell count had not changed in uHICs but had significantly decreased in bHICs (−5.16 CD4/µl/year, *p *= 0.001). About 21% of the uHICs lacked specific anti-HIV IgG antibodies. These individuals also had very low levels of HIV-DNA, and 83% of these patients had both undetectable HIV DNA and us HIV RNA compared with 40% of uHICs with full anti-HIV IgG responses.

**Conclusions**: We suggest that an optimal HIC phenotype combines protective HLA alleles, low level of HIV blood reservoirs and reduced immune activation. Such patients may have limited benefit from antiretroviral therapy.

## MOAA0104

### HIV integration sites in CD4 T cells from virally suppressed individuals show clonal expansion but no preferential location in oncogenes

J Symons ^1^; A Chopra^2^; S Leary^2^; D Cooper^2^; JL Anderson^1^; JJ Chang^1^; J McMahon^3^; SG Deeks^4^; S Mallal^2,5^; PU Cameron^1,6^ and SR Lewin^1,6^

^1^The Peter Doherty Institute for Infection and Immunity, University of Melbourne and Royal Melbourne Hospital, Melbourne, Australia. ^2^Institute for Immunology and Infectious Diseases (IIID), Murdoch University, Perth, Australia. ^3^Department of Infectious Diseases Alfred Hospital and Monash University, Melbourne, Australia. ^4^Department of Medicine, University of California, San Francisco, USA. ^5^Department of Pathology, Microbiology and Immunology, Vanderbilt University, Nashville, USA. ^6^Department of Infectious Diseases, Alfred Hospital and Monash University, Melbourne, Australia

Presenting author email: jori.symons@unimelb.edu.au

**Background**: Assessing HIV integration site location and frequency in latently infected cells can inform how HIV persists on antiretroviral therapy (ART). We aimed to characterize HIV integration sites in CD4 T cells from blood and tissue from HIV-infected individuals on suppressive ART.

**Methods**: Genomic DNA from 1 million CD4 T cells obtained from blood, lymph node (LN) or rectal tissue from virologically suppressed participants on ART was enzymatically cut to random-sized fragments, tagged and amplified by nested PCR with barcoded primers before Miseq sequencing. Chromosomal alignment was determined using Blat-UCSC Genome Browser (GRCH38/hg38). Clonal expansion of HIV integration sites was defined if identical HIV integration sites differed >2 base pairs in PCR-product length and ≥3 length polymorphisms were present.

**Results**: We assessed 1794 integration sites from seven participants receiving ART for a median [range] of 14 [7–19] years with an undetectable viral load for 6 [4–16] years. Integration was enriched in genes vs. non-genes (78 [58–87]% vs. 23 [13–42]%, *p* < 0.001). The majority of integration sites into genes were intronic (median 86 [72–97]% vs. 13 [3–18]% exonic, *p* < 0.001) and demonstrated preferential insertion in the opposite orientation relative to gene transcription (median 60 [47–68]% vs. 40 [32–52]% same orientation, *p* = 0.007). In blood, we observed a median of 54 [25–70]% clonally expanded integration sites. Interestingly, the frequency of clonal expansion was different in blood and tissue. In matched samples of two participants, the frequency of clonal expansion in LN was similar to blood, whereas rectal tissue showed the least amount of clonal expansion (Table 1). Integration sites in expanded compared to non-expanded clones were enriched in genes (84% vs. 69%, respectively, *p* = 0.04), but there was no enrichment in oncogenes (median 22% vs. 25%, respectively, *p* = 0.74).

**Abstract MOAA0104–Table 1.** Clonal expansion of HIV integration sites

	CD4 count	HIV DNA/10^6^ CD4 cells	% Clonal expansion in blood	% Clonal expansion in LN	% Clonal expansion rectal tissue
Participant 6	578	1500	32	38	17
Participant 7	521	494	25	–	10

**Conclusions**: Expanded clones of infected cells in blood and tissues are common. HIV integration is preferentially detected in intronic regions but not oncogenes with orientations that are usually in opposite direction. The relative contribution of expanded clones to HIV persistence may differ in different tissue sites, but analyses of further tissue samples are needed.

## MOAA0105

### The impact of treatment duration on defective and expanded identical HIV genomes in T-cell subsets from peripheral blood and tissues

E Lee ^1,2^; S von Stockenstrom^3^; V Morcilla^1^; L Odevall^4^; B Hiener^1,2^; W Show^5^; W Hartogensis^6^; P Bacchetti^7^; J Milush^6^; T Liegler^6^; E Sinclair^6^; H Hatano^6^; R Hoh^6^; M Somsouk^6^; P Hunt^6^; E Boritz^8^; D Douek^8^; R Fromentin^9^; N Chomont^9^; SG Deeks^6^; FM Hecht^6^ and S Palmer^1,2^

^1^The Westmead Institute for Medical Research, Centre for Virus Research, Westmead, Australia. ^2^The University of Sydney, Sydney Medical School, Camperdown, Australia. ^3^Department of Microbiology, Tumor and Cell Biology, Karolinska Institutet, Karolinska University Hospital, Stockholm, Sweden. ^4^Karolinska Institutet, Karolinska University Hospital, Deparment of Microbiology, Tumor and Cell Biology, Stockholm, Sweden. ^5^Leidos Biomedical Research Inc., Frederick National Laboratory for Cancer Research, Advanced Biomedical Computing Center, Frederick, USA. ^6^Department of Medicine, University of California San Francisco, San Francisco, USA. ^7^Department of Epidemiology and Biostatistics, University of California San Francisco, San Francisco, USA. ^8^National Institute of Allergy and Infectious Diseases, National Institutes of Health, Human Immunology Section, Vaccine Research Center, Bethesda, USA. ^9^Centre de recherche du CHUM and Department of Microbiology, Infectiology and Immunology, Université de Montréal, Montreal, Canada

Presenting author email: eunok.lee@sydney.edu.au

**Background**: Understanding the impact of antiretroviral therapy (ART) duration on HIV-1 reservoirs is critical for implementing effective curative strategies. We studied the distribution of defective viral genomes in T-cell subsets within blood and tissues over 3–18 years of effective ART. We also examined expansions of identical HIV-1 DNA sequences (EIS) to assess the contribution of cellular proliferation to viral persistence during ART.

**Methods**: Using single-genome/proviral sequencing, we performed inter-patient analysis of 479 HIV-1 p6-RT RNA sequences from pre- and early-ART plasma and 2329 HIV-1 DNA sequences from naive (N), central (CM), transitional (TM), effector (EM), gut homing and lymph homing memory CD4+ T cells sorted from peripheral blood (PB), lymph node (LN) and gut tissues from 14 participants who initiated ART during chronic infection (3–18 years on ART). Defective viral sequences had hypermutation, premature stops, frameshifts and large deletions. EIS were determined as ≥2 identical intact or defective HIV-1 DNA sequences across all cell types from all anatomic sites.

**Results**: Defective HIV-1 DNA sequences did not appear to accumulate substantially over 3–18 years of ART in any anatomic site (odds ratio = 0.90–1.02/year, *p* = 0.14–0.98). The viral sequences derived from pre- and early-ART plasma samples were more often genetically intact than sequences derived from PB and LN after several years of therapy (*p* ≤ 0.004). The odds of an HIV-1 DNA sequence belonging to EIS increased in PB by 1.09-fold/year of ART (*p* = 0.003). In tissues, the increase of the proportion in EIS during each additional year of ART was not statistically significant (*p* = 0.15–0.25). Of note, 37–71% of identical HIV-1 DNA sequences were in EM sorted from PB and tissues. The odds that a viral sequence is part of an EIS in PB-derived EM increased by 1.11-fold/year (*p* = 0.007), whereas other cell types showed no statistically significant trend (*p* = 0.12–0.46).

**Conclusions**: The proportion of proviruses that were defective did not increase during effective therapy, suggesting that they are established in cells prior to viral suppression. Our data suggest that proliferation of HIV-infected T cells increased the proportion of sequences in EIS within PB over 3–18 years of therapy, and the EM cells were the major contributor.

## MOAA0201

### Sequential receptor-induced conformational states of native membrane-embedded HIV-1 Env

B Ivan; N Friedrich; Z Sun and A Trkola

University of Zurich, Institute of Medical Virology, Zurich, Switzerland

Presenting author email: ivan.branislav@virology.uzh.ch

**Background**: To fulfil its function of mediating virus attachment and entry, the HIV-1 envelope glycoprotein (Env) trimer undergoes a sequence of conformational changes. Due to their unstable nature, entry intermediates remain challenging to study, leaving several aspects of the entry process unresolved. This includes effects of CD4 and coreceptor engagement on the formation of successive Env intermediates and the stoichiometry of receptor and coreceptor binding. Knowledge on the different conformational states of native Env during the entry process is however crucial to understand its antigenic landscape and vulnerability to neutralization. While Env-directed broadly neutralizing antibodies (bNAbs) are thought to recognize Env preferentially in its closed, unliganded form, we currently lack information on their activity across different entry steps. As activity across intermediate Env conformations could increase the window of action, an antigenic profile of receptor-bound Env needs to be defined to select the best bnAbs for therapy and vaccine design.

**Methods**: We have developed a flow cytometry-based assay to assess and compare antigenic properties and conformational stability of fully native, cell surface-expressed Env trimers using an array of Env-directed Abs and compounds. HIV-1 pseudovirus neutralization activity of a panel of NAbs was determined to explore links between open Env conformation and neutralization sensitivity.

**Results**: Our analysis provides a fine mapping of sequential exposure of shielded Env epitopes upon exposure to increasing doses of soluble CD4 (sCD4). Conformational changes induced by sCD4 binding proved consistent with a model where occupation of a promoter alters conformation of all three simultaneously. Increasing promoter occupancy by sCD4 promoted transition through intermediate, progressively more open Env conformations. According to our data, exposure of the coreceptor-binding site may require at least two CD4 molecules to be bound per trimer. Most intriguingly, upon saturation of CD4-binding sites, the trimer adopts a pre-hairpin conformation even in the absence of coreceptor interaction.

**Conclusions**: We provide here novel insights on antigenic characteristics of unliganded and receptor-bound conformations of native Env, which offer means to decipher critical steps of the entry process with high relevance for Env immunogen selection and design.

## MOAA0202

### Optimized Env trimer immunization parameters amplify onset, magnitude and consistency of autologous Tier 2 neutralizing antibody development in nonhuman primates

M Pauthner ^1^; C Havenar-Daughton^2^; D Sok^1^; J Nkolola^3^; R Bastidas^1^; A Boopathy^4^; D Carnathan^5^; A Chandrashekar^3^; K Cirelli^2^; C Cottrell^1^; A Eroshkin^6^; J Guenaga^1^; D Kulp^1^; J Liu^3^; L McCoy^1^; G Ozorowski^1^; K Post^6^; S Sharma^1^; J Steichen^1^; S de Taeye^7^; T Tokatlian^4^; A Torrents de la Peña^7^; S Butera^1^; C LaBranche^8^; D Montefiori^8^; G Silvestri^5^; IWilson^1^; D Irvine^4^; R Sanders^7^; W Schief^1^; A Ward^1^; R Wyatt^1^; D Barouch^3^; S Crotty^2^ and D Burton^1^

^1^The Scripps Research Institute, La Jolla, USA. ^2^La Jolla Institute for Allergy and Immunology, La Jolla, USA. ^3^Beth Israel Deaconess Medical Center, Harvard Medical School, Boston, USA. ^4^Massachusetts Institute of Technology, Koch Institute for Integrative Cancer Research, Cambridge, USA. ^5^Emory University and Yerkes National Primate Research Center, Atlanta, USA. ^6^Sanford-Burnham-Prebys Medical Discovery Institute, La Jolla, USA. ^7^University of Amsterdam, Amsterdam, The Netherlands. ^8^Duke University Medical Center, Durham, USA

Presenting author email: pauthner@scripps.edu

**Background**: The development of soluble native-like HIV envelope trimers has reinvigorated hopes for protein-based vaccination strategies to induce protective antibody responses to HIV. However, unlike in rabbits, immunogens of this class have thus far induced only modest and inconsistent autologous neutralizing antibody (NAb) titres in rhesus macaques, raising concerns regarding the translatability of this approach. Moreover, Tier 2 NAbs have generally taken six months to develop in macaques.

**Methods**: Here, we investigated the effects of immunization schedule, route, dose and delivery on quantity, quality and rapidity of NAb development in head-to-head comparisons, using 6–12 macaques per group. All 78 animals in the cohort were tightly matched for age, weight and gender.

**Results**: Major effects were observed. We identified strategies in which 100% of animals developed mean Tier 2 NAb titres of over 1:100 by week 10. Geometric mean titres at week 26 following two boosts were consistently higher than 1:200 (Figure 1). Effects of trimerization strategy (SOSIP vs. NFL) and immunization dose (100 vs. 20µg) on autologous NAb titres were not significant. We further evaluated and compared novel SOSIP stabilization techniques (SOSIP v4.1, v5.2, Olio6), some of which significantly (*p* < 0.001) reduced induction of Tier 1 NAbs over previously reported strategies, while maintaining strong Tier 2 NAbs. Germinal centre, T cell and Ab responses were extensively analysed in all animals through fine-needle aspirates, particularly those with BG505 NAb titres of 1:1000–1:20,000. Finally, high autologous Tier 2 neutralizing macaques also developed some neutralization breadth on a global Tier 2 viral panel.

**Abstract MOAA0202–Figure 1.**
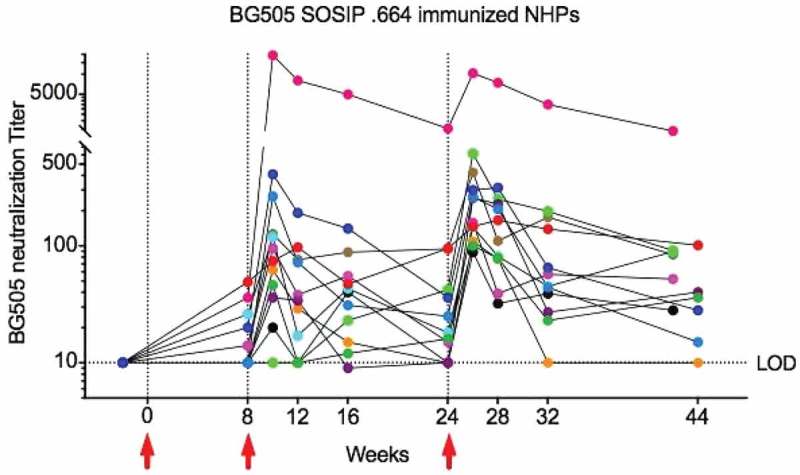
BG505 SOSIP .664 immunized NHPs.

**Conclusions**: In summary, this study provides a framework for preclinical and clinical vaccine studies targeting the elicitation of neutralizing antibodies by trimer immunization. We identified the when, where and how of trimer immunogen delivery to maximize NAb titre induction *in vivo*.

## MOAA0203

### Germinal centres monitored by lymph node fine-needle aspirates correlate with and predict HIV Tier 2 neutralizing antibody responses after HIV trimer immunization

C Havenar-Daughton ^1,2^; D Carnathan^2,3^; M Pauthner^2,4^; J Liu^2,5^; J Nkolola^2,5^; A Torrents de la Pena^6^; B Briney^2,4^; S Reiss^2,7^; J Wood^3^; K Kaushik^2,7^; K Le^2,4^; D Sok^2,4^; M Van Gils^6^; S Kasturi^3^; B Pulendran^2,3^; R Sanders^6^; R Wyatt^2,4^; A Ward^2,4^; W Schief^2,4^; G Seumois^7^; G Silvestri^2,3^; D Barouch^2,5^; D Burton^2,4^ and S Crotty^2,7^

^1^La Jolla Institute for Allergy and Immunology, Vaccine Discovery, La Jolla, USA. ^2^Scripps Center for HIV AIDS Vaccine Immunology and Immunogen Design (CHAVI-ID), La Jolla, USA. ^3^Yerkes National Primate Center and Emory University, Atlanta, USA. ^4^The Scripps Research Institute, La Jolla, USA. ^5^Harvard University, Cambridge, USA. ^6^University of Amsterdam, Amsterdam, The Netherlands. ^7^La Jolla Institute for Allergy and Immunology, Vaccine Discovery, San Diego, USA

Presenting author email: chavenar@lji.org

**Background**: The discovery of HIV broadly neutralizing antibodies in HIV-positive individuals has re-galvanized efforts to develop a protective, antibody-based HIV vaccine. However, generation of neutralizing antibody (nAb) responses to clinically relevant strains of HIV (Tier 2) by immunization remains a challenging problem. Furthermore, the immunological barriers to induction of such responses by Env immunogens are unclear.

**Methods**: To directly study the germinal centre (GC) responses induced after immunization, we have now performed over 1000 lymph node fine-needle aspirates (LN FNA) of draining LNs in rhesus macaques immunized with native-like HIV-1 Env trimer proteins, such as BG505 SOSIP. LN FNA sampling captured GC cells and was highly representative of whole LN biopsies. Greater than 95% of samples provided sufficient cells for identifying the major cell types of the GC, GC B cells and GC T follicular helper (Tfh) cells. LN FNAs also afforded an opportunity for gene expression analysis of antigen-specific GC Tfh cells by RNAseq and longitudinal BCR sequencing to track evolution of the Env-specific B-cell response.

**Results**: A majority of immunized animals developed autologous Tier 2 neutralizing antibodies. Tier 2 nAb development was most strongly associated with the magnitude of the GC response in an initial study (*p* = 0.007). In a subsequent study, the GC responses predicted Tier 2 nAb development (*p* = 0.014). Notably, Tier 2 nAbs did not correlate with BG505 SOSIP Ab binding titres. Thus, the GC responses distinguish between nAb responder and nAb non-responder monkeys, but ELISA binding titre does not.

**Conclusions**: Longitudinal LN FNA sampling has provided strong evidence that GC B and GC Tfh cell responses are central to generating HIV Tier 2 nAbs by immunization.

## MOAA0204

### Rapid elicitation of broadly neutralizing antibodies to HIV by immunization in cows

D Sok ^1^; K Le^2^; M Vadnais^2^; K Saye-Francisco^2^; L Kong^2^; R Stanfield^2^; J Jardine^2^; J Ruiz^1^; A Ramos^1^; C-H Liang^2^; P Chen^3^; M Criscitiello^3^; M Waithaka^3^; I Wilson^2^; V Smider^2^ and D Burton^2^

^1^International AIDS Vaccine Initiative, La Jolla, USA. ^2^The Scripps Research Institute, La Jolla, USA. ^3^Texas A&M University, College Station, USA

Presenting author email: dsok@iavi.org

**Background**: No immunogen to date has reliably elicited broadly neutralizing antibodies (bnAbs) to HIV in humans or animal models. Recent advances in the design of immunogens (BG505 SOSIP) that antigenically mimic the HIV envelope glycoprotein (Env) have improved the elicitation of potent isolate-specific Ab responses in rabbits and macaques, but so far failed to induce bnAbs. One possible contributor to this failure is that the relevant antibody repertoires are poorly suited to target occluded conserved epitope regions on Env relative to exposed variable epitope regions. The antibody repertoire of cows contains long third heavy chain complementary-determining regions (HCDR3) with an ultralong subset that can reach nearly 70 amino acids in length.

**Methods**: Here, we show that immunization of cows with BG505 SOSIP results in the rapid elicitation of broad and potent serum antibody responses. Four cows were immunized in total. Serum were collected over the course of immunization and evaluated for neutralization breadth and potency. Single memory B cells were then antigen sorted with BG505 SOSIP, and heavy and light chain variable genes were amplified by PCR.

**Results**: All immunized cows developed broad and potent neutralizing serum responses. Longitudinal serum analysis in one cow showed the development of neutralization breadth (19%, *n* = 114 cross-clade isolates) in 42 days and peak breadth (100%, *n* = 12 global isolates panel) at 217 days. A monoclonal antibody was isolated from this cow which harboured an ultralong HCDR3 of 64 amino acids and was able to recapitulate 69% of neutralization breadth with a potent median IC_50_ of 0.03µg/ml. The antibody epitope mapped to the CD4-binding site of HIV Env.

**Conclusions**: Despite the inherent difficulty of eliciting broad and potent responses to HIV in most test animals, immunization with a trimer mimic in cows was able to show rapid responses in only 42 days, supporting the notion that the frequency of long HCDR3s is a critical factor in the ability to elicit HIV bnAbs. The results further suggest that immunization of cows may provide an avenue to quickly generate antibody prophylactics and therapeutics to address disease agents that have evolved to avoid human antibody responses.

## MOAA0205

### Killing of HIV-1-infected cells by neutralizing antibodies

T Bruel ^1,2,3^; F Guivel Benhassine^1,2^; V Lorin^4,5^; F Baleux^6^; H Lortat Jacob^7,8,9^; K Bourdic^10,11^; N Noel^10,11,12^; O Lambotte^10,11,13^; H Mouquet^4,14^ and O Schwartz^1,15^

^1^Institut Pasteur, Virology - Virus and Immunity Unit, Paris, France. ^2^CNRS UMR 3569, Paris, France. ^3^Vaccine Research Institute, Créteil, France. ^4^Institut Pasteur, Immunology - Laboratory of Humoral Response to Pathogens, Paris, France. ^5^INSERM U 1222, Paris, France. ^6^Institut Pasteur, Unité de Chimie des Biomolécules UMR CNRS 3523, Paris, France. ^7^Institut de Biologie Structurale, Grenoble, France. ^8^UMR 5075 CNRS CEA, Grenoble, France. ^9^Université Grenoble-Alpes, Grenoble, France. ^10^CEA DSV IMETI Division of Immuno-Virology, IDMIT, Paris, France. ^11^Université Paris Sud UMR 1184, Paris, France. ^12^Center of Immunology of Viral Infections and Auto Immune Diseases, Paris, France. ^13^Center of Immunology of Viral Infections and Autoimmune Diseases, Inserm U 1184, Paris, France. ^14^INSERM U 1222, Vaccine Research Institute, Créteil, France. ^15^CNRS UMR 3569, Vaccine Research Institute, Créteil, France

Presenting author email: timothee.bruel@pasteur.fr

**Background**: Anti-HIV-1 non-neutralizing antibodies (nnAbs) capable of antibody-dependent cellular cytotoxicity (ADCC) have been identified as a protective immune correlate in the RV144 vaccine efficacy trial. Broadly neutralizing antibodies (bNAbs) may also mediate ADCC and rely on their Fc region for optimal efficacy in animal models.

**Methods**: Here, we selected 10 bNAbs and 9 nnAbs, targeting various epitopes and conformations of the gp120/41 complex, and analysed the potency of the two types of antibodies to bind and eliminate HIV-1-infected cells through NK engagement in culture. We tested their activity against 18 HIV-1 strains, including primary viruses.

**Results**: The landscape of Env epitope exposure at the surface and the sensitivity of infected cells to ADCC vary considerably between viral strains. The most potent bNAbs, and not the nnAbs, bound to reactivated infected cells from HIV-positive individuals and mediated effective ADCC against those cells. The nnAbs also modestly recognize cells infected with eight different transmitted founder (T/F) isolates. Efficient ADCC requires sustained cell surface binding of bNAbs to Env proteins, and combining bNAbs allows a potent killing activity. Addition of a synthetic CD4 mimetic enhanced the binding and killing efficacy of some of the nnAbs in an epitope-dependent manner, without reaching the levels achieved by the most potent bNAbs.

**Conclusions**: Our study reveals important qualitative and quantitative differences between bNAbs and nnAbs, delineates the parameters controlling ADCC activity of bNAbs and supports the use of the most potent antibodies to clear the viral reservoir.

## MOAA0206

### Allosteric regulation in human anti-HIV-1 Env ADCC-mediating antibodies upon immune complex (IC) formation enhances the binding to FcγRs for the activation of cytotoxicity against HIV-1 virus

C Orlandi ^1^; D Deredge^2^; K Ray^1^; N Gohain^1^; W Tolbert^1^; M Pazgier^1^; A Devico^1^; P Wintrode^2^ and G Lewis^1^

^1^Human Virology Institute, University of Maryland - School of Medicine, Baltimore, USA. ^2^Department of Pharmaceutical Sciences, University of Maryland, School of Pharmacy, Baltimore, USA

Presenting author email: corlandi@ihv.umaryland.edu

**Background**: The field of HIV-1 vaccine research is in rapid development, and a deeper knowledge of the mechanisms of defence against HIV-1 infection is urgent. In this regard, key insights about antibody-mediated immune response were provided by the RV144 vaccine trial that showed moderate protection correlating with Fc-effector functions. Historically, the activation of antibody-dependent cellular cytotoxicity (ADCC) has been considered dictated by two distinct and critical steps: optimal binding of viral antigens and engagement of FcγRs, respectively, occurring in two distinguished mAb regions (Fab and Fc domains). Only recently, few studies suggested a functional connection of Fab and Fc regions within the IgG.

**Methods**: Utilizing multiple approaches, such as ELISA, fluorescence correlation spectroscopy, hydrogen/deuterium exchange mass spectroscopy (H/DX MS) and crystallography, we studied whether the immune complex (IC) formation by antigen (Ag) binding may impact the ability of IgG to consequently engage FcγRs and, in turn, activate the cytotoxic immune response against HIV-1-sensitized targets.

**Results**: Here, we demonstrate a reciprocal allosteric regulation in anti-HIV gp120 Cluster A mAbs resulting from IC formation with a gp120-CD4 chimera antigen. We establish that the formation of ICs dramatically increases the efficiency of mAbs interaction to low-affinity FcγRs compared to free IgG. Moreover, antigen binding reverses the effect of FcγRs binding-attenuating LALA mutations in the Fc region, resulting in residual ADCC activity. H/DX MS analysis reveals an allosteric increase in conformational dynamics in the Fc domain of wildtype IgG upon Ag binding, highlighting a putative mechanism for effective Fc receptor engagement. In line with crystallographic data, H/DX MS showed a higher flexibility in the LALA mutant. However, antigen binding further stabilized Fab and Fc regions of LALA mAbs, maintaining an adequate level of flexibility in the same crucial residues observed in the wt-IgG–Ag complex. Of note, the more mobile residues map to non-contact regions for FcγRs but include residues that indirectly affect the IgG–FcγRs affinity and the induction of IgG multimerization.

**Conclusions**: Collectively, these findings provide unique insights into reciprocal allosteric regulation between Fab and Fc domains, as IgG intrinsic factors that dictate the ability to tune selective Fc-effector functions, such as ADCC, in vaccine regimens or HIV-1 passive treatments.

## MOAB0101

### Immunodeficiency at the start of combination antiretroviral therapy in low-, middle- and high-income countries

N Anderegg ^1^; O Kirk^2^; for the IeDEA & COHERE collaborations

^1^Institute of Social and Preventive Medicine, University of Bern, Bern, Switzerland. ^2^CHIP, Centre for Health & Infectious Diseases Research, Department of Infectious Diseases, University of Copenhagen, Rigshospitalet, Copenhagen, Denmark

Presenting author email: nanina.anderegg@ispm.unibe.ch

**Background**: Early initiation of combination antiretroviral therapy (cART), at higher CD4 cell counts, prevents disease progression and reduces sexual transmission of HIV. Changes in guidelines are expected to result in increased CD4 cell counts at cART start. We describe temporal trends in the median CD4 cell count at cART start in adult men and women.

**Methods**: We used data from the International epidemiology Databases to Evaluate AIDS (IeDEA) sub-Saharan Africa, Latin America, Asia-Pacific and North America regions and from the Collaboration of Observational HIV Epidemiological Research in Europe (COHERE). We included all HIV-positive adults (≥16 years) initiating cART between 2002 and 2015. We aggregated data by calendar year, country and sex and calculated median CD4 cell counts for each of the data cells. We used additive mixed models to analyse temporal trends in median CD4 cell counts. Sex, country income group and their interaction were included as fixed effects, and yearly trends were smoothed by sex and country income group.

**Results**: We included 652,728 adults from 14 low-, 11 lower middle-, 6 upper middle- and 17 high-income countries. Figure 1 shows the modelled median CD4 cell count (cells/µl): from 2002 to 2015, there was an increase from 66 to 243 (+268%) in low-, from 88 to 170 (+93%) in lower middle-, from 69 to 257 (+272%) in upper middle- and from 163 to 355 (+118%) in high-income countries in males; and from 78 to 309 (+296%) in low-, 118 to 264 (+124%) in lower middle-, 70 to 328 (+369%) in upper middle- and 170 to 302 (+78%) in high-income countries in females.

**Conclusions**: Median CD4 cell count at cART start increased in all income groups but generally remained below 350 cells/µl. Substantial additional efforts are needed to increase the testing coverage with the aim of achieving earlier diagnosis, linkage and initiation of cART globally.

**Abstract MOAB0101–Figure 1.**
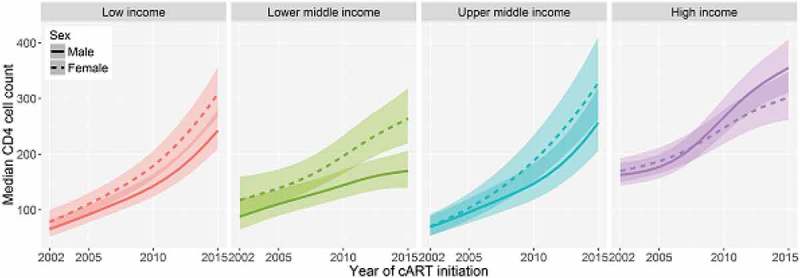
Modelled trends in CD4 cell count (cell/μl) at the start of cART, by sex and country income group.

## MOAB0102

### ANRS 146 - GeSIDA 7211 OPTIMAL phase III trial: maraviroc plus cART in advanced HIV-1-infected individuals

Y Levy^1,2^; J-D Lelièvre ^1,2^; L Assoumou^3^; E Aznar^4^; F Pulido^5^; G Tambussi^6^; M Crespo^7^; A Meybeck^8^; J-M Molina^9^; F Cardon^10^; A Diallo^10^; C Delaugerre^9^; R Lancar^3^; L Béniguel^3^; D Costagliola^3^; on behalf of the ANRS 146 - GeSIDA 7211 study group

^1^VRI/Inserm, U955, Equipe 16; Université Paris Est, Faculté de médecine, Créteil, France. ^2^APHP, Hôpital H. Mondor, Créteil, France. ^3^Sorbonne Universités, INSERM, UPMC Univ Paris 06, Institut Pierre Louis d’épidémiologie et de Santé Publique (IPLESP UMRS 1136), Paris, France. ^4^Fundación SEIMC-GESIDA, Madrid, Spain. ^5^Hospital 12 de Octubre, Madrid, Spain. ^6^IRCCS-Ospedale San Raffaele, Milano, Italy. ^7^Hospital universitario Vall d’Hebron, Barcelona, Spain. ^8^Service Universitaire des Maladies Infectieuses et du Voyageur, Centre Hospitalier de Tourcoing, Tourcoing, France. ^9^AP-HP, Hôpital Saint Louis, Paris, France. ^10^ANRS, France Recherche Nord & Sud Sida-HIV Hépatites, Paris, France

**Background**: Late HIV diagnosis is associated with an excess risk of AIDS-defining events (ADE) and mortality. We hypothesized that - due to its immunomodulating effect - the addition of maraviroc (MVC) to cART in naive patients with low CD4 cell counts will decrease the risk of disease progression and death.

**Methods**: The ANRS 146 OPTIMAL trial (NCT01348308) was a European, multicentre, randomized, double-blind phase III trial, in France, Spain and Italy, in ART-naive HIV-1-infected adults with CD4+ count <200/µl or an ADE. Participants were randomized (1:1) to receive cART plus placebo or MVC for 72 weeks. The primary composite endpoint was any new ADE, serious non-AIDS-defining event, IRIS or death from any cause. The primary endpoint and its components were compared using Kaplan–Meier estimates and Cox proportional hazards models. In a *post* *hoc* analysis, a Poisson regression model was used to analyse occurrence of all events and the interaction between the study period (0–24 versus 24–72 weeks) and the treatment effect.

**Results**: Between October 2011 and November 2014, 409 patients were included. At baseline, median HIV viral load was 5.39 log_10_ copies/ml, median CD4+ count was 80 cells/µl and 42% of participants had an ADE. No difference was seen in CD4 cell increase (+258.3 ± 8.9 vs. +254.2 ± 9.2/µl) (*p* = 0.746). Seventy-four events occurred in 53 participants: 42 events in 27 participants in the placebo group and 32 events in 26 participants in the MVC group. The incidence of the first event was 11.2 events per 100 person-years in the placebo group versus 11.1 events per 100 person-years in the MVC group with a hazard ratio of 0.97 (95% confidence interval (CI): 0.57–1.67). Poisson regression analysis showed that the incidence-rate-ratio (IRR) of the two groups differed significantly between periods 0–24 and 24–72 weeks with respective IRR of 0.61 (95% CI: 0.33–1.08) and 2.90 (95% CI: 0.86–12.49) (*p* = 0.016).

**Conclusions**: The results of this large randomized trial showed that adding MVC to cART does not impact the occurrence of serious disease or death in advanced HIV-1-positive patients. However, *post hoc* analysis showed a trend for a beneficial effect of the addition of MVC in the first 24 weeks that disappeared thereafter.

## MOAB0103

### Safety, efficacy and dose–response of GSK3532795/BMS-955176 plus tenofovir/emtricitabine (TDF/FTC) in treatment-naive (TN) HIV-1-infected adults: week 24 primary analysis

J Morales-Ramirez^1^; J Bogner^2^; J-M Molina^3^; J Lombaard^4^; I Dicker^5^; D Stock^6^; S Min^7^; C Llamoso^5^; SR Joshi^5^ and M Lataillade ^5^

^1^Clinical Research Puerto Rico Inc, San Juan, Puerto Rico. ^2^Med IV, Hospital of the University of Munich, Munich, Germany. ^3^Hôpital St Louis, Paris, France. ^4^Josha Research, Bloemfontein, South Africa. ^5^ViiV Healthcare, Wallingford, USA. ^6^Bristol-Myers Squibb, Wallingford, USA. ^7^ViiV Healthcare, Research Triangle Park, USA

Presenting author email: max.x.lataillade@viivhealthcare.com

**Background**: This Phase 2b study investigated the safety, efficacy and dose–response of GSK3532795 (formerly BMS-955176), a novel second-generation MI, relative to efavirenz (EFV) in treatment-naive (TN), HIV-1-infected subjects.

**Methods**: AI468038 (205891) is a global, randomized, doubled-blind active-controlled trial. TN adults, with HIV-1 RNA ≥1000 c/ml and susceptibility to commercially available study medications, were randomized 1:1:1:1 to 60, 120 or 180mg of GSK3532795 or EFV 600mg once-daily with TDF/FTC. The primary endpoint was the proportion of subjects with HIV-1 RNA <40 c/ml at week 24 using the FDA Snapshot algorithm.

**Results**: A total of 210 subjects were randomized and 206 were treated. The mean age was 33.7 years; 85.4% were male. The mean baseline HIV-1 RNA and CD4+ T cell counts were 4.3 log_10_ c/ml (17% >100,000 c/ml) and 444 cells/µl (5.8% <200 cells/µl), respectively. At week 24, 76–82.7% of subjects across the GSK3532795 arms (60, 120 and 180mg) and 77.4% in the EFV arm achieved an HIV-1 RNA <40 c/ml (mITT, FDA snapshot). More EFV (17%) than GSK3532795 (2–8%) subjects reported AEs leading to discontinuation. Few subjects reported SAEs on EFV (9%) or GSK3532795 (2–4%). The rates of GI AEs, predominately diarrhoea and abdominal pain (all grades, regardless of relationship), in the GSK3532795 arms were 52–72.5%; the EFV rate was 24.5%. The rate of treatment-emergent NRTI resistance in the GSK3532795 arms was 6.5%. The EFV arm did not contain any treatment-emergent NRTI/NNRTI resistance.

**Abstract MOAB0103–Table 1.** Week 24 efficacy data.

Parameter, *n* (%)	GSK3532795 60mg + TDF/FTC QD	GSK3532795 120mg + TDF/FTC QD	GSK3532795 180mg + TDF/FTC QD	EFV 600mg + TDF/FTC
**Week 24 snapshot (mITT)**	(*N* = 50)	(*N* = 52)	(*N* = 51)	(*N* = 53)
HIV-1 RNA <40 c/ml	38 (76.0)	43 (82.7)	42 (82.4)	41 (77.4)
HIV-1 RNA ≥40 c/ml	10 (20.0)	7 (13.5)	4 (7.8)	3 (5.7)
No virologic data at week 24	2 (4.0)	2 (3.8)	5 (9.8)	9 (17.0)
**Week 24 (observed sata)**	(*N* = 46)	(*N* = 47)	(*N* = 45)	(*N* = 44)
HIV-1 RNA <40 c/ml	38 (82.6)	43 (91.5)	42 (93.3)	41 (93.2)

**Conclusions**: The week 24 primary endpoint showed similar efficacy results across GSK3532795 treatment arms relative to EFV when combined with TDF/FTC in TN HIV-1-infected adults. However, the GSK3532795 arms showed a higher rate of GI intolerability and treatment-emergent resistance to the NRTI backbone relative to EFV.

## MOAB0104

### Forty-eight-week efficacy of a third line based on darunavir plus raltegravir regimen in HIV-infected adults who failed second-line protease inhibitor-based regimen in sub-Saharan Africa, ANRS 12269 THILAO study

R Moh ^1,2^; A Benalycherif^3^; D Gabillard^4^; J Lecarrou^4^; L N’guessan^2^; A Anzian^5^; D Minta^6^; A Sawadogo^7^; M Seydi^8^; J Drabo^9^; M-L Chaix^10^; P-M Girard^11^; C Danel^2,4^; X Anglaret^2,4^; S Eholié^2,12^; R Landman^3,13^; Thilao Study Group

^1^Département de Dermatologie et Infectiologie, Abidjan, Cote D’Ivoire. ^2^Programme PACCI/Site ANRS de Côte d’Ivoire, Abidjan, Cote D’Ivoire. ^3^Institut de Médecine et d’Epidémiologie Appliquée, Hopital Bichat, Claude Bernard, Paris, France. ^4^INSERM U1219, ISPED, Université Bordeaux 2, Bordeaux, France. ^5^Centre de Prise en charge, de Recherche et de Formation Abidjan, Abidjan, Cote D’Ivoire. ^6^Service des Maladies Infectieuses et Tropicales, CHU Point G, Abidjan, Cote D’Ivoire. ^7^Hôpital de jour, Bobo-Dioulasso, Bobo-Dioulasso, Burkina Faso. ^8^SMIT/CRCF, Dakar, Dakar, Senegal. ^9^Hopital Yalgado, Ouagadoudou, Ouagadougou, Burkina Faso. ^10^Service de Virologie, St Louis, Paris, France. ^11^Service des Maladies Infectieuses et Tropicales, Hôpital St Antoine, Paris, France. ^12^Département de dermatologie et d’infectiologie, UFR Sciences médicales, Abidjan, Cote D’Ivoire. ^13^INSERM, IAME, UMR 1137, Paris, France

Presenting author email: rmoh73@gmail.com

**Background**: Tolerance and efficacy data on third-line treatment are scarce in sub-Saharan Africa, in a context where viral load and genotype test are limited. This study aims to assess the effectiveness of 48-week third-line therapy after three-month adherence reinforcement in HIV-infected adults who failed second-line antiretroviral therapy (ART).

**Methods**: Thilao is a cohort study conducted in Burkina Faso, Côte d’Ivoire, Mali and Senegal. HIV-1-infected adults with virological failure on a second-line protease inhibitor-based regimen after a first-line non-nucleoside reverse-transcriptase inhibitor were included. Adherence reinforcement measures were proposed at baseline (V0). After three months (V3), a viral load (VL) was performed. The second-line therapy was maintained if VL from baseline to V3 decreases by more than 2 log or below 400 copies/ml and a switch to a third-line darunavir/r 600/100mg BID (DRVr) and raltegravir 400mg BID (RAL) regimen in case of virological failure without knowing genotypic resistance test results. Each patient was followed 64 weeks (W64) and 48 weeks (W48) after third-line initiation. We describe the virological outcomes at W48 for those initiated third-line therapy.

**Results**: A total of 201 patients were included, women: 69%, median age: 41 years old (35–48). Median CD4 count and VL at pre-inclusion were 242/mm^3^ (113–400) and 4.5 log/ml (3.0–5.0); median duration since ART initiation: 8 years (6–10) including median 3 years (2–6) of second-line protease inhibitor. The median of medication possession ratio between W0 and W64 was 97.1 (91.4–100.2). After V3, 34% have initiated third-line ART. Among them, 64% received TDF 3TC/FTC-NNRTI regimen. At W48: 4 are deceased and 0 were lost-to-follow up; 62% had a VL <50 copies/ml. The median of those with a detectable VL was 896 copies (176–24500). The probability of eventually initiating a third-line therapy during follow-up at W64 was 39%. No severe adverse event related to third-line therapy was notified. Among patients who failed DRVr and RAL regimen (*n* = 11), only one mutation E157q to RAL was detected.

**Conclusions**: Sequencing of third-line regimen such as recommended by the WHO based on darunavir/r, raltegravir and recycled NRTI is well tolerated and efficient as salvage therapy.

## MOAB0201

### Maximizing targeted testing to improve HIV yield among children and adolescents in Rwenzori region, Uganda

H Bitimwine ^1^; F Musiime^2^; P Ajuna^2^; P Tumbu^2^; P Nahirya-Ntege^2^ and A Kekitiinwa^2^

^1^Baylor College of Medicine Children’s Foundation, Medical & Psychosocial, Kampala, Uganda. ^2^Baylor College of Medicine Children’s Foundation, Kampala, Uganda

Presenting author email: bitimwine@gmail.com

**Background**: Ugandan national antiretroviral therapy (ART) coverage among children (<15 years) is low, at 42% (61,642/147,394) compared with the target of 90%. This arises from the low identification of HIV-infected children and adolescents through HIV testing service (HTS) models, such as routine counselling and testing. In Rwenzori region, HIV testing yield is low for children (0.7%) and adolescents (1%). Baylor-Uganda implemented targeted HTS models to improve HIV yield among children and adolescents and thus close the ART gap in the Rwenzori region. We determined the HIV yield from these models.

**Methods**: In the period of March–June 2016, we provided HTS to children and adolescents 18 months to 19 years using the following models: HTS outreaches to dwelling homes of orphans and vulnerable children (OVC); Know Your child Status Campaigns (KYCS); HTS outreaches to children of female sex workers (FSWs), fisher folks (FFs) and tea plantation workers; and evening HTS points targeting adolescents after work/school hours. We summarized the HIV yield for the different models in proportions and frequencies.

**Results**: Of the 4091 children and adolescents tested, 2135 (52%) were females and 2030 (50%) adolescents (10–19 years). The overall HIV yield from all models was 53/4091 (1.3%). The HIV yield among adolescents, children (5–9 years) and those under 5 years was 30/2030 (1.5%), 20/1234 (1.6%) and 3/824 (0.4%), respectively. It was highest through outreaches at OVC dwelling homes 7/271 (2.6%) and lowest through outreaches to children of tea plantation workers 0/214 (0%). The HIV yield through outreaches to children of FSWs, children of FFs, KYCS campaigns and evening HTS points was 10/610 (1.6%), 3/283 (1.1%), 16/836 (0.9 %) and 14/815 (1.7%), respectively.

**Conclusions**: A relatively high HIV yield was achieved through HTS at OVC dwelling homes, children of FSWs, FFs and Evening HTS and a low yield through outreaches to children of tea plantation workers and KYCS campaign. Therefore, deliberate efforts should be made to scale up HTS to OVC dwelling homes, children of FSWs and fisher folks and evening HTS for adolescents and consider to discontinue HTS outreaches to children of tea plantation workers and scale down or modify KYCS campaigns.

## MOAB0202

### Impacts of vitamin D and calcium supplementation on bone mineral density among perinatally HIV-infected adolescents: a 48-week randomized clinical trial

T Sudjaritruk ^1,2^; L Aurpibul^2^; T Bunupuradah^3^; S Kanjanavanit^4^; T Chotecharoentanan^2^; S Taejaroenkul^2^; P Ounchanum^5^; P Suntarattiwong^6^; T Puthanakit^3,7,8^; CAL-D Study Group

^1^Department of Pediatrics, Faculty of Medicine, Chiang Mai University, Chiang Mai, Thailand. ^2^Research Institute for Health Sciences, Chiang Mai University, Chiang Mai, Thailand. ^3^HIV-NAT, The Thai Red Cross AIDS Research Centre, Bangkok, Thailand. ^4^Nakornping Hospital, Chiang Mai, Thailand. ^5^Chiangrai Prachanukroh Hospital, Chiang Rai, Thailand. ^6^Queen Sirikit National Institute of Child Health, Bangkok, Thailand. ^7^Department of Pediatrics, Faculty of Medicine, Chulalongkorn University, Bangkok, Thailand. ^8^Research Unit in Pediatric Infectious Diseases and Vaccines, Chulalongkorn University, Bangkok, Thailand

Presenting author email: tavitiya.s@cmu.ac.th

**Background**: This study aimed to identify the impacts of vitamin D and calcium (VitD/Ca) supplementation on bone mineral density (BMD) and bone metabolism among perinatally HIV-infected Thai adolescents.

**Methods**: An ongoing, randomized open-label trial has been conducted. Adolescents aged 10–20 years who were stable on ART (HIV RNA <400 copies/ml) were randomly assigned to receive either “high-dose”: VitD/Ca (3200 IU/1.2 g daily) or “normal-dose”: VitD/Ca (400 IU/1.2 g daily) supplementation for 48 weeks. Lumbar spine BMD and bone metabolism-related markers were evaluated at baseline and 48 weeks. BMD was measured by dual-energy X-ray absorptiometry, of which *z*-score ≤−2 was defined as low BMD. Bone metabolism-related markers included 25-hydroxyvitamin D (25OHD), intact parathyroid hormone (iPTH), alkaline phosphatase (ALP), C-terminal telopeptide (CTX-bone resorption marker) and procollagen type I amino-terminal propeptide (PINP-bone formation marker). An interim analysis, stratified by baseline BMD *z*-score ≤−2 (low BMD) vs. >−2 (normal BMD), was performed using the intention-to-treat analysis.

**Results**: Between April 2015 and October 2016, 166 adolescents were enrolled. The median age and ART duration were 16.0 and 10.0 years, respectively. The median baseline BMD *z*-score was −1.5, and 67 adolescents (40%) had low BMD. Overall adherence to VitD/Ca supplementation was 80%. At week 48, there was a significant increase in BMD *z*-scores in participants with low baseline BMD, particularly among those receiving “high-dose” compared with “normal-dose” supplementation (+0.74 vs. +0.49) (Table 1). The increased 25OHD and the declined iPTH, ALP, CTX and PINP levels were also observed in both treatment groups (*p *< 0.001). No between-group differences in changes from baseline for BMD *z*-scores and all bone biomarkers (*p *> 0.05), except for iPTH (*p *= 0.007).

**Abstract MOAB0202–Table 1.** Changes of bone mineral density *z*-scores and bone metabolism-related biochemical markers from baseline among 166 perinatally HIV-infected Thai adolescents over the 45-week study follow-up.

**Conclusions**: With the preliminary results, BMD was significantly ameliorated in adolescents with low baseline BMD who received VitD/Ca supplementation, regardless of dose, over 48-week follow-up. A prospective study with longer follow-up is warranted to confirm our findings.

## MOAB0203

### Inequality in mortality and access to antiretroviral therapy in adolescents living with perinatally acquired HIV in sub-Saharan Africa: a Collaborative Initiative for Paediatric HIV Education and Research (CIPHER) cohort collaboration analysis

A Slogrove ^1^; V Leroy^2^; A Judd^3^; CIPHER Global Cohort Collaboration Adolescent Project Team

^1^University of Cape Town, Centre for Infectious Disease and Epidemiologic Research, Cape Town, South Africa. ^2^Universite Paul Sabatier Toulouse 3, INSERM U1027, Toulouse, France. ^3^University College London, MRC Clinical Trials Unit, London, UK

Presenting author email: slogrove@gmail.com

**Background**: Eighty per cent of adolescents living with perinatally and behaviourally acquired HIV live in sub-Saharan Africa (SSA), a continent with marked economic inequality. Extending our previous global description of adolescents living with perinatally acquired HIV (APH), this analysis aimed to describe APH outcomes in SSA by country income group (CIG).

**Methods**: Through the CIPHER cohort collaboration, individual retrospective data from 12 cohort networks across 5 continents were pooled; 7 networks representing SSA were included here. APH included were HIV-infected children with entry into care at age <10 years (proxy for perinatally acquired HIV) and follow-up at age >10 years. CIG was classified according to World Bank Classification at median year of first visit by country. Cumulative incidence functions were calculated by competing risk analysis for mortality, transfer-out and loss-to-follow-up.

**Results**: A total of 30,296 APH were included; 75.7% resident in low-income countries (LIC), 4.6% in lower-middle-income countries (LMIC) and 19.8% in upper-middle-income countries (UMIC); 64% of APH were born ≥2000. Median (interquartile range [IQR]) age at antiretroviral therapy (ART) start (8 [6;9] years) and at last follow-up (12 [11;14] years) was equivalent across CIG. About 26,018 (85.9%) ever started ART and 3352 (12.5%) started at age >10 years, both significantly different between CIG (*p* < 0.001) (Table 1). Individual CD4 count improved between ART start and last visit in all CIG (*p* < 0.001). Half of APH had height-for-age *z*-score (HAZ) <−2 at ART start that improved by last visit in LIC (*p* < 0.001) and UMIC (*p* < 0.001) but not in LMIC (*p* = 0.18). Mortality between age 10 and 15 years was lowest in UMIC; however, loss-to-follow-up was highest in UMIC.

**MOAB0203 Table 1.** APH characteristics by CIG (*N* = 30,296).

	LIC *N* = 22,925	LMIC *N* = 1386	UMIC *N* = 5985
Ever started ART, *n* (%)	19,114 (83.4)	1207 (87.1)	5697 (95.2)
Started ART age >10 years, *n* (%)	2829 (14.8)	141 (11.7)	382 (6.7)
CD4 count (cells/µl) at ART start, median [IQR] (*N* = 15,254)	310 [165; 520]	292 [174; 417]	318 [162; 558]
CD4 count (cells/µl) at last visit, median [IQR] (*N* = 24,223)	668 [434; 945]	735 [532; 985]	729 [513; 971]
HAZ at ART start, median [IQR] (*N* = 16,181)	−2.01 [−2.97; −1.08]	−2.08 [−2.95; −1.33]	−2.02 [−2.86; −1.17]
HAZ at last visit, median [IQR] (*N* = 25,333)	−1.77 [−2.60; −0.95]	−2.02 [−2.77; −1.30]	−1.54 [−2.31; −0.77]
Cumulative incidence of mortality, % (95% CI)	3.5 (3.1; 3.8)	3.9 (2.7; 5.4)	1.1 (0.8; 1.4)
Cumulative incidence of transfer-out, % (95% CI)	17.5 (16.8; 18.3)	27.5 (24.2; 31.0)	23.7 (22.4; 25.1)
Cumulative incidence of loss-to-follow-up, % (95% CI)	13.1 (12.4; 13.8)	8.3 (6.3; 10.6)	14.0 (12.9; 15.3)

**Conclusions**: Despite starting ART late, improvements in height and CD4 count were observed in most APH surviving to adolescence. Mortality rates are likely underestimated. However, results highlight inequalities in mortality and access to ART according to CIG in SSA.

## MOAB0204

### Evaluation of the risk of birth defects among children exposed to raltegravir *in utero* in the ANRS-French Perinatal Cohort EPF

J Sibiude ^1,2^; J Warszawski^1,3^; S Blanche^4,5^; O Dialla^1^; A Faye^6,7^; C Dollfus^8^; L Mandelbrot^1,7,9^; R Tubiana^10,11^; for the ANRS-EPF CO1/CO11 Study Group

^1^INSERM, CESP 1018, Le Kremlin Bicêtre, France. ^2^APHP-Hôpital Cochin Port Royal, Paris, France. ^3^Université Paris Sud, Le Kremlin Bicêtre, France. ^4^APHP Hôpital Necker, Paris, France. ^5^Université Paris Descartes, Paris, France. ^6^APHP Hôpital Robert Debré, Paris, France. ^7^Université Paris Diderot, Paris, France. ^8^APHP Hôpital Trousseau, Paris, France. ^9^APHP Hôpital Louis Mourier, Colombes, France. ^10^APHP Hôpital Pitié Salpétrière, Paris, France. ^11^Sorbonne Universités, UPMC Université Paris 6, Paris, France

Presenting author email: jeanne.sibiude@gmail.com

**Background**: Raltegravir is an integrase inhibitor, largely used in the recent years, but tolerance data in pregnancy are scarce. Potential teratogenicity has not yet been evaluated for this molecule in a clinical context. We aimed to describe the rates and types of birth defects among children exposed to raltegravir *in utero* and to study the association with trimester of exposure.

**Methods**: EPF is a multicentre national cohort, which prospectively enrols pregnant HIV-infected women delivering in 90 centres throughout France. Children are followed by paediatricians until two years of age. All births exposed to raltegravir were included. Birth defects were defined using the EUROCAT classification. Rates of birth defects were compared according to timing of exposure to raltegravir (first trimester vs. second and third trimesters) using *χ* ^2^ tests.

**Results**: We included 479 foetuses born between 2008 and 2015, exposed to raltegravir, among which 6 stillbirths (1.3%) and 2 late miscarriages (0.4%). There were no terminations of pregnancies for birth defects. Rates of birth defects were 4.2% for all births (20/479 [95% confidence interval 2.4–6.0]) and 4.2% among live births (20/471 [2.4–6.1]). This incidence was similar to that reported in a previous study in EPF for live births exposed to any ARV (4.4% [4.0–4.7]). Birth defect rates did not differ significantly between first-trimester exposure to raltegravir (5.7%; 8/140) and second- or third-trimester exposure (3.5%; 12/339; *p* = 0.32). The anomalies did not follow any pattern and concerned various organs: seven heart defects, five polydactylies and eight other defects. Other notable adverse outcomes were preterm births (14.2%), and two cases of HIV perinatal infection (0.4%). The follow-up was complete to 24 months for 63% of children. For 15% of children, only the birth questionnaire was available.

**Conclusions**: This is the largest prospective cohort of children exposed *in utero* to raltegravir with homogenous evaluation of birth defects. We did not find a significant association between first-trimester exposure to raltegravir and birth defects. This finding is quite reassuring as this molecule is often prescribed to women of child-bearing age, and thus, many children may be exposed in the first trimester of pregnancy.

## MOAB0205

### High prevalence of respiratory non-tuberculous mycobacteria respiratory infections in children living with HIV in South-East Asia

L Borand ^1^; A de Lauzanne^1^; M Inghammar^1,2^; V Ung^3^; S Cheng^4^; TH Pham^5^; P Msellati^6^; M Tejiokem^7^; A-S Ouedraogo^8^; S Godreuil^9^; C Delacourt^10^; S Blanche^11^; O Marcy^1,12^; ANRS 12229 PAANTHER 01 Study Group

^1^Institut Pasteur du Cambodge, Epidemiology and Public Health Unit, Phnom Penh, Cambodia. ^2^Department of Clinical Sciences, Section for Infection Medicine, Lund University, Lund, Sweden. ^3^University of Health Sciences, Planning and Research Faculty of Medicine, Phnom Penh, Cambodia. ^4^Institut Pasteur du Cambodge, Mycobacteriology Laboratory, Phnom Penh, Cambodia. ^5^Microbiology Department, Pham Ngoc Thach Hospital, Ho Chi Minh City, Vietnam. ^6^IRD, UMI 233 - TransVIHMI, Abidjan, Cote D’Ivoire. ^7^Centre Pasteur du Cameroun, Member of the Institut Pasteur International Network, Epidemiology and Public Health Service, Yaounde, Cameroon. ^8^CHU-Souro-Sanou, Département des laboratoires, Bobo-Dioulasso, Burkina Faso. ^9^CHU Montpellier, Bacteriology, Montpellier, France. ^10^Necker - Enfants Malades Hospital - APHP, Pneumology, Paris, France. ^11^Necker - Enfants Malades Hospital - APHP, Pediatric Hematology-Immunology, Paris, France. ^12^Université de Bordeaux, Bordeaux Population Health - Centre INSERM U1219, Bordeaux, France

Presenting author email: lborand@pasteur-kh.org

**Background**: Data on burden of non-tuberculous mycobacteria (NTM) and related pulmonary diseases are limited in HIV-infected children in developing countries. We investigated NTM respiratory infection (RI) prevalence, species distribution and associated factors in HIV-infected children with a suspicion of tuberculosis in four countries in South-East Asia and Africa.

**Methods**: From 2011 to 2014, HIV-infected children ≤13 years with a suspicion of tuberculosis were included in the ANRS 12229-PAANTHER 01 study in Burkina-Faso, Cambodia, Cameroun and Vietnam after parental consent. Children underwent collection of respiratory and stool samples for mycobacterial culture and molecular identification of species. Children with ≥1 analysable sample in culture were included. NTM-RI was defined as ≥1 sample culture positive for any NTM. Logistic regression models were used to identify factors associated with respiratory NTM or *Mycobacterium avium* complex (MAC) infections.

**Results**: Of 438 children enrolled, 427 had ≥1 analysable sample. Median age was 7.3 years, with 212 (49.7%) male, 245 (57.4%) Asian, 267 (63.9%) underweight, 212 (51.1%) severely immunodepressed and 258 (60.4%) ART naive. Prevalence of culture-confirmed tuberculosis was 13% (55/427), including 5 co-infections *tuberculosis*/NTM. Prevalence of NTM-RI was 10.8% (46/427), 16.7% (41/245) and 2.8% (5/177) in all, Asian and African children, respectively. MAC was isolated in 21/427 (5%) children overall and 17/125 (13.6%) children from Asian origin with severe immune-depression (CDC classification 2014). Majority of NTM patients with severe immune-depression were infected by MAC (*n* = 17/19). In contrast, *Mycobacterium fortuitum, M. scrofulaceum*, *M*. *interjectum* and *M. gordonae were the most frequent* species in non- or moderately immunodepressed children. Overall, South-East Asian origin (odds ratio (OR) 7.2; 95% confidence interval (CI) 2.5–21.1), age 5–9  years compared to 0–2 years (OR 10.1; 95% CI 2.3–44.8) and severe immune-deficit (OR 3.3; 95% CI 1.5–7.2) were factors independently associated with NTM-RI. CD4-T lymphocyte count <50/mm^3^ (OR 9.8; 95% CI 3.6–26.5) and Asian origin (OR 16.5; 95% CI 2.2–126.1) were independently associated with MAC infection.

**Conclusions**: NTM-RIs are frequent in HIV-infected children with presumptive tuberculosis in South-East Asia, not only as opportunistic infection in severe immunodeficiency. NTM contribution to lung disease is unclear in a tuberculosis suspicion context. Empiric treatment for both tuberculosis and MAC may be relevant in most severely immunodepressed HIV children suspected of tuberculosis in South-East Asia.

## MOAB0206

### High-risk vaccine-specific HPV infection in HIV-infected and HIV-uninfected, vaccine-naive Asian female adolescents

S Sricharoenchai ^1^; T Bunupuradah^2^; R Hansudewechakul^3^; DLD Hanh^4^; DNH Tran^5^; S Kerr^2^; A Chalermchockcharoenkit^1^; N Teeratakulpisarn^2^; J Achalapong^3^; DNY Dung^4^; W Termrungruanglert^6^; S Chaithongwongwatthana^6^; T Singtoroj^7^; T Pankam^8^; N Phanuphak^8^; AH Sohn^7^; K Chokephaibulkit^1^; HPV in Adolescents Study team

^1^Faculty of Medicine Siriraj Hospital, Mahidol University, Bangkok, Thailand. ^2^HIV-NAT, The Thai Red Cross AIDS Research Centre, Bangkok, Thailand. ^3^Chiangrai Prachanukroh Hospital, Chiang Rai, Thailand. ^4^Hung Vuong Hospital, Ho Chi Minh City, Vietnam. ^5^Children’s Hospital 1, Ho Chi Minh City, Vietnam. ^6^Faculty of Medicine, Chulalongkorn University, Bangkok, Thailand. ^7^TREAT Asia/amfAR - The Foundation for AIDS Research, Bangkok, Thailand. ^8^Thai Red Cross AIDS Research Centre, Bangkok, Thailand

Presenting author email: sirintipsri@gmail.com

**Background**: Adolescents in resource-limited countries have limited access to human papillomavirus (HPV) vaccination. We assessed the prevalence of seven high-risk vaccine-specific HPV types (HRVS-7) in the nonavalent vaccine and identify factors associated with oral and anogenital HRVS-7 infection in perinatally HIV-infected female adolescents (PHIVA) and HIV-uninfected females (controls).

**Methods**: Sexually active PHIVA were enrolled in a prospective study in Thailand and Vietnam from 2013 to 2015. Controls were matched by age and lifetime number of sexual partners. All participants were naive to HPV vaccination. HPV genotypic assay was performed on oral, anal, cervical and vaginal samples using the Roche Linear Array, and serum neutralizing antibodies (NAb) to HPV types 16 and 18 were measured. An audio-computer-assisted self-interview (ACASI) was performed to assess behavioural risks. Screening for sexually transmitted infections (STIs) included *Chlamydia trachomatis, Neisseria gonorrheae* and herpes simplex virus-2 (HSV2) infections and syphilis. Multiple logistic regression analysis was used to assess factors associated with presence of any HRVS-7 genotypes (16, 18, 31, 33, 45, 52 and 58) at any body sites.

**Results**: A total of 93 PHIVA and 99 controls were enrolled; median (IQR) age 19 (18–20) years, with 2 (1–3) lifetime sexual partners. At enrolment, median CD4 among PHIVA was 593 (392–808) cells/mm^3^, and 62% had HIV-RNA <40 copies/ml. HRVS-7 genotypes were found in 43 (46%) PHIVA and 39 (39%) controls (*p *= 0.3). NAbs were detected in 19 (22%) PHIVA and 26 (28%) controls (*p *= 0.3). For the complete cohort, 20/33 (61%) adolescents 13–16 years, 50/105 (48%) 17–19 years and 28/54 (52%) 20–24 years were positive for HRVS-7 DNA or NAb. A history of ≥3 lifetime partners (vs. 1; odds ratio (OR) 3.34 [1.59–7.04]) and having any non-HPV STI (OR 3.39 [1.56–7.39]) were independently associated with increased risk of infection with an HRVS-7 genotype. Among PHIVA, HIV-RNA >40 copies/ml and having any non-HPV STI were independently associated with increased risk of infection with HRVS-7.

**Conclusions**: Half of Asian PHIVA and HIV-uninfected female adolescents in our cohort had high-risk HPV infection. Greater access to HPV vaccination is needed in the region to reduce future HPV-related cancer risk.

## MOAB0301

### Hepatitis C care cascade for people living with HIV in the country of Georgia

N Chkhartishvili ^1^; A Abutidze^1^; N Bolokadze^1^; O Chokoshvili^1^; N Dvali^1^; L Sharvadze^1,2^ and T Tsertsvadze^1,2^

^1^Infectious Diseases, AIDS & Clinical Immunology Research Center, Tbilisi, Georgia. ^2^Ivane Javakhishvili Tbilisi State University, Tbilisi, Georgia

Presenting author email: nikoloch@yahoo.com

**Background**: Georgia made significant progress in addressing high burden of hepatitis C virus (HCV) infection among people living with human immunodeficiency virus (HIV). During 2011–2015 with the support of the Global Fund, HIV-positive persons in Georgia had access to free HCV treatment with pegylated interferon and ribavirin (PEG/RBV). In April 2015 in partnership with the U.S. CDC and Gilead Sciences, the country launched a national hepatitis C elimination programme, which provides free treatment with modern direct-acting antivirals (DAAs).

**Methods**: The following steps of HCV care cascade were quantified: (1) HIV/HCV co-infected, (2) Diagnosed for both HIV and HCV, (3) treated for HCV infection and (4) achieved sustained virologic response (SVR). The number of HIV/HCV co-infected persons was estimated using modelling and observed HCV prevalence. Data on diagnosed persons were extracted from the national AIDS health information system as of 1 September 2016.

**Results**: Among estimated 3300 persons living with HIV/HCV co-infection in Georgia, 2201 (67%) were not aware of their HIV status and 1099 (33%) were diagnosed with both HIV and HCV. Of those 1099 diagnosed persons, 697 (63%) were treated for hepatitis C with either PEG/RBV- or DAA-based regimen; 480 (69%) of those treated achieved SVR. Rates of SVR were 44% with PEG/RBV and 89% with DAA. Overall, because of gap in diagnosis stage, only 15% of estimated number of HIV/HCV co-infected persons were cured.

**Abstract MOAB0301–Figure 1.**
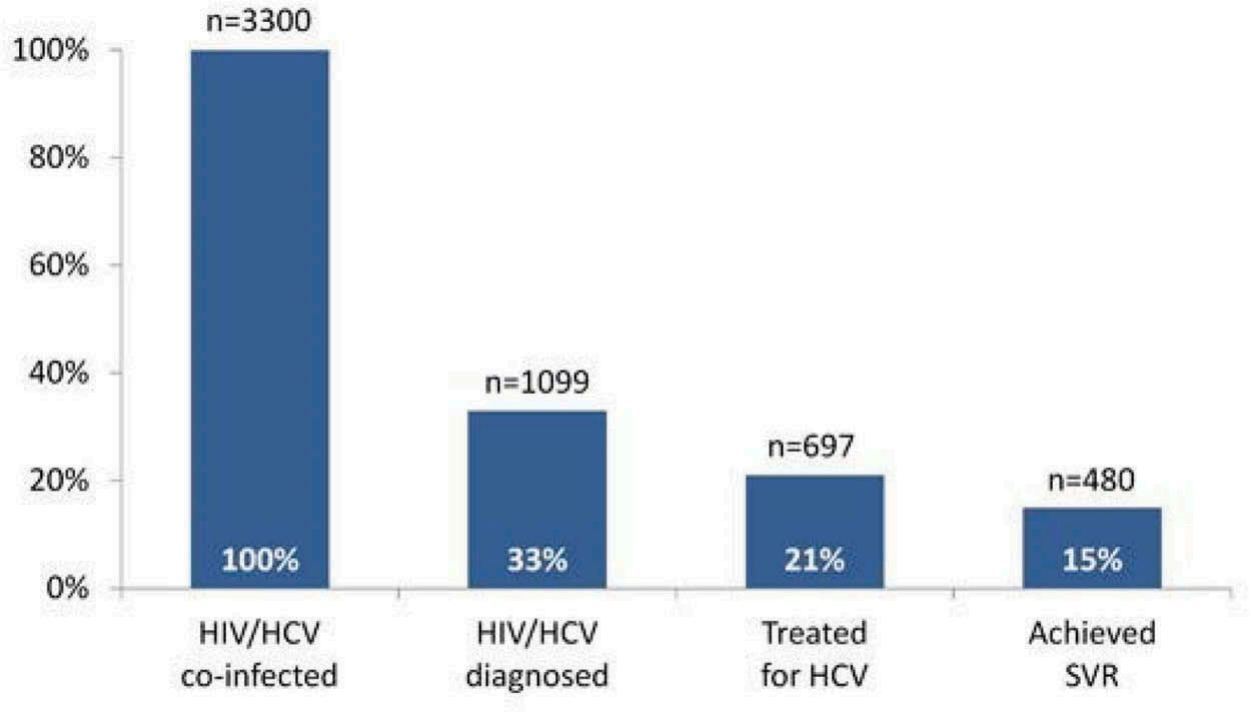
Hepatitis C care cascade.

**Conclusions**: The major gap in the HCV care cascade is at the stage of diagnosis resulting from deficiencies in HIV diagnosis. Reducing the number of people living with undiagnosed HIV/HCV co-infection will be critical for achieving population-level impact of free HCV treatment programme.

## MOAB0302

### Trends in cause-specific mortality in HIV–hepatitis C virus (HCV)-co-infected patients in Canada (2003–2016): early impact of HCV therapy

N Kronfli ^1^; SR Bhatnagar^2^; M Hull^3^; E Moodie^2^; C Cooper^4^; N Pick^3^; S Walmsley^5^; M-L Vachon^6^; V Martel-Leferriere^7^; J Gill^8^; M Klein^1^; on behalf of the Canadian Co-Infection Cohort Investigators

^1^McGill University Health Centre, Montreal, Canada. ^2^McGill University, Epidemiology, Biostatistics & Occupational Health, Montreal, Canada. ^3^University of British Columbia, Medicine, Vancouver, Canada. ^4^Ottawa Hospital Research Institute, Ottawa, Canada. ^5^Toronto General Research Institute, Toronto, Canada. ^6^Universite de Laval, Laval, Canada. ^7^Universite de Montreal, Montreal, Canada. ^8^University of Calgary, Calgary, Canada

Presenting author email: nadine.kronfli@medportal.ca

**Background**: Hepatitis C treatment and an aging population may contribute to shifts in mortality, allowing other causes of death to emerge. We aimed to examine cause-specific mortality among HIV–HCV-co-infected patients, evaluating changes in mortality trends over time.

**Methods**: The Canadian Co-infection Cohort is a prospective multicentre cohort of 1695 co-infected patients from 19 sites in Canada. All reported deaths, classified using a modified “Coding of Cause of Death in HIV” (CoDe) protocol, were analysed from April 2003 to July 2016. Event rates per 1000 person-years before (2003–2009) and after (2010–2016) the availability of effective treatment stratified by age (20–50; 50–80) were calculated. Comparison of trends between periods was performed using Poisson regression. Multinomial regression was used to estimate the cause-specific hazard ratios (HRs) and 95% confidence intervals (CIs) of time and age on cause of death.

**Results**: Overall, 1477 participants (72% men) contributed 6675 person-years of follow-up. Of the 203 (14%) patients who died (152 with assigned causes), end-stage liver disease (ESLD; 20%), smoking related (17%) and drug overdose (16%) were the most common causes of death. All-cause mortality decreased in both age groups over time (Table 1), while HCV treatment increased 2.8 times. Deaths due to ESLD declined by approximately twofold and were no longer the most common cause of death in the 2010–2016 time period for either age category. In contrast, smoking-related deaths increased with time and, among those aged 50–80, accounted for the greatest proportion of deaths from 2010 to 2016 (cause-specific HR 2.8; 95% CI, 1.5–5.4). Drug overdose accounted for the greatest proportion of deaths among individuals aged 20–50 from 2010 to 2016.

**Conclusions**: Increased HCV treatment uptake has coincided with decreased liver-specific mortality in HIV–HCV-co-infected patients. However, these gains may be thwarted if modifiable risk factors (tobacco and drug use) are not addressed.

**Abstract MOAB0302–Table 1.** **All-** **cause and cause-specific event rates (95% CI) per 1000 person-years by age group and time period.**


(*Continued*)

**Abstract MOAB0302–Table 1.** (*Continued*)

## MOAB0303

### Efficacy and safety of glecaprevir/pibrentasvir in patients co-infected with hepatitis C virus and human immunodeficiency virus-1: the EXPEDITION-2 study

J Rockstroh^1^; K Lacombe ^2^; R Viani^3^; C Orkin^4^; D Wyles^5^; A Luetkemeyer^6^; R Soto-Malave^7^; R Flisiak^8^; S Bhagani^9^; K Sherman^10^; P Ruane^11^; J Sasadeusz^12^; J Slim^13^; Z Zhang^3^; T Ng^3^; R Trinh^3^ and M Sulkowski^14^

^1^Universitatsklinikum Bonn, Bonn, Germany. ^2^Université Pierre et Marie Curie, Paris, France. ^3^Abb, North Chicago, USA. ^4^The Royal London Hospital, London, United Kingdom. ^5^Denver Health Medical Center, Denver, USA. ^6^Zuckerber San Francisco General, San Francisco, USA. ^7^Inn, Bayamon, Puerto Rico. ^8^Klinika Chorób Zakaźnych i Hepatologii UM w Białymstoku, Białystok, Poland. ^9^Royal Free London Foundation Trust, London, UK. ^10^University of Cincinnati, Cincinnati, USA. ^11^Ruane Medical & Liver Health Institute, Los Angeles, USA. ^12^Royal Melbourne Hospital, Parkville, Australia. ^13^St. Michael’s Medical Center, Newark, USA. ^14^Johns Hopkins University School of Medicine, Baltimore, USA

Presenting author email: karine.lacombe@sat.ap

**Background**: Pangenotypic, once-daily glecaprevir (identified by AbbVie and Enanta)/pibrentasvir (G/P) has demonstrated high rates of sustained virologic response at 12-week post-treatment (SVR12) in patients with hepatitis C virus (HCV) genotype (GT) 1–6 infection. This phase 3 study evaluated the efficacy and safety of G/P in patients with chronic HCV GT1–6 infection and HIV-1 co-infection, including patients with compensated cirrhosis.

**Methods**: EXPEDITION-2 (NCT02738138) is a phase 3, multicentre open-label study evaluating G/P (300mg/120mg) treatment in HCV/HIV-1-co-infected adults without or with compensated cirrhosis for 8 or 12 weeks, respectively. Patients were either HCV treatment naive or experienced with interferon (IFN), pegylated IFN ± ribavirin or sofosbuvir + ribavirin ± pegylated IFN. GT3 treatment-experienced patients were excluded. The primary endpoint was the proportion of patients with sustained virologic response (HCV RNA < lower limit of quantification) 12-week post-treatment (SVR12).

**Results**: In total, 153 patients were enrolled, including 16 (10%) with cirrhosis. Baseline demographics are shown in Table 1. In patients with available data, rates of response at end of treatment and post-treatment week 4 were 98.7% (151/153) and 98.6% (144/146), respectively. To date, there is one (1/153; 0.65%) virologic failure: a breakthrough in a patient with GT3a infection and cirrhosis. The most common adverse events (AEs) were fatigue (16/153; 10%) and nausea (13/153; 8%). Three patients (2%) had serious AEs, and one serious AE of stroke led to treatment discontinuation on day 23 in one patient with cirrhosis; all were unrelated to G/P. All patients maintained HIV-1 suppression (<200 copies/ml) during treatment.

**Abstract MOAB0303–Table 1.** Baseline demographics and characteristics.

**Conclusions**: G/P for eight weeks in non-cirrhotic and 12 weeks in cirrhotic patients is a well-tolerated and highly efficacious pangenotypic treatment for HCV/HIV-1 co-infection, regardless of baseline HCV RNA or treatment experience. Full SVR12 rates and prevalence of baseline NS3 and NS5A polymorphisms will be presented.

## MOAB0304

### Metabolic syndrome and obesity are the cornerstones of liver fibrosis in HIV-monoinfected patients: results of the METAFIB study

M Lemoine ^1^; K Lacombe^2^; J-P Bastard^3^; M Sebire^4^; L Fonquernie^4^; N Valin^5^; S Fellahi^6^; J Capeau^7^; P-M Girard^8^; J-L Meynard^2^

^1^Imperial College London, Hepatology, London, United Kingdom. ^2^Pierre & Marie-Curie University-INSERM-APHP, Infectious Diseases, Paris, France. ^3^Pierre & Marie-Curie University-INSERM-APHP, Biochemistry and Hormonology, Paris, France. ^4^Pierre & Marie-Curie University-APHP, Infectious Diseases, Paris, France. ^5^Pierre & Marie-Curie University-APHP, Paris, France. ^6^Pierre & Marie-Curie University-APHP, Paris, France. ^7^Pierre & Marie-Curie University-INSERM-APHP, Biochemistry and Hormonology, Paris, France. ^8^Pierre et Marie-Curie University-INSERM-APHP, Infectious Diseases, Paris, France

Presenting author email: m.lemoine@imperial.ac.uk

**Background**: Metabolic syndrome (MetS) has become a common finding in HIV-infected patients. However, the severity, risk factors and pathogenesis of liver fibrosis in this population have been poorly documented. From a matched cohort of HIV-monoinfected patients with and without MetS, this study aimed (1) to assess the impact of MetS on the prevalence and severity of liver fibrosis and (2) to analyse the association between liver fibrosis and markers of adipose tissue, insulin resistance and macrophage activation.

**Methods**: Patients with immune-controlled HIV-1 infection under antiviral therapy (ART) were enrolled in the following exposed-unexposed study. The exposure was defined by the presence of MetS according to international criteria after exclusion of all other causes of chronic liver disease. Fibrosis was assessed using transient elastography (Fibroscan). Adipokines, HOMA index and soluble CD163 and CD14 were measured as markers of fat mass, insulin resistance and macrophage/monocyte activation, respectively.

**Results**: A total of 468 HIV-monoinfected individuals were enrolled (male (89%), mean age 53 (9) years, mean body mass index 24.6 (5.3) kg/m^2^): 246 with MetS and 222 without MetS. Patients with MetS were older and 49% of them had insulin resistance, that is, HOMA-IR ≥2.5 (compared to 8.5% in patients without MetS). The mean value (SD) of liver stiffness measurement (LSM) was 5.6 (2.2) kPa with a minimum and maximum value of 2.4 and 17.1 kPa. Mean LSM was higher in patients with MetS compared to those without MetS (6.3 (2.6) versus 4.9 (1.5) kPa, *p* < 0.0001). In multivariable analysis, obesity (odds ratio: 3.9 (95% confidence interval 2.1–7.1)) and insulin resistance (1.1 (1.06–1.2)) were independent factors of significant fibrosis (≥F2) and remained associated after adjustment on MetS. Serum levels of adipokines and sCD163 were significantly associated with the degree of liver fibrosis. When adjusted on MetS, leptin and sCD163 remained strongly associated to fibrosis. HIV parameters and ART regimen were not associated to fibrosis.

**Conclusions**: In HIV-monoinfected patients, MetS is an important risk factor for liver fibrosis. Obesity and insulin resistance are key factors in the development of liver fibrosis independently of HIV infection parameters. Adipose tissue and macrophage activation certainly play an important role in the development of fibrosis in HIV-monoinfected patients, but the exact mechanisms need to be elucidated.

## MOAB0305

### Predictor factors associated with liver fibrosis and steatosis by transient elastography in HIV-monoinfected patients under long-term combined antiretroviral therapy: the PROSPEC-HIV study

H Perazzo Pedroso Barbosa; S Cardoso; C Yanavich; JC Soares; J Fittipaldi; M Morata; C Cardoso; P Simplicio; C De Almeida; V Veloso and B Grinsztejn

Fundação Oswaldo Cruz, LAPCLIN-AIDS, Rio de Janeiro, Brazil

Presenting author email: perazzohugo@gmail.com

**Background**: Liver disease remains one of the main causes of non-AIDS mortality in HIV-infected individuals. Transient elastography (TE) is an accurate imaging method to estimate liver fibrosis and steatosis. We aimed to evaluate the risk factors associated with liver fibrosis and steatosis in HIV-monoinfected patients under long-term combined-antiretroviral therapy (c-ART).

**Methods**: This cross-sectional study prospectively included HIV-infected adult patients under c-ART (PROSPEC-HIV; NCT02542020). Liver stiffness measurement (LSM) and controlled attenuation parameter (CAP) by TE were used to estimate liver fibrosis and steatosis, respectively. Exclusion criteria were hepatitis co-infection and c-ART naive. Patients with an unreliable M probe LSM or CAP were excluded from the liver fibrosis and steatosis analyses, respectively. Clinical evaluation, fasting blood tests and TE were performed at the same day. TE exams were performed by a single experimented operator blinded to clinical and laboratory data. Metabolic factors were defined according to the International Diabetes Federation criteria. Alcohol consumption was quantified using the AUDIT score. Presence of liver fibrosis and steatosis was considered when LSM ≥ 8.0 kPa and CAP ≥ 250 dB/m, respectively. Age- and gender-adjusted multivariate logistic regression was performed.

**Results**: A total of 348 HIV-monoinfected patients (61% female, median (interquartile range [IQR]) age = 44 (34–52) years, body mass index = 25.4 (23.0–29.3) kg/m²) were included. Median (IQR) time under c-ART and under the current c-ART regimen were 7.3 (4.1–12.8) and 4.3 (1.9–7.5) years, respectively. LSM and CAP were unreliable in 6% and 12%. Liver fibrosis and steatosis prevalence were 9% (*n* = 30/326) and 33% (*i* = 102/305). In age- and gender-adjusted multivariate analysis, factors associated (OR (95% confidence interval)) with liver fibrosis were: age >45 years (2.91 (1.19–7.15); *p* = 0.020); CD4 count <200 cells (5.00 (1.38–18.21); *p* = 0.014) and type-2 diabetes (3.04 (0.97–9.55); *p* = 0.056). Male gender (5.69 (2.68–12.04); *p* < 0.001); dyslipidaemia (2.86 (1.46–5.60); *p* = 0.002); type 2 diabetes (6.00 (2.08–17.28); *p* = 0.001) and central obesity (10.24 (4.11–25.50); *p* < 0.001) were independently associated with liver steatosis.

**Conclusions**: Non-communicable diseases (NCD) can play a major role in the development of liver fibrosis and steatosis. NCD prevention and care services need to be integrated to HIV care to decrease the burden of hepatic events in HIV-infected individuals.

## MOAB0401

### Gaps and opportunities in policy and practice in 20 countries with the highest burden of HIV-associated TB

A Baddeley ^1^; M Doherty^2^; A Kanchar^1^; Y Hamada^1^ and H Getahun^1^

^1^World Health Organization, Global TB Programme, Geneva, Switzerland. ^2^Department of HIV/AIDS & Global Hepatitis Programme, World Health Organization, Geneva, Switzerland

Presenting author email: baddeleya@who.int

**Background**: Despite impressive scale-up of collaborative TB/HIV activities since 2004, TB is still the major cause of morbidity and mortality among people living with HIV (PLHIV). In June 2016, Member States adopted the UN Political Declaration on HIV and AIDS, including a target to reduce TB deaths among PLHIV by 75% by 2020. In order to achieve this, gaps in policy, implementation and recording and reporting need to be identified and urgently addressed.

**Methods**: A total of 20 countries were selected with the highest estimated burden of HIV-positive incident TB in 2015. Data on collaborative TB/HIV activities were extracted from the Global TB Programme Database and the UNAIDS online GARPR tool, and trends and progress analysed. Reviews were further conducted of the latest available policy documents, programme reviews, epidemiological assessments, Global Fund Concept Notes and GARPR reports to identify gaps and opportunities in policy and implementation.

**Results**: Cascade analysis revealed a 57% gap in notification of TB patients living with HIV, compared with estimated cases, and a 19% gap of ART started among notified HIV-positive TB patients (Figure 1). Half of countries did not report isoniazid preventive therapy (IPT). Case fatality among TB patients living with HIV was at least three times higher than HIV-negative TB patients in seven reporting countries. Document review revealed the following gaps and barriers: misalignment of policies on ART and IPT; poorly implemented TB screening and IPT; centralized or underuse of Xpert MTB/RIF; centralized ART provision; stock-outs in IPT, HIV testing kits and ART; and separate planning, supervision, health management information systems and procurement and supply.

**Conclusions**: Considerable gaps and opportunities were identified in this analysis. Countries need to seek ways to resolve barriers, be they policy, implementation or health system-related to ensure access to evidence-informed HIV-associated TB care and to end HIV-related deaths from this preventable disease.

**Abstract MOAB0401–Figure 1.**
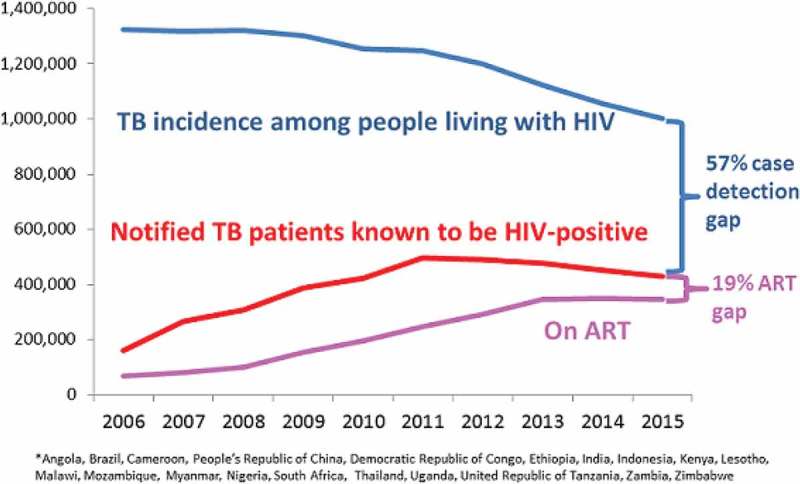
TB/HIV notification and ART initiation compared with estimated HIV-positive incident cases, 20 high-burden TB/HIV countries*, 2015

## MOAB0402

### Genomic epidemiology of extensively drug-resistant tuberculosis in KwaZulu-Natal, South Africa: demographic expansion and genetic determinants of epidemiologic success in a high HIV prevalence setting

TS Brown ^1,2^; S-O Kolokotronis^3^; SC Auld^4,5^; S Omar^6^; A Narechania^7^; NS Shah^8^; KN Nelson^5^; N Ismail^6^; BN Kreiswirth^9^; JCM Brust^10^; P Moodley^11^; NR Gandhi^5^; B Mathema^2^

^1^Columbia University Medical Center, New York, USA. ^2^Columbia University Mailman School of Public Health, New York, USA. ^3^Department of Public Health, SUNY Downstate Medical Center, New York, USA. ^4^Emory University School of Medicine, Atlanta, USA. ^5^Emory University Rollins School of Public Health, Atlanta, USA. ^6^National Institute for Communicable Diseases, Johannesburg, South Africa. ^7^American Museum of Natural History, New York, USA. ^8^Centers for Disease Control and Prevention, Atlanta, USA. ^9^Public Health Research Institute, New Jersey Medical School - Rutgers, Newark, USA. ^10^Albert Einstein College of Medicine, Bronx, USA. ^11^University of KwaZulu-Natal, Durban, South Africa

Presenting author email: tsb2126@columbia.edu

**Background**: Extensively drug-resistant tuberculosis (XDR-TB) has emerged over the last decade as a significant public health threat worldwide, particularly among people with HIV. South Africa first reported XDR-TB in 2005 and now has among the highest burden of XDR-TB worldwide, with >1000 cases diagnosed in 2015. The bacterial evolutionary determinants behind the rise of XDR-TB in South Africa are not well understood.

**Methods**: We enrolled persons with newly diagnosed, culture-confirmed XDR-TB from 2011 to 2014 in KwaZulu-Natal province and performed whole-genome sequencing of their *Mycobacterium tuberculosis* (*Mtb*) isolates. Lineage 4 isolates were selected for phylogenetic reconstruction, dating of drug-resistance mutations and estimates of prior demographic history using Bayesian Markov chain Monte Carlo Methods (BEAST v1.8.3).

**Results**: Among 160 participants with XDR *Mtb* isolates included in this analysis, 127 (79%) were HIV co-infected. Half (51%) of XDR-TB cases were attributable to a single predominant clade of highly monomorphic isolates (Restriction Fragment Length Polymorphism type HP). There were no significant differences between the proportion of participants with HIV or with CD4 counts <200 cells/µl in the HP vs. non-HP isolates. Both HP and non-HP *Mtb* populations exhibit evidence of rapid population expansion beginning 25–30 years ago (Figure 1b). The emergence of key drug-resistance mutations occurred near the historical dates of introduction for their corresponding antibiotics (Figure 1a).

**Abstract MOAB0402–Figure 1.**
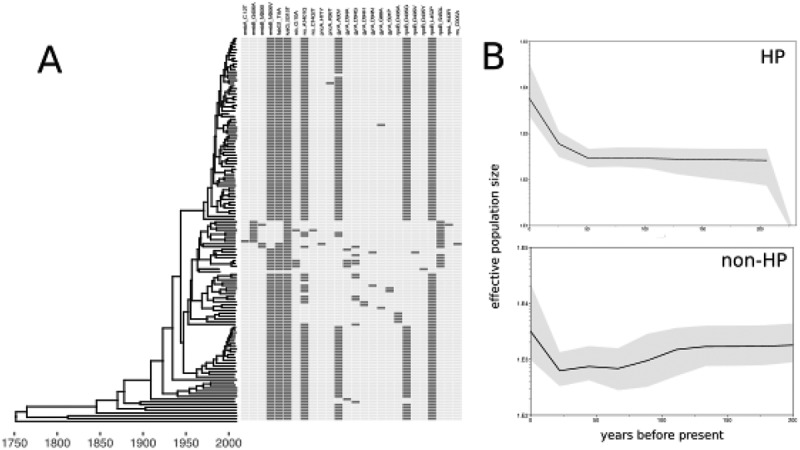
IAS1. (a) Dated phylogenetic tree for HP isolates annotated with known drug-resistance mutations; (b) Bayesian Skyline Reconstruction for HP and non-HP isolates.

**Conclusions**: *Mtb* isolates from the predominant HP clade and from less prevalent XDR-TB strains underwent demographic expansion following the onset of the HIV epidemic in South Africa. Endemic strains in KwaZulu-Natal acquired drug resistance mutations across diverse strain groups and genetic backgrounds, corresponding to the introduction of new antituberculosis medications. The impact of HIV co-infection on pathoadaptive evolution in *Mtb* remains an important area for further investigation.

## MOAB0403

### Incidence of tuberculosis in the first year of antiretroviral treatment in West-African HIV-infected adults

B Tchakounte Youngui ^1^; P Coffie^2^; E Messou^2,3^; A Poda^4^; L Fortes Déguénonvo^5^; D Hawerlander^6^; A Minga^2,7^; E Balestre^8^; F Dabis^8^ and O Marcy^9^

^1^University of Bordeaux, Bordeaux School of Public Health, ISPED, Bordeaux, France. ^2^Programme PACCI, Site ANRS, Abidjan, Cote D’Ivoire. ^3^Centre de Prise en Charge de Recherche et de Formation, CePReF-Aconda-VS, Abidjan, Cote D’Ivoire. ^4^CHU Souro Sanou, Hôpital de Jour, Service des Maladies Infectieuses, Bobo-Dioulasso, Burkina Faso. ^5^CHNU Fann, Service des maladies infectieuses et tropicales Ibrahima Diop Mar, Dakar, Senegal. ^6^Centre Intégré de Recherches Biocliniques d’Abidjan, CIRBA, Abidjan, Cote D’Ivoire. ^7^Centre Médical de Suivi de Donneurs de Sang/CNTS/PRIMO-CI, Abidjan, Cote D’Ivoire. ^8^University of Bordeaux, Centre INSERM U897 - Epidémiologie-Biostatistique, Bordeaux School of Public Health, ISPED, Bordeaux, France. ^9^University of Bordeaux, Centre INSERM U1219, Bordeaux School of Public Health, ISPED, Bordeaux, France

Presenting author email: boris_2022@yahoo.fr

**Background**: Despite evidence that isoniazid preventive therapy (IPT) reduces tuberculosis incidence and mortality, its uptake remains very limited in West Africa. Our objective was to assess tuberculosis incidence during the first year of antiretroviral therapy (ART) and identify associated factors in HIV-infected adults in West Africa to support policy decisions in these countries.

**Methods**: We conducted a retrospective observational cohort analysis using data collected in three HIV outpatient centres from Côte d’Ivoire, Burkina Faso and Senegal participating in the IeDEA West Africa Collaboration. We included HIV-infected adults (≥16 years) initiating ART between 2010 and 2014, without tuberculosis diagnosis at ART initiation and with ≥1 follow-up visit. None of them received IPT. Tuberculosis new diagnoses were documented according to national recommendations. We analysed incidence of tuberculosis and identified associated factors using Poisson regression models.

**Results**: Of 4154 patients who started on ART, 3404 had ≥1 follow-up visit after ART initiation. Of those, 191 (5.6%) had ongoing tuberculosis at ART initiation. Thus, we enrolled 3213 patients in our analysis. Median age was 38.5 (interquartile range (IQR) 32.0–45.4) years, 67.1% were female and median CD4 count at ART initiation was 211 (IQR 95–343) cells/mm^3^. Overall 170 new tuberculosis cases were reported for 2360.5 person-years (PY) at risk. The crude tuberculosis incidence rate during the first year on ART was 7.2 (95% confidence interval (CI) 6.12–8.28) cases per 100 PY. The adjusted tuberculosis incidence rate was 1.42 (95% CI 0.55–3.20) per 100 PY in women, aged 16–30 years, without prior tuberculosis history, with CD4 ≥500 cells/mm^3^ and haemoglobin ≥11 g/dl, followed in Burkina-Faso. A higher tuberculosis risk was significantly associated with male gender (relative risk (RR) 1.87; 95% CI 1.28–2.74), previous history of TB (RR 4.22; 95% CI 2.70–6.42), haemoglobin <9 g/dl (RR 2.26; 95% CI 1.61–4.31) and follow-up in Côte d’Ivoire (RR 4.32; 95% CI 2.90–6.50).

**Conclusions**: Tuberculosis incidence remains high during the first year on ART in the West African context in the absence of IPT. It is crucial to reinforce implementation of IPT in all HIV-infected adults starting ART, especially in this part of the world.

## MOAB0404

### Time to treatment initiation for drug-resistant tuberculosis is delayed in a South African prospective cohort

BJ Sullivan ^1,2^; J Prvu Bettger^1,2,3^; S Silva^1^; J Humphreys^1^; CK Cunningham^2,4^ and JE Farley^5^

^1^Duke University, School of Nursing, Durham, USA. ^2^Duke Global Health Institute, Durham, USA. ^3^Duke University School of Medicine, Orthopaedic Surgery, Durham, USA. ^4^Department of Pediatrics, School of Medicine, Duke University, Durham, USA. ^5^School of Nursing, Johns Hopkins University, Baltimore, USA

Presenting author email: brittney.sullivan@duke.edu

**Background**: Drug-resistant tuberculosis (DR-TB) is a growing threat to TB management and elimination globally. Early initiation of DR-TB treatment is critical to successful treatment outcomes (cure) and to prevent transmission. The South African National TB Programme (NTP) recommends initiating treatment within five days of diagnosis. This study examined time to treatment initiation and its relationship with patient age among a prospective cohort of individuals living with DR-TB in South Africa.

**Methods**: Patients included subjects aged 13 years or older, enrolled in a DR-TB cluster-randomized trial in two South African provinces. Outcomes were treatment initiation within five days of DR-TB diagnosis and days from diagnosis to treatment initiation. Hierarchical mixed-effects models were employed to examine the association between age and these outcomes, adjusted for patient (sex, TB history and HIV co-infection) and site (rural/urban and province) characteristics and random effects of treatment centre.

**Results**: Of 521 patients, there were 55% male, 75% with HIV co-infection and 53% with prior history of TB. Only 82 patients (16%) received DR-TB treatment within five days of diagnosis. The median time to treatment was 11 days (range = 0–180). Age was not associated with treatment initiation within five days (*F* = 0.07, *df* = 1495, *p* = 0.794) or days to treatment initiation (*F* = 1.42, *df* = 1489, *p* = 0.233). Individuals co-infected with HIV tended to have a greater likelihood of receiving treatment within five days relative to those without co-infection (17% vs. 12%, *p* = 0.104; odds ratio = 1.749, 95% confidence interval = 0.891–3.433).

**Conclusions**: Delays in DR-TB treatment increase harm to the patient and risk of disease spread. Only one in six patients with DR-TB received treatment within five days of being diagnosed as recommended by the South African NTP guidelines. Further research is needed to examine what modifiable factors decrease treatment delay and how to most effectively implement treatment guidelines.

## MOAB0405

### High uptake of antiretroviral therapy among HIV-positive TB patients receiving co-located services in Swaziland

I Pathmanathan ^1^; M Pasipamire^2^; S Pals^1^; EK Dokubo^1^; P Preko^3^; T Ao^3^; S Mazibuko^2^; J Ongole^4^ and S Haumba^4^

^1^U.S. Centers for Disease Control and Prevention, Division of Global HIV and TB, Atlanta, USA. ^2^Ministry of Health, Swaziland, Swaziland National AIDS Programme, Mbabane, Swaziland. ^3^U.S. Centers for Disease Control and Prevention, Division of Global HIV and TB, Mbabane, Swaziland. ^4^University Research Co, LLC, Mbabane, Swaziland

Presenting author email: ydi6@cdc.gov

**Background**: Swaziland has the highest adult HIV prevalence, the third highest tuberculosis (TB) incidence and the highest rate of TB/HIV co-infection globally. In recent years, the Ministry of Health and partners have increased integration and co-location of TB and HIV services, but the timing of antiretroviral therapy (ART) initiation relative to TB treatment - a marker of programme quality and predictor of outcomes - is unknown.

**Methods**: We conducted a retrospective review of programmatic data from 11 purposefully sampled facilities to evaluate provision of timely ART for adult (≥15 years) HIV-positive TB patients seen and retained in care between July and September 2014. Timely ART was defined as initiated within two weeks of TB treatment initiation for patients with CD4 <50/µl or missing and within eight weeks for others. Descriptive statistics were estimated and logistic regression was used to assess factors independently associated with timely ART.

**Results**: Of 466 HIV-positive TB patients, 51.5% were male, median age was 35 (interquartile range (IQR): 29–42) and median CD4 was 137/µl (IQR: 58–268). A total of 189 (40.6%) were on ART prior to, and five (1.8%) did not receive ART within, six months of TB treatment initiation. Median time to ART initiation after TB treatment initiation was 15 days (IQR: 14–28). Almost 90% started ART within eight weeks. Twenty-five of 55 patients (45.5%) with CD4 <50/µl started within two weeks. Of 44 (16.1%) patients without a documented CD4, 47.7% began ART within two weeks and 93.2% within eight. Patients with CD4 50–200/µl or ≥200/µl had significantly higher odds of receiving timely ART than patients with CD4 <50/µl, with adjusted odds ratios of 11.3 (95% confidence interval [CI]: 5.0–25.8) and 10.38 (95% CI: 4.89–22.03), respectively; 71.2% achieved TB cure or treatment completion at six months, but this was not associated with timely ART.

**Conclusions**: This study demonstrates the relative success of integrated and co-located TB/HIV services in Swaziland and shows that very high levels of timely ART uptake for HIV-positive TB patients can be achieved in integrated but resource-limited TB/HIV settings. However, gaps remain in getting patients with CD4 <50/µl to receive ART within the recommended two weeks post-TB treatment initiation.

## MOAB0406

### Feasibility of using Determine-TB LAM test in HIV-infected adults in programmatic conditions

SC Mathabire ^1,2^; L Cossa^3^; J Mpunga^4^; I Manhiça^5^; I Amoros Quiles^6^; L Molfino^3^; E Szumilin^7^; O Telnov^8^ and H Huerga^1^

^1^Epicentre, Clinical Research Department, Paris, France. ^2^Medecins Sans Frontieres, Chiradzulu, Malawi. ^3^Medecins Sans Frontieres, Maputo, Mozambique. ^4^National TB Control Program (MoH), Lilongwe, Malawi. ^5^National TB Program, Maputo, Mozambique. ^6^Medecins Sans Frontieres, Lilongwe, Mozambique. ^7^Medecins Sans Frontieres, Paris, France. ^8^Medecins Sans Frontieres, Geneva, Switzerland

Presenting author email: chenai.mathabire@epicentre.msf.org

**Background**: We assessed the feasibility and described the operational aspects of using the Determine-TB LAM (LAM) test for diagnosis of tuberculosis (TB) in adult HIV-infected patients.

**Methods**: This multicentric study was conducted in Malawi and Mozambique from 2014 to 2016. LAM was used as a rule in screening tool for hospitalized adult HIV-infected patients (Malawi) and for ambulatory patients with CD4 <100/µl (Mozambique) and as an additional diagnostic tool for adult HIV-infected TB suspects with CD4 <200/µl (Malawi and Mozambique). Standard questionnaires were used to assess user acceptability of LAM; electronic databases were used to calculate reader agreement between LAM users; and health centre registers were used to calculate workload. Supervision notes, minutes of meetings, training reports and personal observations were used to assess training required, patient flow changes after LAM introduction and strengths and challenges of using the LAM test.

**Results**: Training of LAM users was performed in approximately 1.5 hours in Malawi and 4 hours in Mozambique. All users found the test easy to perform. Reader agreement for test interpretation was excellent: 98.9%, kappa = 0.97, and 98.3%, kappa = 0.94 for Malawi and Mozambique, respectively. Time to results when LAM was performed in the consultation room was two to seven times lower than when performed in the laboratory. LAM-positive patients were started on TB treatment on same day. Introduction of LAM did not require additional space or staff. Strength of LAM was that most patients had a test done with results available: 98.7% and 99.6% in Malawi and Mozambique respectively. In comparison, 69.5% and 67.2% had sputum results and 31.7% and 46.0% had chest x ray results respectively in Malawi and Mozambique. A challenge in Mozambique was the need for CD4 prior to the LAM test to identify LAM-eligible patients.

**Conclusions**: Using the LAM test to diagnose TB among hospitalized or severely immunosuppressed ambulatory HIV patients was feasible, was well accepted and required minimal training. The LAM was a useful additional test for TB in this group because of the ease of providing the urine sample and the rapidity of the results which allowed immediate TB treatment for LAM-positive patients.

## MOAD0101

### Readiness for antiretroviral therapy: implications for linking HIV-infected individuals to care and treatment

B Maughan-Brown ^1^; P Smith^2^; C Kuo^3^; A Harrison^3^; M Lurie^4^; L-G Bekker^2^ and O Galárraga^5^

^1^Southern Africa Labour and Development Research Unit (SALDRU), University of Cape Town, Cape Town, South Africa. ^2^The Desmond Tutu HIV Centre, University of Cape Town, Cape Town, South Africa. ^3^Department of Behavioral and Social Sciences, Brown University School of Public Health, Providence, USA. ^4^Department of Epidemiology, Brown University School of Public Health, Providence, USA. ^5^Department of Health Services, Policy & Practice (HSPP), Brown University School of Public Health, Providence, USA

Presenting author email: brendan.maughanbrown@gmail.com

**Background**: Antiretroviral therapy (ART) readiness is a key predictor of ART initiation. However, there is a paucity of data on ART readiness among individuals at the time of HIV diagnosis and ART eligibility assessment. Under a test-and-treat approach, understanding factors associated with ART readiness can inform strategies to support early engagement in care and thereby maximize the benefits of ART. This study examined demographic and psychosocial factors associated with ART readiness and potential barriers to linkage to care among individuals referred for treatment from a mobile health clinic.

**Methods**: Between April 2015 and May 2016, 87 individuals (18 years and older) in a resource-limited setting in Cape Town, South Africa, completed a face-to-face survey immediately after referral for ART. ART readiness was assessed using key components of this concept identified in the literature: (1) an awareness that treatment will be beneficial; (2) motivation to initiate treatment; and (3) the intention to start treatment soon. Multiple logistic regression analysis, controlling for age, gender and education, identified factors associated with ART readiness.

**Results**: Most participants were very ready (84%) and motivated (85%) to start ART, but 28% reported some uncertainty regarding ART initiation. Treatment readiness was lower among those surprised by their diagnosis (adjusted odds ratio (aOR): 0.26, *p* < 0.05) and among healthier individuals (aOR: 0.44, *p* < 0.01). In contrast, higher readiness was associated with better ART knowledge (aOR: 4.31, *p* < 0.05) and knowing someone who had experienced positive health effects from ART (aOR: 2.65, *p* < 0.05). The three most common self-reported barriers to linking to care were (1) not wanting to be seen at the clinic (31%); (2) no money for transport (29%); and (3) not being able to get time off work (20%).

**Conclusions**: Results indicate that post-test counselling will need to be designed to deal with surprise at HIV diagnosis and that health messaging needs to be carefully crafted for HIV-positive but healthy individuals to improve ART readiness and to increase likelihood of further linkage to treatment and care. Further research is needed on effective post-test counselling approaches (e.g. motivational-interviewing) and effective framing of health messaging to increase awareness of the positive benefits of early ART initiation and corresponding motivation to engage in treatment.

## MOAD0102

### Factors associated with loss to follow-up in a primary healthcare HIV clinic practising test and treat

J Kiwanuka ^1,2^; N Kiwanuka^3^; F Matovu Kiweewa^3^; J Gonzalez Perez^4^; J Kitonsa^2,5^ and J Waila Mukulu^6^

^1^AIDS Healthcare Foundation, M&E, Kampala, Uganda. ^2^Makerere University School of Public Health, Kampala, Uganda. ^3^Makerere University School of Public Health, Epidemiology and Biostatics, Kampala, Uganda. ^4^AIDS Healthcare Foundation, Kigali, Rwanda. ^5^Medical Research Council-Uganda Virus Research Institute, Masaka, Uganda. ^6^Makere University School of Public Health, Kampala, Uganda

Presenting author email: jkiwanuka74@gmail.com

**Background**: The World Health Organization (WHO) currently recommends all people diagnosed HIV positive to start antiretroviral therapy instantly (T&T) regardless of CD4 cell count and WHO stage. Currently, however, a myriad of HIV treatment programmes are plagued with the challenge of patients’ loss to follow-up. We set out to study factors associated with loss to follow-up (LTFU) in a primary healthcare facility practising T&T.

**Methods**: We retrospectively drew and analysed a sample of patients from routine patients’ data for HIV clients enrolled into HIV care from January 2012 to December 2014 at Masaka Regional Referral Hospital - Uganda Cares clinic. We defined loss to follow-up as failure of the client to show up at the Masaka clinic for at least 91 days from the date of their last appointment taking 31 December 2014 as the reference. We determined cumulative incidence of loss to follow-up at differing time intervals and used multivariable cox proportional hazards regression model to determine factors associated with time to LTFU.

**Results**: We included 600 patients in the sample, 64.7% were females and the median (interquartile range (IQR)) age at enrolment of 30.4 (23.8–37.1). The median (IQR) CD4 cell count at start of ART was 373 (204–570), and 15.2% were in WHO stage 3 or 4. By 31 December 2014, 55 cases of LTFU were observed, and the cumulative incidence of LTFU was 8.48% (95% confidence interval (CI) = 6.26–11.12) at 12 months into HIV care. In multivariable analysis, T&T (aHR = 2.49, 95% CI = 1.07–5.78), WHO stage 3 and 4 (aHR = 3.78, 95% CI = 1.70–8.41) and TB suspect (aHR = 3.42, 95% CI = 1.19–9.81) were associated with an elevated risk of LTFU, whereas access to mobile phone (aHR = 0.56, 95% CI = 0.36–0.88) and duration on ART one to three months (aHR = 0.21, 95% CI = 0.08–0.59), three to six months (aHR = 0.03, 95% CI = 0.01–0.11) and ≥6 months (aHR = 0.003, 95% CI = 0.001–0.01) were independently associated with reduced risk of LTFU.

**Conclusions**: This study identified testing and initiating on ART instantly being associated with elevated risk of LTFU and as well TB suspicion and advanced disease at enrolment. In a bid to achieve the 90-90-90 campaign therefore, steep ART initiation should be backed by intensive pre-initiation and adherence counselling for better long-term retention of patients.

## MOAD0103

### Pilot study of a multipronged intervention using social norms and priming to improve adherence to antiretroviral therapy and retention in care among adults living with HIV in Tanzania

S McCoy ^1^; C Fahey^1^; A Rao^2^; N Kapologwe^3^; P Njau^4^ and S Bautista-Arredondo^5^

^1^University of California, Berkeley, Berkeley, USA. ^2^CVS Health, Providence, USA. ^3^Ministry of Health, Community Development, Gender, Elderly, and Children, Shinyanga, United Republic of Tanzania. ^4^Ministry of Health, Community Development, Gender, Elderly, and Children, Dar es Salaam, United Republic of Tanzania. ^5^National Institute of Public Health, Cuernavaca, Mexico

Presenting author email: smccoy@berkeley.edu

**Background**: Interventions incorporating insights from behavioural economics and psychology have been successfully used in the private sector and have the potential to enhance HIV “treatment as prevention” (TasP). This approach recognizes that decisions are influenced by emotions, contexts and decision-making shortcuts outside of conscious awareness. To test this hypothesis, we evaluated an intervention to improve antiretroviral therapy (ART) adherence and retention in care based on concepts of social norms and priming.

**Methods**: We used patient-centred design to develop a combination intervention using social norms and priming, which is when a stimulus subconsciously or indirectly influences another behaviour. The intervention included visual feedback about clinic-level retention in care (social norms), a self-relevant prime and useful take-home items with the priming image (calendar, pill box). The intervention was implemented at two HIV primary clinics in Shinyanga, Tanzania, in two-week intervals for six months. We conducted a quasi-experimental pilot study (Clinicaltrials.gov: NCT02938533) by reviewing the medical records of a random sample of 438 adult patients living with HIV infection (PLHIV, 320 exposed and 118 unexposed) to compare retention and the proportion of patients achieving a medication possession ratio (MPR) ≥95% after six months. Intervention acceptability was determined through an in-person survey with a convenience sample of 405 PLHIV at baseline (*n* = 189) and end line (*n* = 216).

**Results**: In adjusted analyses, PLHIV exposed to the intervention was significantly more likely to be in care after six months (87% vs. 79%, adjusted odds ratio (OR_a_) = 1.73, 95% confidence interval (CI): 1.08–2.78, *p* < 0.05) and were more likely to achieve MPR ≥95% (70% vs. 59%, OR = 1.51, 95% CI: 0.96–2.37, *p* = 0.07). The intervention was associated with increases in staff support of treatment goals (100% vs. 95%, *p* = 0.01) and life goals (66% vs. 50%, *p* < 0.01), the perceived likelihood of other patients’ adherence (54% vs. 32%, *p* < 0.01), support from other patients (71% vs. 60%, *p* = 0.03) and being very satisfied with care (53% vs. 35%, *p* < 0.01).

**Conclusions**: This novel intervention has the potential to improve the clinic experience, short-term retention in care and ART adherence. Future studies are needed to expand the generalizability of the approach and evaluate effectiveness on clinical outcomes.

## MOAD0104

### Multi-month refills of antiretroviral drugs for stable patients in Malawi: assessing accuracy in the application of eligibility criteria at the health facility level

M Prust ^1^; C Banda^2^; R Nyirenda^3^; F Chimbwandira^3^; T Kalua^3^; A Jahn^4^; M Eliya^3^; K Callahan^5^; P Ehrenkranz^6^; M Prescott^7^; E McCarthy^7^; E Tagar^5^ and A Gunda^2^

^1^Clinton Health Access Initiative, Applied Analytics Team, Boston, USA. ^2^Cli, Lilongwe, Malawi. ^3^Department of HIV and AIDS, Ministry of Health, Lilongwe, Malawi. ^4^Department of HIV and AIDS, Ministry of Health/I-TECH Department of Global Health, Lilongwe, Malawi. ^5^Cli, HIV, TB and Health Financing Team, New York, USA. ^6^Bill & Melinda Gates Foundation, Seattle, USA. ^7^Cli, Applied Analytics Team, Boston, USA

Presenting author email: mprust@clintonhealthaccess.org

**Background**: The provision of three-month antiretroviral (ARV) refills, or multi-month scripting (MMS), for stable HIV patients on antiretroviral therapy (ART) can increase service efficiency and decrease congestion. Since 2008, Malawi has offered MMS to patients that are 18 years or older, have been on ART at least six months, are on first-line ART, have no adverse drug reactions or opportunistic infections, have a viral load less than 1000 copies/ml and have good adherence according to pill counts. We assessed the extent to which patients are accurately differentiated as eligible or ineligible for MMS and explored potential causes of inaccurate patient differentiation.

**Methods**: Data were collected from 30 purposefully selected ART facilities in 2016. Participation and eligibility for MMS were determined based on 75,364 patient clinical records, which were analysed using Stata version 13. Results were weighted and clustered by facility. The reasons for inaccurate patient differentiation were explored using structured surveys with 136 health workers and 32 qualitative interviews with clinic management. Interviews were audio recorded, transcribed and thematically coded.

**Results**: A majority of patients (86.4%, 95% confidence interval [CI] 84.0–88.6) were eligible and 68.7% of patients (95% CI 62.5–74.6) were receiving MMS. Among patients eligible for MMS, 72.9% (95% CI 66.3–78.6) received MMS. However, 42.3% (95% CI 33.1–52.0) of ineligible patients (amounting to 5.7% of all patients) also received MMS. Results were similar based on sensitivity analyses using different eligibility criteria scenarios, but variation in the application of criteria existed across facilities. Among ineligible patients receiving MMS, 77% had viral load greater than 1000 copies/ml, and 39% had been on ART less than six months. Inaccurate patient differentiation was suggested to result from lack of health worker knowledge of the criteria for MMS, patient requests, health worker attempts to reduce workload and perceived challenges with low stocks of medications.

**Conclusions**: MMS is being widely implemented in Malawi, but patient differentiation in many facilities is not happening according to the agreed upon definition of eligibility. Simplification of guidance, improvements in health worker mentorship, patient counselling and alignment of patient record forms against eligibility criteria would improve patient differentiation in Malawi.

## MOAD0105

### Multi-month prescription of antiretroviral therapy and its feasibility: experiences from the Baylor International Pediatric AIDS Initiative (BIPAI) in six southern African countries

M Kim ^1^; RS Wanless^2^; S Ahmed^3^; J Mhango^3^; D Damba^4^; A Kayabu^5^; M Chodota^6^; S Dlamini^7^; N Chidah^8^; M Mokhali^9^; N Calles^2^; A Amzel^10^; R Golin^10^ and EJ Abrams^11^

^1^Baylor International Pediatric AIDS Initiative, Children’s Clinical Center of Excellence, Lilongwe, Malawi. ^2^Baylor International Pediatric AIDS Initiative, Houston, USA. ^3^Baylor International Pediatric AIDS Initiative, Lilongwe, Malawi. ^4^Baylor International Pediatric AIDS Initiative, Kampala, Uganda. ^5^Baylor International Pediatric AIDS Initiative, Mwanza, United Republic of Tanzania. ^6^Baylor International Pediatric AIDS Initiative, Mbeya, United Republic of Tanzania. ^7^Baylor International Pediatric AIDS Initiative, Mbabane, Swaziland. ^8^Baylor International Pediatric AIDS Initiative, Gabarone, Botswana. ^9^Baylor International Pediatric AIDS Initiative, Maseru, Lesotho. ^10^United States Agency for International Development, Washington, USA. ^11^International Center for AIDS Care and Treatment Programs, Mailman School of Public Health, Columbia University, New York, USA

**Background**: To improve antiretroviral coverage (ART) and help reach the 90-90-90 treatment targets, differentiated approaches to care are necessary, including reduced frequency of clinic visits for stable patients. Given the paucity of data regarding the impact of differentiated care models on paediatric outcomes, BIPAI conducted a retrospective analysis of clinical outcomes, comparing monthly (MS) with multi-monthly (MMS) ART prescription schedules for children and adolescents in Botswana, Lesotho, Swaziland, Malawi, Uganda and Tanzania.

**Methods**: MMS was introduced in each country in line with national policy. Patients were transferred to MMS when clinically stable and ART adherent, after six to nine months of monthly prescriptions. For analysis, patients were allocated to the MMS group after three consecutive visits at intervals of greater than 56 days. Adherence, lost-to-follow-up rates, CD4 counts and viral load were compared between MS and MMS patients by two-sample tests for binomial proportions. Mortality was compared by log rank test. To avoid bias against the MS groups, deaths in the first six months of MS therapy were excluded, given the known, high early rates of mortality. To avoid immortal time bias, MMS patients contributed person–time to the MS group between ART initiation and the start of MMS. The analysis was conducted according to an IRB approved protocol.

**Results**: There were 11,421 MS and 18,137 MMS patients aged between 0 and 19 years. Comparison of clinical outcomes is displayed in Table 1.

**Abstract MOAD0105–Table 1.** Clinical outcomes of MS and MMS patients.

Variable	MS patients	MMS patients	*p* Value
Mean interval between visits (SD)	39 days (27.5)	61 days (34.9)	
% of patients with good adherence by pill count (95–105%)	68.7% (7846/11,421)	78.5% (14,238/18,137)	<0.0002
Lost-to-follow-up (%)	7.1% (811/11,421)	1.8% (326/18,137)	<0.0002
Mortality (deaths per 100 patient-years)	2.9	0.4	<0.0001
CD4 counts (% reaching >350 or >25% for under age 5 years	78.0% (8312/10,653)	92.8% (16,767/18,067)	<0.0002
Viral load (% undetectable)	63.3% (2976/4703)	78.9% (10,787/13,678)	<0.0002

MMS patients had statistically lower mortality and lost-to-follow-up rates, as well as superior ART adherence rates and response to ART by CD4 counts and viral load measurements.

**Conclusions**: This study, representing data from six African countries, provides reassurance that patients 0–19 years of age who are clinically stable and ART adherent, can do well with reduced clinical visits via MMS. The consequent reduction in visits can yield additional benefits by decreasing the burden on health systems and patient time.

## MOAD0201

### Why do not key populations access HIV counselling and testing centres in Nepal? Findings based on national surveillance survey

R Shrestha ^1^; S Philip^2^; HD Shewade^3^; B Ojha^1^; K Badal^4^; BB Rawal^5^ and K Deuba^6^

^1^Public Health and Environment Research Center, Kathmandu, Nepal. ^2^Government T.D. Medical College, Kerala, India. ^3^International Union Against Tuberculosis and Lung Disease (The Union), New Delhi, India. ^4^UNAIDS, Kathmandu, Nepal. ^5^National Center for AIDS and STD Control, Ministry of Health, Kathmandu, Nepal. ^6^Karolinska Institutet, Stockholm, Sweden

Presenting author email: rachanashrestha2@gmail.com

**Background**: UNAIDS proposed the 90-90-90 targets, that is, that by 2020, 90% of PLHIV will know their HIV status, 90% of PLHIV who know their HIV status will receive HIV treatment and 90% of PLHIV on ART should be virally suppressed. It is estimated that three million new HIV infections and three million AIDS-related deaths could be averted if these targets are achieved. HIV testing and counselling (HTC) is the entry point for HIV care services in Nepal and is provided free of cost to all. This study assesses the demographic, psychosocial and structural factors associated with non-utilization of HTC by female sex workers (FSW) and men who have sex with men/transgender (MSM/TG) in Nepal.

**Methods**: We analysed data from the national surveillance survey which included 610 FSW and 400 MSM/TG recruited from 22 and 3 districts of Nepal, respectively. Adjusted prevalence ratio (PR) using modified Poisson regression was used to assess the association between independent and outcome variables (non-utilization of HTC in last year). FSW was recruited using two-stage cluster sampling, whereas MSM/TG was recruited using respondent-driven sampling.

**Results**: Non-utilization of HTC in last one year was 54% for FSW and 55% for MSM/TG. The prevalence of depression among study populations was very high (≥50%). About 2 of every 10 FSW experienced forced sex in the last 12 months. The significant factors for FSW related to non-utilization of HTC were: depression [PR = 1.4 (1.1–1.6)], injection of drugs (ever) [PR = 1.4 (1.1–1.8)], episode of forced sex in previous year [PR = 1.1 (1.0–1.3)], presence of dependents in the family [PR = 1.1 (1.0–1.3)] and participation in HIV awareness programmes (ever) [PR = 1.2 (1.0–1.4)]. Non-utilization of HTC among MSM/TG had significant association with age 16–19 years [PR = 1.4 (1.1–1.7)], physical assault in previous year [PR = 1.6 (1.3–2.0)], condom use [PR = 1.2 (1.0–1.4)], experience of forced sex [PR = 0.5 (0.3–0.9)] and participation (ever) in HIV awareness programmes [PR = 1.6 (1.3–2.0)].

**Conclusions**: HIV prevention programmes in Nepal need to go beyond condom promotion. Creative strategies should be envisaged for effective behavioural change communication. Psychosocial and structural interventions should be integrated with HIV prevention programmes to support key populations.

## MOAD0202

### Feasibility and acceptability of immediate ART initiation in MSM in West Africa (CohMSM ANRS 12324 - Expertise France)

C Couderc^1^; TTE Dah ^2,3^; F Diallo^4^; MJ-B Kouamé^5^; RMK Agboyibor^6^; A Bernier^7^; M Peeters^1^; E Mensah^6^; N Meda^3^; C Anoma^5^; B Dembélé Keita^4^; B Spire^8^; C Laurent^1^; CohMSM Study Group

^1^Unité TransVIHMI, IRD UMI 233, INSERM U 1175, Université de Montpellier, Montpellier, France. ^2^Association African Solidarité, Ouagadougou, Burkina Faso. ^3^Centre Muraz, Bobo-Dioulasso, Burkina Faso. ^4^ARCAD-SIDA, Bamako, Mali. ^5^Espace Confiance, Abidjan, Cote D’Ivoire. ^6^Espoir Vie Togo, Lomé, Togo. ^7^Coalition Internationale Sida, Pantin, France. ^8^SESSTIM UMR 912, INSERM/IRD/Université Aix-Marseille, Marseille, France

Presenting author email: tertiero81@yahoo.fr

**Background**: Since September 2015, the World Health Organization (WHO) has recommended to initiate antiretroviral treatment (ART) in all adults living with HIV, regardless of WHO clinical stage and at any CD4 cell count. In Africa, few programmes focused on men who have sex with men (MSM). We therefore assessed the feasibility and acceptability of immediate ART initiation in MSM in four West African countries.

**Methods**: A prospective cohort study has been conducted since June 2015 in community-based clinics in Bamako (Mali), Abidjan (Côte d’Ivoire), Lomé (Togo) and Ouagadougou (Burkina Faso). MSM eligibility criteria were as follows: aged 18 years or older, reporting at least one episode of anal intercourse with another man within the previous three months and either HIV seronegative or discovering their HIV infection at study enrolment. HIV-seronegative MSM were offered a quarterly follow-up including clinical examinations, screening for HIV, screening and treatment for sexually transmitted infections, individualized peer-led support for prevention, condoms and lubricants. MSM who discovered their HIV infection at study enrolment and those who seroconverted during the follow-up were offered ART initiation immediately after HIV diagnosis.

**Results**: As of 16 January 2017, 679 MSM were enrolled in the study. Of them, 120 were HIV seropositive at study enrolment and 35 seroconverted during follow-up. The median age of these 155 HIV-infected MSM was 24.5 years (interquartile range [IQR]: 21.8–28.1). A total of 134 (86.5%) MSM initiated ART. The median time from HIV diagnosis was seven days (IQR: 3–16). Only 15 (11.2%) MSM initiated ART more than one month after HIV diagnosis, and 6 (4.5%) MSM after three months. The median CD4 cell count at ART initiation was 433 cells/µl (IQR: 324–535). Few discrepancies were observed between the study sites. MSM mainly received EFV+TDF+3TC (85.1%) or EFV+TDF+FTC (13.4%). Of 57 MSM with available data, 5 were resistant to EFV, of which 2 were also resistant to 3TC/FTC.

**Conclusions**: These preliminary results indicate that immediate ART initiation is feasible and well accepted by MSM in West Africa, strengthening the recent WHO recommendation.

## MOAD0203

### Don’t get lost: how peer navigation can link HIV-positive key populations to care and treatment and re-engage those lost to follow-up

T Lillie^1^; S Nemande^1^; H Gbais^2^; S Kersten^2^; D Levitt^1,3^ and F Ndonko ^2,4^

^1^FHI360, District of Columbia, USA. ^2^CARE, Yaounde, Cameroon. ^3^CARE, Atlanta, USA. ^4^LINKAGES, Washington, DC, USA

Presenting author email: ndonko@carecameroun.org

**Background**: Linking key populations (KP), including men who have sex with men (MSM) and sex workers (SW) to HIV care and treatment programmes, and ensuring they adhere to treatment to reach viral suppression are challenges in many developing countries. The peer navigation system in Cameroon was initiated to support enrolment and retention of HIV-positive KPs in the HIV service cascade and to remain adherent to treatment. Peer navigators (PN) are HIV positive, medication-adherent role models who understand and can convey clearly how to access and utilize key services for people living with HIV and their partners, loved ones and children.

**Methods**: Through the USAID- and PEPFAR-funded LINKAGES and CHAMP projects, we pilot tested the peer navigation system in Yaoundé, Cameroon. Working with two community-based organizations, 24 PN were trained to provide quality support to HIV-positive KP. PN are usually HIV positive, may be a member of the KP community, trained and full-time staff. Programmatic data collected between November 2015 and July 2016 before PN was introduced was compared to data collected between August and December 2016 after PN initiation. For the analysis, linkage to HIV treatment before and after PN was reviewed.

**Results**: A total of 838 HIV-positive FSW and 557 HIV-positive MSM accessed services from the two CBOs between November 2015 and December 2016. Preliminary data showed increases in client linkage to care and treatment, which went from roughly 39% of newly diagnosed HIV-positive clients before PN to as high as 82% after PN was introduced. Also, 38% of MSM and 71% of SWs living with HIV that were lost to follow-up before PN were recovered in just four months of PN implementation and linked to treatment.

**Conclusions**: While data are preliminary, they demonstrate the potential effectiveness of peer navigation programmes that are being initiated in 16 countries where LINKAGES is working. More widespread implementation of PN with high-risk populations could accelerate progress towards 90-90-90 goals.

## MOAD0204

### HIV treatment cascade analysis for people who inject drugs in Ukraine: identifying the correlates of continuum

K Dumchev ^1^; O Varetska^2^; T Salyuk^2^ and C Vitek^3^

^1^Ukrainian Institute on Public Health Policy, Kyiv, Ukraine. ^2^Alliance for Public Health, Kyiv, Ukraine. ^3^US Centers for Disease Control and Prevention, Kyiv, Ukraine

Presenting author email: dumchev@uiphp.org.ua

**Background**: PWID constitute over 50% of PLWHIV and continue driving HIV incidence in Ukraine, but data on HIV care continuum are scarce. Integrated bio-behavioural surveys (IBBS) among PWID are conducted biannually since 2007 to evaluate the effectiveness of HIV response. The aim of this study was to identify gaps in the HIV treatment cascade among PWID and identify correlates of receiving HIV care and ART.

**Methods**: This is a secondary analysis of 2015 IBBS data collected in all 27 regions of Ukraine using RDS. Results of rapid HIV testing and self-reported data on HIV status awareness, enrolment into HIV care and treatment were used to construct the cascade. Socio-demographic variables, drug use patterns, risk behaviours and receiving of prevention and other interventions were evaluated as potential correlates of being registered in HIV clinic and receiving ART. Logistic regression was used to test significance of association.

**Results**: A total of 9405 PWID were recruited (25% females) and 22% tested positive for HIV. Of those, 81% reported being aware of their status. Among PWID who agreed to disclose HIV-positive status, 74.2% were registered in HIV care and 46.8% received ART (see Figure 1).

**Abstract MOAD0204–Figure 1.**
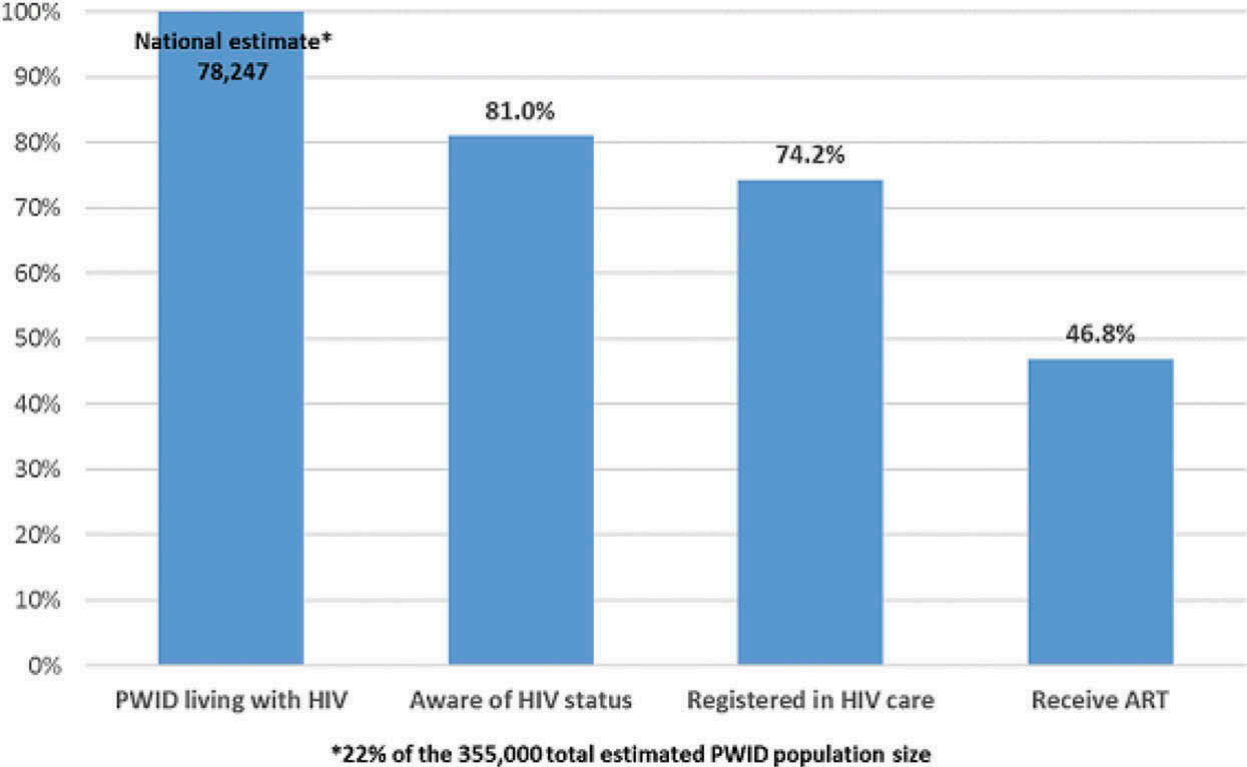
PWID cascade. HIV care and treatment cascade among PWID in Ukraine based on 2015 bio-behavioural survey data.

Being female (aHR = 1.9, 95% confidence interval (CI) 1.02–3.33), age >35 years (aHR = 4.05, 95% CI 1.95–8.41) and being a harm reduction client (aHR = 2.03, 95% CI 1.19–3.46) were positively associated with HIV care registration. Age>35 years (aHR = 2.54, 95% CI 1.49–4.32), being a harm reduction client (aHR = 1.68, 95% CI 1.26–2.24) and current case management (aHR = 1.75, 95% CI 1.04–2.97) were positively associated with receiving ART. Past history of case management was negatively associated with both outcomes (aHR = 0.09, 95% CI 0.04–0.2 and aHR = 0.44, 95% CI 0.22–0.88, respectively). Opioid agonist treatment, drug and alcohol use and risk behaviours were not associated with either outcome.

**Conclusions**: The study confirmed existence of significant gaps in HIV cascade among PWID in Ukraine, especially in access to ART. Services provided at harm reduction programmes, including case management, are important interventions that may improve access to care and treatment.

## MOAD0205

### Effectiveness of comprehensive HIV and stimulant use prevention intervention with Cambodian female entertainment and sex workers

K Page ^1^; A Carrico^2^; E Stein^3^; J Evans^3^; M Sokunny^4^; S Ngak^4^; C Sophal^5^; Y Neak^6^; L Maher^7^ and C McCulloch^3^

^1^University of New Mexico, Albuquerque, USA. ^2^University of Miami, Miami, USA. ^3^University of California, San Francisco, San Francisco, USA. ^4^FHI 360 Cambodia, Phnom Penh, Cambodia. ^5^Department of Mental Health and Substance Abuse, Ministry of Health, Phnom Penh, Cambodia. ^6^National Authority for Combating Drugs, Phnom Penh, Cambodia. ^7^UNSW Australia, Kirby Institute for Infection and Immunity, Sydney, Australia

Presenting author email: pagek@salud.unm.edu

**Background**: HIV prevention services for female entertainment and sex workers (FESW) could serve as a platform for targeting key risk factors for HIV infection, including amphetamine-type stimulant (ATS) use and economic distress. We examined sequentially delivered interventions to decrease ATS use and improve economic well-being to boost HIV risk reduction with FESW.

**Methods**: This cluster-randomized stepped-wedge trial in 10 Cambodian provinces enrolled 1198 FESW to test the effectiveness of a comprehensive HIV and ATS use prevention package leveraging SMARTGirl, an existing HIV prevention platform for Cambodian FESW. The intervention included a conditional cash transfer with cognitive-behavioural aftercare (CCT+AC) intervention to reduce ATS use followed by a microenterprise (ME) opportunity. The co-primary outcomes assessed in 600 FESW purposively targeted for the 18-month follow-up were: (1) self-reported number of sexual partners (past three months) and (2) positive urine toxicology results for ATS (ATS Tox+). After baseline, FESW with problematic patterns of ATS use were allocated to receive a four-month CCT+AC intervention. All FESW who were abstinent from ATS at six months were allocated to receive a ME opportunity.

**Results**: At six months, relative to baseline, participants had 60% lower odds of ATS Tox+ (adjusted odds ratio [AOR] = 0.40; 95% CI 0.25–0.65; *p* < 0.001) and non-significant decreases in number of sexual partners (adjusted risk ratio [ARR] = 0.65; 95% CI 0.38–1.11; *p* = 0.11). At 12 months, FESW reported 50% fewer sexual partners (ARR = 0.50; 95% CI 0.25–0.95; *p* = 0.035) and non-significant reductions in the odds of ATS Tox+ (AOR = 0.58; 95% CI 0.30–1.14; *p* = 0.11). Although FESW continued to display reductions in both primary outcomes at 18 months, these were not statistically significant (*p*’s < 0.09).

**Abstract MOAD0205–Table 1.** Intent-to-treat analyses (*N* = 1198 at baseline).

	No. of sexual partners (3 months)	ATS Tox+
Follow-up assessment	ARR (95% CI)	AOR (95% CI)
6 Months	0.65 (0.38–1.11)	0.40 (0.25–0.65)*
12 Months	0.50 (0.25–0.95)*	0.58 (0.30–1.14)
18 Months	0.45 (0.18–1.14)	0.44 (0.18–1.07)

**Conclusions**: Findings support the robust, short-term effectiveness of the sequentially delivered CCT+AC and ME interventions for optimizing HIV prevention services for Cambodian FESW. Further implementation science research is needed to inform the scale up and improve the durability of this comprehensive approach to boost HIV risk reduction with FESW in South-East Asia.

## MOAD0206

### Viral suppression at the first integrated methadone and antiretroviral therapy programme for people who inject drugs in sub-Saharan Africa

BH Lambdin ^1,2,3^; S Hassan^4^; D Mushi^5^; A Cooke^6^ and J Mbwambo^5^

^1^RTI International, Behavioral and Urban Health, San Francisco, USA. ^2^University of California, San Francisco, USA. ^3^University of Washington, Seattle, USA. ^4^Yale University, School of Medicine, New Haven, USA. ^5^Muhimbili University of Health and Allied Science, Dar es Salaam, United Republic of Tanzania. ^6^University of California, Los Angeles, USA

Presenting author email: blambdin@rti.org

**Background**: The prevalence of HIV among people who inject drugs (PWID) in Dar es Salaam is 42% compared to 7% in the general population. In 2011, an opioid treatment programme (OTP), using methadone, was established in Tanzania to reduce HIV risk behaviours and transmission. Enrolment of PWID into the OTP programme surged, but linking and sustaining HIV-positive, eligible OTP patients in antiretroviral therapy faced many obstacles. We report the results from the first integrated methadone and antiretroviral therapy (IMAT) programme to improve sustained ART access for PWID in sub-Saharan Africa.

**Methods**: A community engagement process with patients, providers and government stakeholders helped us to collaboratively define an integrated model, including: opt-out HIV screening, OTP providers trained in HIV clinical management and monitoring, multiple ART dispensing models from the OTP clinic and intensive case management for people who were not virally suppressed. We assessed viral suppression 6 and 12 months after implementation of the integrated model and used logistic regression to assess predictors of viral suppression (<1000 copies/ml) at 12 months.

**Results**: Of the 126 people receiving HIV treatment at OTP, the median age was 35 years old, and 17% were female. Overall, 72% (95% confidence interval (CI): 60–82) of patients had achieved viral suppression after six months of receiving HIV care at the OTP clinic, and after 12 months, 81% (95% CI: 69–88) had achieved viral suppression. Regarding ART dispensing models, 79% received monthly supplies, while 21% received directly administered ART (DAART) at the pharmacy or by a nurse. Age (*p* = 0.323), sex (*p* = 0.814), time in treatment (*p* = 0.683) and ART regimen (*p* = 0.263) were not significantly associated with viral suppression. Intensive case management was associated with a 16% (95% CI: 1–32; *p* = 0.0362) increase in viral suppression within three months of the case management session.

**Conclusions**: Our findings show a high level of viral suppression among people accessing HIV treatment within the IMAT programme. The success of IMAT was driven by early engagement of OTP patients and providers. Future work will develop differentiated models of care for people receiving ART to achieve sustained viral suppression among HIV-positive PWID without overburdening the region’s health system.

## MOAX0101

### Self-testing: an effective means of increasing HIV-testing and status awareness

A Moore ^1^; T Cassidi^2^; SJ Steele^2^; A Shroufi^2^; N Ntuli^2^; L Ndani^2^; C Metcalf^2^; T Ellman^2^; E Goemare^2^ and L Trivino Duran^2^

^1^MSF, Medical, Cape Town, South Africa. ^2^MSF, Cape Town, South Africa

Presenting author email: msfocb-khayelitsha-doc2@brussels.msf.org

**Background**: HIV self-testing (HST) could potentially improve HIV testing uptake and awareness of serostatus, especially if targeted towards patients who refuse routinely offered facility-based HIV counselling and testing (HCT) due to privacy concerns. We conducted a pilot study of HST at two health facilities in Khayelitsha, South Africa, among patients who refused HCT, and assessed their HST uptake and linkage to care.

**Methods**: Patients who refused HCT were offered HST using OraQuick ADVANCE HIV-1/2. Participants were asked to report their HST result by pre-paid text message (SMS) or by returning to the facility. Participants who did not report their result within seven days were contacted telephonically.

**Results**: From 1 March 2016 to 31 October 2016, 537 patients were offered HST, of whom 422 (78%) accepted. Those who accepted HST had a median age of 28 years; 409 (97%) were female; and 313 (74%) reported their HST result. Of the 422 participants, 245 (58%) reported their result within seven days, and the median time to reporting the result was 1 day. Of those who reported their result, 269 (86%) reported by SMS. Reporting of results varied by facility, being 60% at the facility where many patients did not carry a cell phone due to security concerns, compared to 83% at the other facility. Among participants who reported their HST result, 19 (6%) were positive compared to 7% HCT at same facilities. Of the 19 participants reporting a positive HST result, 10 (53%) returned for confirmatory testing. Of the 294 participants who reported a negative HST result, 53 (18%) returned for confirmatory testing. All confirmatory tests agreed with the reported HST results.

**Conclusions**: Offering HST in public-sector clinics is an effective way of increasing HIV testing uptake among those who refuse HCT, but ensuring that those with positive HST results return for confirmatory testing is challenging. As the majority of patients attending clinics are female, the effectiveness of providing HST in other settings needs to be assessed as a means of increasing HIV testing uptake among males.

## MOAX0102

### HIV self-testing: feasibility and acceptability of a large-scale national service delivered by a community organization

C James; D Edwards; W Harris and M Brady

Terrence Higgins Trust, Health Improvement, London, UK

Presenting author email: cary.james@tht.org.uk

**Background**: The UK needs a dramatic increase in HIV testing to reduce undiagnosed HIV and late diagnoses. HIV self-testing offers the potential to significantly increase the number and frequency of tests.

**Methods:** We piloted a national HIV self-testing service, which was delivered online to men who have sex with men (MSM) and black Africans living in the UK. A dedicated website was created, and the service was promoted through social media. Participants provided demographic information, contact details and answers to HIV risk assessment questions. An HIV self-testing kit was then posted to them. Service users were asked to log onto a secure page on the website to inform us of their result. Anyone with a reactive result was called for support or advice and to ensure access to care for confirmatory testing. An online satisfaction survey was sent to everyone who gave consent.

**Results**: The pilot ran from 24 June to 5 August 2016. A total of 4879 kits were ordered; 3021 people (62%) informed us of their result. Nineteen per cent never had an HIV test before and a further 37% had last tested >1 year ago. Sixty-eight per cent reported condomless anal sex in the previous three months with 28% reporting this with two or more partners. Twenty-eight people (0.92%) reported a reactive result. Three (10.7%) people already knew that they were HIV positive, and one result was confirmed as false positive. Of the remaining 24, all were MSM. Fifteen of 24 (62.5%) were identified as white British. Contact was made with 22 (92%) all of whom had accessed confirmatory testing and HIV services. About 602 people responded to the survey. Ninety-eight per cent would use the service again, 91% felt self-testing encouraged them to test and 91% were happy with the support they received.

**Conclusions**: We have demonstrated the feasibility and acceptability of HIV self-testing in the UK. It also demonstrated that a high percentage were willing to report their results which allowed for confirmation of linkage to care. We believe that an investment in HIV self-testing will compliment existing options and provide a cost-effective way to scale up our approach to testing.

## MOAX0103

### The impact of HIV self-testing among Internet-recruited MSM, eSTAMP 2015 to 2016

RJ MacGowan ^1^; PR Chavez^1^; CB Borkowf^1^; PS Sullivan^2^ and JH Mermin^1^

^1^Centers for Disease Control and Prevention, NCHHSPT/DHAP, Atlanta, USA. ^2^Emory University, Atlanta, USA

**Background**: Knowledge of HIV status is essential for accessing antiretroviral therapy and effective prevention services. Providing HIV rapid diagnostic tests (RDTs) for self-testing to persons at risk of HIV infection, such as men who have sex with men (MSM), could increase the frequency and timeliness of HIV testing. The “Evaluation of HIV self-testing among MSM Project” (eSTAMP) is a 12-month randomized controlled trial (RCT) that evaluates the impact of this strategy.

**Methods**: MSM in the US were recruited online from March through August 2015 and enrolled into eSTAMP. Participants were asked to complete online surveys at baseline and 3, 6, 9 and 12 months. The intervention group was mailed four RDTs at baseline with the option of replenishing the ones used after interim assessments. At the end of the study, all participants who completed the 12-month survey were mailed two RDTs and a dried blood spot (DBS) card. We compare the percentage of participants who tested ≥3 times, mean number of all HIV tests, percentages who accessed clinic-based HIV testing services, mean number of sex partners over 12 months and newly identified cases of HIV infection by intervention and control groups.

**Results**: We randomly assigned 2665 MSM to the Intervention (*n* = 1325) and Control (*n* = 1340) arms. Mean age was 30.4 years; 58% were white, 10% black, 23% Hispanic and 9% other or mixed race; and 17% had never been tested for HIV. Seventy-two per cent completed at least one follow-up survey; retention rate at 12 months was 58%. There was significantly more HIV testing in the intervention group. Forty-two cases of HIV infection were identified; 21 were linked to care.

**Abstract:** MOAX0103–Table 1.

Results over 12-month follow-up period	Intervention	Control	*p* Value
Number of newly identified HIV cases^a^, *n*/*N* (%)	25^b^/966 (2.6%)	11^b^/958 (1.2%)	0.03
MSM reporting ≥3 HIV tests, *n*/*N* (%)	761/965 (79%)	217/958 (22%)	<0.01
Number of HIV tests, mean (SD)	5.3 (3.6)	1.5 (1.8)	<0.01
MSM reporting clinic-based HIV tests, *n*/*N* (%)	395/966 (41%)	614/958 (64%)	<0.01
Number of sex partners, mean (SD)	9.1 (17.0)	9.7 (19.7)	0.57

^a^Includes RDT and clinic-based testing among study participants. ^b^Additional cases (Intervention: *n* = 3, Control: *n* = 3) were identified from the DBS testing after the 12-month survey.

**Conclusions**: Implementing Internet-based HIV screening programmes with free HIV RDT increased HIV testing and diagnosis among MSM, including those who have not previously accessed traditional HIV testing services.

## MOAX0104

### Feasibility of HIV self-test programming among female sex workers in Zimbabwe

S Mavedzenge ^1^; E Sibanda^2^; J Dirawo^2^; K Hatzold^3^; O Mugurungi^4^ and F Cowan^2,5^

^1^RTI International, Women’s Global Health Imperative, San Francisco, USA. ^2^Centre for Sexual Health and HIV/AIDS Research (CeSHHAR), Harare, Zimbabwe. ^3^Population Services International, Harare, Zimbabwe. ^4^Ministry of Health and Child Care, Harare, Zimbabwe. ^5^Liverpool School of Tropical Medicine, International Public Health, Liverpool, UK

Presenting author email: smavedzenge@rti.org

**Background**: Female sex workers (FSW) are disproportionately affected by HIV, yet their engagement in HIV services does not reflect this heightened risk. Increasing HIV testing is the first step towards prevention and care services. There is little research on HIV self-testing (HIVST) among FSW, which may be particularly appropriate for this population. We conducted a pilot study offering HIVST for six months to FSW in Zimbabwe to evaluate programmatic feasibility.

**Methods**: Adult FSW of unknown HIV status presenting for testing at a dedicated FSW clinic were given the option of provider-delivered testing or HIVST. Those opting for HIVST and who had a mobile phone were invited to enrol. Participants received self-test kits and validated instructions. They were contacted after two weeks to complete a questionnaire about their experience.

**Results**: A total of 607 FSW presented for testing and 325 (54%) opted for HIVST (*p* < 0.01). Among self-testers, mean age was 29 years (range 18–62). Most (94%) had previously tested for HIV; 100% reported the test was not difficult to use, and 98% were comfortable learning their result without a provider present. Thirty per cent had a reactive result, and of those, 99% had attended post-test services by the two-week post-test questionnaire; 100% indicated they would want HIVST to be available to them and would recommend HIVST to family/friends. Eighty-one per cent would recommend HIVST to their clients. Though no participants were forced to self-test, 38% thought coercive testing might happen if HIVST became more widely available. FSW thought HIVST distribution should be via clinic (62%), pharmacy (18%), peer (14%) and/or workplace (13%). FSW indicated they would be willing to pay $0.50–$25 for self-tests, with 35% willing to pay $1 and 30% $5.

**Conclusions**: FSW found HIVST highly acceptable and wanted HIVST to be available to them. A high proportion had a reactive self-test, and importantly, virtually everyone had linked to post-test services by the two-week follow-up questionnaire. Some expressed concern about potential for coercive testing. FSW were willing to pay for HIVST and provided useful insight into how to distribute and promote HIVST during future implementation research. HIVST represents a promising strategy to promote regular re-testing among FSW in Zimbabwe.

## TUAA0101

### Evaluation of memory CD8+ T cell responses in individuals initiating cART during hyperacute HIV-1 infection

TP Nkosi ^1^; K Pretorious^1^; N Mewalal^1^; T Ndung’u^1,2^ and Z Ndhlovu^1,2^

^1^University of KwaZulu-Natal, HIV Pathogenesis Programme (HPP), Durban, South Africa. ^2^Ragon Institute of MGH, MIT and Harvard, Massachusetts General Hospital and Harvard Medical School, Massachusetts, USA

Presenting author email: thandekankosi78@gmail.com

**Background**: Previous studies have shown that the emergence and proliferation of antigen-specific CD8+ T cells during early stages of HIV infection are associated with persistent antigenaemia. Early initiation of combination antiretroviral therapy (cART) confers clinical benefit to HIV-infected persons, but the impact of cART on HIV-specific immune responses and the potential for recall or boosting of these responses are unknown. In this study, we evaluated the maintenance of CD8+ T cell responses longitudinally in early treated individuals with hyperacute HIV-1 subtype C infection.

**Methods**: Samples of young females identified with acute HIV-1 infection (HIV PCR positive, antibody negative) who initiated cART very early and untreated patients were used. The magnitude, breadth and maintenance of HIV-1-specific CD8+ T cell responses were defined using IFN-gamma *ex vivo* ELISPOT and cultured ELISPOT. Also, the phenotype (HLA-DR, CD38 and CD127) and functional (IFN-gamma) characteristics of tetramer-specific CD8+ T cells were investigated using MHC class I tetramers and intracellular cytokine staining (ICS).

**Results**: Early treated patients with hyperacute infection induced initial CD8+ T cell responses that coincided with a sharp drop in viraemia and an increase in CD4 counts. These early induced CD8+ T cell responses were however low in magnitude (144.3 SFC/million PBMC) when compared to untreated patients (316.6 SFC/million PBMC (*p* = 0.009)). Interestingly, compared to untreated patients, CD8+ T cells in early treated patients were less activated and had a high expression of CD127, thus suggesting a potential for long-term survival of these responses. Additionally, memory responses specific to HIV-1 measured at later stages (six months onwards) of infection were maintained in these treated patients as indicated by cultured ELISPOT assays.

**Conclusions**: Summarily, our results demonstrate that early initiation of cART led to an induction of CD8+ T cell responses that were less activated and had higher potential for long-term survival. These responses were also maintained as memory responses which may be recalled rapidly upon re-stimulation with HIV-1 antigens. These data may offer insight in implementing novel therapeutic strategies in order to enhance protective immunity and promote control of viral replication post-treatment interruption.

## TUAA0102

### Early anti-SIV CD8^+^ T cell antiviral activity is associated with durable elite control of SIV infection in macaques carrying or not protective MHC alleles: the ANRS SIC study

C Passaes ^1^; A Millet^2^; V Madelain^3^; V Monceaux^1^; A David^1^; P Versmisse^4^; N Sylla^5,6^; M Ploquin^1^; D Duffy^6,7^; C Joubert^5,6^; A Blancher^8^; N Bosquet^5,6^; R Le Grand^5,6^; G Pancino^4^; M Muller-Trutwin^1^; J Guedj^3^; V Avettand-Fenoel^2^; C Rouzioux^2^; B Vaslin^5,6^ and A Sáez-Cirión^1^

^1^Pasteur Institute, Unité HIV, Inflammation et Persistance, Départements de Virologie et Immunologie, Paris, France. ^2^EA 7327, Université Paris-Descartes, AP-HP, Service de Virologie Hôpital Necker-Enfants Malades, Paris, France. ^3^INSERM UMR 738 - University Paris Diderot, Paris, France. ^4^Pasteur Institute, Unité de Régulations des Infections Rétrovirales, Paris, France. ^5^CEA, DRF/iMETI, Fontenay aux Roses, France. ^6^Center for Immunology of Viral Infections and Autoimmune Diseases-Inserm U1184 (Joint Research unit CEA-Université Paris Sud-INSERM) and IDMIT, Fontenay aux Roses, France. ^7^Pasteur Institute, Laboratoire Immunobiologie des Cellules Dendritiques, Département d’Immunologie, Paris, France. ^8^Université Toulouse III - Paul Sabatier, Laboratoire d’Immunogénétique Moléculaire, Toulouse, France

Presenting author email: cpereira@pasteur.fr

**Background**: Natural control of infection has been associated with efficient HIV/SIV-specific CD8^+^ T cell responses. However, the determinants leading to the development of such responses and the role of protective HLA alleles are still unclear.

**Methods**: We monitored for 18 months 16 Mauritius cynomolgus macaques (CM) after infection with SIVmac251 and studied immunological and virological events leading to durable control of infection. CM receiving a low viral dose (nonH6 5AID_50_, *n* = 4) or carrying the protective H6 MHC haplotype (H6 50AID50, *n* = 6), both conditions leading frequently to control of viraemia, were compared to H6-negative CM exposed to the higher dose of the virus (nonH6 50AID50, *n* = 6).

**Results**: Twelve CM spontaneously controlled plasma viraemia (VL) below 400 SIV-RNA copies at six months p.i. No differences were found in VL or SIV-DNA in blood at the VL peak (day 15 p.i.) between SIV controllers (SIC) and non-controllers, but these levels correlated with levels during the chronic phase, and controllers had a faster viral decline to set point. SIC had lower levels of SIV-DNA in lymph nodes at day 15 and in all tissues analysed at the end of the study. SIC and non-controllers had a different cytokine profile during the follow-up. All animals developed SIV-specific CD8 T-cell responses (measured by ICS) coinciding with the start of VL decline at primary infection; however, no differences could be found between SIC and non-controllers. In contrast, an efficient capacity of CD8 T cells to eliminate infected CD4 T cells was developed preferentially in SIC (both H6 and non-H6) and its magnitude increased overtime coinciding with the establishment of elite control. Moreover, SIV-suppressive capacity of CD8+ T cells at day 15 and 70 p.i. negatively correlated with VL at day 15 (in the first case) and at the end of the study (in both cases). This activity was stronger in SIC at the end of the study in all tissues.

**Conclusions**: We provide here unprecedented insight into the dynamic development of effective CD8 T cell responses against SIV. Initial enhanced capacities of CD8 T cells to suppress SIV infection shaped viral levels during primary infection and increased over time until reaching levels allowing viral control.

## TUAA0103

### CD8^+^ T cell depletion leads to a different profile of SIV viral decay under integrase inhibitor monotherapy

B Policicchio ^1,2^; E Fabian Cardozo^3^; C Xu^1^; D Ma^1^; T He^1^; K Raehtz^1^; R Sivanandham^1^; A Kleinman^1^; G Haret-Richter^1^; T Dunsmore^1^; C Apetrei^1^; I Pandrea^1^ and R Ribeiro^3,4^

^1^University of Pittsburgh, Center for Vaccine Research, Pittsburgh, USA. ^2^University of Pittsburgh, Infectious Diseases and Microbiology, Pittsburgh, USA. ^3^Los Alamos National Laboratory, Theoretical Biology and Biophysics Group, Los Alamos, USA. ^4^University of Lisbon, Laboratorio de Biomatematica, School of Medicine, Lisbon, Portugal

Presenting author email: bbp6@pitt.edu

**Background**: How CD8^+^ T cells control virus during HIV infection is not understood. We hypothesized that the main effect of CD8^+^ T cells occurs before viral integration, due to minimal direct viral cytopathic effects. We developed a model of viral dynamics with pre- and post-integration stages to study the effect of CD8^+^ T cell depletion. Model predictions were tested in SIV-infected rhesus macaques (RMs) receiving integrase inhibitor raltegravir (RAL) monotherapy with or without CD8^+^ T cells.

**Methods**: Sixteen SIVmac251-infected RMs were treated with both RAL- and CD8-depleting antibody M-T807R1 (RD), or just RAL (R) and followed, with RAL treatment interrupted after 23 days. Plasma viral loads (VLs) were measured by qRT-PCR. T-cell counts and immune activation were monitored flow-cytometrically. We analysed the VLs during the first approximately 12 days following RAL initiation using a viral dynamic model including infected cells pre- and post-viral DNA integration. We fitted the model to the data using a nonlinear mixed-effect model to estimate the death rate of infected cells pre- and post-virus integration and the efficacy of RAL.

**Results**: CD8^+^ T cell depletion was profound and lasted throughout RAL therapy. Depletion of CD8^+^ T cells led to an increase in VL prior to the start of therapy. Macaques receiving just RAL treatment had much greater decays in VL than those treated with RAL- and the CD8-depleting antibody. The latter group had small decays or rebounded early during RAL therapy. From the fits of the model, we estimated the efficacy of RAL in blocking integration at 96.3%, the half-life of virus-producing cells at approximately 13 h and a different loss rate of infected cells pre-integration in the two groups (0.0016/day vs. 0.19/day, for DR and R groups, respectively). A model allowing for these different loss rates was better than a model with a common loss rate for both groups (*p* = 0.0001).

**Conclusions**: Use of RAL monotherapy revealed that the turnover of infected cells pre-integration has a half-life of about 3.6 days. However, in the absence of CD8^+^ T cells, this half-life reaches >100 days. These results suggest that CD8^+^ T cells have a strong cytolytic effect on infected cells before viral integration.

## TUAA0104

### Memory-like NK cells exploit innate priming and alternative signalling mechanisms to enhance function and mobilize at HIV/SIV mucosal portals of entry

S Shah; C Manickam; A Jimenez and RK Reeves

Beth Israel Deaconess Medical Center, Harvard Medical School, Boston, USA

Presenting author email: roger_reeves@hms.harvard.edu

**Background**: Burgeoning evidence indicates a broader functional repertoire for NK cells beyond innate immunity including memory and other memory-like functions. One recent example is memory-like NK cells identified by lack of the FcR intracellular γ-signalling chain (FcRΔg-NK cells) which still require antibody to grant antigen specificity but are pre-sensitized and capable of rapid mobilization and more robust responses against viral antigens. Interestingly, FcRΔg-NK cells are initially expanded by huCMV infection as part of innate-priming, but can execute memory-like killing against other pathogens through incompletely understood mechanisms.

**Methods**: Sixty rhesus macaques were used in this study: 21 specific pathogen free, rhCMV-; 10 rhCMV+ but otherwise experimentally naive; and 22 chronically SIVmac-infected macaques. Samples were analysed from 10 naive and 10 untreated HIV-infected human subjects. NK cell analyses were performed using polychromatic and phospho-flow cytometric phenotypic and functional assays.

**Results**: FcRΔg-NK cells were systemically distributed in mucosal and secondary lymphoid organs but, correlating with viral load, increased two- and fourfold in CMV+ and HIV/SIV-infected individuals, including the GI tract. CD16 and α4β7 were concomitantly upregulated in infection, suggesting that innate memory-like priming is required for both antibody-dependent functional potency and mucosal homing. FcRΔg-NK cells displayed little difference in binding affinity to virus-antibody immunocomplexes compared to traditional NK cells, but exhibit twofold more robust IFN-γ secretion and cytotoxicity, suggesting disparate signalling or activation could account for improved function. To that end, FcRΔg-NK cells showed significantly reduced expression of Helios and Eomes - indicative of a broader functional repertoire and/or epigenetic modification, and clustered independently from traditional NK cells in 20-parameter analyses via multidimensional *t-SNE*. The γ-chain adaptor, Syk, was reduced or inactively dephosphorylated in FcRΔg-NK cells, but expression of ζ-chain, which is phosphorylated by adaptor Zap70, was significantly upregulated, suggesting that these cells may exploit the ζ-chain/Zap70 pathway in the absence of γ-chain/Syk to achieve greater functional potency.

**Conclusions**: Collectively, our work presents the first description of a combinatorial mechanism of innate-priming and alternative signalling cascade to explain the functional potency of memory-like phenomena of NK cells mobilizing in the mucosae against HIV/ SIV. Future studies harnessing memory-like NK cells could create exciting modalities for both vaccine and curative therapies.

## TUAA0105

### The human penis is an immunologically active tissue: a preliminary study on the development of an HIV vaccine

A Sennepin ^1,2,3^; F Real^1,2,3^; M Duvivier^1,2,3^; Y Ganor^1,2,3^; S Henry^1,2,3^; D Damotte^4^; M Revol^5^; S Cristofari^5^ and M Bomsel^1,2,3^

^1^Cochin Institute, INSERM U1016, Department of Mucosal Entry of HIV-1 and Mucosal Immunity, Cell Biology, and Host Pathogen Interactions, Paris, France. ^2^CNRS, UMR 8104, Paris, France. ^3^Paris Descartes University, Sorbonne Paris Cite, Paris, France. ^4^GH Cochin-Saint Vincent de Paul, Anatomy and Pathological Cytology Service, Paris, France. ^5^Saint Louis Hospital, Plastic Surgery Service, Paris, France

Presenting author email: alexis.sennepin@inserm.fr

**Background**: HIV-1 is primarily sexually transmitted. We have shown that the human penis, including the foreskin but also the urethra, fossa navicularis and glans, is one of the main portal of entry for the virus. Unlike other mucosa, the penile immune system and mechanisms that induce a penile immune response remain unclear, most likely due to the difficulty to access human tissues. Our previous studies demonstrated that the male urethra contains macrophages, the main targets of HIV-1, as well as memory T cells. These studies relied on morphologic analyses and thus failed to provide a comprehensive phenotype. To assess the role of these mucosal immune cells that are a prerequisite to the elaboration of efficient preventive strategies against HIV-1, we characterize extensively the immune profile of immune cells of the different penile regions.

**Methods**: Single-cell suspensions were prepared for each region of 32 penile tissues collected from individuals undergoing transgender surgery and analysed by multi-parametric flow cytometry. The expression patterns of memory, activating and homing receptors of B and T lymphocytes were evaluated as well as that of NK cells. In complement, the tissue distribution of each of these immune populations in the different penile compartments was also studied morphologically.

**Results**: In all penile compartments, CD3-/CD19+ B cells represent around 2% of CD45+ cells and >50% B cells display CD27 and FcRL4 receptors and thus harbour a memory phenotype. However, <5% are IgG+ or IgA+ and thus able to secrete antibodies in the lamina propria. TCD4+ and TCD8+ lymphocytes represent the major populations of CD45+ cells, with 90% with a CD38-/HLADR-/CCR7-/CD45RA-resting effector memory phenotype (T_EM_). These resting T_EM_ cells reside in all penile region epithelium and lamina propria but lack CD103+ resident phenotype. Furthermore, all penile compartments contain low numbers of CD3-/CD56+ NK cells capable of antibody-dependent cell cytotoxicity and surface expressing the NKp44 receptor indicative of activation.

**Conclusions**: Altogether, the human penis is an immunologically active tissue including the cellular machinery required to induce/produce a specific and effective immune response against mucosal pathogen. This must be taken considered when elaborating efficient vaccine strategies against HIV-1.

## TUAB0101

### Efficacy and safety of switching from boosted protease inhibitor plus emtricitabine/tenofovir disoproxil fumarate regimens to the single-tablet regimen of darunavir/cobicistat/emtricitabine/tenofovir alafenamide (D/C/F/TAF) in virologically suppressed, HIV-1-infected adults through 24 weeks: EMERALD study

J-M Molina ^1^; J Gallant^2^; C Orkin^3^; E Negredo^4^; L Bhatti^5^; J Gathe^6^; E Van Landuyt^7^; E Lathouwers^7^; V Hufkens^7^; S Vanveggel^7^ and M Opsomer^7^

^1^University of Paris 7, Denis Diderot, Paris, France. ^2^Southwest CARE Center, Santa Fe, USA. ^3^Barts and Health NHS Trust, London, United Kingdom. ^4^Germans Trias i Pujol University Hospital, Badalona, Spain. ^5^AIDS Healthcare Foundation, Beverly Hills, USA. ^6^Therapeutic Concepts, Houston, USA. ^7^Janssen Pharmaceuticals NV, Beerse, Belgium

Presenting author email: jean-michel.molina@aphp.fr

**Background**: D/C/F/TAF, a once-daily, single-tablet regimen containing darunavir (D 800mg), cobicistat (C 150mg), emtricitabine (F 200mg) and tenofovir alafenamide (TAF 10mg), is undergoing investigation in two phase 3 studies: EMERALD (NCT02269917) and AMBER (NCT02431247).

**Methods**: EMERALD, a randomized (2:1), open-label, international, multicentre, parallel-group, non-inferiority, 48-week study, is evaluating the efficacy and safety of switching to D/C/F/TAF vs. continuing a boosted protease inhibitor plus emtricitabine/TDF (control) in patients who are virologically suppressed (viral load (VL) <50 c/ml) for ≥2 months. The FDA-stipulated primary endpoint is proportion with cumulative virologic rebound (confirmed VL ≥50 c/ml or premature discontinuations, with last VL ≥50c/ml) through week 48 (non-inferiority margin = 4%). Pre-planned week 24 interim results are presented.

**Results**: A total of 1141 patients were randomized and treated (*N* = 763 D/C/F/TAF vs. *N* = 378 control). Baseline characteristics: median age 46 years; 18% women; 25% non-white (21% black); 10% CD4^+^ <350 cells/mm^3^; and 71%, 22% and 8% on darunavir, atazanavir and lopinavir, respectively (15% on cobicistat). Cumulative virologic rebound was 1.8% (*n* = 14 D/C/F/TAF) vs. 2.1% (*n* = 8 control), of which 10/14 and 5/8, respectively, resuppressed (<50 c/ml) by week 24; there were no confirmed rebounds ≥200 c/ml. At week 24, the FDA snapshot analysis showed that virologic suppression (VL <50 c/ml) was 96.3% (D/C/F/TAF) and 95.5% (control), and virologic failure occurred in 0.5% and 0.8%, respectively, with no discontinuations for virologic failure and no detected resistance to any study drug. Safety was similar between arms through 24 weeks, with low incidences of grade 3–4 adverse events (AEs) (D/C/F/TAF 4.5% vs. control 4.5%), serious AEs (2.6% vs. 3.2%) and treatment discontinuations (overall, 2.9% vs. 2.9%; due to AEs, 1.4% vs. 1.1%). The most common AEs (≥5% both arms) were: nasopharyngitis (7.6% vs. 6.6%), URI (6.3% vs. 6.3%) and vitamin D deficiency (5.5% vs. 5%). There were no deaths. Total cholesterol/HDL-cholesterol ratios were similar between arms, with minimal changes from baseline. Changes from baseline in renal safety parameters were consistent with known profiles of the individual D/C/F/TAF components: mean ΔeGFR (cystatin-C clearance by CKD-EPI): +0.3 ml/min/1.73 m² (D/C/F/TAF) vs. −1.0 ml/min/1.73 m² (control).

**Conclusions**: In virologically suppressed adults, switching to once-daily D/C/F/TAF was well tolerated, resulted in a low cumulative virologic rebound rate and a high virologic suppression rate through 24 weeks.

## TUAB0102

### Switching from a boosted protease inhibitor (PI/r)-based regimen to a dolutegravir regimen in virologically suppressed patients with high cardiovascular risk or age ≥50 years is non-inferior and decreases lipids

JM Gatell ^1^; L Assoumou^2^; G Moyle^3^; L Waters^4^; E Martinez^5^; H-J Stellbrink^6^; G Guaraldi^7^; S de Wit^8^; F Raffi^9^; A Pozniak^10^; NEAT022 Study Group

^1^Hospital Clinic/IDIBAPS, University of Barcelona, Infectious Diseases, Barcelona, Spain. ^2^Sorbone Universites, INSERM, UPMC Univ Paris 06, IPLESP UMRS 1136, Paris, France. ^3^Chelsea and Westminster Hospital, London, United Kingdom. ^4^Mortimer Market Center, London, United Kingdom. ^5^Hospital Clinic/IDIBAPS, University of Barcelona, Barcelona, Spain. ^6^Infectiologisches Centrum, Hamburg, Germany. ^7^University of Modena and Reggio Emilia, Modena, Italy. ^8^Saint Pierre University Hospital, Université Libre de Bruxelles, Brussels, Belgium. ^9^CHU Hotel-Dieu Nantes, Nantes, France. ^10^Chelsea & Westminster Hospital, London, UK

Presenting author email: jmgatell@clinic.cat

**Background**: Switching from a PI/r to dolutegravir (DTG) may improve convenience and lipid profile.

**Methods**: NEAT022-NCT02098837 is a European, open-label, randomized non-inferiority trial. HIV-infected adults ≥50 years or with a Framingham score ≥10% were eligible if HIV RNA <50 copies/ml for at least 24 weeks while on a PI/r regimen. Patients were randomized (1:1) to switch to DTG or to remain on PI/r. Primary endpoints were proportion of patients with HIV RNA <50 copies/ml at week 48 and a non-inferiority margin of −10% and percentage change of total plasma cholesterol. Secondary end points included changes in other plasma lipid fractions and adverse events.

**Results**: A total of 415 patients were randomized: 205 to DTG and 210 to continue PI/r; 89% were men, 87% were ≥50 years and 74% had a Framingham score >10% and suppressed viraemia for a median of five years. At week 48, in the intention-to-treat analysis, treatment success rate was 93% in DTG arm and 95% in PI/r arm (difference −2.0%, 95% confidence interval −6.5 to 2.6, non-inferiority demonstrated). There were four virological failures with DTG (from 58 to 130 copies) and one with PI/r (3373 copies) without selection of resistance. There was no significant difference in terms of grade 3 or 4 AEs or treatment-modifying AEs (seven in DTG arm - of whom six due to mood disturbances or insomnia - and three PI/r arm). Total cholesterol and other lipid fractions (except HDL) significantly (*p* < 0.001) improved in the DTG arm overall and in all baseline PI/r strata. About 30% were on lipid-lowering agents at weeks 0 and 48 in each arm.

**Abstract TUAB0102–Figure 1.**
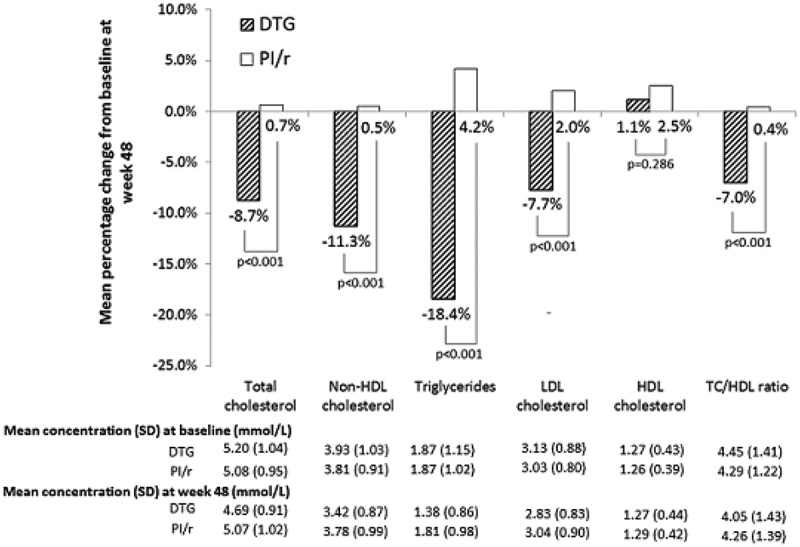
Changes in lipid fractions at 48 weeks.

**Conclusions**: Switching from a PI/r-based regimen to a DTG regimen in virologically suppressed HIV patients ≥50 years old or with a Framingham score ≥10% was non-inferior, was well tolerated and improved the lipid profile.

## TUAB0103

### Efficacy of dual therapy with protease inhibitors plus lamivudine as maintenance treatment in HIV-positive patients on second line in Africa: the ANRS 12286/MOBIDIP trial 96-week results

L Ciaffi ^1^; S Koulla Shiro^2^; A Sawadogo^3^; C T Ndour^4^; S Eymard-Duvernay^5^; S Izard^1^; V Le Moing^5,6^; J Zoungrana^3^; R Toby^2^; C Kouanfack^7^; M Mpoudi^8^; NF Ngoum Gueye^9^; M Diallo^4^; G Bado^3^; K Toure Kane^10^; A Aghokeng^11^; M Peeters^5^; J Reynes^5,6^ and E Delaporte^5,6^

^1^IRD UMI 233, INSERM U1175, Université de Montpellier, Unité TransVIHMI, Yaounde, Cameroon. ^2^Central Hospital Yaounde, Infectious Diseases, Yaounde, Cameroon. ^3^Souro Sanou University Hospital, Hôpital de Jour, Bobo-Dioulasso, Burkina Faso. ^4^Fann University Hospital, CRCF, Dakar, Senegal. ^5^IRD UMI 233, INSERM U1175, Université de Montpellier, Unité TransVIHMI, Montpellier, France. ^6^Montpellier University Hospital, Infectious Diseases, Montpellier, France. ^7^Central Hospital Yaounde, Hôpital de Jour, Yaounde, Cameroon. ^8^Military Hospital Yaounde, Hôpital de Jour, Yaoundé, Cameroon. ^9^Fann University Hospital, Hôpital de Jour, Dakar, Senegal. ^10^A Le Dantec University Hospital, Laboratoire de Bactériologie-Virologie, Dakar, Senegal. ^11^IRD UMI 233, INSERM U1175, Université de Montpellier, Virology Laboratory IMPM-IRD, Yaounde, Cameroon

Presenting author email: lauraciaffi2002@yahoo.fr

**Background**: In the MOBIDIP trial, dual therapy with boosted protease inhibitors (bPI) plus lamivudine showed superiority to bPI monotherapy in maintenance of virologically controlled HIV-positive patients on second-line antiretroviral treatment (ART) at 48 weeks. At week 48, the monotherapy arm was interrupted on DSMB advice, and patients on dual therapy continued their follow-up until week 96. Here, we present the results of the dual therapy arm at 96 weeks.

**Methods**: This open-label trial, conducted in Cameroon, Senegal and Burkina Faso, randomized 265 patients on stable PI plus NRTI second-line ART with HIV-1 RNA (VL) <200 copies/ml, CD4 >100 cell/mm^3^ and adherence >90%, to receive ongoing ritonavir-boosted PI (darunavir or lopinavir) or on going bPI plus lamivudine. The main outcome was failure rate at 96 weeks in the intention-to-treat (ITT) population. Failure was defined as (1) a confirmed VL above 500 copies/ml (VF), (2) reintroduction of the NRTI backbone or (3) interruption of bPI.

**Results**: At inclusion, the 132 patients in the dual arm were mainly women (70%), median CD4 was 472 (interquartile range (IQR) 360–621) cell/mm^3^, 83% had VL <50 copies/ml, median time on second line was 38 (IQR 30–47) months and PI was darunavir (one third) or lopinavir (two third). At first-line failure, 97% had the M184V mutation. At 96 weeks, in ITT analysis, 8.3% (95% confidence interval 4.2–14.4) patients failed in the dual arm (8 VF, 1 death, 2 lost to follow-up). Median delay to failure was 60 weeks. Three of four patients who reintroduced tenofovir had VL <200 copies/ml in a median time of 13 weeks. At 96 weeks, 79% and 91% of patients had VL below 50 and 200 copies/ml, respectively. Median increase in CD4 was 62 cells/mm^3^. We registered 28 severe adverse events, 1 in relation with study drugs. No significant changes in metabolic parameters were observed.

**Conclusions**: After viral suppression with bPI plus NRTIs in second-line therapy, maintenance with bPI plus lamivudine is associated with a high rate of long-term success despite the presence of M184V mutation.

## TUAC0101

### Long-term follow-up of PROUD: evidence for high continued HIV exposure and durable effectiveness of PrEP

E White^1^; D Dunn^1^; R Gilson^2^; A Sullivan^3^; A Clarke^4^; I Reeves^5^; G Schembri^6^; N Mackie^7^; C Dewsnap^8^; C Lacey^9^; V Apea^10^; M Brady^11^; J Fox^12^; S Taylor^13^; J Rooney^14^; M Gafos^1^; ON Gill^15^; S McCormack ^1,16^; PROUD Study Group

^1^MRC CTU at UCL, UCL, London, United Kingdom. ^2^Mortimer Market Centre, Central and North West London NHS Foundation Trust, London, United Kingdom. ^3^St Stephen’s Centre, Chelsea & Westminster NHS Foundation Trust, London, UK. ^4^Royal Sussex County Hospital, Brighton & Sussex University Hospitals NHS Trust, Brighton, UK. ^5^Homerton University Hospital NHS Foundation Trust, London, UK. ^6^Manchester Centre for Sexual Health, Central Manchester University Hospitals NHS Foundation Trust, Manchester, UK. ^7^St Mary’s Hospital, Imperial College Healthcare NHS Foundation Trust, London, UK. ^8^Sheffield Teaching Hospitals NHS Foundation Trust, Sheffield, UK. ^9^York Teaching Hospital and Hull York Medical School, University of York, York, UK. ^10^Ambrose King Centre and Barts Sexual Health Centre, Barts Health NHS Trust, London, UK. ^11^King’s College Hospital NHS Foundation Trust, London, UK. ^12^Guy’s and St Thomas’ NHS Foundation Trust, London, UK. ^13^Birmingham Heartlands Hospital, Heart of England NHS Foundation Trust, Birmingham, UK. ^14^Gilead Sciences, Foster City, USA. ^15^HIV & STI Department, Public Health England Centre for Infectious Disease Surveillance and Control, London, UK, ^16^56 Dean Street, Chelsea and Westminster Hospital NHS Foundation Trust, London, UK

Presenting author email: s.mccormack@ucl.ac.uk

**Background**: The PROUD trial clearly demonstrated the clinical effectiveness of TDF/FTC in the first year of use. The continued follow-up of participants on PrEP (range two to four years), which included regular testing for HIV and other STIS, allows assessment on whether effectiveness is maintained in the longer term and the extent of potential exposure to HIV.

**Methods**: PROUD was a pragmatic trial in which MSM were randomized to receive daily TDF/FTC either immediately (IMM) or after a deferral (DEF) period of 12 months. Main efficacy findings were based on follow-up during the deferred phase when IMM had access to PrEP and DEF did not. Since November 2014, when all participants were offered PrEP, the trial has entered a post-deferred phase. We compare incidence rates of HIV and selective STIs during the deferred and post-deferred phases.

**Results**: A total of 524 (269 IMM, 255 DEF) and 449 (244 IMM, 205 DEF) participants contributed to the deferred and post-deferred phases. Of 368 who attended a clinic in the last six months of follow-up, 327 (89%) had at least one PrEP prescription. HIV and rectal gonorrhoea (rGC)/chlamydia (rCT) incidence in each phase is shown by group in the Table 1.

**Abstract TUAC0101–Table 1.** HIV and STI incidence (per 100 PY).

	Deferred phase		Post-deferred phase	
Infection	IMM	DEF	IMM	DEF
**HIV**	1.6 (4/254)	9.4 (21/223)	1.2 (5/423)	0.3 (1/353)
**Rectal GC**	35.3 (81/229)	33.1 (67/203)	31.4 (129/411)	32.7 (116/355)
**Rectal CT**	33.6 (77/229)	21.2 (43/203)	33.1 (136/411)	29.9 (106/355)

There was no difference in HIV incidence between the groups in the post-deferred phase (*p* = 0.18), but a significant decrease in the DEF group once they had access to PrEP (*p* < 0.0001). The rate in the IMM group remained similar in the two phases (*p* = 0.66). The incidence of rectal infections was high in both groups and phases. rCT was lowest in the DEF group during the deferred phase, and this was driven by those who did not report rCT in the year before enrolment.

**Conclusions**: The reduction in HIV incidence in the DEF group confirms the remarkable effectiveness of TDF/FTC. The relatively stable incidence in the IMM group indicates that this effect is durable. High ongoing incidence of rCT/rGC shows that participants remained at high risk of HIV, and this needs to be taken into account when planning PrEP provision in public health programmes.

## TUAC0102

### On-demand PrEP with TDF/FTC remains highly effective among MSM with infrequent sexual intercourse: a sub-study of the ANRS IPERGAY trial

G Antoni ^1^; C Tremblay^2^; I Charreau^1^; E Cua^3^; D Rojas-Castro^4^; N Hall^5^; J Chas^6^; T Huleux^7^; B Spire^8,9^; C Capitant^1^; L Cotte^10^; L Meyer^1,11,12^; J-M Molina^13,14^; ANRS IPERGAY Study Group

^1^INSERM, SC10-US19, Villejuif, France. ^2^Centre Hospitalier de l’Université de Montréal, Montréal, Canada. ^3^Hôpital de l’Archet, Centre hospitalier de Nice, Department of Infectious Diseases, Nice, France. ^4^Association AIDES, Pantin, France. ^5^CHU Hôtel Dieu, Nantes, France. ^6^Hôpital Tenon, Department of Infectious Diseases, Paris, France. ^7^Hôpital G. Dron, Centre Hospitalier Universitaire de Tourcoing, Department of Infectious Diseases, Lille, France. ^8^INSERM, UMR 912 SESSTIM, Marseille, France. ^9^ORS PACA, Marseille, France. ^10^Department of Infectious Diseases, Hôpital de la Croix Rousse, Centre Hospitalier et Universitaire de Lyon, Lyon, France. ^11^Department of Medicine, Université Paris Sud, Kremlin Bicêtre, France. ^12^Assistance Publique Hôpitaux de Paris, Hôpital Bicêtre, Service d’Epidémiologie et de Santé Publique, Kremlin Bicêtre, France. ^13^Department of Infectious Diseases, Hôpital Saint Louis, Assistance Publique Hôpitaux de Paris, Paris, France. ^14^Université de Paris Diderot, Paris, France

Presenting author email: guillemette.antoni@inserm.fr

**Background**: The ANRS IPERGAY trial demonstrated among MSM a 86% relative reduction of HIV-1 incidence with on-demand PrEP in the TDF/FTC arm (2 infections, 219 person-years (py) of follow-up (FU), incidence: 0.91/100 py) as compared to the placebo arm (14 infections, 212 py of FU, incidence: 6.60/100 py). Participants in this trial used a median of 15 pills/month and had a median of 10 sexual intercourse/month. We wished to investigate whether on-demand PrEP remained effective among participants having less frequent sexual intercourse and using fewer pills.

**Methods**: Assuming that participants with less frequent sexual intercourse would use fewer pills, and because individual patterns of pill use showed large intra-participant variability over time, we focused our analysis on person-time between two consecutive visits when participants used ≤15 pills/month and PrEP was used “systematically or often” during sexual intercourse and not “from time to time or never”. We then cumulated in each arm FU time spent with this pattern of pill use. A fourth-generation HIV-1/2 ELISA assay was performed at each visit allowing to date the time of HIV infection. Incidence rates of HIV infection/100 py in both arms were then compared using mid-*p* exact test.

**Results**: Six HIV-1 infections occurred during FU among participants using ≤15 pills/month taken “systematically or often” during sexual intercourse: 6 in the placebo arm (incidence: 9.3/100 py, total FU time: 64.8 py) and 0 in the TDF/FTC arm (incidence: 0/100 py, total FU time: 68.9 py, *p* = 0.013). The relative reduction of HIV incidence was 100% (95% confidence interval: 20–100). During these follow-up periods, a median of 5 (IQR: 2–10) sexual intercourse/month were reported, and a median of 9.5 (IQR: 6–13) pills/month were used. Restricting the analysis to periods when participants reported at least one condomless sexual act yielded similar results with HIV incidence of 12.3/100 py in the placebo arm (6 infections, 48.8 py of FU) and 0/100 py in the TDF/FTC arm (0 infection, 54.3 py of FU, *p* = 0.011).

**Conclusions**: On-demand PrEP with TDF/FTC remains highly effective in MSM having infrequent sexual intercourse.

## TUAC0103

### An open-label multiple-dose phase 1 assessment of long-acting rilpivirine

I McGowan ^1^; CS Dezzutti^1,2^; A Siegel^2^; J Engstrom^2^; C Shetler^2^; N Richardson-Harman^3^; K Abebe^1^; D Back^4^; L Else^4^; D Egan^4^; S Khoo^4^; PE Williams^5^; RM Brand^1^; BA Chen^1,2^; SL Achilles^1,2^ and RD Cranston^1^

^1^University of Pittsburgh, Pittsburgh, USA. ^2^Magee-Womens Research Institute, Pittsburgh, USA. ^3^Alpha StatConsult, Damascus, USA. ^4^University of Liverpool, Liverpool, UK. ^5^Janssen Research and Development, Beerse, Belgium

Presenting author email: imcgowan@pitt.edu

**Background**: Long-acting (LA) injectable formulations of antiretroviral agents are being developed for HIV-1 prevention. The MWRI-01 phase 1 study was undertaken to characterize the safety, acceptability, pharmacokinetic (PK) and pharmacodynamic (PD) profile of LA rilpivirine (RPV). Single-dose (SD) data have previously been reported (McGowan I et al. Lancet HIV 2016). We now present data on the multiple-dose (MD) phase of the study.

**Methods**: HIV-1-uninfected participants received three intramuscular doses of 1200mg LA RPV at two-month intervals. We collected plasma, genital/rectal fluids and tissue (rectal (RT), cervical (CT) and vaginal (VT)) before and after exposure to LA RPV for assessment of PK and PD (*ex vivo* biopsy challenge with HIV-1). Clade B (HIV-1_BaL_) and Clade C (G147-1) viruses were used separately in the explant challenge model. The primary study objective was to characterize product safety, and the analysis included all enrolled participants.

**Results**: We enrolled eight women and four men. There were 195 adverse events reported, of which 193 (99%) were grade 1 (71%) or grade 2 (28%), and the majority were related to injection-site discomfort. Table 1 provides the PK values (geometric mean; 90% confidence interval) for each compartment 56 days after dosing.

**Abstract TUAC0103–Table 1.** **PK data 56 days after injection (ng/**ml).

	Plasma		RT		VT	CT
	Women	Men	Women	Men	Women	Women
**Dose 1**	39 (33–45)	29 (17–40)	46 (34–53)	29 (17–40)	22 (14–29)	28 (23–33)
**Dose 3**	59 (45–73)	40 (30–51)	61 (52–70)	40 (30–51)	40 (28–52)	44 (22–66)

We found significant suppression of viral replication in RT for both Clade B (*p* < 0.05) and Clade C (*p* < 0∙0001) viruses at all time points in RT. In contrast, viral suppression was only seen in CT at day 56 after the first dose of LA-RPV and at no time point for VT.

**Conclusions**: MD administration of LA RPV was safe and well tolerated. As with our previous SD data, we saw prolonged suppression of viral replication in RT following exposure to LA RPV. Interestingly, despite modest accumulation of plasma RPV over time, there was minimal evidence of viral suppression in CT or VT.

## TUAC0104

### Impact of microbiota on female genital tissue and plasma concentrations of dapivirine

S Hillier ^1,2^; L Meyn^1^; K Bunge^1^; M Austin^2^; B Moncla^1,2^; C Dezzutti^1,2^; B Devlin^3^; M Marzinke^4^; C Hendrix^4^ and L Rohan^2,5^

^1^University of Pittsburgh, Obstetrics, Gynecology and Reproductive Sciences, Pittsburgh, USA. ^2^Magee-Womens Research Institute, Pittsburgh, USA. ^3^International Partnership for Microbicides, Silver Spring, USA. ^4^Johns Hopkins University, Baltimore, USA. ^5^University of Pittsburgh, Pharmaceutical Science, Pittsburgh, USA

Presenting author email: hillsl@mwri.magee.edu

**Background**: Women having a non-*Lactobacillus-*dominant vaginal microbiota enrolled in CAPRISA 004 had lower detection of tenofovir (TFV) in cervicovaginal lavage (CVL) fluid than women having a *Lactobacillus-*dominant microbiota. In FAME-04, decreased concentrations of TFV diphosphate in genital tissues and TFV in the plasma were highly correlated to higher Nugent score and increased vaginal concentrations of *Gardnerella vaginalis* and *Atopobium vaginae*. Vaginal rings containing dapivirine, a nonnucleoside reverse-transcriptase inhibitor, have been shown to reduce incident HIV. The objective of this secondary analysis was to evaluate whether vaginal microbiota associated with bacterial vaginosis similarly impacted dapivirine concentrations in genital tract tissues and plasma following vaginal application.

**Methods**: Twenty-four healthy HIV-negative women (mean age 27, 58% white) used either dapivirine 0.05% gel (1.25mg) or films (1.25mg) for six days at home. On the seventh day, women inserted the final dose in the clinic with confirmation of correct product placement. Two hours later, cervical and vaginal biopsies along with CVL and plasma were obtained for dapivirine quantification using a validated liquid chromatography tandem mass spectrometry assay. Vaginal samples for diagnosis of bacterial vaginosis using the Nugent criteria and quantitative polymerase chain reaction (qPCR) detection of *G. vaginalis* and *A. vaginae* were collected prior to product use. The relationship between vaginal microbiota and dapivirine levels was assessed using linear regression models.

**Results**: There was no association between increasing concentrations of *G. vaginalis* in the vagina detected by qPCR and dapivirine concentrations in vaginal tissue, cervical tissue, CVL or plasma (*p* = 0.45, 0.93, 0.51 and 0.99, respectively). Similarly, vaginal concentrations of *A. vaginae* were not associated with dapivirine concentrations in CVL, vaginal and cervical tissues or plasma (*p* ≥ 0.31). Nugent criteria associated with bacterial vaginosis were not associated with lower CVL and tissue or plasma concentrations of dapivirine (*p* ≥ 0.19).

**Conclusions**: In contrast to tenofovir, genital and plasma concentrations of dapivirine were not impacted by increasing concentrations of vaginal bacteria associated with bacterial vaginosis. While replication of these results is needed, these data suggest that the levels of dapivirine following vaginal application should not be impacted by the microbiota associated with bacterial vaginosis.

## TUAC0105

### Experiences and perceptions of PrEP among gay and other men who sex with men (MSM) using PrEP in the PROUD study in England

M Gafos ^1^; W Nutland^2^; S Wayal^3^; G Bell^4^; M Rayment^5^; C Rae^5^; S McCormack^1^ and R Horne^6^

^1^MRC Clinical Trials Unit at UCL, Institute of Clinical Trials & Methodology, London, UK. ^2^London School of Hygiene and Tropical Medicine, London, UK. ^3^UCL, London, UK. ^4^She, Sheffield, UK. ^5^Chelsea and Westminster Hospital NHS Foundation Trust, Directorate of HIV/GU Medicine, London, UK. ^6^UCL, School of Pharmacy, London, UK

Presenting author email: m.gafos@ucl.ac.uk

**Background**: There are concerns that PrEP could increase risk compensation, especially reducing condom use. The PROUD study (November 2012 to November 2016) reported an 86% reduction in HIV and no increase in sexually transmitted infections. We explore PROUD participants’ experiences and perceptions of PrEP in relation to other risk reduction strategies.

**Methods**: We conducted semi-structured in-depth interviews with 41 HIV-negative MSM, purposively selected based on self-reported high/low PrEP adherence and increased/same risk behaviour. Interviews were digitally recorded, transcribed and analysed using framework analysis.

**Results**: The majority of participants reported risk reduction strategies including occasional condom use, strategic positioning or sero-sorting. Participants applied rules to their sexual behaviour, such as using condoms “if it was a one night stand”, or not being receptive “outside of a relationship”. Typically, PrEP was added to the existing set of “rules”. For some participants, PrEP allowed a relaxing of the rules, for example, about strategic positioning: “I have definitely experienced more as a bottom”, or about condomless sex: “I have had more unprotected sex than before … it doesn’t mean that I only have unprotected sex”. Other participants insisted PrEP had not changed their rules: “I haven’t changed the way I think because I am taking this pill”. Participants described PrEP as a “security blanket”, an added “defence mechanism” and used analogies such as wearing a “crash helmet … on my bicycle”. PrEP was described as affording “more intimacy”, “reassurance” and giving “added control”. By using PrEP, many participants with HIV-positive partners sought to reduce their partner’s anxiety about the risk of transmission. The benefits of PrEP were described within the social context of risk environments in cities like London, the chemsex scene and the digitization of sexual contact. PrEP use was viewed as time-limited: “clearly it is a period, a moment … it is not going to be a lifetime”.

**Conclusions**: These data suggest that PrEP was added to a range of ‘rules’ already used to mitigate risk, rather than replacing them. PrEP impacted on the boundaries of the rules for some people but not all. In social contexts of high-risk behaviour, PrEP offers added protection and psychosocial benefits that increase individual choice in the mitigation of risk.

## TUAC0201

### High uptake of community-based HIV testing by adolescent girls and young women aged 15–24: implications and synergies for PrEP rollout?

A Medina-Marino ^1,2^; A Mumbauer^3^; T Farirai^4^; L-G Bekker^5^; S Johnson^6^ and N Nkhwashu^4^

^1^Foundation for Professional Development, Research Unit, Pretoria, South Africa. ^2^University of Pretoria, School of Health Systems and Public Health, Pretoria, South Africa. ^3^Foundation for Professional Development, Strategic Information Department, Pretoria, South Africa. ^4^Foundation for Professional Development, HIV Prevention Department, Pretoria, South Africa. ^5^University of Cape Town, The Desmond Tutu HIV Centre (DTHC), Cape Town, South Africa. ^6^Foundation for Professional Development, Technical Assistance Cluster, Pretoria, South Africa

Presenting author email: andrewm@foundation.co.za

**Background**: HIV incidence among female youth aged 15–24 in South Africa is four times higher than their male counterparts. Recent HIV prevention trials in South Africa documented incidence of 5% to 6% per year in 15–24-year-old adolescent girls and young women (AGYW). HIV counselling and testing is the entry point for treatment and prevention services and is key to implementing effective HIV prevention strategies. Community-based HIV counselling and testing (CBCT) has the potential to increase testing among key populations. We present interim findings of our at-scale CBCT programme targeting high transmission areas in 13 high HIV burden districts in South Africa.

**Methods**: Routine programmatic data from October 2015 to September 2016 were used. Descriptive statistics were performed. HIV positivity and testing uptakes rates were calculated and stratified by age group, gender and district.

**Results**: A total of 660,351 individuals were tested (positivity = 6.6%; uptake = 54.9/1000 population). The largest number of testers were individuals age 25–49 (*n* = 309,323; uptake = 68.2/1000 population). The highest testing uptake was by individuals age 15–24 (uptake = 90.6/1000 population). Further disaggregation into 15–19- and 20–24-year-old age groups reveal that adolescent girls aged 15–19 (uptake = 91.6/1000 population) and young women aged 20–24 (uptake = 112.8/1000 population) had the highest testing uptake of any age groups of either gender (Figure 1). These finding persist when uptake was disaggregated by district.

**Conclusions**: Implementation of our CBCT programme has been extremely successful. Though the largest number of tests was performed on those aged 25–49 years, AGYW had the highest testing uptake. If access to PrEP by AGYW is to be scaled up, innovative supply-side interventions and service delivery platforms must be identified. Stakeholders involved in PrEP implementation should consider the synergies that CBCT programmes may provide for identifying and delivering PrEP services to AGYW.

**Abstract TUAC0201–Figure 1.**
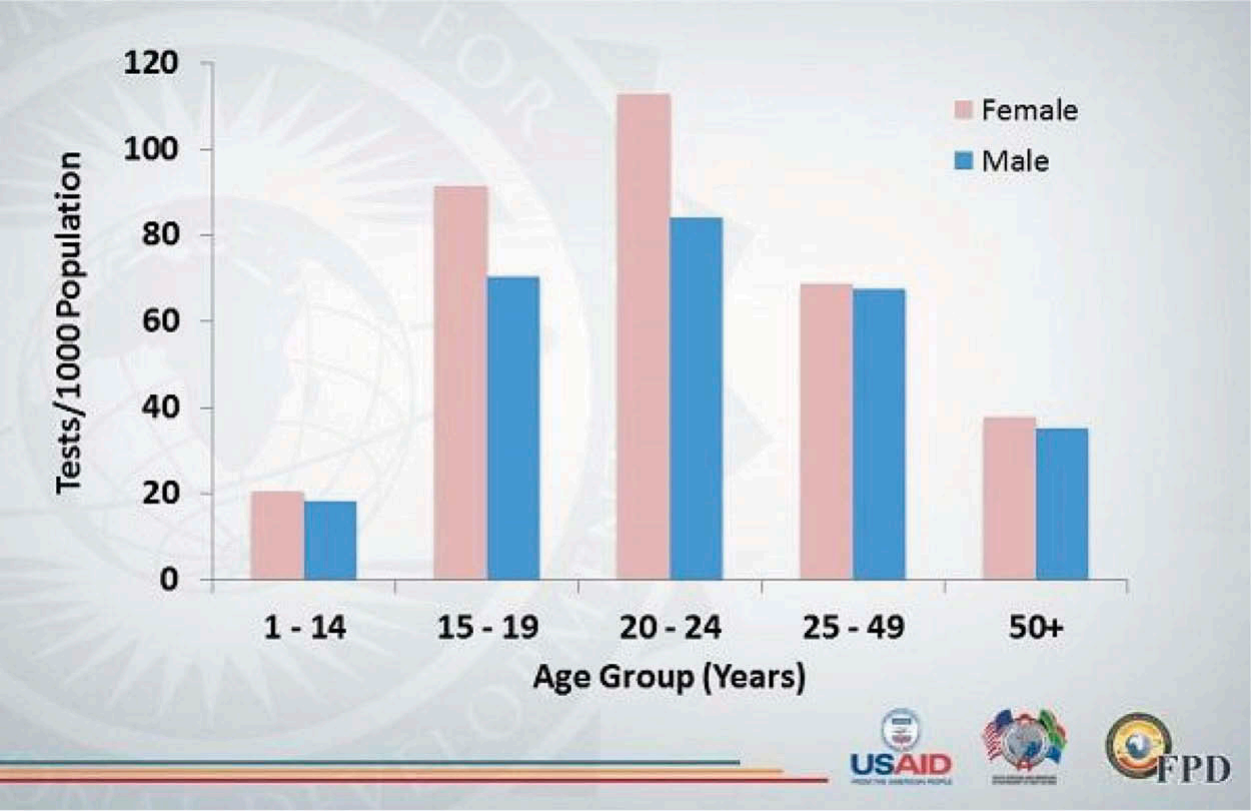
Testing uptake by age group and gender.

## TUAC0201

### Finding the right target population for PrEP: the cost-effectiveness of pre-exposure prophylaxis provision to female and male adolescents and young women in South Africa

G Meyer-Rath ^1,2^; L Jamieson^2^ and L Johnson^3^

^1^Boston University, Center for Global Health and Development, Boston, USA. ^2^University of the Witwatersrand, Health Economics and Epidemiology Research Office, Johannesburg, South Africa. ^3^University of Cape Town, Centre for Infectious Disease Epidemiology and Research, Cape Town, South Africa

**Background**: The South African government is in the process of identifying a suitable rollout strategy for providing oral PrEP to young people at risk of acquiring HIV. We were tasked to evaluate the cost-effectiveness of providing PrEP to young women (20–24 years) vs. male or female adolescents (15–19 years), both when covering everyone in these groups or only those self-identifying as being at high risk.

**Methods**: We used Thembisa, an existing HIV transmission model, and cost input from the first PrEP demonstration projects to model the impact of PrEP provision on new HIV infections and cost per HIV infection averted over 20 years, over a baseline of the current HIV programme, including potential cost savings due to reduced treatment need. We compared provision to all young people in a sub-population to targeting via self-selection by high-risk individuals. Target coverage was set to 18% of either population and varied in sensitivity analysis.

**Results**: PrEP provision to adolescents of both genders is the most cost-effective option, being more effective and less costly than provision to young women (see Table 1). Provision to female adolescents is slightly more effective and cost-effective than provision to male adolescents. At 18% coverage of either all young people in the target population or only those at high risk, the impact on HIV infections averted is similar, but self-selection by high-risk individuals results in much smaller populations on PrEP. As a result, targeted provision is much more cost-effective. PrEP is however not cost saving at any of the coverage rates tested (1–99%) for any of the populations, with or without successful targeting to high-risk sub-populations.

**Abstract TUAC0201–Table 1.** Results by sub-population.

	Baseline	Young women (20–24)		Adolescents; both genders (15–19)		Adolescents; females (15–19)		Adolescents; males (15–19)	
Risk group		All	High risk	All	High risk	All	High risk	All	High risk
Number of person years on PrEP (millions)	-	8.7	1.4	10.7	3.7	5.9	1.6	4.7	2.1
Total cost (billions USD)	43.86	45.43	44.10	45.67	44.38	44.86	44.06	44.69	44.21
Incremental cost (billions USD) (% change)	-	1.53 (3.5)	0.20 (0.5)	1.76 (4.0)	0.48 (1.1)	0.96 (2.2)	0.16 (0.4)	0.79 (1.8)	0.31 (0.7)
Total new HIV infections (millions)	4.63	4.54	4.56	4.38	4.39	4.47	4.49	4.52	4.53
HIV infections averted (thousands) (% change)	-	82.4 (1.8)	65.61 (1.5)	250.0 (6.1)	230.3 (5.5)	153.4 (3.6)	140.1 (3.2)	106.4 (2.4)	98.7 (2.2)
Incremental cost-effectiveness ratio (USD/HIV infection averted)	-	$18,511	$3013	$7058	$2099	$6264	$1164	$7438	$3144

**Conclusions**: Provision of PrEP to female adolescents is more cost-effective than to male adolescents or young women. PrEP, although expensive, can be made more cost-effective if high-risk sub-populations successfully self-select for PrEP.

## TUAC0203

### State-level school-based sex education policies on sexual orientation are associated with changes in teaching about HIV prevention

A Grosso; D Bermudez and MA Chiasson

Public Health Solutions, Research and Evaluation, New York, USA

Presenting author email: grossoas@gmail.com

**Background**: The Sexuality Information and Education Council of the United States (SIECUS) reported in 2015 that 11 states had state-level laws or policies regarding school-based sex education that were discriminatory towards lesbian, gay, bisexual and/or transgender (LGBT) individuals or stated that homosexuality must not be promoted or addressed as a socially acceptable alternative. This was an 83% increase from the number of states in 2014. The implementation of these policies may limit the HIV education available to LGBT students and their heterosexual peers.

**Methods**: We constructed a panel data set for even-numbered years from 2006 to 2014. The independent variable was an indicator from SIECUS State Profiles about whether states’ sex education laws were LGBT stigmatizing. The three dependent variables were from the Centers for Disease Control and Prevention (CDC) School Health Profiles system. Based on self-administered questionnaires from the principal and the lead health education teacher in a sample of high schools, the CDC reported for each state the percentage of schools that tried to increase students’ knowledge about HIV prevention, human sexuality and sexually transmitted disease (STD) prevention. We conducted ordinary least-squares regression analysis in Stata/SE 14 with state- and year-fixed effects to control for unobserved time-invariant state-level confounders and state-invariant time-varying confounders.

**Results**: In the regression models, having a state sex education policy that was anti-LGBT was associated with a 17.4%, 21.7% and 16.3% decrease in the percentage of schools that tried to increase student knowledge about HIV prevention, human sexuality and STD prevention, respectively (*p* < 0.05).

**Abstract TUAC0203–Table 1.** Sex education implementation by LGBT policy.

Year →	2006	2008	2010	2012	2014	State sex education law is negatively LGBT-biased (combined states and years) ↓	
% (*n*/*N*) of states with negatively LGBT-biased sex education laws →	21.6 (11/51)	13.7 (7/51)	13.7 (7/51)	11.8 (6/51)	11.8 (6/51)	No	Yes
% of schools that tried to increase student knowledge about HIV prevention	81.4	88.9	88.4	86.9	85.9	87.4	81.4
% of schools that tried to increase student knowledge about human sexuality	73.7	84.6	84.2	82.4	80.5	82.4	75.6
% of schools that tried to increase student knowledge about STD prevention	77.5	86.4	86.7	85.7	84.9	85.4	79.4

**Conclusions**: State sex education laws that are negatively biased towards LGBT people may increase reluctance of teachers to try to increase student knowledge about sex education topics in general, including those that are not related to sexual orientation or gender identity. Given the increase in these laws in the past year, we expect that additional teens will be denied instruction on these topics. Further research is necessary to assess how this may affect youth risk behaviours and sexual health outcomes.

## TUAC0204

### Potential for HIV transmission among adolescents and young adults receiving antiretroviral therapy

S Wood ^1,2^; N Dowshen^1,2^; C Gowda^3^; S Lee^4^; S Ratcliffe^5^ and R Gross^6,7^

^1^Children’s Hospital of Philadelphia, Division of Adolescent Medicine, Philadelphia, USA. ^2^Perelman School of Medicine, Department of Pediatrics, University of Pennsylvania, Philadelphia, USA. ^3^Ohio State University College of Medicine, Infectious Diseases, Columbus, USA. ^4^Children’s Hospital of Philadelphia, Craig Dalsimer Division of Adolescent Medicine, Philadelphia, USA. ^5^Department of Biostatistics, University of Pennsylvania Perelman School of Medicine, Epidemiology, and Informativs, Philadelphia, USA. ^6^Departments of Medicine and Epidemiology, Philadelphia, University of Pennsylvania, Perelman School of Medicine, USA. ^7^University of Pennsylvania, Center for Clinical Epidemiology and Biostatistics, Philadelphia, USA

Presenting author email: woodsa@email.chop.edu

**Background**: Adolescents and young adults (AYA) living with HIV have lower rates of virologic suppression and higher rates of sexually transmitted infections (STIs) than older adults, which increases HIV transmission potential. We aimed to identify the proportion of participants with, and risk factors for, high HIV transmission potential within a cohort of HIV-positive AYA.

**Methods**: Retrospective cohort study of HIV-positive, antiretroviral therapy (ART)-treated, AYA, ages 13–24, at a U.S. adolescent HIV clinic from 2002 to 2015. We included all visits with viral load (VL) measurement after ART initiation. High transmission potential was defined as incident STI (*Neisseria gonorrheae, Chlamydia trachomatis* or *Treponema palladium*) with concurrent VL >1500 copies/ml. Generalized estimating equations (GEE) were used to calculate odds ratios (ORs) and 95% confidence intervals (CI) for hypothesized risk factors for high transmission potential, including age, gender, insurance status, sexual orientation, race and history of STI at entry to care.

**Results**: Participants (*n* = 251) were followed for a median of 3.2 years (interquartile range (IQR) 1.5–5.3), contributing 2860 visits. Participants were 87% African-American (*n* = 218), and 73% men and transgender women who have sex with men (*n* = 182) and 48% (*n* = 120) had a history of STI at entry to care. The median visit age was 21 years (IQR 19–23). Incident STI was detected in 68% (*n* = 166) of participants comprising 15% (*n* = 299) of visits. Participants were viraemic (VL >1500 copies/ml) at 27% (*n* = 640) of visits. High transmission potential occurred at least once in 16% (*n* = 39) of participants and 3% of visits. In the final GEE model, history of STI at or before entry to HIV care conferred a nearly fourfold increased odds of high transmission potential (OR 3.8, 95% CI: 2.0–7.1, *p* < 0.001). There was no significant association between age, gender, sexual orientation, race or insurance status and high HIV transmission potential.

**Conclusions**: In this conservative model of transmission potential, 16% of ART-treated AYA in care were episodically at high risk of HIV transmission, demonstrating the limits of treatment as prevention in this setting. A baseline history of STI conferred higher risk of transmitting HIV, emphasizing the need for secondary prevention interventions targeting both ART adherence and sexual risk reduction for HIV-positive youth.

## TUAC0205

### HIV and HSV-2 risk among young women in age-disparate partnerships: evidence from KwaZulu-Natal, South Africa

B Maughan-Brown ^1^; G George^2^; S Beckett^2^; M Evans^3^; C Cawood^4^; D Khanyile^4^; L Lewis^5^ and A Kharsany^5^

^1^Southern Africa Labour and Development Research Unit (SALDRU), University of Cape Town, Cape Town, South Africa. ^2^Health Economics and HIV and AIDS Research Division (HEARD), University of KwaZulu-Natal, Durban, South Africa. ^3^Department of Anthropology, York University, Toronto, Canada. ^4^Epicentre AIDs Risk Management (Pty) Limited, Cape Town, South Africa. ^5^Centre for the AIDS Programme of Research in South Africa (CAPRISA), University of KwaZulu-Natal, Durban, South Africa

Presenting author email: brendan.maughanbrown@gmail.com

**Background**: Young women in sub-Saharan Africa continue to exhibit high HIV prevalence and incidence rates. We explored the role age-disparate partnerships play in HIV infection risk among 15–24-year-old women in an endemic setting in South Africa.

**Methods**: During June 2014–June 2015, a cross-sectional household survey was conducted in KwaZulu-Natal Province, South Africa, comprising 9812 individuals aged 15–49 years. Venous blood samples were collected for HIV antibody and viral load tests and herpes simplex virus type 2 (HSV-2) antibody tests. A partnership was defined as age disparate if the age difference between partners was five years or more. Multiple logistic regression analyses were first used to assess the associations between age-disparate partnerships and both HIV and HSV-2 status - HSV-2 may increase a young women’s risk of HIV infection - among 15–24-year-old women who reported at least one sexual partner (*n* = 1557). The second set of analyses used partnership data reported by men - restricted to ongoing partnerships with 15–24-year-old women (*n* = 1078) - to assess whether age-disparate partners of young women were more likely to be HIV positive with a detectable viral load (≥20 copies/ml) and therefore pose a greater level of risk than age-similar partners.

**Results**: Women who reported any age-disparate partnerships were more likely to test positive for HIV (37% vs. 22%, *p* < 0.01) and HSV-2 (65% vs. 46%, *p* < 0.01). After controlling for, inter alia, age and number of lifetime sexual partners, the odds of young women having HIV (adjusted odds ratio (aOR): 1.56, *p* < 0.01, 95% confidence interval (CI): 1.13–2.17) and HSV-2 (aOR: 1.85, *p* < 0.01, 95% CI: 1.41–2.44) were greater for those who reported age-disparate partnerships. Men in age-disparate partnerships with young women were also more likely to be HIV positive (5–9-year age difference: aOR 2.12, *p* < 0.01, 95% CI: 1.26–3.57; 10+ year age difference: aOR 4.93, *p* < 0.01, 95% CI: 2.67–9.12) and be HIV positive with a detectable viral load (5–9-year age difference: aOR 2.30, *p* < 0.01, 95% CI: 1.36–3.87; 10+ year age difference: aOR: 2.57, *p* < 0.01, 95% CI: 1.34–4.92) compared to men in age-similar partnerships with young women.

**Conclusions**: Results suggest a positive association between age-disparate partnerships and young women’s HIV risk. Expanding treatment and combination prevention, including targeted interventions addressing risk from age-disparate sexual partnering, is vital to reducing HIV incidence amongst young women.

## TUAC0301

### Findings from the 2016 Zambia Population-based HIV Impact Assessment (ZAMPHIA): HIV prevalence, incidence and progress towards the 90-90-90 goals

DT Barradas ^1^; S Gupta^1^; C Moyo^2^; K Sachathep^3^; K Dzekedzeke^4^; T Nkumbula^4^; DB Williams^5^; H Patel^5^; T Dobbs^5^; C Nakazwe^6^; W Kasongo^7^; H Cai^1^; S Kamocha^1^; CB Ndongmo^1^; K Hageman^1^; MA Riggs^1^; ZAMPHIA Study Team

^1^Centers for Disease Control and Prevention, Lusaka, Zambia. ^2^Ministry of Health, Lusaka, Zambia. ^3^ICAP, Mailman School of Public Health, Columbia University, New York, USA. ^4^ICAP, Lusaka, Zambia. ^5^Centers for Disease Control and Prevention, Division of Global HIV and TB, Atlanta, USA. ^6^Central Statistics Office, Lusaka, Zambia. ^7^Tropical Disease Research Centre, Ndola, Zambia

**Background**: Based on modelled estimates, 13% of people in Zambia were living with HIV in 2014. The 2016 Zambia Population-based HIV Impact Assessment (ZAMPHIA) is the first national survey to directly assess the status of Zambia’s HIV epidemic by measuring HIV incidence. Findings from ZAMPHIA and progress towards meeting the UNAIDS 90-90-90 targets, including viral load suppression (VLS), are presented.

**Methods**: A nationally representative household-based sample of 12,310 eligible households was selected in 511 enumeration areas; analyses account for study design. Consenting participants provided demographic and clinical information and blood samples for household HIV testing per national guidelines. HIV-seropositive results were confirmed using the Geenius supplemental assay; viral load and limiting antigen (LAg) avidity EIA testing were performed at a central lab on all HIV-seropositive samples. HIV incidence estimates were based on World Health Organization criteria for recent infection (LAg <1.5 OD units and HIV RNA >1000 c/ml). VLS was defined as HIV RNA <1000 c/ml.

**Results**: In total, 19,029 adults and 7959 children provided interviews and blood samples (response rate: 68%). Participation by eligible adults was higher for women than men (71% vs. 63%, *p* < 0.0001). HIV prevalence estimates among adults aged 15–59 and children aged 0–14 were 12.3% and 1.3%, respectively. Adult HIV incidence was 0.66% (female 1.00%, male 0.33%); mean VLS prevalence among all HIV-seropositive adults was 59.8%. An estimated 67.3% of persons living with HIV (PLHIV) knew their HIV status (first 90), 85.4% of PLHIV who reported knowing their status also reported receiving ART (second 90) and 89.2% of these PLHIV who reported receiving ART were virally suppressed (third 90).

**Conclusions**: Zambia has achieved progress towards meeting the UNAIDS 90-90-90 goals. HIV prevalence is stabilizing; HIV incidence is low; and prevalence of VLS is high. Identification of gaps in testing, ART and viral load suppression is needed to better target expansion of HIV treatment services in Zambia.

**Abstract TUAC0301–Table 1.** Selected findings from 2016 ZAMPHIA.

Indicator	Males	Females	Total
HIV prevalence among adults, % (95% CI)	9.5 (8.8, 10.3)	14.9 (14.0, 15.8)	12.3 (11.6, 12.9)
HIV prevalence among children, % (95% CI)	–	–	1.3 (1.0, 1.6)
HIV incidence among adults, % (95% CI)	0.33 (0.11, 0.56)	1.00 (0.65, 1.36)	0.66 (0.45, 0.88)
Viral load suppression (VLS) prevalence among HIV-positive adults, % (95% CI)	57.4 (53.4, 61.5)	61.3 (58.7, 63.8)	59.8 (57.4, 62.2)
Prevalence of HIV-positive adults who report knowing their HIV status, % (95% CI)	62.8 (58.7, 66.8)	70.0 (67.5, 72.5)	67.3 (64.8, 69.7)
Self-reported ART prevalence among HIV-positive adults who report knowing their HIV status, % (95% CI)	86.2 (83.1, 89.4)	84.9 (82.5,87.4)	85.4 (83.4, 87.4)
VLS prevalence among HIV-positive adults who report ART and knowing their HIV status, % (95% CI)	88.2 (85.1, 91.4)	89.7 (87.7, 91.8)	89.2 (87.4, 91.0)

## TUAC0302

### Correlates of being outside the 90-90-90 cascade among adults aged 15–64 years in Zimbabwe

A Hakim ^1^; E Radin^2^; L Ruangtragool^3^; A Herman-Roloff^4^; N Ahmed^2^; G Musuka^5^; H Dube^5^; M Mhangara^6^; L Gwanzura^7^; S Munyati^7^; E Gonese^8^; A Nwankwo-Igomu^8^; H Patel^1^; K Sleeman^1^; S Kinchen^1^; J Justman^2^; BA Tippett-Barr^8^; ZIMPHIA Survey Group

^1^US Centers for Disease Control and Prevention, Division of Global HIV and Tuberculosis, Atlanta, USA. ^2^ICAP at Columbia University, New York, USA. ^3^ASPPH/CDC Allan Rosenfield Global Health Fellow, U.S. Centers for Disease Control and Prevention, Harare, Zimbabwe. ^4^US Centers for Disease Control and Prevention, Pretoria, South Africa. ^5^ICAP at Columbia University, Harare, Zimbabwe. ^6^Zimbabwe Ministry of Health and Child Care, Harare, Zimbabwe. ^7^Biomedical Research Training Institute, Harare, Zimbabwe. ^8^US Centers for Disease Control and Prevention, Harare, Zimbabwe

Presenting author email: hxv8@cdc.gov

**Background**: Zimbabwe has made great strides in combatting HIV, partially through increasing testing and treatment. To reach the UNAIDS 90-90-90 targets, it is necessary to know who is unaware of their HIV infection, who is not on treatment and who is not virally suppressed in order to engage or re-engage them in HIV services. We identify correlates of being outside this cascade.

**Methods**: The 2015 to 2016 Zimbabwe Population-based HIV Impact Assessment was a cluster-based nationally representative household survey. Face-to-face interviews were conducted with 22,496 adults aged 15–64 years, and blood specimens were collected from 20,572 of them for HIV testing following the national serial rapid testing algorithm of Determine, First Response and Stat-Pak. Treatment status was self-reported, and viral load testing was conducted using Roche Taqman 96. Weighted analysis was conducted in SAS. Variables associated with being outside the 90-90-90 cascade at *p* < 0.1 in bivariate analysis were included in the multivariate model.

**Results**: HIV prevalence was 14.6% among 15–64 year olds. Among HIV-infected participants, 25.8% were unaware of their infection. Among those aware of their infection, 13.2% were not on treatment. Of those on treatment, 13.5% were not virally suppressed. In multivariate analysis, males were more likely than females to be unware of their HIV infection (adjusted odds ratio (AOR): 1.74, 95% confidence interval (CI): 1.43–2.10) as were those aged 15–24 years (AOR: 4.59, 95% CI: 3.13–6.72) and aged 25–34 (AOR: 2.43, 95% CI: 1.73–3.41) compared to those aged 55–64 years. Compared to those ages 55–64 years, those aged 15–24 and 25–34 years were least likely to be on treatment (AOR: 3.88, 95% CI: 1.93–7.80 and AOR: 5.03, 95% CI: 2.85–8.89, respectively). Being on treatment and virally unsuppressed was associated with being male (AOR: 1.57, 95% CI: 1.17–2.09) and being aged 15–24 and 25–34 years compared to 55–64 years (AOR: 2.99, 95% CI: 1.50–6.11, and AOR: 3.82, 95% CI: 2.09–7.01, respectively). Neither province nor urban residence was associated with being outside any step of the cascade. Sex was not associated with being aware and not on treatment.

**Conclusions**: People <35 years and men should be further targeted for HIV testing and additional support for linkage to and retention on treatment.

## TUAC0303

### 90-90-90 targets in HIV-positive women using results from MPHIA: a Malawi success story

N Wadonda-Kabondo ^1^; C West^1^; R Nyirenda^2^; F Chimbwandira^1^; S Nkoka^3^; A Voetsch^4^; T Dobbs^4^; G Chipungu^1^; I Chirwa^5^; M Blackson^6^; G Sundeep^1^; E Radin^7^; MPHIA Study Team

^1^CDC, Lilongwe, Malawi. ^2^Malawi Ministry of Health, Lilongwe, Malawi. ^3^ICAP at Columbia University, Lilongwe, Malawi. ^4^CDC, Atlanta, USA. ^5^National Statistical Office of Malawi, Lilongwe, Malawi. ^6^Malawi National AIDS Commission, Lilongwe, Malawi. ^7^ICAP at Columbia University, New York, USA

Presenting author email: vzn7@cdc.gov

**Background**: While women in Malawi continue to experience a high burden of HIV infection, implementation of Option B+ since 2011 has led to substantial improvement in increasing access to antiretroviral therapy (ART) for HIV-positive women. This study aimed at describing the progress in women towards achieving the UNAIDS 90-90-90 targets in Malawi.

**Methods**: The Malawi Population-Based HIV Impact Assessment (MPHIA) was a two‐stage cluster survey of randomly selected households in Malawi. Data collection occurred from November 2015 to August 2016. Participants answered a questionnaire involving reproductive history, PMTCT/Option B+ and HIV testing and care. The survey involved collection of blood samples, home-based counselling and testing (HBCT) using the national rapid HIV test algorithm followed by laboratory-based confirmation using Geenius™. Incidence was measured using the Lag-Avidity EIA. Viral load suppression (VLS) was defined as <1000 HIV RNA copies/ml. Descriptive analyses were conducted to examine progress towards 90-90-90 parameters in women and accounted for the study design.

**Results**: Of 12,231 eligible women aged 15–64 years, 9956 (81.4%) women were interviewed and tested. Seventy-six per cent (76.3%, 95% confidence interval (CI):74.2–78.9) of women reported knowledge of their HIV-positive status. Of the women who knew their HIV-positive status, 90.0% (95% CI: 87.1–92.9) reported being on ART. Of the women who reported ART use, 92.3% (95% CI: 90.4–94.2) had achieved VLS. Progress towards 90-90-90 targets also varied by age (see Table 1). At population level (irrespective of knowledge of HIV and ART status), prevalence of VLS among HIV-positive women aged 15–64 years was 72.9% (95% CI: 69.9–75.9), highest among older women but much lower in young women. HIV incidence among women was 0.48% (95% CI: 0.20–0.76), which was about 50% greater in women than men (0.25%, 95% CI: 0.05–0.46).

**Abstract TUAC0303–Table 1.** 90-90-90 targets by age group among women.

Age group	Diagnosed	On treatment	Virally suppressed
	% (95% CI)	% (95% CI)	% (95% CI)
15–24 years	58.7 (48.4–69.0)	76.9 (59.0–94.7)	79.3 (68.4–90.2)
25–34 years	72.6 (67.5–77.8)	88.0 (82.0–94.1)	94.5 (92.0–97.0)
35–44 years	84.8 (81.0–88.5)	91.7 (88.5–94.9)	91.7 (88.3–95.1)
45–54 years	76.4 (68.8–84.0)	95.3 (92.3–98.2)	93.6 (89.4–97.7)
55–64 years	85.1 (76.7–93.5)	94.7 (89.3–100.0)	95.3 (88.7–100.0)

**Conclusions**: Although women are at greater risk of HIV infection in the reproductive age group, they seem to get into treatment, stay on treatment and achieve VLS. However, the first 90 was not reached, suggesting that a reasonable number of women are not accessing Malawi’s PMTCT programme, and further efforts are needed to diagnose and treat these cases and prevent new infections.

## TUAC0304

### Children living with HIV in Malawi: first survey-based measurement of national paediatric HIV prevalence and viral suppression

S Jonnalagadda ^1^; G Bello^2^; S Suzue^3^; J Burnett^4^; E Radin^3^; K Brown^1^; J Cuervo-Rojas^5^; F Ogollah^5^; E Kim^6^; D Payne^6^; H Patel^1^; K Sleeman^1^; S Hrapcak^1^; A Voetsch^1^; Malawi Population-based HIV Impact Assessment Study Group

^1^Centers for Disease Control and Prevention, Division of Global HIV and TB, Atlanta, USA. ^2^Malawi Ministry of Health, Lilongwe, Malawi. ^3^ICAP at Columbia University, New York, USA. ^4^Centers for Disease Control and Prevention, Division of HIV/AIDS Prevention, Atlanta, USA. ^5^ICAP at Columbia University, Lilongwe, Malawi. ^6^Centers for Disease Control and Prevention, Division of Global HIV and TB, Lilongwe, Malawi

Presenting author email: wau4@cdc.gov

**Background**: To date, national paediatric HIV burden estimates in Malawi were derived from modelling using clinic-based data. In 2015, an estimated 84,000 children 0–14 years old were living with HIV in Malawi. The Malawi Population-based HIV Impact Assessment (MPHIA), a national household survey conducted from 2015 to 2016, provides the first direct measurement of national HIV prevalence and viral suppression (VS) prevalence among children 0–14 years.

**Methods**: MPHIA tested children in every other surveyed household (*n* = 13,234) using the national HIV rapid test (RT) algorithm consisting of Determine™ (screening RT) and UniGold™ (confirmatory RT). Children >18 months who tested positive by both RTs were confirmed by laboratory-based testing using Geenius™ HIV 1/2 Confirmatory Assay (Bio-Rad). Children ≤18 months screening reactive on Determine underwent DNA PCR testing for HIV diagnosis. HIV RNA viral load suppression was defined as <1000 copies/ml. Weighted national paediatric HIV prevalence and VS prevalence were measured using SI-CHAID weights. Jackknife Replication method was used to calculate 95% confidence interval (CI). The number of children living with HIV was estimated using population projections from the Malawi National Statistics Office (NSO).

**Results**: Of 9952 eligible children, 6143 (61.7%) provided blood for HIV testing. Of those tested, 99 children were HIV positive; 2 were DNA PCR positive. Overall prevalence was 1.6% (95% CI: 1.2–2.0), equivalent to 122,721 children living with HIV (95% CI: 90,868–154,573). Among those infected, VS prevalence was 42.9% (95% CI: 30.5–55.4). HIV prevalence was greater in urban compared to rural areas; VS was considerably low among children<5 years and those residing in urban areas.

**Abstract TUAC0304–Table 1.** HIV prevalence and viral suppression in children.

Variable	Unweighted number of children who tested HIV positive/number tested	Prevalence (%)	95% CI	Weighted number of children living with HIV	95% CI	Number of children (NSO projection)	Viral suppression (%)	95% CI
0–4 years	20/1854	1.2	0.6–1.8	30,541	14,492–46,591	2,583,791	21.9	3.6–40.4
5–9 years	37/2197	1.6	0.9–2.3	49,271	27,076–71,466	3,063,666	49.1	31.2–67.0
10–14 years	42/2092	2.0	1.3–2.7	42,909	27,069–58,749	2,143,287	50.3	29.7–70.9
Urban residence	37/1911	2.3	1.4–3.1	26,826	13,621–40,032	1,181,940	28.9	12.2–45.5
Rural residence	62/6143	1.5	1.0–1.9	95,894	65,917–125,872	6,608,804	47.0	32.2–61.9

**Conclusions**: The MPHIA estimate of the number of children living with HIV is 46% greater than the current estimate which is currently being used for planning of paediatric HIV interventions. Low rates of viral suppression seen in children indicate the need for uptake of effective HIV treatment and adherence interventions to achieve the UNAIDS target of 73% viral suppression among children.

## TUAC0305

### Estimating HIV incidence and the undiagnosed HIV population in the European Union/European Economic Area

A van Sighem ^1^; A Pharris^2^; C Quinten^2^; T Noori^2^; AJ Amato-Gauci^2^; and the ECDC HIV/AIDS Surveillance and Dublin Declaration Networks

^1^Stichting HIV Monitoring, Amsterdam, The Netherlands. ^2^European Centre for Disease Prevention and Control (ECDC), Stockholm, Sweden

Presenting author email: a.i.vansighem@amc.uva.nl

**Background**: Each year, about 30,000 people are newly diagnosed with HIV in the 31 countries of the European Union/European Economic Area (EU/EEA). We aimed to estimate the number of people living with undiagnosed HIV in the entire EU/EEA and in four sub-regions.

**Methods**: Annual data on HIV diagnoses in 2003–2015 were retrieved from a database for HIV/AIDS within the European Surveillance System (TESSy). HIV diagnoses were adjusted for reporting delay and stratified by the presence of an AIDS-defining event within three months of HIV diagnosis and, for individuals without AIDS, by CD4 cell count (≥500, 350–499, 200–349, <200 cells/mm^3^) at the time of diagnosis. Countries were grouped in sub-regions as defined by United Nations. A back-calculation method based on the ECDC HIV Modelling Tool was used to estimate annual numbers of newly acquired HIV infections, the distribution of time between infection and diagnosis by calendar year and the number of people still undiagnosed by the end of 2015.

**Results**: In 2003–2015, there were 403,169 HIV diagnoses: 142,010 (35%) in western, 121,624 (30%) in northern, 27,662 (7%) in eastern and 111,873 (28%) in southern Europe. In the entire EU/EEA, 120,100 (95% confidence interval (CI): 113,000–127,800) people were estimated to be living with undiagnosed HIV by the end of 2015, of whom 47% had a CD4 count ≥500 cells/mm^3^ and 31% <350 cells/mm^3^, with 28,000 (95% CI: 24,700–31,700) new infections in 2015. The estimated number of undiagnosed HIV infections was highest in southern Europe, while infection rates were highest and time to diagnosis shortest in northern and western Europe (Table 1).

**Abstract TUAC0305–Table 1.** Undiagnosed population and infection rates.

Sub-region	Undiagnosed, total	Undiagnosed, CD4 ≥500		Undiagnosed, CD4 <350		Infection rate (/100,000 population)	Time to diagnosis (years)
Western	36,000 32,500–39,600	18,700 16,700–20,800	52%	9800 9200–10,700	27%	5.8 5.0–6.8	2.5 [1.2–4.6]
Northern	27,800 25,700–30,700	14,200 12,900–15,900	51%	7800 7300–8400	28%	9.4 8.5–10.8	2.3 [1.1–4.3]
Eastern	12,700 10,900–14,900	5800 4800–7100	46%	4100 3600–4700	32%	3.3 2.4–4.2	3.3 [1.6–6.0]
Southern	42,900 39,800–46,400	17,800 16,000–19,900	41%	15,600 14,900–16,500	36%	3.4 2.1–4.6	3.9 [1.9–7.0]
Total	120,100 113,300–127,800	56,600 52,400–61,000	47%	37,600 35,900–39,300	31%	5.4 4.8–6.2	2.9 [1.4–5.4]

Undiagnosed population and infection rate with 95% confidence intervals and median time to diagnosis (interquartile range) in 2015.

**Conclusions**: A substantial number of people in the EU/EEA are living with undiagnosed HIV. Although the estimated CD4 distribution suggests that approximately half of them are in an early stage of infection, a significant proportion are estimated to have late-stage infection, suggesting that more efforts are needed to test and diagnose these people.

## TUAC0401

### The effect of a conditional cash transfer for HIV prevention on the experience of partner violence for young women: evidence from a randomized experiment in South Africa HPTN 068

K Kilburn ^1^; A Pettifor^2,3^; J Edwards^2^; A Selin^2^; S Delong^2^; R Twine^3^; J Hughes^4^; J Wang^4^; X Gomez-Olive^3^; C Macphail^3,5^ and K Kahn^3^

^1^University of North Carolina at Chapel Hill, Global Health and Infectious Diseases, Chapel Hill, USA. ^2^University of North Carolina at Chapel Hill, Epidemiology, Chapel Hill, USA. ^3^University of the Witwatersrand, MRC/Wits Rural Public Health and Health Transitions Research Unit (Agincourt), Johannesburg, South Africa. ^4^Statistical Center for HIV/AIDS Research and Prevention (SCHARP), Seattle, USA. ^5^University of New England, Armindale, Australia

Presenting author email: kkilburn@unc.edu

**Background**: Evidence has shown that the experience of violence by a partner has important influences on women’s risk of HIV acquisition. Conditional cash transfers (CCTs) targeted to young women in sub-Saharan Africa have been advocated as an intervention to reduce the risk of HIV infection, but the success of such interventions may be conditional upon changes in gendered power inequalities. Using a randomized experiment in northeast South Africa, we find that a CCT targeted to poor girls in high school reduced the risk of intimate partner violence (IPV) by 34%. The purpose of this study is to understand the pathways through which the CCT affects IPV.

**Methods**: Our study is a phase 3, randomized controlled trial (HPTN 068) in a rural area in Mpumalanga province, South Africa. Eligible young women (aged 13–20) and their parents or guardians were randomly assigned (1:1) to receive a monthly cash transfer conditional on school attendance versus no cash transfer. Participants (*N* = 2448) were interviewed at baseline and then at annual follow-up visits at 12, 24 and 36 months. We estimate the primary outcome, physical IPV in the past 12 months, using a GEE log-binomial regression model. We examined mediation of direct effects through intermediate pathways using methods designed for nonlinear models under the counterfactual framework. Mediators include sexual behaviours, empowerment and economic well-being measures.

**Results**: We find evidence that the CCT works through delaying sexual debut or reducing the likelihood of having a sexual partner. The intervention interacts with these mediators leading to a larger reduction in IPV risk. Compared to the direct effect of the CCT on any physical IPV (relative risk (RR) 0.66, confidence interval (CI) (95%):0.59–0.74), the risk of IPV is further reduced when we set the controlled direct effect to either no sexual debut (RR 0.53, CI (95%):0.45–0.63) or to no sexual partners (RR 0.56, CI (95%):0.48–0.63).

**Conclusions**: Results indicate that a CCT for adolescent school girls has protective effects on girls’ experience of violence in part because the intervention reduces the likelihood of debut or having a sexual partner, thereby reducing the opportunity for IPV. Since these behaviours also protect against HIV acquisition, this evidence strengthens the case for CCTs for HIV prevention.

## TUAC0402

### The effect of school attendance and school dropout on incident HIV and HSV-2 among young women in rural South Africa enrolled in HPTN 068

MCD Stoner ^1^; JK Edwards^1^; WC Miller^1,2^; AE Aiello^1^; CT Halpern^1^; A Julien^1^; A Selin^1^; R Twine^3^; J Hughes^4,5^; J Wang^5^; FX Gomez-Olive^3^; RW Wagner^3^; C Macphail^6^; O Laeyendecker^7^; Y Agyei^7^; SM Tollman^3,8^; K Kahn^3,8^; A Pettifor^1^; HPTN 068

^1^University of North Carolina at Chapel Hill, Chapel Hill, USA. ^2^Ohio University, Athens, USA. ^3^MRC/Wits Rural Public Health and Health Transitions Unit CRS, Agincourt, South Africa. ^4^University of Washington, Seattle, USA. ^5^Fred Hutchinson Cancer Research Center, Seattle, USA. ^6^University of New England, Biddeford, USA. ^7^Johns Hopkins Bloomberg School of Public Health, Baltimore, USA. ^8^University of Witwatersrand School of Public Health, Johannesburg, South Africa

Presenting author email: stonerm@email.unc.edu

**Background**: Education may protect against sexually transmitted infections but has primarily been studied as educational attainment in adults or using measures of prevalent rather than incident infection. Few studies have explored schooling as a measure of time spent in a structured school environment. We hypothesize that low versus high attendance in school and school dropout versus staying in school are associated with a higher risk of incident HIV and HSV-2 infection among young women

**Methods**: We used longitudinal data from the HPTN 068 randomized trial in Agincourt, South Africa, to determine if percentage of school days attended between annual surveys and school dropout affect incident HIV and HSV-2 in young women aged 13–23. We examined inverse probability of exposure-weighted survival curves and used them to calculate one-, two- and three-year risk differences and risk ratios for the effect of school attendance on incident HIV and HSV-2, accounting for confounding. A marginal structural cox model was then used to estimate the hazard ratios for the effect of school attendance and school dropout on incident HIV and HSV-2.

**Results**: Over the study period, 107 incident HIV cases occurred among the 2328 women without HIV at baseline and 208 HSV-2 incident cases occurred among the 2238 women without prevalent HSV-2 at baseline. Risk of HIV and HSV-2 increased over time and was lower for young women who had high attendance (≥80% school days) versus low attendance (<80%) at all time points. After accounting for relevant confounders, young women with low attendance were more likely to develop HIV (hazard ratio (HR): 2.97; 95% confidence interval (CI): 1.62, 5.45) and HSV-2 (HR: 2.47; 95% CI: 1.46, 4.17) over the follow-up period than young women with high attendance. Similarly, young women who dropped out of school had a higher weighted hazard of both HIV (HR: 3.25 95% CI: 1.67, 6.32) and HSV-2 (HR: 2.70; 95% CI 1.59, 4.59).

**Conclusions**: Young women who attend more school and stay in school have a lower risk of incident HIV and HSV-2 infection. Interventions to prevent infections should continue to encourage young women to attend school more frequently and to avoid dropouts.

## TUAC0403

### Increasing HIV test uptake and case finding through assisted HIV partner notification services: a systematic review and meta-analysis

S Dalal ^1^; C Johnson^1^; V Fonner^2^; C Kennedy^3^; N Siegfried^4^; C Figueroa^1^ and R Baggaley^1^

^1^World Health Organization, HIV/AIDS, Geneva, Switzerland. ^2^Medical University of South Carolina, Charleston, USA. ^3^Johns Hopkins University Bloomberg School of Public Health, Baltimore, USA. ^4^Independent Clinical Epidemiologist, Cape Town, South Africa

Presenting author email: dalals@who.int

**Background**: Despite the expansion of HIV testing services (HTS), an estimated 40% of people with HIV infection remain undiagnosed. New approaches are needed to enhance HTS efficiency in order to reach the first UN 90-90-90 goal to diagnose 90% of people with HIV infection by 2020. We conducted a systematic review on the effectiveness of assisted partner notification in improving HIV test uptake and diagnosis, and the occurrence of adverse events, to inform the development of WHO normative guidelines.

**Methods**: We systematically searched five electronic databases through June 2016. We also contacted experts in the field and authors for additional information where needed. Eligible studies compared assisted HIV partner notification services to passive or no notification and measured one of the following outcomes: HIV test uptake, proportion of partners tested and diagnosed HIV positive, CD4 or viral load, linkage to clinical assessment or antiretroviral therapy, linkage to prevention for HIV-negative partners and any social harm among HIV-positive patients or their partners. We used the Cochrane Collaboration’s tool to assess risk of bias and GRADE to evaluate the quality of the evidence. Where appropriate, random-effects meta-analysis was conducted.

**Results**: Of 1742 citations identified, four randomized controlled trials (RCTs) and six observational studies totalling 5150 index patients from eight countries were included. Meta-analysis of three individually randomized trials provided moderate-quality evidence that assisted partner notification services increased HTS uptake among partners 1.5 times compared to passive referral (relative risk [RR] = 1.46; 95% confidence interval (CI): 1.22–1.75; *I* ^2^ = 0%). Overall, studies reported that 13–72% of partners were HIV positive, and between 12% and 66% of partners were newly diagnosed. The proportion of HIV-positive partners was 1.5 times higher with assisted partner notification than with passive referral (RR = 1.47; 95% CI: 1.12–1.92; *I* ^2^ = 0%; moderate-quality evidence). Few instances of violence or harm were reported.

**Conclusions**: Assisted partner notification improved partner testing and increased diagnosis of people with HIV infection, with few reports of harm. The WHO strongly recommends offering voluntary-assisted HIV partner notification services as part of a comprehensive package of testing to all newly diagnosed persons and periodically to all HIV-positive persons throughout their care and treatment.

## TUAC0404

### Association between HIV and sexually transmitted infections and partner circumcision among women in uMgungundlovu District, South Africa: a cross-sectional analysis of HIPSS baseline data

S Davis^1^; C Toledo ^2^; L Lewis^3^; C Cawood^3^; A Bere^4^; M Glenshaw^4^ and A Kharsany^3^

^1^US Centers for Disease Control, HIV Prevention Branch, Atlanta, USA. ^2^US Centers for Disease Control and Prevention, Atlanta, USA. ^3^Center for the AIDS Program of Research in South Africa, Durban, South Africa. ^4^US Centers for Disease Control and Prevention, Durban, South Africa

Presenting author email: cot8@cdc.gov

**Background**: Randomized controlled trials and observational data have demonstrated that circumcision partially protects men from acquiring HIV and some sexually transmitted infections (STIs) through heterosexual sex. They also suggest that female partners receive some protection, possibly indirectly through lower infection prevalences among men. However, population-level data outside experimental settings are lacking. The HIV Incidence Provincial Surveillance System (HIPSS) is a longitudinal study in Vulindlela and Greater Edendale sub-districts, South Africa, which collected population-level baseline data in 2014 and 2015.

**Methods**: Female HIPPS participants were aged 15–49 years. Those with at least one past or current male sexual partner who were able to report his circumcision status were analysed. Participants were assessed for HIV status via double fourth-generation ELISA testing with confirmatory Western blot; *N. gonorrhoeae*, *C. trachomatis*, *T*. *vaginalis* and human papillomavirus infection with standard testing of self-collected vulvovaginal swabs; *T. pallidum*, HSV-2 and hepatitis B with serology; and STI diagnosis history and current STI symptoms with self-report. They were grouped by circumcision status of their most recent partner, stratified into age below or at least equal to 25 years and compared on presence of STI outcomes by chi-square testing, weighted for selection probability and nonresponse.

**Results**: A total of 4766 women were included. Women with circumcised partners had similar numbers of lifetime partners (mean 2.45) to those with uncircumcised partners (mean 2.71). In the younger stratum, partner circumcision was negatively associated with HIV (24% vs. 35%, *p* < 0.01) and HSV-2 (49% vs. 62%, *p* < 0.01). In the older stratum, partner circumcision was negatively associated with syphilis (1.5% vs. 3.4%, *p* = 0.04) and HSV-2 (83% vs. 86%, *p* = 0.04), but was associated with ever having had an STI (11% vs. 7%, *p* < 0.01).

**Conclusions**: Partner circumcision was associated with decreased prevalence of HSV-2 in all female HIPSS participants, decreased prevalence of HIV in younger women and decreased prevalence of syphilis in older women. Its positive association with self-reported STI history in older participants may derive from differential ascertainment; circumcision typically involves STI screening in men, potentially leading to partner notification. Findings support community-level protection against HIV and some other STIs among women from male circumcision.

## TUAC0405

### Effects of syringe distribution policy change at a syringe services programme in Baltimore, MD: a forecast analysis

S Allen ^1^; J Park^2^; B Weir^1^; D Holtgrave^1^ and S Sherman^1^

^1^Johns Hopkins University, Health, Behavior and Society, Baltimore, USA. ^2^Johns Hopkins University, Epidemiology, Baltimore, USA

Presenting author email: sean.travis.allen@gmail.com

**Background**: Syringe services programmes (SSPs) are associated with decreases in prevalence and incidence rates of blood-borne diseases (e.g. HIV and hepatitis C virus [HCV] infections), soft-tissue infections and other morbidities among people who inject drugs (PWID). The core goal of SSPs is to decrease the circulation time of contaminated syringes and to increase the volume of new syringes, effectively increasing the “coverage” of sterile needles and syringes for every injection. In 2014, the Baltimore City SSP shifted from a strict one-to-one syringe exchange policy to a needs-based distribution policy whereby PWID could receive as many syringes as they require. The purpose of this research is to examine the impact of this policy change on syringe distribution among PWID.

**Methods**: Syringe distribution data from April 2012 to November 2016 were abstracted from the Baltimore City SSP and divided into monthly observations. These data were used to build an ARIMA model that forecast the estimated number of syringes that would have been distributed had the syringe distribution policy not changed in the 26-month period following the policy change.

**Results**: There were significant (*p* < 0.05) differences in the mean number of syringes distributed per month in the pre- and post-policy change periods (44,410 and 96,187, respectively). During the post-policy change period, we forecast that 1,786,174 syringes would be distributed. In actuality, 2,500,857 syringes were distributed during this period. Changing to a needs-based syringe distribution policy resulted in the distribution of an additional 714,683 syringes.

**Conclusions**: Needs-based syringe distribution leads to greater circulation of sterile syringes and may prevent new HIV/HCV infections.

## TUAC0501

### Are migrants acquiring HIV before or after migration to Spain? Results from the aMASE study

D Alvarez-del Arco^1,2,3^; J del Romero^4^; F Pulido^5^; M Velasco Arribas^6^; F Dronda^7^; JR Blanco^8^; P Garcia de Olalla^9^; I Ocaña^10^; J Belda Ibáñez^11^; MJ Barbera^12^; S Cuéllar^13^; J Iribarren^14^; A López-Lirola^15^; M Masiá^16^; E Fernández^17^; G Mateu^18^; A Peña^19^; P Ndumbi^1^; J del Amo ^1^; aMASE Study Group

^1^Instituto de Salud Carlos III, National Centre for Epidemiology, Madrid, Spain. ^2^Universidad Complutense de Madrid, Madrid, Spain. ^3^CIBER de Epidemiologia y Salud Publica, Barcelona, Spain. ^4^Centro Sanitario Sandoval, Madrid, Spain. ^5^Hospital Universitario Doce de Octubre, Madrid, Spain. ^6^Hospital Universitario Fundación Alcorcón, Madrid, Spain. ^7^Hospital Ramón y Cajal, Madrid, Spain. ^8^Hospital San Pedro de La Rioja, Logroño, Spain. ^9^Agencia de Salud Pública de Barcelona, Barcelona, Spain. ^10^Hospital de la Vall d’Hebron, Barcelona, Spain. ^11^CIPS Alicante, Alicante, Spain. ^12^Unidad de infecciones de transmisión sexual de Drassanes-Hospital de la Vall d’Hebron de Barcelona, Barcelona, Spain. ^13^Hospital La Fe, Valencia, Spain. ^14^Hospital Donosti, Donosti, Spain. ^15^Hospital de Poniente, Almeria, Spain. ^16^Hospital de Elche, Elche, Spain. ^17^Hospital Clínic, Barcelona, Spain. ^18^Hospital de Sant Pau, Barcelona, Spain. ^19^Complejo Hospitalario Universitario Granada, Hospital del Campus de la Salud, Granada, Spain

Presenting author email: jdamo@isciii.es

**Background**: To know if HIV acquisition among migrants living in Spain takes place before or after migration to the country.

**Methods**: Cross-sectional study in 18 centres/hospitals following patients with HIV. We selected patients diagnosed with HIV in the last five years, aged 18 and more, born outside Spain and able to complete the survey in one of the 15 available languages. Two questionnaires were used: one self-completed by participants in a computer/tablet and another about patients’ clinical data. We collected epidemiological, socio-economic, clinical, behavioural, migratory trajectory, previous HIV serology, CD4 determinations, viral load and viral subtype data. To estimate timing of HIV acquisition, Bayesian methods with longitudinal measurements of CD4 and viral load were used, and estimates were made applying mixed linear models based on HIV natural history (data from the European collaboration of seroconverters CASCADE).

**Results**: Of 710 participants, 685 (97%) had data to estimate time of HIV acquisition (TA). Participants with information on TA had a median of nine years [RI: 6–13] of residence in Spain, were mainly male (77%) and were from Latin America and the Caribbean (LAC) (64%), Other European Countries (OEC) (17%) and sub-Saharan Africa (SSA) (13%). Most common route of transmission was the relationship between men who have sex with men (MSM) (60%), followed by heterosexual transmission (34%); injecting drug users (IDUs) were minority (3%) and in 3% were unknown. Seventy-six per cent of patients acquired HIV after migrating to Spain. HIV post-migration acquisition was higher among MSM (85%) than in heterosexuals (67%) and in people from LAC (77%) and OEC (75%) compared to those from SSA (62%). HIV was acquired after migrating in greater proportions among those aged more than 50 years (82%), with secondary or university studies (77%) and with legal residency status (79%).

**Conclusions**: A significant proportion of migrants living with HIV in Spain acquired HIV after migration. The proportion of post-migration HIV acquisition is higher among MSM and in people from LAC and OEC. These results show a failure in preventive interventions in specific migrants’ groups and emphasize the need to design interventions taking into account migrants’ heterogeneity.

## TUAC0502

### Prevalence, trends and risk factors of transactional sex among men who have sex with men in Metro Vancouver, Canada: a longitudinal event-level analysis

NJ Lachowsky ^1,2,3^; L Wang^1^; J Zhu^1^; H Armstrong^1,4^; M Taylor^5^; G Olarewaju^1^; R Hogg^1,6^; EA Roth^7^; DM Moore^1,4^; Momentum Health Study

^1^British Columbia Centre for Excellence in HIV/AIDS, Division of Epidemiology & Population Health, Vancouver, Canada. ^2^University of Victoria, School of Public Health and Social Policy, Victoria, Canada. ^3^Centre for Addictions Research BC, Victoria, Canada. ^4^University of British Columbia, Faculty of Medicine, Vancouver, Canada. ^5^Health Initiative for Men, Vancouver, Canada. ^6^Simon Fraser University, Faculty of Health Sciences, Burnaby, Canada. ^7^Department of Anthropology, University of Victoria, Victoria, Canada

Presenting author email: nlachowsky@cfenet.ubc.ca

**Background**: Debate continues with respect to whether transactional sex among men who have sex with men (MSM) constitutes increased risk of HIV transmission. We sought to identify factors associated with transactional sex using event-level data from a population-based longitudinal behavioural cohort of MSM in Metro Vancouver, Canada.

**Methods**: Sexually active MSM aged ≥16 years were recruited using respondent-driven sampling (RDS), and from February 2012 to February 2016, participants completed study visits every six months, which included a computer-assisted self-interview. At each visit, participants provided event-level data by reporting on their last sexual encounter with their five most recent partners (e.g. participant and partner substance use, partner’s relative age). We used a four-level mixed-effects model (RDS recruitment chain; participant; visit; and event) to evaluate temporal trends and factors associated with transactional sex. We built a multivariable model to compare events where money, drugs or goods was received for sex to events with no transaction reported using backward selection with minimization of QIC and type III *p*-values.

**Results**: Of 690 participants, 8990 sexual events were reported across 2792 study visits (median follow-up time of 3.62 years). Although 11.7% of participants reported any transactional sex, event-level reports were rare: 2.4% of events included receiving money/drugs/goods, 1.5% of events included providing money/drugs/goods and 96.0% of events did not include transactional sex. Transactional sex decreased over time (3.8% to 1.0% from first to eighth visit: odds ratio [OR] = 0.82, 95% confidence interval [CI]:0.73–0.92). After controlling for significant individual-level factors, the following event-level factors were associated with greater odds of transactional sex: having met online (adjusted OR [aOR] = 3.32, 95% CI:1.32–8.37), having a shorter relationship (aOR = 0.99 per month, 95% CI:0.99–1.00), having an older partner (aOR = 2.42, 95% CI:1.02–5.75) and having a partner who used crystal methamphetamine (aOR = 3.85, 95% CI:1.12–13.17) or used gamma-hydroxybutyrate (aOR = 6.79, 95% CI:2.19–21.01). Across all events, sexual HIV risk was not associated with transactional versus non-transactional sex: 23% versus 24% reported condomless anal sex with a sero-concordant partner (*p* = 0.212) and 17% versus 12% reported event-level condomless anal sex with a sero-discordant/unknown status partner (*p* = 0.946).

**Conclusions**: Transactional sex events were rarely reported. Partner substance use was strongly associated with transactional sex, but we found no significant associations with HIV risk behaviour.

## TUAC0503

### Is on-demand HIV pre-exposure prophylaxis (PrEP) a suitable tool for men who have sex with men (MSM) who participate in chemsex? Results from a sub-study of the ANRS-IPERGAY trial

P Roux ^1^; L Fressard^1^; M Suzan-Monti^1^; J Chas^2^; C Capitant^3^; L Meyer^3^; C Tremblay^4^; J-M Molina^5^; G Pialoux^2^; B Spire^1^; ANRS IPERGAY Study Group

^1^INSERM, INSERM U912 - SESSTIM, Marseille, France. ^2^Department of Infectious Diseases, Hôpital Tenon-APHP, Paris, France. ^3^INSERM, Inserm SC10 US019, Villejuif, France. ^4^Centre Hospitalier de l’Université de Montréal, Laboratoire de santé publique du Québec, Montréal, Canada. ^5^University, Paris Diderot, Paris, France

Presenting author email: perrine.roux@inserm.fr

**Background**: Chemsex - the use of psychoactive substances during sexual encounters - is a growing concern among men who have sex with men (MSM). It includes the even riskier practice of “slamming”, which consists in injecting a substance before having sex. On-demand HIV pre-exposure prophylaxis (PrEP) may be a suitable tool to prevent HIV transmission in “chemsexers” (i.e. those practicing chemsex). We used a sub-study of the ANRS-IPERGAY trial to describe chemsexers and their PrEP use.

**Methods**: Among the 361 MSM (3051 visits) enrolled in ANRS-IPERGAY’s open-label extension study, we selected the 331 (1657 visits) who reported drug use during at least one sexual encounter. A two-monthly web questionnaire over 12 months collected socio-behavioural data including the use of PrEP and practicing chemsex. We compared sexual behaviours between visits where chemsex was reported and those where it was not. A GEE logistic regression was used to study whether practicing chemsex was associated with PrEP use.

**Results**: Among the 331 participants, during follow-up, 29% reported chemsex while 8% reported slamming at least once. Chemsex was reported in 16% of all visits (12% and 4%, respectively, with one and multiple partners) mainly involving the use of GHB/GBL (51%) and synthetic cathinones (46%). Chemsexers were not significantly different from non-chemsexers regarding sociodemographic characteristics, although they reported higher use of anxiolytics during the previous 12 months (*p* < 10^–2^). When MSM reported chemsex during their most recent sexual encounter, it was associated with a greater likelihood of receptive anal sex (*p* < 10^–3^), hardcore sexual practices (*p* < 10^–3^), casual partner(s) (*p* < 10^–3^) and a higher perception of risk (*p* < 10^–3^). Those who reported chemsex at their most recent encounter were more likely to use PrEP than those who did not (*p* < 10^–3^) and less likely to use condoms (*p* < 10^–3^). After adjustment for other potential correlates, chemsex remained associated with PrEP use (odds ratio [95% confidence interval] = 2.18 [1.04; 4.59]).

**Conclusions**: Our findings show that chemsexers are more likely to report high-risk sexual practices but also have a higher perception of risk. Consequently, they are also more likely to use PrEP when practicing chemsex; PrEP may be a suitable tool to reduce HIV risk transmission among chemsexers.

## TUAC0504

### Assessing HIV prevalence and health outcomes of children of female sex workers in Port Elizabeth, South Africa, to guide PMTCT programming for vulnerable populations

S Schwartz ^1^; Z Kose^2^; M Mcingana^3^; A Rao^1^; A Lambert^4^; V Srivatsan^1^; N Phaswana-Mafuya^2^; H Hausler^4^ and S Baral^1^

^1^Johns Hopkins University, Epidemiology, Baltimore, USA. ^2^Human Sciences Research Council, Port Elizabeth, South Africa. ^3^TB/HIV Care Association, Port Elizabeth, South Africa. ^4^TB/HIV Care Association, Cape Town, South Africa

Presenting author email: sschwartz@jhu.edu

**Background**: Female sex workers (FSW) are disproportionately affected by HIV. Although the majority of FSW are mothers, little is known about the health outcomes, including HIV prevalence and programmatic needs, among their children.

**Methods**: FSW in Port Elizabeth, South Africa, were recruited at mobile clinics and within the community to bring their children ≤12 years to the study site. A cross-sectional interview with the mother, followed by health assessments for the mother and her children, was completed. HIV testing was completed for mothers and children; children were tested using rapid antibody tests (≥18 months) or polymerase chain reaction (<18 months). HIV outcomes and health status of mothers and children are characterized. Stunting and wasting were estimated using WHO 2006 Child Growth Standards.

**Results**: From July 2015 to February 2016, 114 mothers and 200 children were enrolled. Overall, 77/114 (68%) mothers were living with HIV, of which 53% (41/77) were on antiretroviral therapy. On average, FSW continued sex work for a median of five months during their last pregnancy (interquartile range (IQR) 4–7). The median age of children attending was 6 years (IQR 3–9). The majority (73%, *n* = 145/200) of children were breastfed; the median and mean duration of breastfeeding were 6 (IQR 3–24) and 12 months (SD 12), respectively. Just over half (108/200) of children had ever been tested for HIV, including 95/133 (71%) of children with HIV-positive mothers. HIV prevalence among children was 3% (5% among those with HIV-positive mothers, *n* = 6/133). Seventy-three per cent of children were reported as up-to-date on their vaccinations. Among the 79 children under 5, 29% were stunted according to height-for-age and 13% wasted per weight-for-age.

**Conclusions**: The majority of FSW mothers were HIV positive, and many were not on treatment. HIV prevalence among children was higher than the national average among children, and nearly one-third of children with HIV-positive mothers had never received HIV testing. Aside from children’s HIV risks, substantial chronic nutritional deficiencies were identified through height-for-age, alongside acute nutritional deficiencies (weight-for-age) and immunization gaps. Programmes for FSW should address vertical transmission risks including treatment support during pregnancy and breastfeeding and consider catch-up HIV testing and vaccination campaigns to promote children’s health.

## TUAC0505

### Low HIV incidence but high HCV incidence among people who inject drugs in Haiphong, Vietnam: results of the ANRS 12299/NIDA P30DA011041 DRIVE-IN study

MK Pham ^1^; JP Moles^2^; H Duong Thi^1^; T Nguyen Thi^3^; G Hoang Thi^4^; TT Nham Thi^5^; V Vu Hai^6^; HO Khuat Thi^5^; R Vallo^2^; M Peries^2^; K Arasteh^7^; C Quillet^2^; J Feelemyer^7^; L Michel^8^; T Hammett^9^; N Nagot^2^; D Des Jarlais^7^; D Laureillard^2,10^; DRIVE study group

^1^Haiphong University of Medicine and Pharmacy, Faculty of Public Health, Haiphong, Vietnam. ^2^University of Montpellier, Inserm U1058, Etablissement Français du Sang, Montpellier, France. ^3^Haiphong Provincial AIDS Center, Haiphong, Vietnam. ^4^Haiphong University of Medicine and Pharmacy, Haiphong, Vietnam. ^5^Supporting Community Development Initiatives, Hanoi, Vietnam. ^6^Dept of Infectious and tropical diseases, Viet Tiep Hospital, Haiphong, Vietnam. ^7^Icahn School of Medicine at Mount Sinai, New York, USA. ^8^CESP/Inserm U1018, Pierre Nicole Centre, French Red Cross, Paris, France. ^9^Abt Associates, MA, USA. ^10^Infectious Diseases Department, Caremeau University Hospital, Nimes, France

Presenting author email: khuepm@gmail.com

**Background**: In Vietnam, HIV control programmes targeting persons who inject drugs (PWID), including harm reduction and scaled-up ART, have been implemented for about 10 years. Although HIV prevalence is declining in this group, the impact of this programme on the rate of HIV new infections, but also on HCV transmission, remains unknown.

**Methods**: We carried out a community-based respondent-driven sampling (RDS) survey among ‘active PWID’ (i.e. with positive urine test for heroin and presence of injection marks) in Haiphong, with HIV and HCV testing. Then, HIV-negative participants and HCV-negative participants not on methadone maintenance therapy (MMT) were eligible for one-year follow-up. HIV/HCV was tested at six months and one year along with routine harm reduction activities from community-based organizations (CBO) and support to access MMT. We estimated HIV and HCV incidence and risk factors associated with HCV seroconversion.

**Results**: Among 603 RDS participants, 90% were males, and their median age was 36.5 years. HIV prevalence was 25% (95% confidence interval (CI): 22–29) and HCV prevalence was 66% (95% CI: 63–70). A total of 204 RDS participants were enrolled in the cohort, including 94 HIV negative/HCV negative, 5 HIV positive /HCV negative, 105 HIV negative /HCV positive. The cohort participants were mainly males (90%) with a median age of 37 years [IQR 30–44] and a median number of injections over the last month of 90 [IQR 60–90]. The retention rate at 63 weeks was 78%. No participant seroconverted for HIV during the 206.0 person-years of follow-up (HIV incidence unilateral CI: 0–1.8/100 persons-years). Eighteen participants seroconverted for HCV, mainly during the first six months (13/18), for a HCV incidence of 18.8/100 person-years [95% CI; 11.2–29.8]. In multivariable analyses, only injecting more than 75 times per month vs. injecting less than 75 times per month (odds ratio: 8.7 [95% CI; 2.1–41.4]) was associated with HCV seroconversion.

**Conclusions**: In Haiphong, harm reduction activities and high levels of antiretroviral treatment likely contributed to the reduction of HIV epidemic among PWID. However, HCV incidence is still unacceptably high. Current harm reduction activities cannot control the HCV epidemic; additional strategies such as universal ART and large-scale HCV treatment should be urgently evaluated to end the HIV epidemic and tackle HCV transmission among PWID in Vietnam.

## TUAD0101

### Wide-ranging real-world impacts of a policy change on treatment eligibility on ART initiation and retention in care in Zambia

A Mody ^1^; A Zanolini^2^; K Sikombe^2^; P Somwe^2^; I Sikazwe^2^; C Bolton^2^; C Holmes^2,3^; N Padian^4^ and E Geng^1^

^1^University of California, San Francisco, Division of HIV, ID, and Global Medicine, San Francisco, USA. ^2^Centre for Infectious Disease Research in Zambia, Lusaka, Zambia. ^3^Johns Hopkins University, Division of Infectious Diseases, Baltimore, USA. ^4^University of California, Berkeley, School of Public Health, Berkeley, USA

Presenting author email: aaloke.mody@ucsf.edu

**Background**: Incomplete uptake as well as unforeseen consequences often accompanies changes in public health guidance. In April 2014, Zambia increased the CD4 threshold for HIV treatment from 350 to 500 cells/µl. We evaluated the effect of this guideline change on ART uptake and retention in patients both targeted and not targeted by this change to evaluate for unintended consequences using a regression discontinuity design.

**Methods**: We analysed non-pregnant ART-naive patients in Zambia who newly enrolled within six months of the guideline change (1 September 2013 to 1 November 2014), excluding patients enrolled within 30 days of the implementation date to account for imprecise rollout. We utilized a quasi-experimental regression discontinuity design with local linear regression to estimate the effects of this policy change on ART initiation within three months of enrolment and retention in care at six months (defined as clinic attendance between three and nine months after enrolment) in all new enrolees, stratifying by enrolment treatment eligibility.

**Results**: A total of 20,513 patients (53.1% female, median age 34 years (interquartile range (IQR) 28–41)) were eligible for our analysis. Newly eligible patients (CD4 350–500, 15.5% of patients) saw a significant increase in ART initiation within three months (risk difference [RD] +35.3%, 95% CI 28.1–42.4, *p* < 0.001) and retention at six months (RD +7.4%, 95% CI 0.5–14.3, *p* = 0.034) with the policy change. Additionally, never-eligible patients (CD4 >500, 17.0% of patients) also saw an increase in ART initiation with the guideline change (RD +16.3%, 95% CI 9.8–22.7, *p* < 0.001), though retention was unaffected (*p* = 0.203). Among always-eligible patients (CD4 ≤350 or WHO stage≥3, 67.6% of patients), ART initiation at three months (*p* = 0.955) and retention at six months (*p* = 0.600) were unaffected.

**Conclusions**: Policy increasing CD4 threshold for treatment eligibility led to rapid changes in ART initiation practices as well as enhanced retention in the group targeted by the guidelines. ART initiation also improved amongst treatment-ineligible patients with the policy change, a positive spillover effect perhaps due to expansion of ART supply, while initiation and retention among those always eligible was not compromised. Real-world implementation of evidence-based practice often has broader impacts than those directly targeted that should be routinely evaluated to guide policy interventions.

## TUAD0102

### Pre-ART peak and plateau: early lessons from Zimbabwe on operational impact of “pre-ART mop-up” on ART initiation rates under Treat All

K Webb ^1^; V Chitiyo^1^; P Nesara^1^; S Page-Mtongwiza^1^; J Murungu^2^; T Maphosa^1^; P Mbetu^1^ and B Engelsmann^1^

^1^Organisation for Public Health Interventions and Development, Harare, Zimbabwe. ^2^Ministry of Health and Child Care, AIDS & TB, Harare, Zimbabwe

Presenting author email: kwebb@ophid.co.zw

**Background**: The WHO 2015 test and treat guidelines recommend ART initiation for all people living with HIV regardless of CD4 count or WHO clinical staging. Implementation of Treat All will require identification and return of pre-ART patients previously clinically ineligible for ART into care for timely initiation. Little is known about the feasibility and timelines required to conduct pre-ART mop-up in resource-limited settings. Our objective was to establish the proportion of clients initiated on ART from pre-ART mop-up following start of Treat All in 92 public health facilities in seven districts of Zimbabwe.

**Methods**: We purposively selected 92 health facilities implementing Treat All learning phase. We analysed routinely reported data from April–December 2016, comparing proportion of new ART initiations to patients newly diagnosed as HIV positive using chi-square testing. A facility-based survey of pre-ART register data and healthcare worker (HCW) perceptions and experiences of pre-ART mop-up during initial stages of Treat All was conducted to identify key operational themes.

**Results**: Over the period of interest, 9875 clients newly initiated on ART. The proportion of new ART initiations vs. newly diagnosed peaked at 160% after start of Treat All and plateaued back to below 100% of new diagnoses after six months, rates significantly higher than before Treat All (69.4% vs. 91.9%, *p* < 0.0001).

**Abstract TUAD0102–Figure 1.**
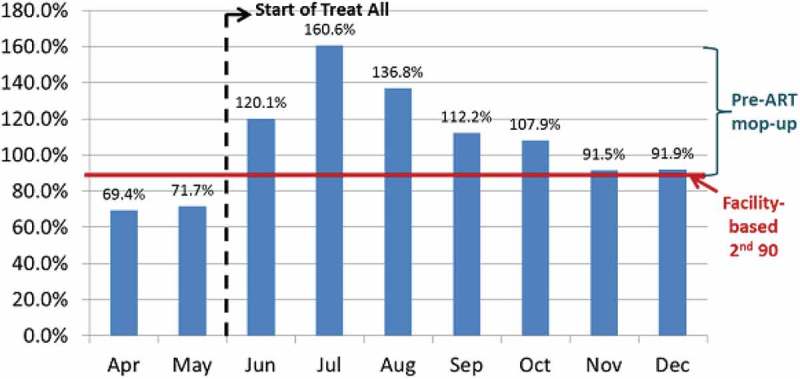
Proportion of ART initiation from new HIV diagnosed

Additional cell phones, air time and staff for identifying and contacting pre-ART patients were crucial for tracing large numbers of pre-ART clients at start of Treat All.

**Conclusions**: We demonstrate success of Treat All at returning previously ineligible HIV-positive clients for ART initiation in a resource-limited, high-burden setting. Provision of additional resources to support pre-ART mop-up phase was required and recommended for replication as Treat All scales-up. Following return to care of traceable pre-ART clients, ART initiations stabilize to rates above 90%, indicating the value of Treat All for progress towards the 2nd 90.

## TUAD0103

### Evaluating the feasibility of implementing UNAIDS’ 90-90-90 strategy, achieving universal access to treatment and eliminating HIV in Malawi

S Blower and L Palk

UCLA Medical School, Semel Institute, Santa Monica, USA

Presenting author email: sblower@mednet.ucla.edu

**Background**: Malawi has a severe HIV epidemic: prevalence in the general population is approximately 11%. The majority of the population of approximately 16 million live in rural communities. We estimate the total number of HIV-infected individuals (both diagnosed and undiagnosed) in Malawi and determine their geographic location. We use these results to evaluate the feasibility of implementing UNAIDS’90-90-90 strategy and of achieving universal access to treatment.

**Methods**: We constructed an epidemic surface prevalence (ESP) map using geo-referenced HIV-testing data from approximately 14,000 individuals (15–49 years old) who participated in a nationally representative population-level survey: the 2010 Malawi Demographic and Health Survey. We constructed a density of infection (DoI) map by combining the ESP map with gridded demographic data from the WorldPop database and a census-based age-structure map. The DoI map shows the estimated number of HIV-infected individuals (15–49 years old) in each square kilometre in Malawi, diagnosed and undiagnosed individuals. We calculated the total number of HIV-infected individuals by aggregating the mapped estimates.

**Results**: The ESP map shows that prevalence (in 15–49 year olds) varies from approximately 1% to 25%, and there is a strong north–south trend in increasing prevalence and a substantial urban–rural difference. Prevalence is highest in cities in the south (Blantyre and Zomba), Lilongwe in the central region, Mzuzu in the north and villages along Lake Malawi. The DoI map reveals the geographic dispersion pattern of all infected individuals. DoI ranges from one infected individual/km^2^ in rural areas to more than 1000 infected individuals/km^2^ in the four major urban centres. The map shows substantial regional differences in the DoI; our results show that these reflect differences in settlement patterns, population density and prevalence. We estimate that there were approximately 692,000 HIV-infected individuals (15–49 years old) in 2010 in Malawi, and only approximately 25% were living in urban areas.

**Conclusions**: Our results show the vast majority of HIV-infected individuals in Malawi live in rural areas in small communities where the DoI is low. This indicates that implementing the 90-90-90 strategy and achieving universal access to treatment may not be feasible. We recommend that HIV elimination strategies for resource-constrained countries are designed based on DoI and not on prevalence.

## TUAD0104

### Generic treatments for HIV, HBV, HCV and TB could be mass produced for <$90 per patient

A Hill^1^; M Barber^2^; D Gotham ^3^; J Fortunak^4^ and A Pozniak^5^

^1^University of Liverpool, Liverpool, UK. ^2^London School of Hygiene and Tropical Medicine, London, UK. ^3^Imperial College London, London, UK. ^4^Howard University, Washington, USA. ^5^Chelsea & Westminster Hospital, London, UK

Presenting author email: dzintarsgotham@googlemail.com

**Background**: High prices to treat HIV, viral hepatitis and TB can limit treatment access. This analysis aimed to determine prices currently feasible for HIV, HBV, HCV and first-line (1L) DS-TB treatment, assuming competitive generic manufacture.

**Methods**: Data on API exported from India were collected from an online database (www.infodriveindia.com) for July 2014–July 2016. Linear regression was used to plot API cost/kg versus export date, weighted by export volumes: the generated model was used to calculate current average cost/kg of API. Target prices were calculated based on the per-pill cost of API, plus costs of manufacture ($0.01/pill), 10% profit margin and assumed 27% tax on profit. Current lowest global prices are from public reports and the Global Drugs Fund (TB) and US prices from the Centers for Medicare and Medicaid Services. Patent protection expiry dates are from FDA Orange Book and Medicines Patent Pool Patent Status Database.

**Results**: Table 1 shows current prices of antiretrovirals for HIV, entecavir (ETV) for HBV (per person-year), HCV treatments (per 12-week course) and 1L DS-TB treatment (RHZE, per six-month course). API costs/kg were $1189 for ATV, $182 for TDF, $241 for 3TC, $109 for EFV, $380,965 for ETV, $1224 for SOF, $4448 for LDV and $852 for DCV. EFV, 3TC, ETV and RHZE are already generic in the USA. The US substance patents on atazanavir expire in 2017, TDF in 2018, sofosbuvir in 2030 and daclatasvir in 2031. Sofosbuvir + ledipasvir combination patents expire in 2032.

**Abstract TUAD0104–Table 1.** Prices and year of patent expiry.

Drug	USA	Global lowest	Target	Patent expiry
ATV	$16,093	$170	$151	2017
TDF/3TC/EFV	$23,965	$107	$83	2018
ETV	$5915	$409	$82	Generic
SOF/LDV	$91,207	$408	$85	2032
SOF/DCV	$142,710	$66	$52	2031
TB: RHZE	$945	$27	$28	Generic
Prices: HIV and HBV - per year, HCV - per 12-week course, TB - per standard six-month first-line regimen.				

**Conclusions**: Treatment of HIV, HBV, HCV and TB could be achieved for <$90 per person globally if robust generic competition is enabled. In most countries, generic TDF/3TC/EFV, TDF/3TC, ETV or 1L DS-TB treatment could be available for <$90 by early 2018 after patent expiry. Most HCV direct-acting antivirals (DAAs) will remain on patent for ≥12 more years. Voluntary licensing or other mechanisms will be required to enable access to HCV DAAs at low prices.

## TUAD0105

### Comparative analysis of ARV costs before and after the Clinical Protocol and Therapeutic Guidelines for the management of adult HIV infection (PCDT) was adopted in 2013 in Brazil

MC Pimenta ^1,2^; L Hasenclever^3,4^; CF Rocha^3^; G Cunha^3^; A Ana Pati Pascom^5^; M Freitas^5^; R Girade Corrêa^5^; GF Mendes Pereira^5^; CJ Braga Batista^6^ and J Monteiro da Cruz^6^

^1^Ministry of Health of Brazil, STI/HIV/VH Department, Rio de Janeiro, Brazil. ^2^Veiga de Almeida University, School of Nursing, Rio de Janeiro, Brazil. ^3^Federal University of Rio de Janeiro, Institute of Economics, Rio de Janeiro, Brazil. ^4^University Candido Mendes, Economics, Rio de Janeiro, Brazil. ^5^Ministry of Health of Brazil, STI/HIV/VH Department, Brasilia, Brazil. ^6^Ministry of Health, STI/HIV/VH Department, Brasilia, Brazil

Presenting author email: cpimenta48@gmail.com

**Background**: The PCDT of 2013 established standardization of treatment, practice of early treatment and treatment for all. These initiatives have diverse and opposing consequences on the costs of antiretrovirals (ARVs). Objectives were to know and ponder these consequences by comparing costs between the antiretroviral treatment structure used until 2013 and the PCDT changes applied since 2014. The problem evaluated was if the number of patients and the resulting increase in total costs would be counterbalanced by a significant reduction in average costs as consequence of recommendations for standardization and simplification of treatment, considering the 2020 timeline.

**Methods**: The study covered 2009–2013 and 2014–2015 scenarios before and after the adoption of the PCDT in Brazil by the Institute of Economics of the Federal University of Rio de Janeiro with the Ministry of Health. The study design estimated the total and average costs for the total adult patients treated in December 2009 and 2015, comparing them to estimate the observed cost difference between the two strategies adopted, followed by a simulation of results for 2020. Scenarios of the evolution of costs of HAART took into account treatment targets of the analysed population and changes of schemes. The study population considered were all adult patients under treatment for both periods, 207.014 and 444.093 respectively. Data collection used Systems for Control of Laboratory Tests (CD4 and viral load), for ARV Dispensation, and price records of ARV.

**Results**: Findings indicate decrease in the average costs due to a higher concentration of patients in first-line regimens in 2015. The difference is even more important in the incoming population. The significance is that the total cost of patients in 2015, with an average cost of 41.46% lower, allowed savings of more than 250 million US$. With the average cost of 2009, the total annual cost of patients in 2015 would be 70.83% higher if the PCDT had not been adopted. This is enough to cover 675,000 new patients and more than compensate for the adoption of treatment as prevention.

**Conclusions**: Results become even more relevant since cost savings were accompanied by a better clinical outcome of patients with simplification of treatments.

## TUAD0201

### Association between user fees and dropout from methadone maintenance therapy: results of a cohort study in Vietnam

B Johns^1^; LB Chau^2^; KH Hanh^3^; ND Manh^4^; HM Do^2^; AT Duong ^5^ and LH Nguyen^4^

^1^Abt, International Health, Bethesda, USA. ^2^Hanoi School of Public Health, Hanoi, Vietnam. ^3^Health Financing and Governance Project, Hanoi, Vietnam. ^4^Vietnam Authority of HIV/AIDS Control, Hanoi, Vietnam. ^5^Financing Department, Vietnam Authority of HIV/AIDS Control, Hanoi, Vietnam

Presenting author email: thuyanhvaac@gmail.com

**Background**: Starting in 2015 with the devolution of fiscal responsibility for methadone maintenance therapy (MMT) to provinces in Vietnam, some provinces started to collect user fees at MMT clinics to cover the costs of incidentals. A pilot study in Hai Phong province suggested that user fee collection is feasible and affordable to MMT clients. How user fees might affect clients’ ability to maintain MMT attendance in other provinces has not been assessed. The primary objective of this study is to assess the association between user fees and client dropout from MMT, exploiting the fact that some provinces had implemented user fees in 2015 while other had not. The secondary objective was to estimate the catastrophic payments associated with MMT.

**Methods**: An observational cohort study of 1021 MMT clients in seven provinces from May/June 2015 to May/June 2016. Three provinces implemented user fees, while four provinces did not. Provinces and facilities were randomly selected, while all MMT clinics in the selected provinces were included. Box Cox proportional hazard models were used to assess the association between user fees and (i) dropout and (ii) being inactive.

**Results**: About 85% of the cohort was actively on MMT at the end of the observation period, including 10% of clients had transferred facilities and 1% of clients quit with facility staff permission. About 14% of the cohort had stopped MMT care, about 8% dropped out, 3.5% were incarcerated, 1.5% died and 2% stopping for other reasons. The hazard ratio for paying user fees compared to not paying user fees ranged from 0.70 (unadjusted, *p* = 0.26) to 0.29 (adjusted, *p* = 0.33) for dropout and from 0.75 (unadjusted, *p* = 0.24) to 0.50 (adjusted, *p* = 0.48) for being inactive. However, 29% of clients at facilities paid more than 40% of non-subsistence expenditures for MMT-associated user fees and transportation.

**Conclusions**: Over the course of one year, we do not find evidence that user fees increased dropout or reduced retention in MMT. Overall, dropout rates in Vietnam are very low compared to other countries. However, catastrophic payment rates remain a concern, and longer-term follow-up is needed.

## TUAD0202

### A combination intervention strategy to enhance outcomes across the HIV care continuum and support epidemic control: data from a cluster-randomized trial in Mozambique

B Elul ^1,2^; M Lamb^1,2^; L Ahoua^3^; F Abacassamo^4^; S Kujawski^1^ and M Lahuerta^1,2^

^1^Mailman School of Public Health, Department of Epidemiology, Columbia University, New York, USA. ^2^ICAP at Columbia University, New York, USA. ^3^ICAP at Columbia University, Maputo, Mozambique. ^4^Center for Collaboration and Health, Maputo, Mozambique

Presenting author email: be2124@columbia.edu

**Background**: Identifying practical interventions that can be implemented at scale to enhance outcomes across the HIV care continuum is essential to maximizing individual and population benefits of ART.

**Methods**: Engage4Health, an implementation science study using a cluster-randomized design, was conducted at 10 health facilities in Mozambique and evaluated the effectiveness of a combination intervention strategy (CIS) vs. the standard of care (SOC) on important HIV care continuum outcomes. CIS included: (1) point-of-care CD4+ count at HIV testing sites; (2) accelerated ART initiation for eligible patients; and (3) SMS appointment reminders. A subset of CIS participants additionally received non-cash financial incentives (CIS+FI). Adults >18 years newly diagnosed with HIV and willing to receive HIV care at the diagnosing health facility were enrolled from April 2013 to June 2015 and followed for 12 months. Clinic-based outcomes were assessed using electronic medical records, while death was determined by triangulating data from medical records, patient tracing and mortality registries.

**Results**: A total of 2004 participants (767 SOC, 744 CIS and 493 CIS+FI) were enrolled; the majority (64%) were female, and the mean age was 34 years. In intent-to-treat analyses, participants at CIS vs. SOC sites were more likely to link to care on the day they were diagnosed (89% vs. 16%, relative risk (RR) = 5.60, 95% confidence interval (CI) 4.79–6.69); have their ART eligibility assessed (100% vs. 77%, RR = 1.30, 95% CI 1.25–1.35), be identified as ART eligible (75% vs. 60%, RR = 1.24, 95% CI 1.15–1.33), initiate ART (65% vs. 54%, RR = 1.20, 95% CI 1.10–1.30) and be retained at 12 months (58% vs. 44%, RR = 1.32, 95% CI 1.19–1.45). No additional benefits on these outcomes were observed when FI were added to the CIS. Neither CIS nor CIS+FI had a significant effect on mortality within 12 months of diagnosis or after ART initiation, but mortality prior to ART initiation was lower at CIS+FI vs. SOC sites (1% vs. 4%, RR = 0.27, 95% CI 0.10–0.69).

**Conclusions**: The CIS offers a promising, pragmatic approach for enhancing critical outcomes necessary for epidemic control. Further efforts are needed to identify interventions to reduce mortality, particularly among patients on ART.

## TUAD0203

### Economic evaluation of non-financial incentives to increase couples HIV testing and counselling uptake in Zimbabwe

C Mangenah ^1^; E Sibanda^2^; K Hatzold^3^; G Maringwa^4^; S Gudukeya^5^; N Madidi^3^; O Mugurungi^6^; F Terris-Prestholt^7^; F Cowan^4^ and H Thirumurthy^8^

^1^Centre for Sexual Health, HIV and AIDS Research (CeSHHAR), Health Economics, Harare, Zimbabwe. ^2^Centre for Sexual Health, HIV and AIDS Research (CeSHHAR), Sexual and Reproductive Health Studies, Harare, Zimbabwe. ^3^Population Services International, Zimbabwe, HIV Prevention, Harare, Zimbabwe. ^4^Centre for Sexual Health, HIV and AIDS Research (CeSHHAR), Harare, Zimbabwe. ^5^Population Services International, Zimbabwe, Harare, Zimbabwe. ^6^Ministry of Health and Child Care (MOHCC), Harare, Zimbabwe. ^7^London School of Hygiene and Tropical Medicine (LSHTM), London, UK. ^8^University of North Carolina at Chapel Hill, Chapel Hill, Zimbabwe

Presenting author email: cmangenah1@gmail.com

**Background**: Uptake of couples HIV testing and counselling (CHTC) in southern Africa remains low despite multiple HIV prevention and health benefits. Small incentives can increase CHTC uptake by offsetting real and perceived costs to couples. We estimate cost-effectiveness of providing non-financial incentives for CHTC in a cluster randomized controlled trial (RCT).

**Methods**: Thirty-four communities in rural Zimbabwe received standard community mobilization for HIV testing at mobile clinics while in 34 randomly selected communities individuals seeking CHTC were offered in-kind incentives (choice between a laundry bar, petroleum jelly or cooking oil) worth $10,859.22. Costs for community mobilization, CHTC and incentives were calculated from the programme perspective *(2015 US$)*. Incremental cost-effectiveness ratios (ICERs) were estimated for the number tested, number tested with a partner and number of HIV-positive individuals tested.

**Results**: In control communities, 1062/10,580 (10.0%) individuals tested as couples compared to 7852/14,099 (55.7%) in intervention communities. Overall 530 additional HIV-positive persons were identified in intervention communities. Total incremental intervention cost was $25,687.50, translating to an ICER of $7.98 per couple tested ($3.99 per individual client tested) and $48.47 per HIV-positive diagnosis. Mean costs per person tested in control and intervention communities were $8.18 and $7.96, respectively, with costs per HIV-positive person identified $93.10 and $128.10, respectively.

**Conclusions**: This RCT provides evidence that in addition to increasing HTC access, policymakers, implementers and external donors should consider providing in-kind incentives as they are cost-effective at increasing CHTC uptake and identifying HIV-positive persons.

**Abstract TUAD0203–Table 1.** Economic cost ($) calculation

Capital costs	Intervention cost ($)	% contribution	Control cost ($)	% contribution
Incentives	$10,859.22	10	$0.00	0
Human resources	$78,705.00	70	$69,423.75	80
Equipment	$1154.95	1	$1154.95	1
Medical supplies	$2541.42	2	$1919.76	2
HIV test kits	$2266.16	2	$1537.96	2
Stationary and other supplies	$16,753.95	15	$12,556.78	15
Total cost ($)	$112,280.70	100	$86,593.20	100
Mean cost/client	$7.96		$8.18	
Cost per HIV + client	$93.10		$128.10

**Abstract TUAD0203–Table 2.** Cost-effectiveness analyses.

Intervention incremental cost (Incentive arm–standard mobilization arm)	$25,687.50
Intervention effect (incentive effects–non-incentive effects)	46%
Additional clients tested as a couple (14,099*46%)	6437
Additional clients tested individually	3519
Additional clients tested HIV positive	530
ICER per individual client tested HIV positive	$48.47
ICER per individual client tested ($25,687.50/6437)	$3.99
ICER per couple tested ($3.99x2)	$7.98

## TUAD0204

### The effects of short-term cash and food incentives on food insecurity and labour force participation among HIV-infected adults initiating antiretroviral therapy in rural Tanzania

C Fahey ^1^; PF Njau^2^; N Kapologwe^3^; WH Dow^1^ and SI Mccoy^1^

^1^University of California, Berkeley, USA. ^2^Prevention of Mother-to-Child HIV Transmission Programme, Ministry of Health, Community Development, Gender, Elderly, and Children, Dar es Salaam, United Republic of Tanzania. ^3^President’s Office - Regional Administration and Local Government Authorities (PO-RALG), Shinyanga, United Republic of Tanzania

Presenting author email: cfahey@berkeley.edu

**Background**: We previously demonstrated that short-term cash and food incentives increased antiretroviral therapy (ART) possession and retention in HIV services in Tanzania. To elucidate potential pathways that led to these achievements, we examined whether these incentives also improved food security and labour force participation.

**Methods**: At three clinics, 805 food-insecure adults who recently initiated ART (≤90 days prior) were randomized to receive cash or food transfers (approximately $11/month for ≤6 months, conditional on visit attendance) or standard-of-care (SOC) ART. After six months, we re-assessed food security (Household Hunger Scale), body mass index (BMI), employment and functional limitation (illness prevented work or housework) among patients returning for ART. The incentives’ effectiveness at increasing retention contributed to differential loss-to-follow-up (13% overall; 20% SOC; 8% cash; 15% food; *p* < 0.01), which we accounted for in the current analysis using inverse probability weighting.

**Results**: After six months, food security improved from 0% to 37% (*p* < 0.01) and did not differ comparing the SOC (33%) to cash (36%, *p* = 0.64) or food (39%, *p* = 0.37) groups. Mean BMI increased from 21.5 to 22.7 kg/m^2^ (*p* < 0.01), with no differences comparing SOC (22.5 kg/m^2^) to cash (22.6 kg/m^2^, *p* = 0.76) or food (22.9 kg/m^2^, *p* = 0.15). Employment rose from 58% to 76% (*p* < 0.01), with no differences between SOC (80%) and cash (75%, *p* = 0.47) or food (75%, *p* = 0.43). Functional limitation decreased from 55% in the year before enrolment to 12% in the following six months (*p* = 0.02) and was significantly lower in the food group (9% vs. 18% SOC, adjusted odds ratio (OR) = 0.41, 95% confidence interval (CI): 0.18, 0.96, *p* = 0.04); cash was non-significantly lower (13% vs. 18% SOC, adjusted OR = 0.64, 95% CI: 0.27, 1.5, *p* = 0.32).

**Conclusions**: Food security and employment substantially improved across all study groups after six months of ART. Short-term cash and food incentives did not augment these gains relative to standard-of-care ART, with the exception of significantly less functional limitation among the food group. This suggests that cash and food transfers acted primarily via the price incentive (increasing ART adherence), rather than mitigating socio-economic barriers through income effects. Future studies are needed to better understand the mechanisms through which incentives may increase and sustain retention in HIV services.

## TUAD0205

### Using conjoint analysis to model hospital directors’ decision-making in adoption of an evidence-based stigma-reduction intervention

C Lin ^1^; L Li^2^; S-J Lee^2^ and Z Wu^3^

^1^UCLA, NPI-Center for Community Health, Los Angeles, USA. ^2^UCLA, Los Angeles, USA. ^3^China CDC, Beijing, China

Presenting author email: chunqinglin@hotmail.com

**Background**: Behavioural interventions that have demonstrated efficacy in randomized trial conditions have been underutilized in healthcare delivery. This study used conjoint analysis, a marketing research technique, to quantify the impact of different aspects of intervention in hospital stakeholders’ decision-making in adoption of evidence-based interventions (EBI).

**Methods**: The authors used a real-life intervention with efficacious outcome to reduce HIV-related stigma in healthcare settings as a “product” to study adoption of EBI. Conjoint analysis was conducted among 60 hospital directors recruited from 30 hospitals of different levels and types in Fujian Province, China. The directors evaluated their willingness to adopt the evidence-based stigma reduction intervention in their hospitals by rating across eight hypothetical scenarios with preferred and non-preferred levels of seven attributes, including (1) administrative support, (2) cost, (3) personnel involvement, (4) format, (5) duration, (6) technical support and (7) priority alignment with the hospital. A mixed-effect model was fit to the likelihood of intervention adoption for the eight scenarios, and the seven attributes (categorized as preferred = 1 or not preferred = 0) served as independent variables in the model.

**Results**: Monetary cost of intervention implementation (impact score = 24.8) had the greatest impact on the directors’ willingness to adopt a certain EBI, followed by duration of the intervention (impact score = 10.0), availability of technical support (impact score = 7.5) and flexibility of format (impact score = 4.6). The majority (88.3%) of the hospital directors perceived the conjoint administration process as clear and easy to understand. The data collection time was relatively short, which was approximately 30 minutes.

**Conclusions**: Conjoint analysis was proven to be feasible in modelling hospital directors’ decision-making in adoption of EBI. There were several issues that one should consider when operationalizing conjoint analysis in dissemination and implementation research, including selection of EBI example, assigning the component level of the attributes, generating scenarios and interviewer training. The findings have implications for design and dissemination of existing EBI in healthcare settings to optimize the public health impact.

## WEAA0101

### Impaired Nef’s ability to counteract SERINC5 is associated with decreased plasma viraemia

M Toyoda^1^; D Kamori^1^; J Carlson^2^; H Gatanaga^3^; A Kawana-Tachikawa^4^; S Oka^3^; M Pizzato^5^ and T Ueno ^1^

^1^Kumamoto University, Center for AIDS Research, Kumamoto, Japan. ^2^Microsoft Research, Los Angeles, USA. ^3^National Center for Global Health and Medicine, Tokyo, Japan. ^4^University of Tokyo, Tokyo, Japan. ^5^University of Trento, Trento, Italy

Presenting author email: uenotaka@kumamoto-u.ac.jp

**Background**: It is recently revealed that an HIV-1 accessory protein Nef plays an essential role in virion infectivity by antagonizing a host restriction molecule SERINC5. However, it remains elusive whether Nef’s ability to counteract SERINC5 influences viral fitness *in vivo*. Because Nef is a highly polymorphic protein due to the selective forces by host cellular immunity, we hypothesized that certain immune-escape polymorphisms might affect the Nef function and thereby plasma viraemia.

**Methods**: We collected plasma viral RNA from HLA-typed, treatment-naive, chronically HIV-1-infected subjects (*n* = 375) and analysed DNA sequences of Nef-encoding region. Immune-associated Nef polymorphisms were analysed by a phylogenetic network model. We also introduced several mutations to a control strain and patient-derived Nef clones and tested various Nef functions *in vitro*, including downregulation of CD4 and HLA class I as well as enhancement of virion infectivity and counteraction of SERINC5.

**Results**: We identified 112 Nef polymorphisms that were overrepresented within patients sharing the same HLA genotypes. Specifically, two mutations, Tyr-120 to Phe and Gln-125 to His, were overrepresented in patients carrying HLA-B*51:01 and HLA-C*14:03, and the number of the two mutations correlated inversely with plasma viral load (*p* = 0.004). Nef functional assays demonstrated that the double-mutant Nef impaired in SERINC5 counteraction and enhancement virion infectivity whereas other Nef functions such as CD4 and HLA class I downregulation remained unchanged. Jurkat cells lacking SERINC5 expression lost such functional difference between the parental and mutant Nef clones.

**Conclusions**: Taken together, these results suggest that naturally occurring immune-associated mutations impair Nef’s ability to counteract SERINC5 and enhance virion infectivity, associating with reduced plasma viral load *in vivo*.

## WEAA0102

### HIV-1 concentrates and shelters cell-associated infectivity a “viral biofilm”

C Inizan^1^; A Derames^1^; M Caillet^1^; A David^1^; P Versmisse^1^; A Saez-Cirion^1^; M Mesel-Lemoine^1^; A Mallet^1^; M Sachse^1^; H Mouquet^1^; F Boufassa^2^; O Lambotte^3^; K Bourdic^3^ and M-I Thoulouze ^1^

^1^Institut Pasteur, Virology, Paris, France. ^2^INSERM, CHU Kremlin Bicêtre, Kremlin Bicêtre, France. ^3^CHU Kremlin Bicêtre, AP-HP, Le Kremlin Bicêtre, France

Presenting author email: thoulouz@pasteur.fr

**Background**: Highly active antiretroviral therapy (HAART) does not allow the complete clearance of the virus since it does not target viral reservoirs nor efficiently block HIV-1 cell-to-cell transmission *in vivo*. HIV-1 cell-to-cell spread is thousands-fold more efficient than cell-free infection; yet, how virions are transferred *via* cell contacts remains unknown.

**Methods**: Using a panel of cutting-edge imaging techniques (Cryo-TEM, CL/SEM, CL/FIB and super-resolution imaging) to functional assays, we investigated and characterized viral structures involved in HIV-1 cell-associated infectivity. We analysed a range of infected T-cell cultures (chronically infected T-cell lines, primary CD4+ T cells infected *in vitro* with virus primary isolates and CD4+ T lymphocytes from untreated HIV-1-infected patients with a detectable viraemia).

**Results**: We show here that HIV-1 cell-associated infectivity mostly resides at the surface of CD4+ T lymphocytes in a viral biofilm, formed by viral particles aggregated within a scaffold of extracellular matrix (ECM) components. Our set of data demonstrates that (i) biofilm-associated viral particles are more efficient in establishing infection than free viral particles and (ii) they confer HIV with important properties characterizing cell-to-cell spread. This includes the decreased sensitivity to HAART and to the broad neutralizing antibody 3BNC117. HIV-1 regulates biofilm matrix composition that controls both viral particles organization at the cell surface and the resulting cell-associated infectivity. The organized clustering of viral particles along an ECM framework locally concentrates virions, favours their collective transfer to target cells and limits their exposure to nAbs. Finally, CD4+ T cells from HIV-1-infected patients produce and transmit viral biofilms, supporting that they may also be involved *in vivo*.

**Conclusions**: This study thus unveils HIV biofilm as a new highly infectious extracellular entity that concentrates, stores, disseminates and shelters HIV-1 infectivity. This may have implications for HIV-1 spread and persistence in host, including for maintaining HIV-1 sanctuaries in treated patients. Our results unveil a new role for the ECM in clustering and protecting HIV-1 at the plasma membrane and in their collective transfer through virological synapses. Targeting biofilm ECM components could represent a promising approach to favour HIV-1 clearance or to potentiate the effect of available anti-viral therapies.

## WEAA0103

### Coordinated mTOR-mediated rewiring of nucleotide anabolism regulates HIV-1 infection of CD4 T lymphocytes

HE Taylor ^1^; I Clerc^1^; N Calantone^1^; GE Simmons^2^ and RT D’Aquila^1^

^1^Department of Medicine and Northwestern Medicine HIV Translational Research Center, Northwestern Feinberg School of Medicine, Division of Infectious Diseases, Chicago, USA. ^2^UT Southwestern, Dallas, USA

Presenting author email: harryetaylor@yahoo.com

**Background**: HIV-1 replication is restricted in resting CD4 T lymphocytes. Stimulation of these cells via either the T-cell receptor (TCR) or gamma-cytokine receptors up-regulates HIV provirus formation at levels of reverse transcription (RT) and nuclear import. However, the enhancements of both RT and nuclear import are not fully explained by activation-induced changes in known restriction factors. Here, we study a “master regulator” activated by both TCR and gamma-cytokine signalling: the mechanistic target of rapamycin (mTOR) kinase.

**Methods**: Resting CD4 T cells purified from blood donor PBMCs using immunomagnetic cell separation were stimulated with anti-CD3/anti-CD28 beads, PHA/IL2 or IL7/15. Inhibitors were used before activation to study the role of mTOR. qPCR quantified HIV-1 RT products and 2-long terminal repeat (2-LTR) circles. Flow cytometry after infection with a single-round HIV-1-GFP reporter virus monitored productive infection. Quantitation of dNTPs used ultra-sensitive LC-MS/MS detection. Flow cytometry and immunoblotting assessed effects of treatments on mTOR activity.

**Results**: mTOR activity induced by engagement of either T cell or gamma-cytokine receptors coordinates expression of transporters for glucose (*GLUT1*), glutamine (*ASCT2*) and transferrin (*CD71*), as well as rate-limiting enzymes for pyrimidine (*CAD*), purine (*IMPDH2*) and deoxyribonucleotide (dNTP) synthesis (*RRM1*). mTOR has been previously reported to govern the expression of nutrient transporters and pyrimidine biosynthetic genes, but this is the first demonstration of this global mTOR-dependent programme in activated CD4 T lymphocytes. Pharmacological ablation of mTOR activity suppressed dNTP pool expansion after activation. Multiple chemically distinct catalytic inhibitors of mTOR were found to reduce HIV-1 RT products after TCR stimulation. Moreover, both TCR and gamma-cytokine-activation induced mTOR inhibitor-sensitive accumulation of 2-LTR circular forms of HIV-1 DNA, indicating that mTOR activity also regulates active, energy (GTP/ATP)-dependent HIV-1 nuclear import.

**Conclusions**: CD4 T lymphocyte activation-induced mTOR “metabolic reprogramming” drives increased susceptibility to HIV-1 by expanding key nucleotide substrate and energy pools necessary for both reverse transcription and nuclear import. This adds mechanistic understanding, confirms earlier reports that catalytic inhibitors of mTOR hold promise for improving HIV-1 chemotherapy and prevention and suggests continued investigation of mTOR’s role in establishment as well as reactivation of HIV-1 infection.

## WEAA0104

### Membrane-associated RING-CH (MARCH) 1 and 2 are other members of MARCH proteins that inhibit HIV-1 infection

W Yao^1,2^; T Tada^1^; Y Zhang^1,2^; H Fujita^3^; S Yamaoka^2^ and K Tokunaga ^1^

^1^Department of Pathology, National Institute of Infectious Diseases, Tokyo, Japan. ^2^Department of Molecular Virology, Tokyo Medical and Dental University, Tokyo, Japan. ^3^Faculty of Pharmaceutical Sciences, Nagasaki International University, Nagasaki, Japan

Presenting author email: tokunaga@nih.go.jp

**Background**: Membrane-associated RING-CH 8 (MARCH8), which is one of the 11 members of MARCH family, downregulates several host membrane proteins (MHC-II, CD86, transferrin receptor, etc.). We have recently reported that this protein also targets HIV-1 envelope glycoproteins and acts as an antiviral factor (*Nat. Med*. 21:1502–1507, 2015). It remains unclear whether other family members might have similar antiviral functions to those seen in MARCH8. Here, we show that MARCH1 and MARCH2 are such MARCH family members that reduce virion incorporation of envelope glycoproteins.

**Methods**: Plasmids expressing family members of MARCH (MARCH1, MARCH2, MARCH3, MARCH5, MARCH6 and MARCH7) were created by RT-PCR-amplifying their mRNAs. The HIV-1 proviral luciferase indicator plasmid was cotransfected with plasmids expressing either HIV-1 Env or VSV-G, and those expressing MARCH family members including MARCH8, into 293T cells. Supernatants were subjected to assays for infectivity, viral entry or virion incorporation, and producer cells were used for flow cytometry. Real-time RT-PCR was performed to determine endogenous levels of MARCH1 and MARCH2 expression.

**Results**: Infectivity assays showed that, two other members of MARCH family, MARCH1 and MARCH2 had the antiviral activity in a dose-dependent manner. The expression of these proteins in virus-producer cells decreased the efficiency of viral entry and indeed downregulated HIV-1 envelope glycoproteins from the cell surface, resulting in reduced incorporation of envelope glycoproteins into virions, exactly as observed in MARCH8 expression. Endogenous expression of MARCH1 and MARCH2 was enhanced in monocyte-derived macrophages by treatment with type I interferon.

**Conclusions**: As the antiviral MARCH family members, MARCH1 and MARCH2 join a growing list of host factors that inhibit HIV-1 infection.

## WEAA0105

### Bicaudal D2 facilitates the cytoplasmic trafficking and nuclear import of HIV-1 genomes during infection

A Dharan^1^; S Opp^2^; F Diaz-Griffero^2^ and E Campbell ^1^

^1^Loyola University Chicago, Microbiology and Immunology, Maywood, USA. ^2^Albert Einstein School of Medicine, Microbiology and Immunology, Bronx, USA

Presenting author email: ecampbell@luc.edu

**Background**: Numerous viruses, including HIV-1, exploit the microtubule network to traffic towards the nucleus during infection. Although numerous studies have observed a role for the plus end microtubule motor dynein in HIV-1 infection, the mechanism by which the viral core containing the viral genome associates with dynein and induce their perinuclear trafficking has remained unclear.

**Methods**: We utilized CRISPR-mediated depletion of the dynein adapter protein Bicaudal D2 (BICD), monitored the stages of viral replication by qPCR and measured viral trafficking using live cell imaging and the induction of antiviral gene expression by RT-PCR.

**Results**: We observe that BICD2 depletion inhibits infection, preventing the nuclear entry of the viral genome, while fusion and reverse transcription are unimpaired. Using live cell imaging, we show that viral trafficking is altered in BICD2-depleted cells and show that NC-CA tubes bind cellular BiCD2. Finally, we observe that disruption of the CA–BiCD2 interaction in the monocytic cell line THP-1 results in the accumulation of viral genomes in the cytoplasm and the induction of interferon-stimulated genes in these cells.

**Conclusions**: These studies demonstrate that BICD2 is required for efficient HIV-1 infection and reveal the BICD2/CA interaction as a novel target for possible therapeutic interventions designed to increase innate immune activation in response to HIV-1 infection.

## WEAA0201

### Investigating the SHIV reservoir in a non-human primate model following allogeneic bone marrow transplantation

L Colonna ^1,2^; JB Schell^2^; CW Peterson^3^; J Carlson^2^; M Brown^2^; V Tkachev^2^; A Taraseviciute^1^; SN Furlan^1^; H Zheng^1^; DJ Hunt^2^; A Yu^2^; J Lane^1^; CR Moats^1^; K Vogel^1^; CE Hotchkiss^1^; RD Murnane^1^; A Baldessari^1^; C English^1^; M Hoffman^2^; K Zeleski^2^; J Olvera^2^; CA Astley^1^; S Wangari^1^; B Agricola^1^; J Ahrens^1^; N Iwayama^1^; P Polacino^1^; HM Mack^1^; S-L Hu^1^; L Stensland^1^; M-LW Huang^1^; HP Kiem^3^ and LS Kean^1,2,3^

^1^University of Washington, Seattle, USA. ^2^Seattle Children’s Research Institute, Ben Towne Center for Childhood Cancer Research, Seattle, USA. ^3^Fred Hutchinson Cancer Research Center, Seattle, USA

Presenting author email: lcolonna@uw.edu

**Background**: Three components are thought to have contributed to the Berlin patient’s cure following BMT: myeloablative conditioning, graft-versus-viral reservoir (GVVR) and HIV resistance of the donor CCR5D32 cells, yet the contributions of each to reservoir clearance is unknown. Moreover, while the Boston patients’ viral rebound suggests that HIV resistance will be key, this has not yet been rigorously determined. To dissect the role of myeloablative conditioning, GVVR and viral resistance on the SHIV reservoir, we have developed the first NHP model for allogeneic BMT in SHIV-infected, cART-treated rhesus macaques.

**Methods**: We intravenously infected six animals with SHIV-1157ipd3N4 and left them untreated for six months, followed by six months of cART (raltegravir, PMPA and FTC). Three animals remained untransplanted, and three animals received myeloablative total body irradiation (1020 Gy) followed by haplo-identical BMT without ART discontinuation. Donor chimerism was monitored, and viral DNA and RNA were measured by qPCR in approximately 35 tissues following necropsy.

**Results**: All animals showed peak viral titres in the range of approximately 10^7^ copies/ml approximately two-week post-infection, which subsequently reached steady state. One animal controlled viraemia before ART initiation (day 184). In the other animals, plasma viral RNA became undetectable two to three weeks post-cART initiation. The three transplanted animals were euthanized at day 47, 29 and 9 post-transplant, due to infection, acute graft-versus-host disease and renal complications, respectively. Analysis of whole blood and sorted CD4+ T cells showed rapid acquisition of 100% donor blood chimerism, with lower chimerism in multiple tissues. Despite undetectable viraemia post-transplant, viral DNA quantification in 35 tissues, including haematopoietic and major organs, CNS and reproductive organs, revealed an increased reservoir in transplanted non-controllers versus untransplanted controls.

**Conclusions**: Our results indicate that the DNA reservoir significantly increases early after transplant, despite control of peripheral viraemia. This suggests that transplant represents a significant initial “shock” with loss of anti-HIV immunity contributing to reservoir enlargement, potentially explaining the rebound observed in the Boston patients. We believe that full recovery of anti-HIV immune control may be restored if additional HIV resistance factors and/or anti-HIV strategies are incorporated post-transplant to enhance the targeted killing of infected cells.

## WEAA0202

### Investigating clinical therapeutics to target infected cells and promote HIV clearance

P Arandjelovic ^1,2^; C Allison^1,2^; S Preston^1,2^; J Cooney^1,2^ and M Pellegrini^1,2^

^1^The Walter and Eliza Hall Institute of Medical Research, Infection and Immunity, Melbourne, Australia. ^2^Department of Medical Biology, The University of Melbourne, Melbourne, Australia

Presenting author email: arandjelovic.p@wehi.edu.au

**Background**: Efforts to eliminate the latent HIV reservoir highlight the need for alternative strategies geared towards killing latently infected cells. HIV manipulates host apoptotic pathways in order to sustain the infection cycle and evade immune surveillance. BH3-mimetic inhibitors antagonize pro-survival proteins and promote intrinsic apoptosis. Here, we employ a novel humanized mouse model of latent HIV infection, together with *in vitro* cell survival assays, to investigate the usefulness of BH3-mimetics in clearing the latent reservoir and achieving a functional cure.

**Methods**: Primary human CD4^+^ T lymphocytes were isolated from healthy donors, activated and infected with HIV-GFP. Infected primary CD4^+^ T cells were treated with BH3-mimetics and assessed for death of GFP-positive, HIV-infected cells. Briefly, humanized mice were generated as follows: NOD.Cg-*Prkdc^scid^ Il2rg^tm1Wjl^*/Sz (NSG) neonates were sub-lethally irradiated in order to achieve myeloablation, followed by injection of human CD34^+^ cord blood haematopoietic stem cells into the orbital vein. Mice were assessed for degree and quality of reconstitution at 16 weeks of age before infection with HIV. Virological suppression was recapitulated using a clinically relevant ART regimen before testing novel interventions.

**Results**: We first sought to investigate the *in vitro* sensitivity of actively infected cells to pro-apoptotic interventions. Treatment with the pre-clinical BH3-mimetic ABT-737, which targets pro-survival proteins Bcl-2, Bcl-xL and Bcl-w, resulted in preferential killing of HIV-infected cells in a concentration-dependent manner. A similar outcome was observed using the FDA-approved, clinical-stage Bcl-2 inhibitor Venetoclax. Our humanized mouse model successfully recapitulates key stages of human disease, including chronic infection, viral suppression, as well as subsequent rebound following treatment interruption. Suppressed mice were administered Venetoclax over three treatment cycles before therapy was interrupted to assess for viral rebound. Humanized mice treated with Venetoclax experienced a substantial delay in rebound up to one week longer than vehicle-treated controls.

**Conclusions**: These results pave the way for further *in vivo* studies using a humanized mouse model of HIV latency. Efforts are underway to optimize treatment regimens as well as to investigate the combination of BH3-mimetics with traditional latency-reversing agents. Overall, these findings represent a novel approach to killing latently infected cells and purging the proviral reservoir.

## WEAA0203

### Inhibiting memory CD4+ T cell self-renewal to reduce HIV persistence

M Mavigner ^1^; M Zanoni^2^; J Habib^1^; C Mattingly^2^; J Demarest^3^; H Kouji^4^; B Lawson^2^; T Vanderford^2^; G Tharp^2^; S Bosinger^2^; G Silvestri^2^ and A Chahroudi^1^

^1^Emory University School of Medicine, Pediatrics, Atlanta, USA. ^2^Emory University, Yerkes National Primate Research Center, Atlanta, USA. ^3^ViiV Healthcare, Durham, USA. ^4^Prism Biolab, Kanagawa, Japan

Presenting author email: maud.mavigner@emory.edu

**Background**: Long-lived memory CD4^+^ T-cells with high self-renewal capacity such as central memory (T_CM_) T-cells and T memory stem cells (T_SCM_) are major contributors to the viral reservoir in HIV-infected individuals on antiretroviral therapy (ART). The Wnt/beta-catenin signalling pathway primarily regulates the balance between self-renewal and differentiation of T_SCM_/T_CM_, and pharmacological manipulation of this pathway offers an opportunity to reduce persistence of latently infected cells.

**Methods**: To block self-renewal and promote T_SCM_/T_CM_ differentiation, we used an inhibitor targeting the Wnt/beta-catenin pathway initially developed to target cancer stem cells (called PRI-724). We evaluated the safety of PRI-724 with a dose-escalation study in healthy rhesus macaques (RM). We then conducted an efficacy study in 12 SIV_mac251_-infected RM treated with ART. After suppression of viraemia, 8/12 RM received PRI-724 at 10 or 20mg/kg/day, while 4/12 RM were maintained on ART only. The gene profile of CD4^+^ memory T-cell subsets was assessed longitudinally by RNAseq analyses. RM were sacrificed on ART following 12 weeks of PRI-724 treatment for assessment of SIV reservoirs.

**Results**: PRI-724 was well tolerated in both healthy and ART-treated SIV-infected RM, with blood counts, liver function and renal function within normal limits. No alteration of tri-lineage haematopoiesis was observed in bone marrow. The transcriptomic profiling of genes associated with T-cell differentiation showed an increased expression of the EOMES transcription factor in the CD4^+^ T_SCM_ of PRI-treated animals compared to controls. Moreover, Gene Set Enrichment Analyses showed that gene sets distinguishing effector versus memory were significantly enriched in the CD4^+^ T_SCM_ compared to the naive T-cells. Interestingly, this CD4^+^ T_SCM_ enrichment in effector-like genes was significantly higher in the PRI-treated than in the control RM. These results suggest an effect of PRI-724 on CD4^+^ T_SCM_ differentiation. PCR analyses in the sorted subsets of memory CD4^+^ T-cells showed no significant difference in cell-associated SIV DNA levels between PRI-724-treated and PRI-724-untreated RM.

**Conclusions**: This work provides novel support for the strategy of promoting CD4^+^ T_SCM_ differentiation by pharmacological modulation of the Wnt/beta-catenin pathway. However, combination strategies targeting stem cell pathways and/or additional anti-reservoir strategies are likely necessary to reduce SIV/HIV persistence.

## WEAA0204

### PBMCs from patients with chronic myeloid leukaemia treated with different tyrosine kinase inhibitors show variable susceptibility to HIV-1 infection: searching for the best therapeutic approach

M Bermejo^1^; J Ambrosioni^2^; G Bautista^3^; N Climent^2^; E Mateos^1^; C Rovira^2^; R Duarte^3^; F Cervantes^2^; M Plana^2^; JM Miro^2^; J Alcami^1^ and M Coiras ^1^

^1^National Center of Microbiology, Instituto de Salud Carlos III, Madrid, Spain. ^2^Hospital Clinic, Barcelona, Spain. ^3^Hospital Puerta de Hierro, Madrid, Spain

Presenting author email: mcoiras@isciii.es

**Background**: Tyrosine kinase inhibitors (TKIs) such as imatinib, nilotinib, dasatinib, bosutinib and ponatinib are currently used for treating chronic myeloid leukaemia (CML). Imatinib was the first TKI introduced in clinical practice and is very selective of the chimeric fusion protein BCR-ABL responsible for causing CML. Second-generation TKIs nilotinib, dasatinib and bosutinib are 20-, 300- and 200-fold more potent than imatinib against BCR-ABL, respectively. Third-generation ponatinib has been approved for patients resistant to other TKIs. Our group demonstrated that dasatinib interferes with SAMHD1 phosphorylation, preserving its antiviral activity and avoiding HIV-1 retrotranscription and proviral integration. Therefore, using dasatinib as adjuvant of antiretroviral therapy in HIV-infected patients could reduce the formation of the viral reservoir. In this work, we evaluated which TKI would be the best choice for treating HIV-infected patients.

**Methods**: PBMCs isolated from 42 CML patients on treatment with imatinib, nilotinib, dasatinib, bosutinib or ponatinib and 44 healthy controls. All patients were treated for >2 years with normal TKI dose; they showed no HIV-1 infection, normal lymphocyte count and general good health.

**Results**: (1) IC_50_ for inhibiting HIV-1 replication was calculated *in vitro* for each TKI: imatinib IC_50_ = 10µM; nilotinib IC_50_ = 10µM; dasatinib IC_50_ = 37.5nM; bosutinib IC_50_ = 0.5µM; ponatinib IC_50_ = 150nM. (2) *Ex vivo* infection of PBMCs from CML patients showed that imatinib, dasatinib and bosutinib effectively inhibited both early and late reverse transcription (*p *< 0.001). Ponatinib was also very effective, but we only recruited one patient. (3) PBMCs from CML patients showed lower SAMHD1 phosphorylation after PHA/IL-2 treatment for five days (*p *< 0.0001 for dasatinib; *p *< 0.01 for bosutinib). (4) Proviral integration was inhibited after treatment with all TKIs, being dasatinib the most significant (*p *< 0.01). (5) Synthesis of viral proteins was mostly significantly interfered by dasatinib (*p *< 0.0001).

**Conclusions**: PBMCs from CML patients on chronic treatment with TKIs showed resistance to SAMHD1 phosphorylation after activation with PHA/IL-2. HIV-1 reverse transcription and proviral integration was reduced in these cells. Dasatinib was the most potent against HIV-1 replication, closely followed by ponatinib. Consequently, preserving SAMHD1 antiviral function with TKIs such as dasatinib could reduce the reservoir size during HIV-1 acute infection.

## WEAA0205

### Eradication without reactivation: suppression of HIV-1 transcription and reactivation from latency by a Tat inhibitor

C Kessing^1^; G Mousseau^1^; C Nixon^2^; H Takata^3^; C Li^1^; R Arora^4^; P Tsai^2^; S Mediouni^1^; L Trautmann^3^; JV Garcia^2^ and S Valente ^5^

^1^The Scripps Research Institute, Jupiter, USA. ^2^University of North Carolina, Chapel Hill, USA. ^3^US Military HIV Research Program, Silver Spring, USA. ^4^The Scripps Research Institute, Jupiter, USA. ^5^The Scripps Research institute, Immunology and Microbial Sciences, Jupiter, USA

Presenting author email: svalente@scripps.edu

**Background**: HIV persists in latently infected CD4^+^T cells in infected individuals even after prolonged periods on suppressive antiretroviral therapy (ART). Transcriptional reactivation from latency is not inhibited by current ART, highlighting the need for novel approaches. The HIV-1 Tat protein exponentially activates viral transcription, and limited Tat-transactivation correlates with HIV-1 latency establishment. We identified didehydro-cortistatin A (dCA) as a potent Tat inhibitor. Over time, treatment of infected cells with dCA drives HIV-1 gene expression into an induced state of persistent latency, refractory to viral reactivation by a standard panel of latency-reversing agents (LRAs), suggesting that the HIV promoter is epigenetically repressed. We postulated a strategy for a functional cure, dubbed “block-and-lock”, where a specific HIV-1 transcriptional inhibitor would promote a durable state of latency, reduce ongoing viral replication during ART and inhibit spontaneous reactivation during ART or when ART is discontinued.

**Methods**: We will present our long-term studies of the activity of dCA in primary human CD4^+^ T cells isolated from aviraemic infected individuals, using an optimized approach that maintains cell cultures up to 10 weeks. To explore the *in vivo* efficacy of dCA, we used the bone marrow–liver–thymus (BLT) humanized mouse model of HIV-1 latency. We will also discuss epigenetic mechanisms regulating dCA-induced deep latency in CD4^+^ T memory T cells, as well as mechanisms of viral evasion to dCA.

**Results**: We demonstrate in CD4+ T cells from five suppressed infected individuals that not only combining dCA with ART promotes faster HIV-1 suppression, but more importantly that prior treatment with dCA significantly reduces viral rebound up to 25 days of treatment interruption, even when viral reactivation is stimulated by LRAs. In the BLT mouse model of latency, we demonstrate that adding dCA to ART-suppressed mice, for a period of 14 days, results in a significant 1.5–10.5-fold reduction in viral RNA production in all tissues. Experiments assessing the ability of dCA to inhibit viral rebound upon treatment interruption are ongoing and will be presented.

**Conclusions**: In combination with ART, dCA abrogates residual HIV production from cellular reservoirs, blocks viral reactivation and may ultimately reduce reservoir replenishment and latent reservoir size.

## WEAA0206

### Myeloablative conditioning is dispensable for transplant-dependent HIV cure

C Peterson^1,2^; J Kaur^1^; C Benne^3^; P Polacino^4^; A Filali-Mouhim^3^; W Obenza^1^; M-L Huang^5^; K Jerome^5,6^; S-L Hu^4,7^; R Sekaly^3^ and H-P Kiem ^1,2,8^

^1^Fred Hutchinson Cancer Research Center, Clinical Research Division, Seattle, USA. ^2^Department of Medicine, University of Washington, Seattle, USA. ^3^Case Western Reserve University, Cleveland, USA. ^4^Washington National Primate Research Center, Seattle, USA. ^5^Fred Hutchinson Cancer Research Center, Vaccine and Infectious Disease Division, Seattle, USA. ^6^Department of Laboratory Medicine, University of Washington, Seattle, USA. ^7^Department of Pharmaceutics, University of Washington, Seattle, USA. ^8^Department of Pathology, University of Washington, Seattle, USA

Presenting author email: jisenber@fredhutch.org

**Background**: Over 10 years ago, haematopoietic stem cell transplant (HSCT) with infection-resistant CCR5Δ32 cells led to cure/remission of HIV infection and leukaemia in the Berlin patient. HIV cure was likely a combined result of the conditioning regimen, an allogeneic “graft versus reservoir” effect, and the CCR5Δ32 donor cells. We studied the impact of conditioning and CCR5-edited cells in simian–human immunodeficiency virus (SHIV)-infected macaques suppressed by combination antiretroviral therapy (cART). The goal of this study was to identify the mechanisms by which each facet impacted the latent viral reservoir.

**Methods**: Pigtailed macaques were challenged with CCR5-tropic, HIV-enveloped SHIV and suppressed by cART following viral set point. In one cohort, autologous HSCT was performed with unmodified stem cells. In a second cohort, animals received CCR5-edited stem cells. Control animals were infected and suppressed, but did not undergo transplantation. Flow cytometry and ELISA were used to monitor changes in immune homeostasis, and quantitative PCR and viral reservoir assays were used to identify virologic changes between experimental and control animals.

**Results**: The conditioning regimen resulted in a dramatic, but incomplete depletion of CD4^+^, CD8^+^ T-cells and B-cells, disrupted T-cell homeostasis, elevated markers of microbial translocation, a significant loss of SHIV-specific antibodies and increased viral rebound, relative to untransplanted controls. Although CCR5 editing did not reach threshold levels for post-cART control of viraemia, correlates of protection were observed: quantitative viral outgrowth, Tat/Rev-induced limiting dilution and tissue viraemia assays showed that the size of SHIV reservoirs was impacted.

**Conclusions**: Our data identify perturbations of the immune system as a mechanism for the failure of autologous transplantation (without gene-protected cells) to eradicate HIV, highlighting the importance of an intact immune system for viral control after cART withdrawal. We conclude that reduced-intensity conditioning, which is safer and less toxic, should be a focus for transplant-based approaches. Analogous to cutting-edge therapies for cancer patients, next-generation HIV cure strategies should balance killing of virus-infected target cells with retention of greater immune function, for example, with immune modulators. High-efficiency gene therapy/gene editing to protect transplanted cells and actively target the viral reservoir during ongoing cART will be essential.

## WEAB0101

### Trends and predictors of non-communicable disease multi-morbidity among HIV-infected adults initiating ART in Brazil, 2003–2014

J Castilho ^1^; MM Escuder^2^; V Veloso^3^; JO Gomes^2^; K Jayathilake^1^; S Ribeiro^3^; RA Souza^4^; ML Ikeda^5^; PR de Alencastro^5^; U Tupinanba^6^; C Brites^7^; C McGowan^1^; A Grangeiro^8^ and B Grinsztejn^3^

^1^Vanderbilt University Medical Center, Division of Infectious Diseases, Nashville, USA. ^2^São Paulo State Department of Health, Health Institute, São Paulo, Brazil. ^3^Oswaldo Cruz Foundation, Evandro Chagas Clinical Research Institute, Rio de Janeiro, Brazil. ^4^São Paulo State Department of Health, AIDS Reference and Training Center, São Paulo, Brazil. ^5^Rio Grande do Sul State Department of Health, Care and Treatment Clinic of the Partenon Sanatorium, Porto Alegre, Brazil. ^6^Federal University of Minas Gerais School of Medicine, Belo Horizonte, Brazil. ^7^Federal University of Bahia, Edgar Santos University Hospital Complex, Salvador, Brazil. ^8^Department of Preventive Medicine, University of São Paulo School of Medicine, São Paulo, Brazil

Presenting author email: jessica.castilho@vanderbilt.edu

**Background**: HIV-infected adults on ART experience high rates of non-communicable diseases (NCDs). These comorbidities can accumulate, and older HIV-infected adults often suffer from multi-morbidity. Little is known of the burden of multi-morbidity in HIV-infected adults in low- and middle-income countries.

**Methods**: HIV-Brazil Cohort is an observational, multi-site study of HIV-infected adults initiating ART between 2003 and 2014. We studied NCDs and multi-morbidity in patients from seven clinical sites in six Brazilian cities. NCDs included coronary artery disease, cerebrovascular disease, high-grade hyperlipidaemia (HLD), diabetes, chronic kidney disease, cirrhosis, osteoporosis, osteonecrosis, venous thromboembolism (VTE) and non-AIDS-defining cancers. Multi-morbidity was defined as the incident accumulation of two or more unique NCDs. We examined incidence trends using Poisson regression and predictors of multi-morbidity using competing risk and Cox regression models.

**Results**: Of the 5786 adults included, 388 (7%) developed multi-morbidity during the study period. From 2003 to 2014, parallel to the rise of patients over the age of 50 in the cohort, the incidence of multi-morbidity rose to 21 patients per 1000 person-years (Figure 1). HLD and VTE incidence decreased while diabetes and osteoporosis rates significantly increased from 2003 to 2014. In adjusted Cox models, female sex, age and low CD4 nadir at baseline were significantly associated with risk of multi-morbidity (Table 1, also adjusting for education, race, year, hepatitis C). Among all patients with multi-morbidity, the most common NCDs were HLD (87%) and diabetes (59%); however, women with multi-morbidity were more likely to have osteoporosis than men (15.4% vs. 6.8%).

**Abstract WEAB0101–Figure 1.**
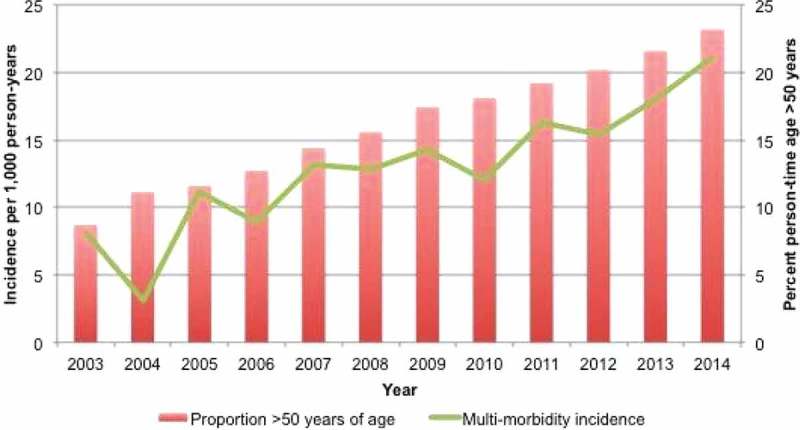
Incidence of multi-morbidity and proportion of patients over 50 years by year.

**Conclusions**: Multi-morbidity of NCDs increased from 2003 to 2014. Females were more likely to develop multi-morbidity in adjusted analyses. Further studies examining sex-specific screening and prevention and management of NCD comorbidities in HIV-infected adults are needed.

**Abstract WEAB0101–Table 1.** Multivariable Cox proportional hazard model.

	Adjusted hazard ratio [95% CI]	*p* Value
Female sex (ref: male sex)	1.35 [1.06–1.70]	0.014
CD4 cell count nadir at baseline (μ/l)		
>200	(ref)	
100–199	1.59 [1.18–2.13]	0.002
<100	1.77 [1.35–2.32]	<0.001

## WEAB0102

**HIV infection and the risk of peripheral arterial disease**

M Freiberg ^1,2^; M Duncan^3^; A Justice^4,5^; J Beckman^3^; Veterans Aging Cohort Study

^1^Vanderbilt University Medical Center, Medicine, Nashville, USA. ^2^Tennessee Valley Veterans Affairs Medical Center, Nashville, USA. ^3^Vanderbilt University Medical Center, Nashville, USA. ^4^Yale University School of Medicine, New Haven, USA. ^5^West Haven Veterans Affairs Medical Center, West Haven, USA

Presenting author email: matthew.s.freiberg@vanderbilt.edu

**Background**: Peripheral arterial disease (PAD) affects approximately 8–10 million U.S. adults annually and is the second most common clinical manifestation of atherosclerosis after acute myocardial infarction (AMI). While the increased risk of AMI and ischemic stroke among HIV-infected (HIV positive) compared to uninfected people is well documented, data linking HIV to incident PAD events are sparse. We, therefore, compared PAD risk among HIV-positive and uninfected veterans.

**Methods**: We analysed data on 91,457 veterans (33% HIV positive) without prevalent cardiovascular disease from the Veterans Aging Cohort Study (VACS). VACS is an observational, longitudinal cohort of HIV-positive veterans matched 1:2 with uninfected veterans on age, gender, race/ethnicity and clinical site. Participants were followed from their first clinical encounter on or after 1 April 2003 until a PAD event, death, their last follow-up date or 30 September 2012. We used ICD-9 and CPT codes to identify participants with incident PAD. Cox proportional hazard regression models were utilized to assess the association between HIV, CD4+ T cell count and PAD adjusting for atherosclerotic risk factors (Table 1). Finally, we constructed cumulative incidence curves to examine PAD risk stratified by HIV status and CD4+ T cell count.

**Results**: During a median follow-up of seven years, there were 5091 PAD events. See Table 1 and Figure 1 for rates and risk of PAD stratified by HIV status and CD4+ T cell count.

**Conclusions** **and Relevance**: HIV-positive veterans have a significantly higher risk of PAD than uninfected veterans.

**Abstract WEAB0102–Table 1.** Rates and risk of PAD by HIV status and CD4+ T cell.

Group	*N*	PAD events	Rate/1000 PY [95% CI]	Unadjusted PAD risk [HR 95% CI]	Adjusted PAD risk [HR 95% CI]^a^
HIV uninfected	61,498	3103	8 [7.8, 8.4]	1.00	1.00
HIV positive, CD4: 500+	10,682	663	10 [9.4, 11.0]	1.23 [1.13, 1.34]	1.31 [1.20, 1.43]
HIV positive, CD4: 200, 500	12,368	835	11 [10.6, 12.1]	1.41 [1.31, 1.52]	1.46 [1.35, 1.59]
HIV positive, CD4: <200	6909	490	14 [12.8, 15.3]	1.77 [1.60, 1.95]	1.62 [1.45, 1.80]

^a^Adjusted for age, sex, race/ethnicity, hypertension, diabetes, LDL and HDL cholesterol, triglycerides, HCV infection, smoking status, renal disease, BMI, anaemia, cocaine dependence or abuse, alcohol dependence or abuse and COPD.

**Abstract WEAB0102–Figure 1.**
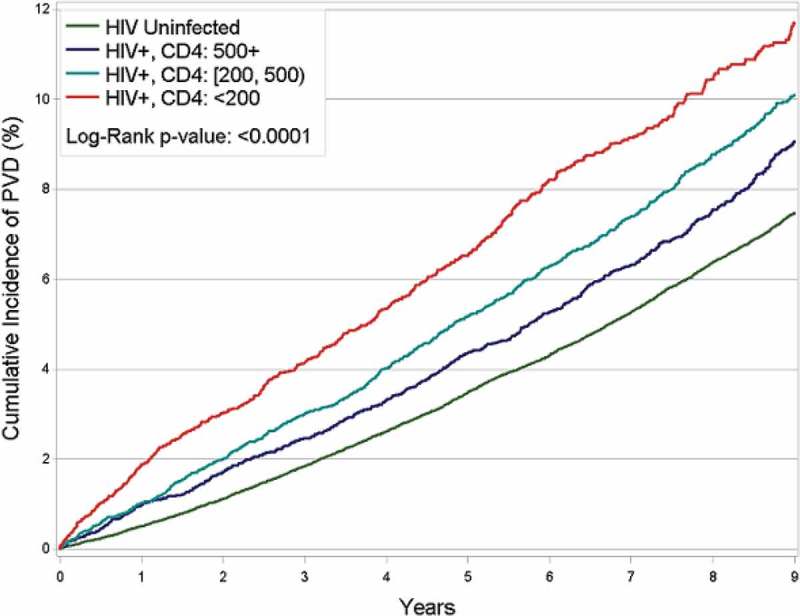
Cumulative incidence of PAD stratified by HIV status and CD+ T cell count.

## WEAB0103

### Impact of exposure to each antiretroviral treatment (ARV) on the risk of fracture in HIV-1-infected individuals: an analysis from FHDH ANRS CO4

D Costagliola ^1^; V Potard^1,2^; S Lang^1,2^; S Abgrall^1,3^; C Duvivier^4,5^; H Fischer^6^; V Joly^7^; J-M Lacombe^1,2^; M-A Valantin^1,8^; M Mary-Krause^1^; S Rozenberg^9^; on behalf of the FHDH ANRS CO4

^1^Sorbonne Universités, INSERM, UPMC Univ Paris 06, Institut Pierre Louis d’épidémiologie et de Santé Publique (IPLESP UMRS 1136), Paris, France. ^2^INSERM Transfert, Paris, France. ^3^AP-HP, Hôpital Antoine Béclère, Service de Médecine interne/Immunologie clinique, Clamart, France. ^4^AP-HP, Hôpital Necker-Enfants Malades, Service des maladies infectieuses et tropicales, Paris, France. ^5^Institut Pasteur, Centre Médical de l’Institut Pasteur, Paris, France. ^6^ACT-UP Paris, Paris, France. ^7^AP-HP, Hôpital Bichat, Service de maladies infectieuses et tropicales, Paris, France. ^8^AP-HP, Hôpital Pitié-Salpêtrière, Service de maladies infectieuses et tropicales, Paris, France. ^9^AP-HP, Hôpital Pitié-Salpêtrière, Service de rhumatologie, Paris, France

**Background**: HIV-infected patients have a lower bone mineral density (BMD) and a higher incidence of fractures compared to the general population of same age and sex. To assess the impact of exposure to each ARV on the risk of osteoporotic fractures, we conducted a nested case-control study.

**Methods**: Cases were individuals enrolled while ART naive, with a first prospectively reported fracture between January 2000 and December 2010. Controls were randomly selected after matching on sex, age (±3 years), diagnosis period (<1997/≥1997) and clinical centre. The risk of fracture was analysed using conditional logistic regression models. Exposure to each ARV was measured either by cumulative duration of exposure (model1) or by exposure yes/no (model2). In a third model, the exposure variable was chosen for each ARV according to the lowest Akaike criterion value. All exposure variables and potential confounders were included in the multivariable models.

**Results**: Among the 861 reviewed fractures, 261 were osteoporotic fractures and 254 were matched to at least one control (376 controls). The median year of fracture diagnosis was 2007 (interquartile range 2004–2009), 67% of cases were men, 71% diagnosed with HIV infection before 1997, median age was 49 years, CD4 436 [293–592], nadir CD4 196 [82–287], 31% at AIDS-stage, 65% with VL <50 cp/ml and 49% exposed to tenofovir, 82% to PIs and 37% to efavirenz. After accounting for transmission group, AIDS stage, geographic origin, BMI, current smoking, alcohol consumption, exposure to systemic glucocorticoids and period of enrolment, no association was found between the risk of fracture and exposure to tenofovir (odds ratio (OR) for cumulative exposure: 1.03 [0.86–1.24], similar results for exposure yes/no), or to NRTIs, or to PIs (exposure to PI overall: OR 1.01 [0.92–1.11] or to each PI). Cumulative exposure to efavirenz was associated with a lower risk of fracture in models 1 and 3 (respective OR 0.81 [0.69–0.95] and 0.82 [0.70–0.96] per year of exposure), while exposure to efavirenz (yes/no) was not (OR 0.84 [0.51–1.40]). Sensitivity analyses do not favour the causal nature of the association with exposure to efavirenz.

**Conclusions**: There was no evidence of excess risk of fracture following exposure to tenofovir or to PIs. This is an important result in the debate about TAF versus generic tenofovir.

## WEAB0104

### Being HIV-1 infected is independently associated with decreased erectile function among older men who have sex with men

M Dijkstra ^1^; RHW van Lunsen^2^; KW Kooij^3^; U Davidovich^1^; RA van Zoest^3^; FWMN Wit^3^; M Prins^1^; P Reiss^3^; MF Schim van der Loeff^1^; AGEhIV Cohort Study Group

^1^Public Health Service of Amsterdam, Department of Infectious Diseases, Amsterdam, The Netherlands. ^2^Department of Sexology and Psychosomatic Gynaecology, Academic Medical Center, Amsterdam, The Netherlands. ^3^Department of Global Health and Amsterdam Institute for Global Health and Development, Academic Medical Center, Amsterdam, The Netherlands

Presenting author email: mdijkstra@ggd.amsterdam.nl

**Background**: Several studies have reported that HIV-1-infected men who have sex with men (MSM) more often experience sexual dysfunction compared to HIV-1-uninfected MSM. HIV-1-infected individuals have a higher prevalence of non-communicable comorbidities, which may affect sexual health, compared to HIV-1-uninfected individuals. We assessed whether HIV-1 infection is independently associated with decreased sexual functioning among MSM aged 45 years and older.

**Methods**: Data from HIV-1-infected and HIV-1-uninfected MSM aged ≥45 years at the time of enrolment into the ongoing AGE_h_IV Cohort Study were used. The questionnaire included three questions (representing three sexual domains) on sexual functioning from the International Index of Erectile Function (IIEF), addressing erectile function, sexual desire and sexual satisfaction (scale 1–5, higher score represents better function). The three separate questions were dichotomized using a cutoff of ≤2. Three multivariable logistic regression models were constructed to investigate the association between HIV-1 infection and the three domains. Variables associated with both HIV-1 infection and one of the outcomes (at *p* < 0.20) were included in all three multivariable models. We explored HIV-1- and ART-related variables in the established multivariable models including only HIV-1-infected individuals.

**Results**: In total, 399 HIV-1-infected and 366 HIV-1-uninfected MSM were included in the analyses. Decreased erectile function (13.0% vs. 3.4%, *p* < 0.001), decreased sexual desire (7.0% vs. 3.6%, *p* = 0.033) and decreased sexual satisfaction (17.8% vs. 11.8%, *p* = 0.019) were more prevalent among HIV-1-infected MSM compared to HIV-1-uninfected MSM. In multivariable logistic regression models including age, ethnicity, waist-to-hip ratio, non-communicable comorbidities, depression, frailty, use of antidepressants and antihypertensive medication, HIV-1 infection was independently associated with decreased erectile function (adjusted odds ratio (aOR) 2.53, 95% confidence interval (CI) 1.23–5.21), but not with decreased sexual desire (aOR 1.78, 95% CI 0.81–3.92), and decreased sexual satisfaction (aOR 1.35, 95% CI 0.84–2.17). Among HIV-1-infected participants, previous (aOR 3.20, 95% CI 1.52–6.75) and current (aOR 4.71, 95% CI 1.90–11.71) use of lopinavir was independently associated with decreased erectile function.

**Conclusions**: Among MSM aged ≥45 years, being HIV-1-infected was independently associated with decreased erectile function. Exposure to lopinavir appeared to be an independent risk factor for decreased erectile function. No independent association between HIV-1 infection and decreased sexual desire or decreased sexual satisfaction was observed.

## WEAB0105

### SHIV infection and drug transporters influence brain tissue concentrations of efavirenz

N Srinivas ^1^; J Fallon^1^; C Sykes^1^; N White^1^; A Schauer^1^; L Adamson^2^; M Matthews^1^; P Luciw^2^; P Smith^1^ and A Kashuba^1^

^1^University of North Carolina at Chapel Hill, Chapel Hill, USA. ^2^University of California, Davis, Davis, USA

Presenting author email: nithyas@email.unc.edu

**Background**: Despite antiretroviral (ARV) therapy, there is a high prevalence of HIV-associated neurocognitive disorder (HAND) in HIV-infected individuals. Using CSF data, it has been theorized that inadequate ARV concentrations may contribute. However, information on brain tissue concentrations is sparse. This study compared the concentration of ARVs in four regions of brain tissue with CSF in uninfected and SHIV-infected rhesus macaques.

**Methods**: In 12 male macaques (6 uninfected; 6 SHIV-infected) dosed to steady-state condition, concentrations of 6 ARVs - tenofovir (TFV), emtricitabine (FTC), efavirenz (EFV), raltegravir (RAL), maraviroc (MVC) and atazanavir (ATZ) - were measured by LC-MS/MS in the CSF (LLOQ = 0.5 ng/ml) and cerebrum, cerebellum, basal ganglia and parietal cortex regions of the brain (LLOQ of homogenate ranged from 0.002 to 0.01 ng/ml). Tissue concentrations were converted into ng/g using density of 1.06. To assess the influence of drug transporters on ARV concentration, brain tissue was analysed for Pgp and BCRP efflux transporter proteins by LC-MS proteomics (LLOQ = 0.1 pMol/mg protein). Data are presented as median (range); statistical analysis was by Kruskal–Wallis test.

**Results**:

**Abstract WEAB0105–Table 1.** Concentration of ARVs in brain and CSF.

	CSF concentration (ng/ml)		Brain tissue concentration (ng/g)	
	Uninfected	Infected	Uninfected	Infected
TFV	0.8 (0.0, 4.6)	2.2 (1.5, 3.0)	55.0 (47.1, 392.1)	34.9 (22.7, 65.1)
FTC	2.1 (0.0, 11.7)	5.7 (3.9, 7.3)	29.9 (19.5, 85.8)	28.4 (14.8, 33.6)
EFV	2.1 (1.4, 3.4)	0.5 (0.5, 1.4)	1615.2 (965.2, 1983.0)	391.6 (239.8, 792.3)
RAL	1.2 (0.6, 1.3)	0.5 (0.5, 0.5)	27.7 (15.8, 78.3)	14.7 (9.7, 21.8)
MVC	2.9 (0.5, 11.1)	0.0 (0.0, 0.0)	57.5 (21.9, 193.0)	48.7 (34.8, 104.8)
ATZ	0.5 (0.0, 40.5)	0.5 (0.5, 0.5)	84.1 (49.7, 554.1)	133.1 (59.4, 138.0)

CSF concentrations did not differ by infection status (*p* > 0.1). Since there was no difference in ARV concentration in the various regions of the brain (*p* > 0.1), these data were combined. Concentrations in brain tissue were significantly greater than CSF for TFV, FTC and EFV: ranging from 5-times (FTC) to 769-times (EFV) higher. Brain tissue concentration of EFV was 4.1 times higher in uninfected animals. BCRP concentration was 1.7 times higher in infected animals (*p* = 0.02); Pgp concentration did not differ with infection status (*p* = 0.06).

**Conclusions**: In this study, brain tissue concentration of EFV was fourfold lower in infected macaques, and this may be due to increased BCRP concentrations. Further, we have shown that ARV CSF concentrations may need cautious interpretation when used as surrogate for brain tissue exposure. Based on these data, further investigations are needed to determine how ARV brain tissue concentrations influence HAND prevalence.

## WEAC0101

### Barriers to uptake of pre-exposure prophylaxis among respondents to the Flash! PrEP in Europe survey

A Bernier ^1^; RM Delabre^2^; V Schlegel^2^; A Vilotitch^3,4^; S Duken^5^; R Stranz^6^; D Rojas Castro^2,7,8^ and K Jonas^9^

^1^Coalition Internationale Sida, Community-based Research Department, Pantin, France. ^2^AIDES, Community-based Research Department, Pantin, France. ^3^Aix Marseille Univ, INSERM, IRD, SESSTIM, Sciences Economiques & Sociales de la Santé & Traitement de l’Information Médicale, INSERM U912, Marseille, France. ^4^ORS PACA, Observatoire Régional de la Santé Provence-Alpes-Côte d’Azur, Marseille, France. ^5^Psychology Department, University of Amsterdam, Amsterdam, The Netherlands. ^6^Advocacy Department, AIDES, Pantin, France. ^7^University of Lyon 2, Social Psychology Research Group (GRePS), Lyon, France. ^8^Aix Marseille Univ, INSERM, IRD, SESSTIM, Sciences Economiques & Sociales de la Santé & Traitement de l’Information Médicale, Marseille, France. ^9^Work and Social Psychology Department, Maastricht University, Maastricht, The Netherlands

Presenting author email: abernier@coalitionplus.org

**Background**: Pre-exposure prophylaxis (PrEP) has been shown to effectively reduce HIV infection risk and is recommended by the World Health Organization (2015) and the European AIDS Clinical Society (2015). As of January 2017, only two European countries, France and Norway, have authorized prescription and reimbursement of PrEP. In this analysis, we identify potential barriers to PrEP uptake across Europe.

**Methods**: The Flash! PrEP in Europe (FPIE) online survey was a community-based research study aiming to assess interest in and barriers to PrEP uptake amongst respondents from 11 European countries. Data was collected from June to July 2016. Respondents were ≥18 years old and self-reported HIV-negative or unaware of their serological status. A 5-point Likert scale was used to assess potential PrEP uptake barriers, responses were dichotomized (Yes, probably/Yes, definitely vs. Maybe/No, probably/No, definitely). To assess barriers amongst different groups, and due to high response rate from Germany, four groups were analysed separately: German men (GM), other European men (OEM), women and transgender men and women (TMW).

**Results**: Of 15,461 respondents, there were 10,288 GM, 4201 OEM, 690 women, 245 TMW and 37 did not provide gender information. Among the 10,833 (72.7%) respondents potentially interested in PrEP, the greatest potential barriers were fear of side effects (GM: 53.6%, OEM: 39.0%, women: 55.8%, TMW: 40.0%) and necessary hospital visits for PrEP (GM: 49.1%, OEM: 27.3%, women: 33.0%, TMW: 35.7%). Among respondents not interested in PrEP, a majority (>64% in each group) did not want to take PrEP daily, feared side effects or did not feel the need to change their protection strategy. Fear of getting other sexually transmitted infections (STI) was also predominant particularly among GM (72.8%).

**Conclusions**: FPIE results highlight high interest but widespread knowledge gaps in relation to PrEP use among potential users. Improved communication on PrEP, including regular STI testing, follow-up for side effects, possibility of event-driven regimen and facilitating PrEP access, may help address some barriers. Better understanding of PrEP uptake barriers may help inform public health policies which meet the needs of at-risk populations.

## WEAC0102

### Preferences regarding emerging HIV prevention technologies among Toronto men who have sex with men

DHS Tan ^1,2,3^; J Rana^4^; S Fowler^5^; TA Hart^4,6^; J Wilton^7^ and A Bayoumi^2,3,8^

^1^St. Michael’s Hospital, Division of Infectious Diseases, Toronto, Canada. ^2^University of Toronto, Institute of Health Policy, Management and Evaluation, Toronto, Canada. ^3^St. Michael’s Hospital, Centre for Urban Health Solutions, Toronto, Canada. ^4^University of Toronto, Dalla Lana School of Public Health, Toronto, Canada. ^5^Hassle Free Clinic, Toronto, Canada. ^6^Department of Psychology, Ryerson University, Toronto, Canada. ^7^Ontario HIV Treatment Network, Toronto, Canada. ^8^Department of Medicine, St. Michael’s Hospital, Toronto, Canada

Presenting author email: darrell.tan@gmail.com

**Background**: New HIV prevention technologies (NPTs) currently in development include long-acting injectables and topical microbicides and have unique attributes that may appeal differently to different users. We used a discrete choice experiment (DCE), in which participants’ choices between hypothetical alternatives are used to infer preferences for attributes, to characterize NPT preferences among men who have sex with men (MSM) in Toronto, Canada.

**Methods**: MSM undergoing anonymous HIV testing completed a DCE with 12 “choice sets” by selecting their preferred option within each set. Each set included “usual methods to prevent HIV infection” (excluding pre-exposure prophylaxis) as one option and two hypothetical NPT options which differed according to HIV prevention efficacy (50%, 65%, 80% or 99% risk reduction), route of administration, side effects (none or mild) and risk of drug resistance (none, low or moderate). We used mixed logistic regression to infer relative preferences for NPT attributes and latent class analysis to determine patterns of responses.

**Results**: Of 306 participants, 54% were white and median (interquartile range) age was 30 (25, 38). Participants reported 6 (3, 10) partners and 0 (0, 2) condomless receptive anal sex-acts in the preceding six months. Most had heard of post-exposure prophylaxis (80%) and pre-exposure prophylaxis (91%), but only 11% and 5%, respectively, had used them. We excluded 40 participants who had all missing data or gave invariant responses. An on-demand pill was the most preferred NPT, followed by a daily pill, monthly injection and on-demand rectal gel. Resistance was an important determinant of NPT preference if the risk was moderate, but not if low. The minimum NPT efficacy required for an on-demand pill to be preferred over usual methods was 52.8% (95% confidence interval (CI) = 46.9–58.7); for a daily pill, injections and rectal gel, estimates were 60.1% (95% CI = 53.8–66.5), 67.0% (95% CI = 61.0–73.0) and 78.3% (95% CI = 70.9–85.7), respectively. Latent class analysis identified one subset of participants clearly favouring on-demand PrEP (40.5%) and three others preferring usual methods but with an aversion to injections (20.7%), aversion to rectal gels (21.9%) or relative indifference to NPTs (16.9%).

**Conclusions**: Attitudes towards NPTs among MSM are heterogeneous. Understanding these preferences and aversions may help predict NPT uptake.

## WEAC0103

### “Early adopters” of PrEP in SEARCH study in rural Kenya and Uganda

J Ayieko ^1^; C Koss^2^; A Owaraganise^3^; F Mwangwa^3^; D Kwarisiima^3^; D Black^2^; T Clark^2^; M Kaur^2^; J Wallenta^2^; E Charlebois^2^; C Cohen^4^; EA Bukusi^1^; M Kamya^3^; M Petersen^5^ and D Havlir^2^

^1^Kenya Medical Research Institute, Nairobi, Kenya. ^2^University of California, San Francisco, Division of HIV, Infectious Diseases & Global Medicine, San Francisco, USA. ^3^Infectious Diseases Research Collaboration, Kampala, Uganda. ^4^Department of Obstetrics, Gynecology & Reproductive Sciences, University of California, San Francisco, San Francisco, USA. ^5^University of California, Berkeley, Berkeley School of Public Health, Berkeley, USA

Presenting author email: jimayieko@gmail.com

**Background**: PrEP is now recommended for high-risk persons in Africa. There are limited data on PrEP uptake in Africa outside of clinical efficacy trials. “Early adopters” can provide insights for programme strengthening. We report on early PrEP adopters in SEARCH (NCT01864603), an ongoing population-based combination prevention study of 320,000 persons in rural Uganda and Kenya.

**Methods**: Following mobilization and community education, two groups were offered PrEP: (i)HIV-uninfected adults at high risk (R) based an HIV risk score that maximized observed seroconversion coverage under a minimized number of persons needed to treat and (ii)those who perceived themselves at risk (S) including being in serodiscordant relationship. “Early adopters” were defined as those who started PrEP within 30 days of being offered. To estimate predictor coefficients for early PrEP uptake, we used generalized linear models with binomial distribution.

**Results**: Of 24,709 HIV-uninfected individuals in six communities, 4622 were identified for PrEP: 2995 based on risk score (Rs) and 1627 as self-referrals (Ss). A total of 2374 (51%) scheduled an appointment with 946 (20%) initiating PrEP; 916 (97%) of these were “early adopters” with a vast majority 712 (78%) starting PrEP on the same day. “Early adopters” tended to be Ss (64%), women (52%) and married (68%). Youth accounted for only 29% of “early adopters.” Predictors of PrEP uptake among Rs were older age (ref: 18–25, age 36–45, OR 1.6, 95% confidence interval (CI) 1.0–2.5; age 46–55, OR 2.1, 95% CI 1.2–3.9), polygamy (OR 1.9, 95% CI 1.3–2.7), serodiscordant spouse (OR 4.3, 95% CI 1.6–11.5), no history of recent migration (ref: 0 months, 1–6 months, OR 0.6, 95% CI 0.4–1.0; >6months, OR 0.3, 95% CI 0.2–0.7) and perceived current risk of HIV (OR 2.2, 95% CI 1.8–2.8). Among Ss, predictors were gender (male vs. female, OR 0.7, 95% CI 0.6–0.9), age ≥26 (ref: 18–25, age 26–35, OR 1.4, 95% CI 1.1–1.9; age 36–45, OR 1.8, 95% CI 1.4–2.5; age 46–55, OR 2.4, 95% CI 1.7–3.4; age >55, OR 2.0, 95% CI 1.3–3.2) and a serodiscordant spouse (OR 2.5, 95% CI 1.0–6.1).

**Conclusions**: Among the 916 PrEP “early adopters”, most started the same day as offered, two-thirds were married and perceived themselves as high risk. Low participation among certain crucial groups such as youth (18–25 years) emphasizes the need for more effective mobilization.

## WEAC0104

### Health systems and study design features permitting rapid enrolment of individuals at high-risk of HIV acquisition into a pre-exposure prophylaxis study in Melbourne, Victoria, Australia

J Lockwood^1^; J Asselin^2^; A Mak^1^; B Price^1^; D Murphy^3^; L Lal^4^; C El-Hayek^5^; N Roth^6^; J Wilcox^7^; C Fairley^8^; C Chang^9^; BK Tee^10^; M Penn^11^; G Forgan-Smith^12^; S Ruth^13^; P Joffe^14^; C Williams^15^; B Allan^16^; M Stoove^5^; RM Grant^17^; J de Wit^3^ and E Wright ^18^

^1^Alfred Health, Melbourne, Australia. ^2^Burnet Institute, Melbourne, Australia. ^3^Centre for Social Research in Health, University of New South Wales, Sydney, Australia. ^4^Burnet Institute, Alfred Health, Melbourne, Australia. ^5^Burnet Institute, Monash University, Melbourne, Australia. ^6^Prahran Market Clinic, Alfred Health, Melbourne, Australia. ^7^Northside Clinic, Melbourne, Australia. ^8^Melbourne Sexual Health Centre, Alfred Health, Monash University, Melbourne, Australia. ^9^Alfred Health, Monash University, Melbourne, Australia. ^10^Centre Clinic, Victorian AIDS Council, Melbourne, Australia. ^11^PRONTO! Clinic, Victorian AIDS Council, Melbourne, Australia. ^12^ERA Health, Melbourne, Australia. ^13^Victorian AIDS Council, Melbourne, Australia. ^14^PrEPaccessNOW, Melbourne, Australia. ^15^PrEP’D For Change, Melbourne, Australia. ^16^Living Positive Victoria, Melbourne, Australia. ^17^Gladstone Institute for Virology and Immunology, University of California, San Francisco, San Francisco, USA. ^18^Alfred Health, Monash University, Burnet Institute, Melbourne, Australia

Presenting author email: edwina.wright@monash.edu

**Background**: Australia’s Medicare system provides clinicians fee-for-service and residents receive free or low-cost healthcare. Australia’s Pharmaceutical Benefits Scheme (PBS) subsidizes medication costs. Tenofovir/emtricitabine (TDF/FTC) is registered in Australia for HIV pre-exposure prophylaxis (PrEP) but is not PBS subsidized; hence, individuals must import generic TDF/FTC or pay A$800 monthly. In 2016, we implemented a 2600-person PrEP demonstration study in Victoria, hypothesizing a resultant 33% decline in new HIV infections in men who have sex with men (MSM). We describe the health systems and processes that facilitated rapid study enrolment and concomitant increases in HIV and sexually transmitted infection (STI) testing.

**Methods**: Victoria’s population is approximately 6 million, including an estimated 37,000 HIV negative, sexually active MSM. From January 2016, individuals registered study interest online and nominated which of seven study clinics in Melbourne they would attend, whether they already attended that clinic and whether they were currently using PrEP. At study commencement, on 26 July 2016, 2198 individuals had registered interest. Key community stakeholders, study clinics and retail pharmacies were engaged in the study design and service system planning. Clinics were incentivized with A$100/participant or a study nurse. Australian PrEP guidelines specified eligible individuals at high risk. Participants were enrolled electronically. HIV/STI test results were extracted automatically using a sentinel surveillance system (ACCESS), extant in five clinics. We report HIV and syphilis testing rates in three of seven clinics across five-month pre-intervention (26 July 2015–26 December 2015) and PrEP-intervention (26 July 2016–26 December 2016) periods.

**Results**: A total of 1000 participants were enrolled within 21 days of the study commencing, and one in three participants were using PrEP; 2350 participants were enrolled in six months. Six clinics chose the A$100 payment per patient. HIV tests increased from 3009 to 4952, and syphilis tests increased from 2926 to 4704 compared to the same five-month period in 2015, respectively.

**Conclusions**: In a free healthcare system that provides clinicians fee-for-service, rapid enrolment into PrEP programmes appears feasible. A detailed registry of interest, prior use of PrEP, clinic remuneration, electronic enrolment and data extraction and collaborative planning were features of the study’s rapid enrolment rate. A substantial rise in HIV and syphilis testing accompanied the study rollout.

## WEAC0105

### Self-report and medication possession ratio are accurate measures of HIV pre-exposure prophylaxis use in a real-world clinical setting

R Patel ^1^; L Harrison^1^; A Liu^2^; P Chan^3^; R Presti^1^; P Anderson^4^; K Mayer^5^; J Liu^6^; W Powderly^1^ and KR Amico^7^

^1^Washington University in St. Louis, Infectious Diseases, St. Louis, USA. ^2^San Francisco Department of Health, San Francisco, USA. ^3^Brown University, Infectious Diseases, Providence, USA. ^4^University of Colorado Anschutz Medical Campus, Pharmaceutical Sciences, Aurora, USA. ^5^The Fenway Institute, Boston, USA. ^6^Washington University in St. Louis, Public Health Sciences, St. Louis, USA. ^7^University of Michigan, Health Behavior and Health Education, Ann Arbor, USA

Presenting author email: rupapatel@email.wustl.edu

**Background**: Oral, daily pre-exposure prophylaxis (PrEP) prevents HIV acquisition in optimally adherent men who have sex with men (MSM). Given the importance of adherence in PrEP-related outcomes, accurately and affordably monitoring adherence is a priority during implementation. We evaluated two low-burden measurements, self-report (SR) and medication possession ratio (MPR), for concordance with the well-established method of determining tenofovir diphosphate (TFV-DP) levels in dried blood spot (DBS).

**Methods**: We reviewed behavioural and DBS data on patients presenting to the Washington University in St. Louis (USA) PrEP Clinic between November 2015 and August 2016. Optimal adherence was defined as TFV-DP ≥700 fmol/punch and was compared to patient seven-day SR and three-month MPR using pharmacy refill data. Sensitivity, specificity and negative and positive predictive value (NPV, PPV) for SR and MPR in relation to DBS were calculated.

**Results**: From 88 MSM, 137 DBS TFV-DP levels were analysed. Their median age was 27 years; 58% were white, 30% black, 6% Latino; 69% graduated college; and 71% reported condomless receptive anal sex in the last three months. Ten patients had a DBS <700 fmol/punch. Drug concentration was not related to demography and did not significantly decline over time. By SR, five patients took <4 doses/week, four of whom had sub-optimal DBS (NPV 80%), and of the 83 reporting ≥4 doses/week, 77 had optimal DBS (PPV 93%), resulting in 99% sensitivity and 40% specificity. For MPR, three patients had an MPR <0.60 (indicating <4 doses/week), all of whom had sub-optimal DBS (NPV 100%), and of the 84 with MPR ≥0.60, 77 had optimal DBS (PPV 92%), resulting in 100% sensitivity and 30% specificity. MPR and SR correlated with DBS TFV-DP levels (*r* = 0.55, *p* < 0.001; *r* = 0.48, *p* < 0.001). More stringent cutoffs to the strategies produced higher specificity - 60% for SR ≥6 doses/week and 70% for MPR at 0.70.

**Conclusions**: In a real-world clinical setting, SR and MPR correlated with optimal DBS concentrations despite different measurement windows (past 7, 30 and 90 days). Specificity in this sample was improved when more stringent SR and MPR cutoffs were used. Results provide evidence for using low-burden measurements for PrEP adherence monitoring.

## WEAC0201

### Raltegravir vs. lopinavir/r for late-presenter pregnant women

C Brites ^1,2^; I Nobrega^3^; AG Travassos^3^; E Luz^2^; C Stelitano^2^; S Fernandes^3^; C Figueredo^3^; C Lorenzo^3^ and E Martins Netto^1,2^

^1^Universidade Federal da Bahia, Medicine, Salvador, Brazil. ^2^Fundação Bahiana de Infectologia, Clinical Research, Salvador, Brazil. ^3^Centro Especializado em Diagnóstico Assistência e Pesquisa, Salvador, Brazil

Presenting author email: crbritesl@gmail.com

**Background**: Late-presenter pregnant women need aggressive antiretroviral therapy to reach a plasma viral load (PVL) <50 copies/ml before delivering. We compared the safety and efficacy of LPV/r and raltegravir (RAL) in decreasing PVL in late-presenter pregnant women in Salvador, Brazil.

**Methods**: in this open-label, pilot trial (*N* = 40), we included drug-naive pregnant women who started antiretroviral therapy (ART) at a gestational age ≥28 weeks. They were randomly assigned to receive AZT+3TC+LPV/r or AZT+3TC+RAL. We measured time to reach undetectable PVL and compared the proportion of women with PVL <50 copies/ml at delivery, in each group. PVL was measured weekly by real-time PCR, up to delivery. Frequency of side effects and MTCT rate were assessed. Babies were tested for HIV-1 plasma RNA at four weeks of age.

**Results**: we already enrolled 28 women (14/arm). Groups were comparable for age, education, smoking/alcohol use and number of previous gestations/miscarriages. Most (73%) were black/racially mixed and single (82%). Twenty-five women already completed the trial. Median baseline PVL was similar for LPV/r (4.26 log_10_, interquartile range (IQR): 4.02–4.04) and RAL (4.05 log_10_, IQR: 3.55–4.31) groups, as well as mean gestational age (32.8 ± 11.4 vs. 32.9 ± 10.4 weeks, respectively). At delivery, only 1/11 (9%; 95% confidence interval (CI): 0.5–37%) women in group LPV/r had PVL <40 cps/ml, compared with 9/14 (62%; 95% CI:38–86%) in RAL group (*p* = 0.01). The median time to reach undetectable PVL was significantly shorter for RAL (44 days) in comparison with LPV/r arm (69 days, *p* = 0.009). In RAL arm, 2 (14%) patients reached PVL <50 cps/ml at week 1 and 3 (21%) at week 2 of ART. In contrast, in LPV/r arm, the first PVL <50 copies/ml was reached only after six weeks of therapy. More gastrointestinal adverse events were observed in LPV/r arm (5/11) than in RAL one (1/14). No case of MTCT was detected.

**Conclusions**: use of RAL was associated with significantly higher rate of undetectable PVL at delivery and lower incidence of adverse events, in comparison with LPV/r. Time to reach undetectable PVL was significantly shorter in RAL group. RAL should be the preferential option to treat late-presenters pregnant women to minimize the risk of HIV-1 MTCT.

## WEAC0202

### Intensification of antiretroviral treatment with raltegravir for late-presenting HIV-infected pregnant women

N Thepnarong ^1^; T Puthanakit^1^; S Chaithongwongwatthana^2^; S Anugulruengkitt^1^; O Anunsittichai^3^; T Theerawit^3^; C Pancharoen^1^ and P Phanuphak^4^

^1^Research Unit in Pediatric Infectious Diseases and Vaccines, Department of Pediatrics, Faculty of Medicine, Chulalongkorn University, Bangkok, Thailand. ^2^Research Unit in Pediatric Infectious Diseases and Vaccines, Faculty of Medicine, Department of Obstetrics and Gynecology, Faculty of Medicine, Chulalongkorn University, Bangkok, Thailand. ^3^Research Unit in Pediatric Infectious Diseases and Vaccines, Faculty of Medicine, Chulalongkorn University, Bangkok, Thailand. ^4^Thai Red Cross AIDS Research Centre, Bangkok, Thailand

Presenting author email: j_nattawan@hotmail.com

**Background**: The risk of HIV perinatal transmission in HIV-infected pregnant women who are started late on antiretroviral therapy during the third trimester is estimated to be up to 6–10%. Raltegravir, HIV integrase inhibitor, has rapid viral reduction and is recommended by the British HIV Association (BHIVA) guideline for late-presenting HIV-positive pregnant women. This study aims to describe HIV perinatal transmission from high-risk HIV-positive pregnant women who have received raltegravir intensification antiretroviral treatment.

**Methods**: A prospective cohort study was conducted at the Thai Red Cross AIDS Research Centre. Inclusion criteria were HIV-positive pregnant women with high risk of HIV vertical transmission defined as (1) having been started on antiretroviral therapy (ART) at gestational age (GA) >32 weeks or (2) having received ART but having HIV-RNA >1000 copies/ml at GA 32–38 weeks. Pregnant women received standard three-drug ART regimen plus raltegravir 400mg twice daily until delivery and then were continued on three-drug ART after delivery. Plasma HIV-RNA was performed before adding raltegravir and at the time of delivery. The HIV status of infant was determined by HIV-DNA PCR at birth and one, two and four months.

**Results**: From January to December 2016, 57 pregnant women were enrolled. Median CD4 count was 307 cell/mm^3^ (interquartile range (IQR) 155–507). Median plasma HIV-RNA before initiation of raltegravir was 3.6 log_10_copies/ml (IQR 2.9–4.3). Median GA at time of starting raltegravir was 35 weeks (IQR 33–37). Combinations of ART were 32 EFV based (56%), 21 LPV/r based (37%) and 4 others (7%). Median duration of raltegravir was 18 days (IQR 7–28). The proportion of pregnant women who had plasma HIV-RNA <50 and <1000 copies/ml at time of delivery were 47% and 81%, respectively. To date, 50 infants were born, 40% by caesarean section and 8% preterm (GA <37 weeks). HIV perinatal transmission rate was 0% (95% confidence interval 0–6.3). No rash, hepatitis and jaundice in mothers or infants have been reported.

**Conclusions**: No HIV vertical transmission occurred among high-risk HIV pregnant women who received raltegravir intensification ART. This strategy is feasible and effective, supporting elimination of HIV mother-to-child transmission.

## WEAC0203

### Spatial-temporal trend of mother-to-child HIV transmission in western Kenya, 2007–2013

A Waruru ^1^; T Achia^1^; H Muttai^1^; L Ng’ang’a^1^; E Zielinski-Gutierrez^1^; B Ochanda^1^; A Katana^1^; P Young^1^; J Tobias^2^ and T Tylleskär^3^

^1^Centers for Disease Control and Prevention, Division of Global HIV and TB (DGHT), Nairobi, Kenya. ^2^Centers for Disease Control and Prevention, Division of Global HIV and TB (DGHT), Atlanta, USA. ^3^University of Bergen, Bergen, Norway

Presenting author email: awaruru@cdc.gov

**Background**: Using spatial–temporal analyses to understand coverage and trends in elimination of mother-to-child transmission of HIV (e-MTCT) may be helpful in understanding effectiveness of interventions while refocusing e-MTCT programme efforts to the right places to achieve epidemic control. We measured MTCT rates using early infant diagnosis (EID) programme data collected from January 2007 to November 2013 in Western Kenya and assessed associated HIV transmission risk factors within a spatial context irrespective of treatment guideline changes.

**Methods**: We performed trend analysis for 102,116 HIV-exposed infants (HEIs) using extended Cochran–Mantel–Haenszel stratified test and logistic regression models to determine associations of infant HIV status with maternal and infant characteristics recorded on EID laboratory test request forms. We fitted spatial and spatial–temporal semi-parametric Poisson regression models with infant and maternal covariates to explain MTCT rates. We used R-Integrated Nested Laplace Approximation (INLA) package and Quantum GIS to map raw and fitted estimates.

**Results**: Median age of HEIs was two months, interquartile range (IQR) 1.5–6 months. Pooled positivity was 11.8% in the seven-year period, which significantly spatial-temporarily declined from 17.9% in 2007 to 8.4% in 2013, *p* < 0.001 (Figure 1). Uptake of polymerase chain reaction (PCR) HIV testing ≤8 weeks after birth was under 40% in 2007 and increased to 60% by 2013. A spatial–temporal model with covariates was better in explaining geographical variation in MTCT (deviance information criterion (DIC 296)) than either a non-temporal spatial model (DIC 326) or temporal model without covariates (DIC 311).

**Conclusions**: Improved EID uptake and reduced MTCT rates are indicators of success of the e-MTCT programme in this low-resource setting. Adding both time and covariates in spatial–temporal analysis provides a robust approach for explaining programmatic impact over time. Geographical disparities in programme achievements may signify gaps in spatial distribution of e-MTCT efforts and indicate areas needing further resources and intervention.

**Abstract WEAC0203–Figure 1.**
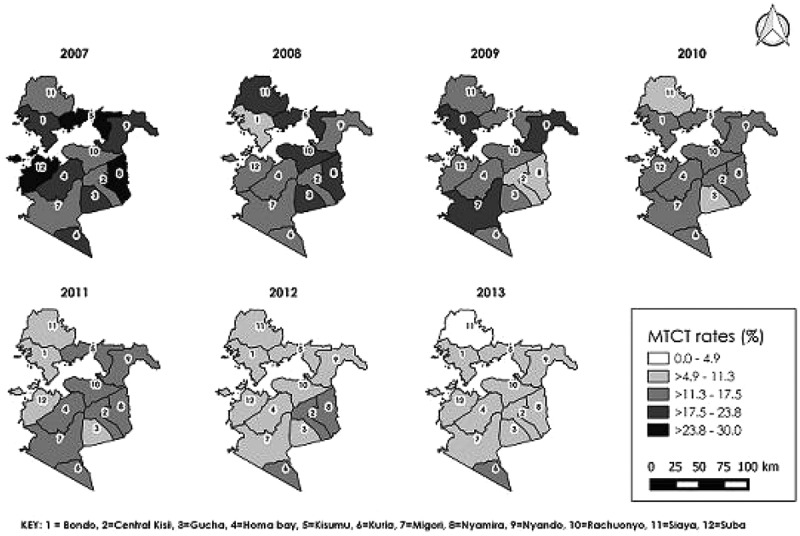
Spatial–temporal trend of fitted MTCT (%).

## WEAC0204

### Cost and cost-effectiveness analysis of a randomized controlled trial evaluating perinatal home visiting among South African mothers/infants

A Wynn^1^; M Tomlinson^2^; MJ Rotheram ^1^ and I Le Roux^3^

^1^University of California, Los Angeles, Psychiatry, Los Angeles, USA. ^2^Stellenbosch University, Psychology, Stellenbosch, South Africa. ^3^Philani Nutrition Centers, Cape Town, South Africa

Presenting author email: mrotheram@mednet.ucla.edu

**Background**: South Africa faces a high antenatal HIV prevalence and infant mortality rate. Community-based programmes involving home visits contribute to reductions in neonatal mortality. However, most low- and middle-income countries lack the budget to deliver such preventive interventions by nurses. Therefore, paraprofessional interventionists may be an innovative alternative strategy to healthcare workers. This study assesses the costs and benefits of implementing a home visiting programme utilizing community health workers (CHWs).

**Methods**: We conducted an economic evaluation alongside a cluster RCT in Cape Town, South Africa, called Philani+. The trial assessed the impacts of training CHWs to deliver antenatal and postnatal home visits to address maternal and child health risks. Financial costs were collected from the perspective of the health system. We calculated incremental cost-effectiveness ratios by dividing the costs of the intervention by the number of low birthweight newborns and cases of infant undernutrition averted among intervention participants compared with controls. These measures are strong indicators of maternal and newborn health. Numbers of averted cases were modelled as the product of intervention subjects and differences in the rates of adverse outcomes between intervention and control groups.

**Results**: The total cost of the intervention over 24 months was estimated at US$91,574. The average cost of supporting 12 CHWs was $7631 per CHW and the cost per mother was US$142. The intervention group had higher HIV treatment adherence and longer breastfeeding duration. The intervention was associated with averting an estimated 55 (90% CI: 41–74) cases of low birthweight and 59 (90% CI: 42–83) cases of undernutrition. The estimated cost per low birthweight averted was $1664, and the estimated cost of averting an undernourished child was US$1552.

**Conclusions**: Philani+ was innovative because it integrated HIV care and prevention with activities to improve maternal and child health. The employment of CHWs provides cost savings compared to use of nurses and builds capacity in a country with a high unemployment rate and shortage of healthcare workers. Finally, Philani+ was able to improve child health at a relatively low cost considering the costs to the health system caused by low birthweight and undernutrition.

## WEAC0205

### A community-based, household survey to determine mother to child HIV transmission rates and HIV-free survival in Swaziland

C Chouraya ^1^; R Machekano^2^; S Mthethwa^3^; L Krysia^4^; M Mirira^5^; K Kudiabor^1^; M Gill^2^; G Maphalala^6^; G Woelk^2^ and L Guay^2^

^1^Elizabeth Glaser Paediatric AIDS Foundation, Mbabane, Swaziland. ^2^Elizabeth Glaser Paediatric AIDS Foundation, Washington, USA. ^3^Ministry of Health, Sexual Reproductive Health Unit, Mbabane, Swaziland. ^4^Department of Epidemiology and Biostatistics, University of California, San Francisco, USA. ^5^United States Agency for International Development, Mbabane, Swaziland. ^6^Ministry of Health, Health Laboratory Services, Mbabane, Swaziland

Presenting author email: cchouraya@pedaids.org

**Background**: The Joint United Nations Programme on HIV/AIDS renewed efforts to virtually eliminate mother-to-child HIV transmission (MTCT) with a target of reducing the mother-to-child transmission rate to 5% or less among breastfeeding populations by breastfeeding cessation and to 2% or less among non-breastfeeding populations. In Swaziland, although data are available on MTCT rates at 6 weeks, no study has been performed to determine MTCT and HIV-free survival through the end of breastfeeding.

**Methods**: The Elizabeth Glaser Pediatric AIDS Foundation performed a national, cross-sectional study of children born 18–24 months prior to the study launch among HIV-infected mothers to determine MTCT rates and HIV-free survival through a community survey in randomly selected constituencies in all four regions of Swaziland. At the time of this cohort’s birth, Swaziland had been implementing World Health Organization Option A for prevention of MTCT (PMTCT). We also evaluated the relationship between both maternal and child characteristics and child infection or death.

**Results**: Most HIV-positive mothers (91.8%) received antiretroviral prophylaxis for PMTCT or antiretroviral treatment during pregnancy. Among 724 known HIV-exposed children between 18 and 24 months, 26 children were HIV-positive and 694 were HIV-negative and alive. Four (all with unknown HIV status at time of death) HIV-exposed children died by 24 months of birth. The overall 18–24-month HIV-free survival among this cohort was 95.9% (95% CI: 94.1–97.2). At 18–24 months, the estimated proportion of HIV-positive children among known HIV-exposed children was 3.6% (95% CI: 2.4–5.2). Older maternal age, delivering in a health facility, high maternal CD4 count and receiving antenatal antiretroviral drugs were associated with reduced risk of child infection or death. Child hospitalization was associated with higher rates of child HIV infection or death.

**Conclusions**: The Swaziland PMTCT programme under Option A was largely effective with a high HIV-free survival of 95.9% and low MTCT at 18–24 months of 3.6%. This would be expected to improve further under current Option B+ (universal maternal antiretroviral therapy).

## WEAD0101

### Trends in pediatric HIV testing across six African countries

T Wolters; E Okoth; A Ahimbisibwe; G Antelman; D Brou Charles-Joseph; M Dlamini; N Nguessan Jean-Paul Kouadio; K Moyo; E Tumbare and R Van de Ven

Elizabeth Glaser Pediatric AIDS Foundation, Washington, USA

Presenting author email: twolters@pedaids.org

**Background**: Improving the identification of HIV-positive children is integral to increasing the number of HIV-positive children on lifesaving treatment; however, there is limited current data on the yield of intensified pediatric HIV case finding approaches. Between July 2015 and September 2016, the Elizabeth Glaser Pediatric AIDS Foundation scaled up intensified pediatric HIV case finding in facility and community-based service delivery points (SDPs) in six countries (Cote d’Ivoire, Kenya, Lesotho, Malawi, Swaziland and Tanzania). Through this initiative, 791,851 children, aged 0–14, were tested.

**Methods**: We conducted a descriptive analysis of aggregate pediatric HIV testing data from the six countries to identify cross-country trends in HIV positivity, including data disaggregated by age group and SDPs, where available.

**Results**: From July 2015 to September 2016, the number of children tested for HIV and identified as HIV positive increased by 275% and 60%, respectively. Total HIV positivity decreased from 2.2% in Q3/2015 to 0.9% in Q3/2016. In Kenya, where SDP disaggregated data is available, outpatient departments (OPDs) represented 63% of children tested and 62% of HIV-positive children identified (0.8% positivity). TB clinics, malnutrition wards and MNCH services had higher positivity (10.2%, 1.9% and 1.4%, respectively). The proportion of HIV-positive children identified in those services was lower than OPD (3%, 2% and 10%, respectively).

**Abstract WEAD0101–Figure 1.**
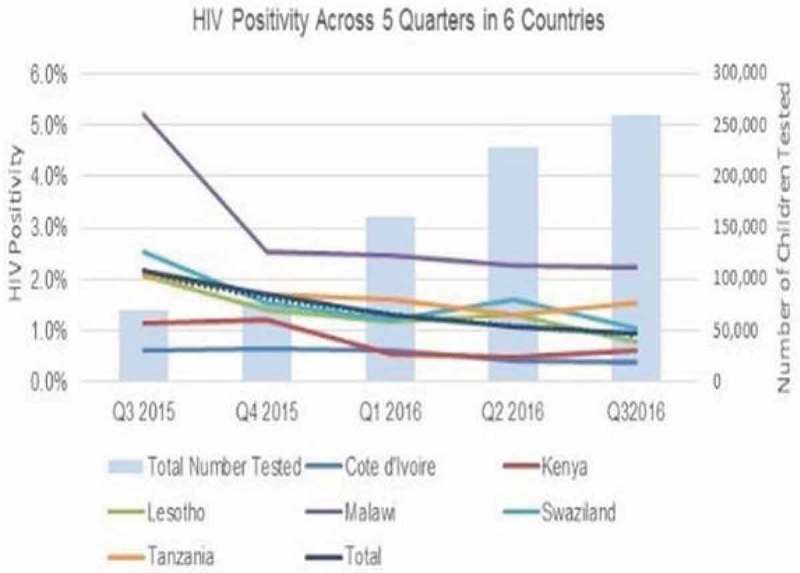
Pediatric HIV positivity in six countries.

**Conclusions**: The rate of HIV positivity in most countries remained relatively stable, although there was large decrease between Q3 2015 and Q4 2015 in Malawi. Intensified pediatric HIV testing dramatically increased the numbers tested and identified 9688 HIV-positive children. Overall HIV positivity was relatively low, likely due to effective prevention of mother-to-child HIV transmission programmes in these countries combined with mortality among undiagnosed older children. Testing of higher-risk children (e.g. TB clinics, malnutrition wards, risk-screening in OPDs) as opposed to broad testing may be a more effective way to identify HIV-positive children in an era of maternal treatment.

## WEAD0102

### Evaluation of the impact of the accelerating children’s HIV/AIDS treatment (ACT) initiative on pediatric and adolescent HIV testing and yield in Western Kenya

NA Okoko ^1^; AR Mocello^2^; J Kadima^1^; JL Kulzer^2^; G Nyanaro^1^; C Blat^2^; M Guze^2^; E Bukusi^1^; CR Cohen^2^; L Abuogi^3^ and S Shade^2^

^1^Kenya Medical Research Institute (KEMRI), Family AIDS Care and Education Services (FACES), Center for Microbiology Research (CMR), Kisumu, Kenya. ^2^Department of Obstetrics, Gynecology and Reproductive Sciences,University of California, San Francisco, USA. ^3^Department of Pediatrics, University of Colorado, Aurora, USA

Presenting author email: awuornicollate@gmail.com

**Background**: Despite declining new infections, pediatric HIV remains significant, with 150,000 new infections annually and 1.8 million children (<15 years old) living with HIV globally. We examined whether activities under the Accelerating Children’s HIV/AIDS Treatment (ACT) initiative increased testing and identification of children with HIV.

**Methods**: Family AIDS Care and Education Services implemented activities under the ACT initiative in 144 health facilities in Western Kenya between October 2015 and September 2016. Interventions targeting pediatric testing included: provision of HIV-testing counsellors; renovation/allocation of space for HIV testing and counselling (HTC space); use of a Family Information Table (FIT) and FIT chart audits; community outreach testing; and text message reminders for HIV-exposed infants. We compared the number of children tested monthly and the number of HIV-positive children between intervention and control sites using negative binomial generalized estimating equations. Analyses adjusted for repeated measures, geographic location, health facility tier and test kit stock-outs.

**Results**: Mean number of children tested monthly increased across all age groups: from 2.8 to 7.2 (*p* < 0.0001) in infants <18 months; from 44.8 to 142.0 (*p* < 0.0001) in children 18 months to 9 years; and from 30.1 to 123.3 (*p* < 0.0001) in adolescents 10–14 years. Identification of HIV-positive children increased: 0.06 to 0.37 (per month per facility; *p* < 0.0001) in infants; 0.34 to 0.62 (*p* = 0.002) in children; and 0.17 to 0.26 (*p* = 0.03) in adolescents. Use of the FIT was significantly associated with increased HIV testing in infants, incidence rate ratio (IRR) = 2.89 (95% confidence interval (CI) = 1.53–5.49; *p* < 0.001) and identification of HIV-positive infants, IRR = 8.71 (95% CI = 1.45–52.4; *p* < 0.02). Among children, FIT chart audits were significantly associated with increased testing, IRR = 2.15 (95% CI = 1.36–3.40; *p* < 0.001). Among adolescents, HTC space was significantly associated with increased HIV testing, IRR = 1.45 (95% CI = 1.09–1.93; *p* < 0.01).

**Conclusions**: Targeted testing of family members of HIV-positive adults increased both testing and identification of HIV-positive children. Our findings suggest that the one-time investment in improving HTC space may be an effective approach for increasing HIV testing among adolescents in this context. Significant increases in number of children tested resulted in only a modest number of new children identified with HIV, highlighting the need for multiple testing approaches.

## WEAD0103

### Disclosure of HIV status to children living with HIV in Malawi: needs assessment and formative evaluation of an intervention intended to help with the disclosure process

F Kalembo ^1,2^; GE Kendall^2^ and M Ali^2^

^1^Mzuzu University, Mzuzu, Malawi. ^2^Curtin University, Nursing and Midwifery, Perth, Australia

Presenting author email: kalembofatch@yahoo.com

**Background**: Approximately 10% of people living with HIV in Malawi are children under the age of 15 years. While the World Health Organisation recommends that disclosure of HIV status should take place between the ages of 6 and 12 years, very little is known about the practice of HIV disclosure in Malawi. This study aimed to evaluate the current practice of HIV disclosure to Malawian children and to assess the acceptability of a series of age-appropriate, culturally acceptable story books intended to help with the disclosure process.

**Methods**: Questionnaires, interviews and focus group discussions were used to collect data from caregivers, healthcare workers, school teachers, adolescents living with HIV and community leaders across the three administrative regions of Malawi. Data on disclosure of HIV to the child, reasons for non-disclosure, the need and acceptability of the proposed series of story books, the child’s mental health and the family psychosocial characteristics were collected using reliable instruments. Data were analysed using chi-square test, multiple logistic regression and thematic analysis.

**Results**: The response rate was 99%: 600 questionnaires, 19 interviews and 12 focus groups were completed. The prevalence of non-disclosure was 64%. Non-disclosure of HIV status was more likely for younger children (aOR 3.8; 95% CI: 2.1–6.8), those in a farming family (aOR 3.4; 95% CI: 1.2–9.3) and those whose healthcare workers lacked training about disclosure (aOR 7.7; 95% CI: 3.4–11.6). The lack of disclosure guidelines and materials (33%), the child’s capacity to cope with the diagnosis (29%) and a lack of confidence to disclose appropriately (19%) were cited as the main reasons for non-disclosure. Ninety-eight per cent of participants supported the idea of developing the proposed series of story books. More than three-quarters of the participants emphasized the need for all stakeholders involved in caring for children living with HIV to work together towards promoting effective HIV disclosure.

**Conclusions**: The rate of non-disclosure in Malawi is high. The results of this study support the need for the development and rigorous evaluation of disclosure materials and the involvement of all stakeholders to promote effective disclosure and meet the evolving needs of children.

## WEAD0104

### An assessment of the effectiveness of reaching undiagnosed HIV-positive children through community-based testing in Lesotho

K Sindelar ^1^ and J Joseph^2^

^1^Clinton Health Access Initiative, Lesotho – Pediatric HIV, Maseru, Lesotho. ^2^Clinton Health Access Initiative, Applied Analytics, Denver, USA

Presenting author email: ksindelar@clintonhealthaccess.org

**Background**: All health facilities in Lesotho offer free, provider-initiated HIV testing and counselling (PITC) and treatment, and the country adopted the WHO-recommended “Treat All” policy in 2016. However, the antiretroviral treatment coverage for children, 0–14 years, is less than 60%, suggesting many children are not regularly accessing services at facilities. Recognizing that testing is the critical first step in initiating HIV-positive children to lifesaving treatment, community-based PITC strategies were piloted to understand the effectiveness of identifying undiagnosed children in Lesotho beyond the facility.

**Methods**: From December 2015 to December 2016, four community-based strategies were utilized: (1) mobile outreach clinics; (2) door-to-door testing; (3) household-based index patient testing; and (4) targeted testing events conducted at venues thought to have high-risk children. A mobile application was designed for use by healthcare workers for real-time data collection at point-of-care to capture data on newly identified HIV-positive children; this included residence, gender and age, as well as HIV testing history and health facility attendance history.

**Results**: Out of 36,121 children tested, 161 were HIV positive (0.44% positivity yield), and 123 were enrolled in the programme with a 1:1.2 male-to-female ratio. Only 23.5% of all enrolled patients were known to be previously tested. Overall, 12% were 0–2 years, 25% were 2–5 years and 63% were 5–14 years. Of the 52% of patients with known facility attendance history, 20% had not been in over one year, 17% had attended within four months to one year and 15% had visited a facility within the previous three months.

**Conclusions**: The majority of children testing HIV positive through community-based PITC were 5–14 years, nearly half had unknown facility attendance history and 9.5% who had been to a facility in the previous year did not receive an HIV test. This establishes the need for investigation into causes of Lesotho’s shortcomings in offering facility-based PITC. Additionally, it proves that community-based PITC is a necessary tool in closing the access gap by bringing these lifesaving services to children in need, particularly older children. National scale-up is essential in reaching UNAIDS’ first 90 target and is recommended for other countries struggling to achieve widespread coverage for children through facility-based testing.

## WEAD0105

### The clinical impact and cost-effectiveness of incorporating point-of-care (POC) assays into early infant HIV diagnosis (EID) programmes at 6 weeks of age in Zimbabwe: a model-based analysis

SC Frank ^1,2^; J Cohn^3,4^; L Dunning^5^; E Sacks^6^; RP Walensky^1,2,7^; S Mukherjee^6^; E Turunga^3^; KA Freedberg^1,2,7^ and AL Ciaranello^1,2^

^1^Massachusetts General Hospital, Medical Practice Evaluation Center, Boston, USA. ^2^Massachusetts General Hospital, Division of General Medicine, Boston, USA. ^3^Elizabeth Glaser Pediatric AIDS Foundation, Geneva, Switzerland. ^4^University of Pennsylvania, Division of Infectious Disease, Philadelphia, USA. ^5^University of Cape Town, Division of Epidemiology and Biostatistics, School of Public Health & Family Medicine, Cape Town, South Africa. ^6^Elizabeth Glaser Pediatric AIDS Foundation, Washington, USA. ^7^Massachusetts General Hospital, Division of Infectious Disease, Boston, USA

Presenting author email: scfrank@mgh.harvard.edu

**Background**: Many EID programmes use laboratory-based total nucleic acid (conventional) assays. New POC EID assays are costlier, but may increase access to testing and shorten time to result-return and ART initiation.

**Methods**: We used the CEPAC-Pediatric model to examine the clinical benefits, costs and cost-effectiveness of using POC EID assays at 6 weeks of age in Zimbabwe. We simulated two EID strategies: conventional and POC. Positive results led to ART initiation; ART was stopped if a confirmatory assay of the same type and a third conventional assay (all sent pre-ART) were negative. Modelled assays differed in sensitivity (conventional: 100%; POC: 96.9%), specificity (conventional: 98.8%; POC: 100%), time and probability of result-return (conventional: one-month delay, 71%; POC: immediate, 97%) and cost (conventional: $15; POC: $21). Model outcomes included early survival, life expectancy (LE), and average lifetime per-person cost for HIV-infected infants and all HIV-exposed infants. We calculated incremental cost-effectiveness ratios (ICERs) using discounted (3%/year) costs and LE for all HIV-exposed infants, defining ICERs ≤$930/life-year saved (Zimbabwe *per-capita* GDP) as cost-effective.

**Results**: With conventional EID, projected undiscounted LE was 24.95 years (HIV-infected infants) and 60.16 years (all HIV-exposed infants), at $1050/HIV-exposed infant (Table 1). POC EID improved projected undiscounted LE (HIV-infected: 26.58 years, HIV-exposed: 60.27 years) at $1120/infant, and increased survival by 4.5% in months 1–2 of life. The ICER of POC vs. conventional was $730/life-year saved (LYS). This ICER remained <$930/LYS if POC specificity was >95% or POC sensitivity was >85%. Large improvements in conventional assay result-return were needed to offset slightly lower POC assay sensitivity (Figure 1).

**Abstract WEAD0105 – Table 1.** POC and conventional EID: modelled outcomes.

Strategy for EID testing at 6 weeks of age in Zimbabwe	Outcomes for HIV-infected infants	Outcomes for all HIV-exposed infants (including HIV-infected and HIV-uninfected infants)				
	LE (years, undiscounted)	LE (years, undiscounted)	LE (years, discounted)	Lifetime per-person costs (USD, undiscounted)	Lifetime per-person costs (USD, discounted)	Incremental cost-effectiveness ratio ($/life-year saved)
Conventional	24.95	60.16	24.84	$1050	$570	Comparator
POC	26.58	60.27	24.91	$1120	$620	$730

**Abstract WEAD0105 – Figure 1.**
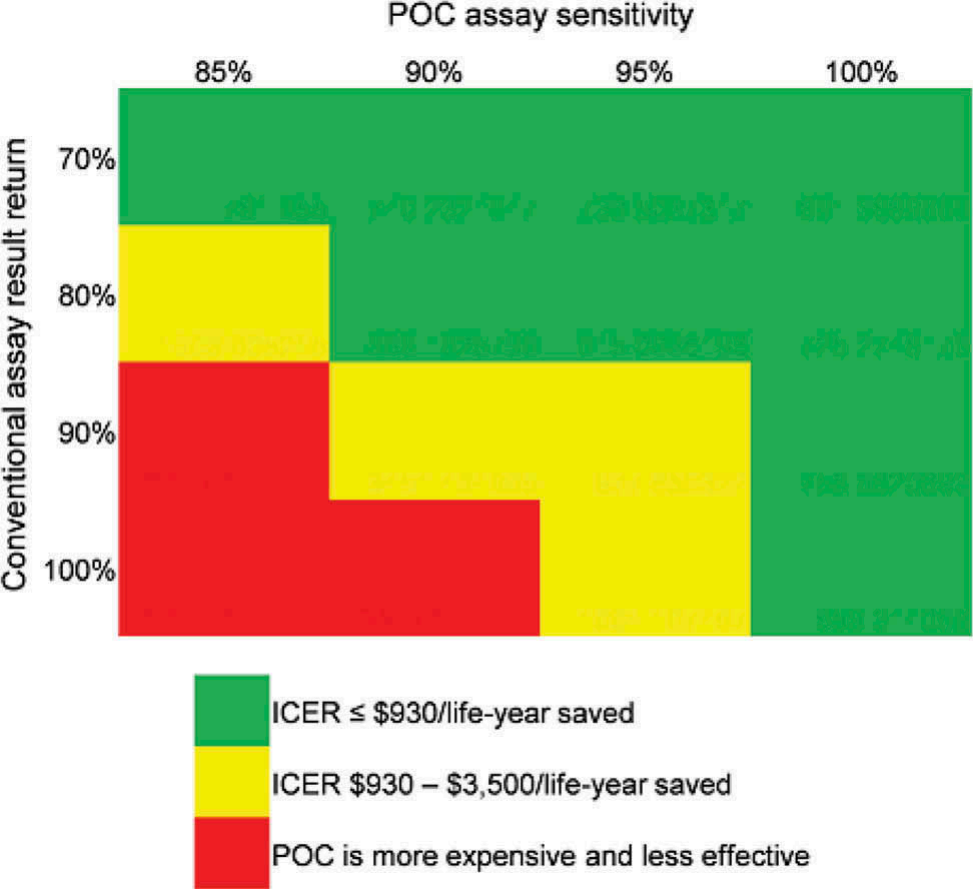
Sensitivity analysis: ICER of point-of-care (POC) compared to conventional EID.

**Conclusions**: POC assays for HIV-exposed infants improve survival and LE and are cost-effective compared to conventional assays. EID programmes in Zimbabwe should replace conventional testing with POC assays.

## WEAD0201

### When donor funding leaves: the immediate impact on resources of USAID’s withdrawal of support for direct HIV care and treatment at a public health facility in South Africa

N Lince-Deroche; R Mohamed; S Kgowedi and L Long

Health Economics and Epidemiology Research Office, Johannesburg, South Africa

Presenting author email: nlince-deroche@heroza.org

**Background**: From 2008 to 2014, USAID-funded organizations were responsible for directly delivering HIV care and treatment via “Comprehensive Care, Management and Treatment” facilities (CCMTs) in South Africa. Despite USAID having communicated its plans to phase out funding for direct service delivery (DSD) and instead focus primarily on technical assistance, there has been a sense that public clinics were not adequately prepared for the transition. The aim of this study was to examine the impact on financial and human resources and workloads immediately after the withdrawal of funds for a CCMT at a clinic in Johannesburg.

**Methods**: In late 2016, we conducted a natural experiment in which trends in budgets and expenditure, clinical staff complements, patient loads and services rendered were compared at the study clinic before (2007–2012), during (2012–2014) and after (2014–2016) the withdrawal of the clinic’s USAID-supported CCMT site. Data were drawn from the country’s District Health Information System, local budget and expenditure reports, and staff records supplied by the city. Analysis was conducted in Excel (2013).

**Results**: Phasing out of the CCMT occurred between July 2012 and June 2015. Reductions in clinic staff occurred in parallel, first by 33% in 2012–2013 and then by a further 29% in 2014–2015. The reduction in staff drastically raised the workload per staff member (i.e. patient-to-staff headcount ratio) in 2013 and 2015. The withdrawal of the CCMT was not accompanied by an increase in real expenditure. Real expenditure per capita was on average lower after 2012 (ZAR 77) than it had been between 2008 and 2012 (ZAR 85) when the CCMT was providing HIV treatment to patients. Despite increased workload, service volumes for primary healthcare services at the clinic (HIV counselling and testing, tuberculosis testing and family planning) did not decrease after the departure of the CCMT.

**Conclusions**: The phasing out of funding for DSD by USAID at a clinic in Johannesburg negatively impacted on human resources and staff workload while decreasing per capita expenditure. The volume of primary healthcare services delivered at the clinic did not decline; however, the impact on service quality is unknown.

## WEAD0202

### How changes in United States funding policies could impact the HIV epidemic in sub-Saharan Africa

J McGillen ^1^; A Sharp^2^; B Honermann^2^; G Millett^2^; C Collins^3^ and T Hallett^1^

^1^Department of Infectious Disease Epidemiology, Imperial College London, London, UK. ^2^amfAR, The Foundation for AIDS Research, Washington DC, USA. ^3^Friends of the Global Fight Against AIDS, Tuberculosis, and Malaria, Washington, DC, USA

Presenting author email: j.mcgillen@imperial.ac.uk

**Background**: The United States is a giant in the global fight against HIV/AIDS. The bipartisan foundation of PEPFAR in 2003 transformed the world’s approach to antiretroviral therapy in developing countries, and the US is the largest contributor to the Global Fund. However, the isolationism of the Trump Administration may soon place these activities under threat.

**Methods**: To test the potential impact of changes in American HIV/AIDS funding policies, we employed a mathematical model of the HIV epidemic and response across 18 countries in sub-Saharan Africa. We used financing data from PEPFAR, the Global Fund and the IMF to estimate the US share of the total HIV/AIDS response historically and in future. We then removed this US share from the total funding available, or changed the way it could be allocated to future prevention efforts, to explore a series of alternative policy strategies that the administration might adopt.

**Results**: The model finds that US participation in the AIDS response is likely to have averted 2.5 million AIDS deaths and 21 million HIV infections in sub-Saharan Africa between the start of the epidemic and the end of 2016. Looking forward, sustained US funding could avert 300,000 AIDS deaths and 8.4 million HIV infections on the subcontinent between now and 2030. If the US instead withdraws from the funding landscape, for example by defunding PEPFAR in 2017 and breaking its pledge to the Global Fund, the cost could reach 298,000 AIDS deaths and 7.9 million HIV infections by 2030. How funds are disbursed also matters. If the new administration continues to fund PEPFAR but turns a moralistic blind eye to sex workers and gay men, an avoidable 239,000 AIDS deaths and 5.4 million HIV infections could occur.

**Conclusions**: Our work suggests that the choice before the US government is stark: it can shirk the mantle of global leadership in the AIDS response and thereby reverse the past decade of progress against the epidemic, or it can continue to fund PEPFAR and the Global Fund and potentially save 8.4 million people across sub-Saharan Africa from infection with HIV.

## WEAD0203

### Estimating the size of the pediatric antiretroviral (ARV) market in 26 low- and middle-income countries (LMICs) through 2025 as prevention of mother to child transmission (PMTCT) initiatives continue to succeed

VR Prabhu ^1^; S Mcgovern^1^ and P Domanico^2^

^1^Clinton Health Access Initiative, HIV Access Program, Boston, USA. ^2^Clinton Health Access Initiative (CHAI), Boston, USA

Presenting author email: vprabhu@clintonhealthaccess.org

**Background**: PMTCT initiatives have significantly reduced HIV infections in children, a trend expected to accelerate with the “Start Free, Stay Free, AIDS Free” initiative. Its “Super Fast-Track” targets aim, by 2020, to reduce new perinatal infections to <20,000 annually and achieve universal pediatric ART coverage. Population projections are needed to help optimize supply of pediatric ARVs, especially new formulations.

**Methods**: Provisional single age band estimates for HIV-infected children (ages 0–14) for 26 high-burden LMICs, provided by UNAIDS, representing approximately 77% of the global pediatric HIV burden in 2015, were used. For each forecast year, age cohorts were moved from one age bracket to the next, new perinatal HIV infections were added to the age 0 cohort and the age 14 cohort “aged out” of the market. Annual decreases in AIDS-related deaths were assumed to be similar to 2011–2015, and evenly distributed across age groups.

Three scenarios of the “Super Fast-Track” targets for new infections were modelled: “Aggressive” (targets met), “Moderate” (two-year delay) and “Conservative” (similar decrease as 2011–2015). Resulting cohorts were converted to weight bands using published tables.

**Results**: The number of HIV-infected children will continue to rise until 2019 before decreasing. By 2025, there could be between 350,000 and 500,000 children living with HIV, needing ART, across the 26 high volume countries. The more aggressive scenarios suggest an increasing proportion of >20 kg children.

**Abstract WEAD0203 – Figure 1.**
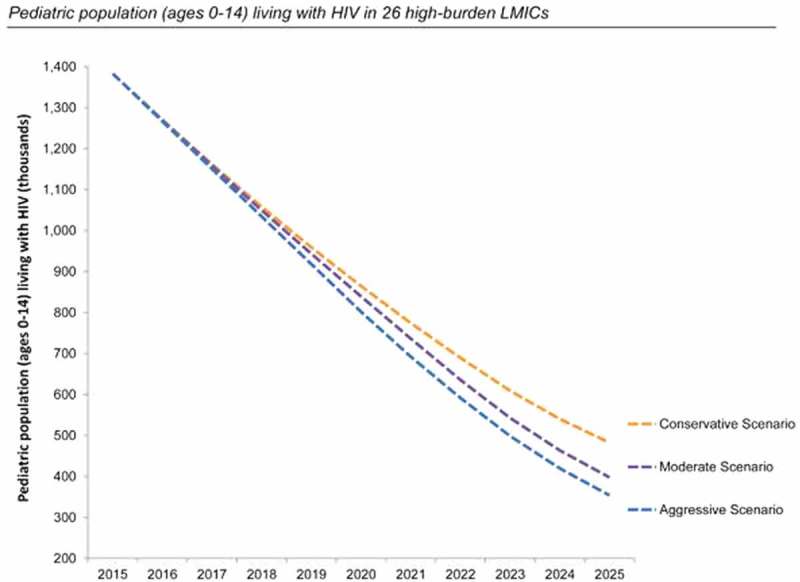
Total ped. PLWHA.

**Abstract WEAD0203 – Figure 2.**
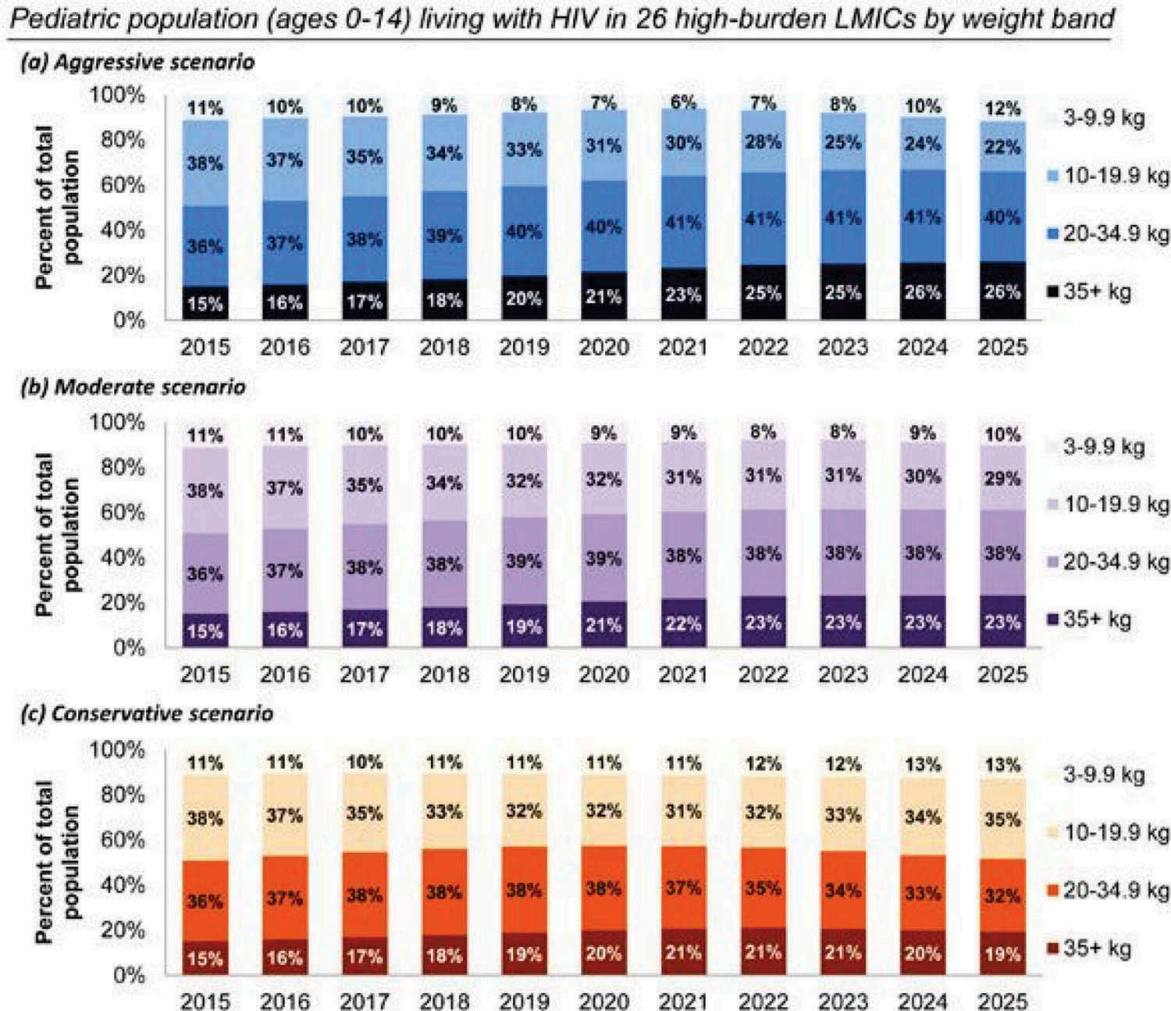
Weight distribution.

**Conclusions**: As pediatric ART coverage increases, more children will need second- or third-line treatment. These population estimates can inform discussions on the development of new pediatric ARV formulations.

## WEAD0204

### Can differentiated care models solve the crisis in treatment financing? Analysis of prospects for 38 high-burden countries in sub-Saharan Africa

C Barker; A Dutta and K Klein

Palladium, Washington, USA

Presenting author email: arin.dutta@thepalladiumgroup.com

**Background**: Rapid scale-up of antiretroviral therapy (ART) in the context of financial and health system constraints has resulted in calls to maximize efficiency in ART service delivery. Adopting differentiated care models (DCMs) for ART could potentially be more cost-efficient and improve outcomes. However, no study comprehensively projects the cost savings across countries. We model the potential reduction in facility-level costs and number of health workers needed when implementing two types of DCMs while attempting to reach 90-90-90 targets in 38 sub-Saharan African countries from 2016 to 2020.

**Methods**: We estimated the costs of three service delivery models: (1) undifferentiated care, (2) differentiated care by patient age and stability and (3) differentiated care by patient age, stability, key versus general population status, and urban versus rural location. Frequency of facility visits, type and frequency of laboratory testing, and coverage of community ART support varied by patient subgroup. For each model, we estimated the total costs of antiretroviral drugs, laboratory commodities and facility-level personnel and overhead. Community-based ART costs were included in the DCMs. We took into account underlying uncertainty in the projected numbers on ART and unit costs.

**Results**: Total five-year facility-based ART costs for undifferentiated care are estimated to be US$23.33 billion (95% confidence interval (CI) $23.3–$23.5 billion). An estimated 17.5% (95% CI 17.4–17.7) and 16.8% (95% CI 16.7–17.0) could be saved from 2016 to 2020 from implementing the age and stability DCM and four-criteria DCM, respectively, with annual cost savings increasing over time. DCMs decrease the full-time equivalent (FTE) health workforce requirements for ART. An estimated 46.4% (95% CI 46.1–46.7) fewer FTE health workers are needed in 2020 for the age and stability DCM compared with undifferentiated care.

**Conclusions**: Adopting DCMs can result in significant efficiency gains in terms of reduced costs and health workforce needs, even with the costs of scaling up community-based ART support under DCMs. Efficiency gains remained relatively unchanged with increased differentiation due to some groups requiring more intensive inputs. More evidence is needed on how to translate analysed efficiency gains into implemented cost reductions at the facility level.

## WEAD0205

### Characterizing the South African private sector ART market

H Awsumb; K Little; P Aylward and N Hasen

Population Services International, Washington, USA

Presenting author email: hawsumb@psi.org

**Background**: South Africa has the largest public sector HIV treatment programme in the world, with >3 million clients on ART. It also has one of the most robust and diverse private healthcare sectors in Africa. We hypothesized that a substantial segment of South African PLHIV are accessing ART through the private sector, especially for 2nd line (2L) treatment regimens. We characterized the private sector ART market indirectly by comparing public and private sector drug procurement data from 2012 to 2015.

**Methods**: For the private sector, we analysed IMS Health data from pharmaceutical wholesalers and distributors in South Africa. For the public sector, we analysed data from the Global Price Reporting Mechanism. To avoid double counting, we used regimens with efavirenz or nevirapine as a proxy for 1st line (1L) regimens and regimens with atazanavir or lopinavir/rotanivir as a proxy for 2L regimens.

**Results**: The total patient years of treatment (PYTs) for 1L and 2L regimens in the private sector ART market peaked at 244,760 in 2014 and then decreased to 231,938 PYTs in 2015. Total public sector PYTs for 1L and 2L regimens in South Africa increased more than seven-fold during 2012–2015, from 544k to 4.3 million PYTs. Rapid growth in public sector ARV procurement did not appear to significantly displace ARV purchasing in the private sector. Growth in the private sector 1L treatment decreased only 4.2% on average from 2013 to 2015 while the number of PYTs for 2L treatment in the private sector increased 3.9% on average for the same time period.

**Conclusions**: Our analysis found a substantial private sector market for ART in South Africa. By 2015 PYTs, the private sector ART market in South Africa would have been the 14th largest treatment programme in the world and the 10th largest in Africa. As public sector treatment budgets reach capacity, there may be opportunities to leverage domestic financing through private sector channels in South Africa. Better landscaping of the private sector ART market is needed to guide future interventions and investments.

## POSTER DISCUSSION ABSTRACTS

## MOPDA0101

### A higher fraction of drug-resistant proviruses express unspliced HIV RNA during ART compared to the archival wild-type proviruses that comprise the HIV-1 reservoir

A Musick^1^; J Spindler^1^; M Sobolewski^2^; M Bale^1^; B Keele^3^; W Shao^3^; A Wiegand^1^; S Hughes^1^; J Mellors^2^; J Coffin^4^; F Maldarelli^1^ and M Kearney ^1^

^1^National Cancer Institute, Frederick, USA. ^2^University of Pittsburgh, Pittsburgh, USA. ^3^Lei, Frederick, USA. ^4^Tufts University, Boston, USA

Presenting author email: kearneym@mail.nih.gov

**Background**: The fraction of proviruses persisting during ART that are latent vs. transcriptionally active has not been determined. To address this question, we investigated the expression of unspliced HIV RNA *in vivo* in single cells carrying either wild-type (WT) proviruses or those with drug resistance (DR) mutations.

**Methods**: Peripheral blood mononuclear cells (PBMCs) were analysed from Patient #1 in Maldarelli et al. (2014). The fraction of the proviruses expressing HIV RNA was determined by single-genome sequencing of cell-associated HIV RNA and DNA from single cells. Intact proviruses, capable of infectious virus production, were identified using viral outgrowth assays (VOAs). The levels of viral RNA present in infected cells were determined for the archival drug-sensitive population, the recently infected DR population and for clones carrying intact and defective proviruses.

**Results**: We analysed a total of 77 million PBMC, of which 10,450 contained HIV *pro-pol* sequences: 7137 were WT, 1714 were DR and 1599 were defective (contained stop codons). The median fraction of proviruses that expressed RNA in cells more recently infected with DR virus was 25%, whereas in cells with WT or hypermutant proviruses, it was 14% (*p* = 0.0008). Levels of expression in single cells with DR proviruses were higher than in cells with WT proviruses (*p* = 0.002). The median fraction of cells in apparent clonal populations carrying intact proviruses (*N* = 3) expressing HIV RNA was 2.3% (1.2–8.8%). For clones carrying defective proviruses (*N* = 5), the median fraction expressing was 3.5% (0.9–7.0%), and for clones carrying proviruses that did not have obvious defects in the *pro-pol* region but were not recovered in the VOA (*N* = 26), the median was 6.6% (1.3–66.7%).

**Conclusions**: A small fraction of the proviruses in clones of HIV-infected cells expressed HIV RNA. The fraction and levels of proviral expression were significantly higher in more recently infected cells than in those that persisted during long-term ART. These findings show that ART can select both for cells infected before ART initiation that either do not express HIV RNA or express at low levels and for cells infected recently with drug-resistant viruses that express higher levels of HIV RNA.

## MOPDA0102

### Evidence of production of HIV-1 proteins from “defective” HIV-1 proviruses in vivo: implication for persistent immune activation and HIV-1 pathogenesis

H Imamichi ^1^; M Smith^1^; A Pau^1^; CA Rehm^1^; M Catalfamo^1,2^ and HC Lane^1^

^1^NIAID, NIH, Laboratory of Immunoregulation, Bethesda, USA. ^2^Department of Microbiology and Immunology, Georgetown University School of Medicine, Washington, USA

Presenting author email: himamichi@nih.gov

**Background**: Greater than 95% of proviruses detected in circulating peripheral blood mononuclear cells are referred to as “defective” and are unable to encode intact viruses. They have been thought to represent a silent graveyard of viruses with little contribution to HIV-1 pathogenesis. We have recently shown that these “defective” proviruses are capable of transcribing novel mRNA species. In the present study, we demonstrate that these “defective” proviruses are also capable of producing HIV-1 proteins.

**Methods**: CD4+ T cell clones were obtained from an HIV-infected individual who had recently been placed on suppressive combination antiretroviral therapy (cART). The CD4+ T cells were initially plated in 96-well cell culture plates at a cell density of 100 cells/well and expanded for 2 weeks in the presence of autologous feeder cells. Positive wells by HIV-DNA PCR were further expanded in 48-well plates for another week. The identification of single-cell clones harbouring “defective” proviral DNA was confirmed by combining 5´LTR-to-3´LTR single-genome amplification and direct amplicon sequencing of the genomic DNA. Cellular expression of HIV-1 proteins was analysed by western blot and flow cytometry.

**Results**: Two months after suppression of plasma viremia to <40 copies/ml, the estimated frequency of CD4+ T cells containing HIV-DNA was 1%. Multiple cell lines harbouring defective proviruses ranging from 6.5 to 8.2 kb in length were derived from the CD4+ T cells. Most prominent among these were cells containing an identical 6.5 kb provirus. Sequencing of this 6.5 kb provirus revealed a 2.4 kb internal deletion affecting the region encoding the HIV-1 accessary proteins and the gp120 portion of Env protein. The Gag, Pol and Nef regions remained intact in the 6.5 kb provirus. Consistent with the DNA data, western blots revealed the presence of the Gag and Nef proteins.

**Conclusions**: These data indicate that “defective” proviruses in successfully treated HIV-infected patients are not silent dead-end products but rather capable of producing HIV-1 proteins in vivo. The proteins encoded by these defective “zombie” proviruses may be responsible for persistent seropositivity and immune activation in most patients with controlled HIV-1 infection during suppressive cART.

## MOPDA0103

### HIV reservoirs in the brain and association with sex and neurocognition

MF Oliveira^1^; M Nakazawa^1^; A Vitomirov^1^; M Zhao^1^; B Gouaux^1^; D Moore^1^; R Ellis^1^; D Smith ^1,2^ and S Gianella^1^

^1^University of California San Diego, La Jolla, USA. ^2^Veterans Affairs San Diego Healthcare System, La Jolla, USA

Presenting author email: d13smith@ucsd.edu

**Background**: Although antiretroviral therapy (ART) reduces plasma HIV-RNA below the detection limit, HIV reservoirs persist in anatomic compartments, as the central nervous system. The clinical and biological factors that influence HIV reservoirs in brain are unknown.

**Methods**: Paired autopsy tissues from frontal cortex (FC, *N* = 61), occipital cortex (OCC, *N* = 60), basal ganglia (BG, *N* = 31) and peripheral lymphoid tissue (*N* = 37) from 63 HIV-positive adults were selected from the National NeuroAIDS Tissue Consortium. All participants died with virologic suppression on ART (<50 or 400 copies/ml, assay-dependent) without evidence of CNS opportunistic disease, between 1999 and 2014. Genomic DNA was extracted by magnetic beads; HIV-DNA levels were measured by ddPCR and normalized by *RPP30*. Neurocognitive (NC) functioning was assessed at the last visit (median three months before death) by Clinical Rating based on seven neuropsychological abilities. Bayesian hierarchical regression model was used to evaluate the relationship between brain regions, sex and NC functioning. The model used a zero-inflated negative-binomial family with a logit link function.

**Results**: The study cohort consists of 12 females and 51 males (median age: 55 years). Median CD4+ at the last visit was 164 (IQR: 80–390), and median estimated duration of infection was 14 years (IQR: 10–19). HIV-DNA was detected in 62.5% of brain and 100% of lymphoid tissue. Lymphoid tissue has higher HIV-DNA levels than brain (85.6 vs. 14.2, *p* < 0.001). BG has higher HIV-DNA levels (20.3 copies/10^6^ cells) compared to FC (13, *p* = 0.018) and OCC (9.3, *p* = 0.005). Female sex and younger age are associated with higher HIV-DNA in brain (*p* = 0.026 and *p* = 0.06, respectively), but not in lymphoid tissue (*p* = 0.31). When evaluating NC sub-domains, higher HIV-DNA (any brain region) was associated with worse speed of information processing (*p* = 0.012) and better verbal fluency (*p* < 0.05). No sex differences in Clinical Rating were observed.

**Conclusions**: HIV-DNA was detected in most of brains despite virologic suppression. While levels of HIV-DNA were comparable in lymphoid tissue, women had higher brain HIV-DNA levels than men. Higher brain HIV-DNA levels negatively affected the speed of information in both sexes. The negative association between HIV-DNA and verbal fluency requires more investigation.

## MOPDA0104

### Monocyte-derived reactive oxygen species impair CD4+ T cell restoration in HIV-1 patients under therapy

M Younas^1^; Y-L Lin^1^; S Gimenez^1^; P Portalès^2^; M Morchikh^1^; D Maiorano^1^; J Reynes^2^; P Pasero^1^; C Psomas^1^ and P Corbeau ^1,3^

^1^Institute of Human Genetics, CNRS, Montpellier University, UMR9002, Montpellier, France. ^2^University Hospital, Montpellier, France. ^3^University Hospital, Nîmes, France

Presenting author email: pierre.corbeau@igh.cnrs.fr

**Background**: Antiretroviral therapy is highly efficient at suppressing viral replication in over 90% of HIV-1-infected patients. However, 5–25% of these virologic responders do not restore correctly their CD4 count. This suboptimal immunologic response is often correlated with a persistent hyperactivity of the immune system. However, the molecular mechanisms linking residual immune activation to impaired CD4 cell recovery remain to be identified. We tested the hypothesis that peripheral blood mononuclear cells (PBMCs) from aviremic HIV adults might induce DNA damage in CD4+ T cells resulting in their apoptosis.

**Methods**: We probed by immunofluorescence the presence of g-H2AX, 53BP1 and 8-hydroxy-2-deoxyguanosine, markers of genostress, DNA double-strand break and oxidation, respectively, in primary fibroblasts co-cultured in transwells with PBMC from virologically suppressed patients. PBMC sub-populations were sorted using magnetic beads. The amount of reactive oxygen species (ROS) expressed in the monocytes and CD4+ T cell apoptosis were measured by flow cytometry using dichloro-dihydro-fluorescein diacetate and fluorescent Annexin V, respectively. DNA-dependent protein kinase (DNA-PK) and p53 phosphorylation were analysed by western blot.

**Results**: PBMC of 56 out of 103 virologic responders (54%) induced g-H2AX nuclear foci in cocultured fibroblasts. Cell sorting and inhibition of this phenomenon by a ROS scavenger and an NADPH oxidase-inhibitor established that this genostress, characterized by DNA oxidation and double-strand break, was due to ROS released by monocytes. In cocultured CD4+ T cells, this resulted in DNA-PK as well as p53 phosphorylation, and finally in apoptosis. Patients with PBMC able to damage DNA, a phenotype that we found stable over time, presented with lower CD4 recovery than patients whose PBMC did not induce DNA damage (*p* = 0.003). Patient CD4 slopes were inversely correlated with the intensity of DNA damage induced by their PBMC (*r* = −0.419, *p* = 0.006).

**Conclusions**: ROS are persistently produced by monocytes in half of virologic responders, inducing DNA damage, cell death in CD4+ T cells and impaired CD4 restoration. This phenomenon could pave the way for oncogenesis and could be an important driver of CD4 loss in non-treated persons. ROS inhibitors deserve to be tested in non-immunologic responders.

## MOPDA0105

### HIV-1-mediated induction of hypoxia inducible factor-1 alpha activity in CD4+ T cells modifies immunometabolic phenotype and decreases cell survival

GA Duette ^1^; J Rubione^1^; P Pereyra Gerber^1^; F Erra Diaz^1^; A Varese^1^; C Palmer^2^ and M Ostrowski^1^

^1^Instituto de Investigaciones Biomédicas en Retrovirus y SIDA, UBA-CONICET, Buenos Aires, Argentina. ^2^Burnet Institute, Melbourne, Australia

Presenting author email: gabriel.duette@gmail.com

**Background**: Chronic T cell activation and dysfunction are hallmarks of HIV infection. Taking into consideration that T cell metabolism influences T cell functionality, we hypothesized that CD4+ T cell dysfunction during HIV infection could be associated with virus-induced metabolic alterations. A critical transcription factor in the coordination of T cell metabolism, differentiation and effector function is hypoxia inducible factor-1α (HIF-1α). Herein, we analysed the expression, activity and function of HIF-1α in CD4+ T cells.

**Methods**: CD4+ T cells isolated from the blood of healthy donors were activated with anti-CD3/CD28 antibodies and infected *in vitro* with HIV. HIF-1α activity was evaluated by using a reporter cell line, expressing GFP under the control of the Hypoxia-Responsive-Element. Cytokine production was evaluated by CBA kit. Cell viability was evaluated by 7-AAD staining and Annexin V binding. Silencing of HIF-1α expression was achieved by transduction with lentivirus-encoded shRNAs. To analyse *ex vivo* the relationship between HIF-1α levels and cell death in CD4+ T cells, a total of seven HIV-1-infected patients on cART and six healthy donors were recruited.

**Results**: We show that HIV-1 infection triggers HIF-1α expression and activity, promoting aerobic glycolysis and the production of proinflammatory cytokines in CD4+ T cells infected *in vitro*. We also observed that the promotion of aerobic glycolysis by HIV is associated with a higher rate of CD4+ T cell death. Remarkably, silencing HIF-1α expression in CD4+ T cells reverted the promotion of cell death and production of proinflammatory cytokines induced by HIV-1 infection. Finally, we also analysed HIF-1α expression in samples from HIV-1-infected patients on cART. Interestingly, these patients also exhibit higher levels of HIF-1α expression compared to healthy donors. Moreover, the expression levels of this transcription factor presented a negative correlation with CD4+ T cell counts.

**Conclusions**: In conclusion, we show that HIV infection induces the activity of HIF-1α in productively infected cells promoting glycolytic activity, a proinflammatory phenotype and cell death. These results pave the way to explore the possibility of targeting HIF-1α and/or T cell metabolism to restore CD4+ T cell physiology in HIV-1-infected individuals.

## MOPDA0106

### Toll-like receptor activation modulates inflammation and HIV-1 infection in the female reproductive tract (FEMINIVI study)

FD Benjelloun ^1,2^; H Quillay^1,3^; C Cannou^1,2^; R Marlin^1,2,4^; Y Madec^5^; H Fernandez^6^; F Chrétien^7^; R Le Grand^2,4^; F Barré-Sinoussi^8^; M-T Nugeyre^1,2,4^ and E Menu^1,2,4^

^1^Institut Pasteur, MISTIC Group – Virology, Paris, France. ^2^CEA, U1184 ImVA-IDMIT, Fontenay aux Roses, France. ^3^University paris diderot, Paris, France. ^4^Vaccine Research Institute (VRI), Creteil, France. ^5^Institut Pasteur, Research Unit Epidemiology of Emerging Diseases, Paris, France. ^6^Bicêtre Hospital AP-HP, Gynecology-Obstetrics, Le Kremlin Bicêtre, France. ^7^Institut Pasteur, Research Unit Human Histopathology and Animal Models, Paris, France. ^8^Institut Pasteur, Paris, France

Presenting author email: fahd.benjelloun@pasteur.fr

**Background**: The female reproductive tract (FRT) mucosae are the main sites of exposure and entry of sexually transmitted infections (STIs) including HIV-1. The toll-like receptors (TLRs), widely expressed in the FRT, recognize pathogenic motifs and modulate immune responses. TLR stimulation induces an immune activation and/or a local production of inflammatory markers. This inflammation has a controversial impact on the antiviral activity. The aim of this project is to determine *in vitro* the impact of the TLR activation in the control of HIV-1 infection and on the inflammation in the major mucosal sites of the human FRT.

**Methods**: Samples from different compartments of the FRT (vagina, cervix and uterus) were obtained from HIV-1 negative non-menopausal women who gave their written informed consent. To test the potential antiviral effect of the TLRs, mononuclear cells isolated from the samples were stimulated by TLR agonists 72 h prior to HIV-1 infection: PolyI:C (TLR3), LPS (TLR4), R848 (TLR7/8) and the CpG ODN (TLR9). Viral production was measured in cell culture supernatants by p24 Ag ELISA. Prior to HIV-1 BaL infection, the modulation of cytokine production was quantified by Luminex assay and evaluated by confocal microscopy in tissue sections of TLR pre-stimulated biopsies.

**Results**: Each compartment of the FRT presents a specific composition in immune cells. Among CD45+ cells, CD3+ T cells (30–56%) and CD14+ cells (9–16%) are the major populations in the FRT. The NK cells (CD56+) are more abundant in the uterus (4%). The stimulation of TLR3, 7/8 and 9 controls more efficiently HIV-1 infection in the uterus than in other compartments, while TLR4 stimulation does not have a major impact. TLR stimulation also modulates the production of pro- and anti-inflammatory cytokines such as interleukin (IL)-6, IL-8 and IL-10.

**Conclusions**: Our results show that the TLR stimulation modulates the cytokine expression and impacts the HIV-1 infection according to compartments in the FRT. Our aim is now to determine if a synergetic effect is obtained by stimulating more than one TLR at the time. The effect of induced inflammation on HIV-1 infection in the FRT is under investigation. Our findings will give clues for developing novel strategies against STIs.

## MOPDB0101

### More and earlier cardiovascular events (CVEs) and shorter overall survival (OS) in HIV-positive patients (HIV positive) compared to the general population differ by sex

S Esser ^1^; M Arendt^2^; C Schulze^1^; V Holzendorf^3^; NH Brockmeyer^4^; K-H Jöckel^2^; R Erbel^2^; N Reinsch^5^; HIV HEART Study Group and Heinz Nixdorf Recall Investigative Group

^1^Department of Venereology, University Hospital Essen, Clinic of Dermatology, Essen, Germany. ^2^University Hospital Essen, Institute for Medical Informatics, Biometry and Epidemiology (IMIBE), Essen, Germany. ^3^University Leipzig, Clinical Trial Centre Leipzig – Coordination Centre for Clinical Trials (ZKS Leipzig – KKS), Leipzig, Germany. ^4^Ruhruniversity Bochum, Germany Clinic of Dermatology, Venerology and Allergology, Center of Sexual Health and Medicine, Bochum, Germany. ^5^Department of Internal Medicine I and Cardiology, Division of Electrophysiology, Alfried Krupp Hospital, Essen, Germany

**Background**: The OS of HIV positive should be adapted to the general population by antiretroviral treatment. But in the ageing HIV positive, CVE and strokes became more frequent.

**Methods**: We compare CVE, stroke and OS of HIV-positive outpatients of the HIV HEART study (HIVH) and of HIV-negative controls of the population-based Heinz Nixdorf Recall study (HNR), both recruited from the German Ruhr area. HIVH cases with HNR controls are matched in a 1:2 ratio by sex and age. CVEs are defined by myocardial infarction and sudden cardiac death. Cox proportional hazard models are used to investigate the impact of study affiliation on OS, CVE and stroke with time from study start to event or last contact. We adjust for age, active smoking and for men additionally for diabetes.

**Results**: Descriptions are shown in Table 1. We observe adjusted Hazard ratios (HRs) of 3.5 (95% CI: 2.2; 5.5) for CVE for male HIVH vs. HNR and for stroke of 1.8 (0.8; 3.9) for male and 2.2 (0.1; 42.9) for female HIVH vs. HNR. The OS in male and female HIVH vs. HNR has an HR of 3.9 (2.5; 6.1) and 1.7 (0.2; 12.3), respectively. The smoking status is different in male subjects (*p* < 0.0001) and the Framingham Risk Score (FRS) is different in female subjects (*p* < 0.0001). Men differ highly in variables related to blood fats and BMI, and women differ in terms of blood pressure and heartrate which is also displayed in the highly different FRS.

**Abstract MOPDB0101 – Table 1.** Description of the matched study population of 537.

	HNR male	HIVH male	*p*-Value	HNR female	HIVH female	*p*-Value
*N* (%)	950 (66.7)	475 (33.3)		124 (66.7)	62 (33.3)	
Age mean ± SD	54.5 ± 6.7	54.5 ± 6.7		51.3 ± 6.1	51.3 ± 6.1	
Cardiovascular events *N* (%)	40 (4.2)	47 (9.9)	<0.0001	0	2 (3.2)	0.0443
Stroke *N* (%)	16 (1.7)	12 (2.5)	0.2803	1 (0.8)	1 (1.61)	0.6152
Deceased *N* (%)	49 (5.2)	52 (11.0)	<0.0001	2 (1.6)	2 (3.2)	0.4747
Never smoker *N* (%)	278 (29.3)	111 (23.4)		51 (41.5)	22 (36.1)	
Former smoker *N* (%)	371 (39.1)	126 (26.5)		38 (30.9)	10 (16.4)	
Active smoker *N* (%)	299 (31.5)	238 (50.1)	<0.0001	34 (27.6)	29 (47.5)	0.0157
FRS mean ± SD	12.6 ± 7.3	11.8 ± 3.1	0.0295	5.5 ± 5.6	14.6 ± 4.7	<0.0001

**Conclusions**: HIV-positive males had an increased incidence of CVE compared with HIV-negative controls in spite of similar FRS at baseline in contrast to HIV-positive females with higher FRS than controls but comparable rates of CVE. HIV-positive males had the highest mortality rate and a higher risk to die or to get CVE at younger age than the general population.

## MOPDB0102

### Incidence of renal Fanconi syndrome in patients taking antiretroviral therapy including tenofovir disoproxil fumarate

NA Medland ^1^; EPF Chow^2^; RG Walker^3^; M Chen^2^; THR Read^2^ and CK Fairley^2^

^1^Monash University, Central Clinical School, Melbourne, Australia. ^2^Monash University, Melbourne Sexual Health Centre, Alfred Health, Central Clinical School, Melbourne, Australia. ^3^Monash University, Renal Medicine Department, Alfred Hospital, Central Clinical School, Melbourne, Australia

Presenting author email: nmedland@mshc.org.au

**Background**: Fanconi syndrome (FS) is a well-recognized complication of use of the antiretroviral agent of tenofovir disoproxil fumarate (TDF). Despite millions of patient years of TDF use, the incidence and predictors of FS are not known. The aim of this study was to determine the incidence and predictors of FS in a clinic cohort of patients taking TDF.

**Methods**: Clinical records and laboratory investigations from patients receiving ART between January 2002 and March 2016 at the Melbourne Sexual Health Centre, Australia, were extracted. FS was defined as new onset normoglycaemic glycosuria and proteinuria and at least one other marker of renal dysfunction. Kaplan–Meier survival curves were performed using duration of exposure to ART: not containing TDF, or containing TDF, with or without ritonavir co-administration. Cox regression analysis was performed on TDF exposures with using the covariates ritonavir co-administration, age, sex, co-morbidities (hypertension, hyperlipidemia, diabetes and viral hepatitis), CD4 cell count nadir and baseline eGFR.

**Results**: 1537 patients received ART, including 1260 who received TDF, of whom 398 patients received TDF co-administered ritonavir, representing 10,401 patient years (PYs) of ART, 5327 PYs of TDF and 1641 PYs of TDF ritonavir co-administration. Thirteen cases of FS were identified. All cases were taking TDF and the mean duration of exposure was 55 months (12–98) before developing FS. The incidence of FS was 1.09/1000 PYs (95% CI 0.54–1.63) of TDF exposure (without ritonavir) and 5.48/1000 PYs (3.66–7.33) of TDF–ritonavir co-administration (*p* = 0.0057). The adjusted hazards ratio for ritonavir co-administration was 4.71 (1.37–16.14, *p* = 0.014). Known risk factors for chronic kidney disease were not associated with an increased risk of FS.

**Conclusions**: In this first published study of the incidence of FS, we find that ritonavir co-administration but not other factors is associated with a greater risk of FS. FS developed late. Our study supports ongoing renal monitoring in long-term supressed patients with twice yearly urinalysis, particularly if serum laboratory monitoring is not available.

## MOPDB0103

### Higher HDL, better brain? Higher HDL cholesterol is associated with better cognition in a cohort of older persons living with HIV infection

A Makanjuola ^1,2,3^; K Wu^4^; K Tassiopoulos^4^; B Berzins^3^; A Ogunniyi^1,2^; K Robertson^5^; B Taiwo^3^ and F Chow^6^

^1^University of Ibadan, Ibadan, Nigeria. ^2^University College Hospital, Ibadan, Nigeria. ^3^Northwestern University, Chicago, USA. ^4^Harvard School of Public Health, Boston, USA. ^5^University of North Carolina at Chapel Hill, Chapel Hill, USA. ^6^University of California San Francisco, San Francisco, USA

Presenting author email: tomimakay@gmail.com

**Background**: Despite effective combination antiretroviral therapy (ART), neurocognitive impairment (NCI) remains prevalent in persons living with HIV (PLWH). The contribution of cardiovascular comorbidities to NCI may increase as PLWH age. We investigated the association of cardiovascular risk factors with prevalent NCI in a prospective cohort of older PLWH at entry into the AIDS Clinical Trials Group A5322 study.

**Methods**: Participants who underwent a brief neurocognitive screen (Trailmaking Tests A and B, HVLT-R, Digit Symbol) at entry into A5322 were eligible. Primary outcomes were overall cognitive performance summarized by mean *z*-scores of the 4 tests (NPZ-4) and presence of NCI, defined as >1 SD below the mean on two or more tests or >2 SD below the mean on one test. We used linear and logistic regression models to determine the association between cardiovascular risk and the primary outcomes.

**Results**: Of 988 participants (30% black, 21% Hispanic/Latino, 20% women), mean age was 52 years and education 14 years. Median ART duration was eight years, mean CD4 count 661 cells/mm^3^ and 90% of participants had viral load <40 copies/mL. Current smoking (26%), statin (27%) and anti-hypertensive (36%) use were common, while stroke (2%), myocardial infarction (3%) and injection drug use (<1%) were uncommon. Mean LDL and HDL cholesterol were 109 and 49 mg/dL, respectively, and systolic blood pressure was 126 mmHg. One hundred and eighty participants (18%) had NCI. In demographics and education-adjusted models, higher HDL was associated with better NPZ-4 (+0.04, *p* = 0.040) and lower odds (OR 0.88, *p* = 0.043) of NCI per 10 mg/dL higher HDL, as was statin use (+0.15 NPZ-4, *p* = 0.037). An association between smoking and worse NPZ-4 (−0.15, *p* = 0.053) became non-significant after controlling for anti-depressant use and hepatitis C. In a multivariable model including factors significant at *p* < 0.10 in demographics and education-adjusted analyses, older age, female sex, Hispanic/Latino ethnicity, high school education or less, and anti-depressant use were associated with worse NPZ-4. Longer ART duration and higher HDL were associated with better NPZ-4.

**Conclusions**: Among older PLWH with well-controlled cardiovascular risk factors, higher HDL was associated with better cognition. Investigation into the impact of modifying HDL cholesterol on cognition in PLWH is merited.

## MOPDB0104

### The combination of tai chi, cognitive behavioural therapy and motivational text messaging improves physical function, reduces substance use and improves pain in older HIV-infected adults

JE Lake ^1,2^; MC Reid^3^; SG Edwards^4^; A Kuerbis^5^; J Candelario^6^; D Liao^7^; L Tang^7^; AA Moore^8^; for the STOP PAIN Study

^1^University of Texas Health Science Center Houston, Medicine/Infectious Diseases, Houston, USA. ^2^University of California, Los Angeles, Medicine/Infectious Diseases, Los Angeles, USA. ^3^Cornell University, New York, USA. ^4^University of California, Los Angeles, Psychiatry and Biobehavioral Sciences, Los Angeles, USA. ^5^Hunter College, New York, USA. ^6^APAIT, Special Service for Groups, Los Angeles, USA. ^7^University of California, Los Angeles, Los Angeles, USA. ^8^University of California, San Diego, San Diego, USA

Presenting author email: jordan.e.lake@uth.tmc.edu

**Background**: Chronic pain is common among older HIV-infected (HIV positive) adults and contributes to substance use, reduced physical function and disability. We designed a pilot randomized controlled trial of cognitive behavioural therapy (CBT) + tai chi + motivational texting vs. standard of care to reduce pain, disability and substance use in older HIV-positive adults.

**Methods**: Evidence-informed chronic pain and substance abuse CBT protocols were adapted and combined with group tai chi and motivational text messaging (EXP). HIV-positive adults ≥50 years of age with chronic pain and substance use (*n* = 55) were randomized to EXP (*n* = 18), support group control (cINT, *n* = 19) or no intervention (noINT, *n* = 18) for 8 weeks plus a 4-week post-study follow-up. All participants also completed daily diary assessments. Effects were compared within and between groups. Linear regression models assessed factors, including treatment assignment, associated with physical function, pain, substance use and quality of life.

**Results**: Participants had mean age 55 years, 17 years HIV positive, 11 years chronic pain; 84% were non-white; and 76% male and 9% transgender women. Approximately 1/3 each reported alcohol, marijuana and stimulants as their preferred substance. At baseline, all participants had physical and mental health (SF-12) scores below population means, and 87% had reduced physical function (short physical performance battery [SPPB] ≤10). After 8 weeks, only EXP participants had significantly improved SPPB scores (+31%, within-group *p* < 0.001, between-group *p* = 0.04) and physical function (60% less reduced physical function) that persisted at the post-study follow-up visit and after controlling for age, education, HIV and baseline pain severity. After 12 weeks, both EXP (−55%, *p* = 0.04) and cINT (−120%, *p* = 0.03) participants demonstrated reduced 30-day preferred substance use. However, only EXP participants experienced reduced overall substance use (−51%, *p* = 0.02) and improved percent pain relief (−76%, *p* = 0.03).

**Conclusions**: An eight-week combined CBT, tai chi and motivational text messaging intervention significantly improved physical function in older HIV-positive adults with chronic pain and substance use. After 12 weeks, substance use decreased and achievable pain relief also improved, possibly reflecting delayed onset of improvement in these outcomes. Further study will determine whether CBT + tai chi + motivational texting can provide sustained improvements in this vulnerable population.

## MOPDB0105

### Capacity to screen and manage mental health disorders at HIV treatment sites in low- and middle-income countries

A Parcesepe ^1^; C Mugglin^2^; M Egger^2^; F Nalugoda^3^; C Bernard^4^; E Yunihastuti^5^; S Duda^6^; C William Wester^7^; D Nash^8^; IeDEA Network Collaboration

^1^Columbia University, HIV Center for Clinical and Behavioral Studies, New York, USA. ^2^University of Bern, Institute of Social and Preventive Medicine, Bern, Switzerland. ^3^Rakai Health Sciences Program, Kalisizo, Uganda. ^4^Bordeaux School of Public Health, Bordeaux, France. ^5^Universitas Indonesia/Cipto Mangunkusumo Hospital, Faculty of Medicine, Jakarta, Indonesia. ^6^Vanderbilt University, Nashville, USA. ^7^Vanderbilt University School of Medicine, Nashville, USA. ^8^City University of New York, Institute for Implementation Science in Population Health, New York, USA

Presenting author email: ap3471@cumc.columbia.edu

**Background**: Mental disorders are prevalent among people living with HIV (PLWH) and are associated with inferior HIV treatment outcomes, including poor adherence to antiretroviral therapy and lack of viral suppression. Integrating interventions to treat mental disorders into HIV care is a key strategy to improve HIV treatment outcomes in low- and middle-income countries (LMIC), but data on HIV treatment sites’ capacities to screen and manage mental disorders are limited.

**Methods**: We report preliminary findings from an ongoing survey among a stratified random sample of 142 HIV treatment sites in 36 LMIC that participate in the International epidemiologic Databases to Evaluate AIDS (IeDEA). This analysis focuses on depression, posttraumatic stress disorder (PTSD) and substance use disorders (SUD) screening and management in the 53 adult HIV treatment sites that have completed the survey so far.

**Results**: Most sites (*n* = 40, 75%) were in urban areas. Although 34 sites (64%) reported screening for depression, only 14 (26%) sites had guidelines for screening. Screening for depression and PTSD was more common in low-income countries than middle-income countries. This trend was reversed for SUD screening (Table 1). Depression, PTSD and SUD were managed on site (defined as having services provided at the HIV clinic or the same health facility) in 62%, 43% and 36% of sites, respectively. Selective serotonin reuptake inhibitors (SSRIs) were available in all sites from upper-middle income countries, but only in 22% and 35% of low-income and lower-middle income countries, respectively.

**Conclusions**: Interim findings suggest that most of the HIV treatment facilities surveyed have integrated some mental health services and that screening for depression is commonly reported in low-income countries. However, on-site management of depression is less common in these settings. Additional research is needed to understand individual, organizational and contextual factors that may influence availability of mental health interventions in LMIC.

**Abstract MOPDB0105–Table 1.** Screening and management of mental disorders.

	Low-income countries *N* = 18, *n* (%)	Lower-middle-income countries *N* = 23, *n* (%)	Upper-middle-income countries *N* = 12, *n* (%)	Total *N* = 53, *n* (%)
Depression screening	13 (72)	14 (61)	7 (58)	34 (64)
Guideline available for depression screening	8 (44)	3 (13)	3 (25)	14 (26)
Depression management on site	10 (56)	12 (52)	11 (92)	33 (62)
PTSD screening	8 (44)	2 (9)	2 (17)	12 (23)
Guideline available for PTSD screening	7 (39)	2 (9)	1 (8)	10 (19)
PTSD management on site	8 (44)	5 (22)	10 (83)	23 (43)
Substance use disorders screening	8 (44)	18 (78)	8 (67)	34 (64)
Guideline available for substance use disorders screening	6 (33)	4 (17)	4 (33)	14 (26)
Substance use disorders management on site	6 (33)	5 (22)	8 (67)	19 (36)

## MOPDC0101

### Methods of gestational age (GA) assessment influence the observed association between ART exposure and preterm delivery (PTD): a prospective study in Cape Town, South Africa

T Malaba ^1,2^; M-L Newell^3^; H Madlala^1,2^; A Perez^1,2^; C Gray^4^; L Myer^1,2^; for the PIMS Study

^1^University of Cape Town, Division of Epidemiology and Biostatistics, Cape Town, South Africa. ^2^University of Cape Town, Centre for Infectious Diseases Epidemiology & Research, Cape Town, South Africa. ^3^University of Southampton, Faculty of Medicine, Southampton, UK. ^4^University of Cape Town, Division of Immunology, Cape Town, South Africa

Presenting author email: thokomalaba@gmail.com

**Background**: The association between antenatal ART and PTD in HIV-positive women is controversial and has not been reliably quantified. Measuring GA is challenging in resource-limited settings; different methods could explain heterogeneous findings. We examined the impact of GA estimation methods on observed PTD deliveries rates, by maternal ART.

**Methods**: Between April 2015 and October 2016, we enrolled women regardless of HIV status attending a large primary care antenatal clinic. Midwives estimated GA by last menstrual period (LMP) and symphysis-fundal height (SFH); separately, obstetric ultrasound was performed by a research sonographer blinded to midwife GA assessment if clinical GA was <24 weeks. Analyses compared GA estimated by ultrasound, SFH and LMP; the association between HIV/ART status and PTD was examined by GA assessment method using multivariable logistic regression.

**Results**: Of 1060 women (median age 28 years; 46% HIV positive, of whom 48% initiated ART pre-conception vs. 52% during pregnancy), 82% had LMP-based GA, 71% had SFH-based GA, 58% had ultrasound-based GA and 54% (*n* = 576) had GA based on ultrasound and at least one other method. At first ANC visit, estimated median (interquartile range) GA was 18 weeks (12–23 weeks) by LMP, 23 weeks (18–28 weeks) by SFH and 17 weeks (13–21 weeks) by ultrasound. Overall PTD <37 weeks was observed in 41% by LMP, 27% by SFH and 12% by ultrasound. In 1037 live-singleton births (mean birthweight 3124 g; 10% SGA <10th centile), PTD risk was doubled for HIV-infected compared to uninfected women for ultrasound-based GA (odds ratio = 2.01, 95% confidence interval = 1.15–3.51); but for LMP/SFH-based GA, the association was not significant (Figure 1). These differences between GA assessment methods persisted after adjustment for age, parity, height and previous PTD; PTD risk did not vary by ART initiation timing for any GA method.

**Conclusions**: Findings for an association between HIV/ART and PTD are substantially influenced by GA assessment method. With growing scientific interest in this association, future research efforts should seek to standardize optimal measures of gestation.

**Abstract MOPDC0101–Figure 1.**
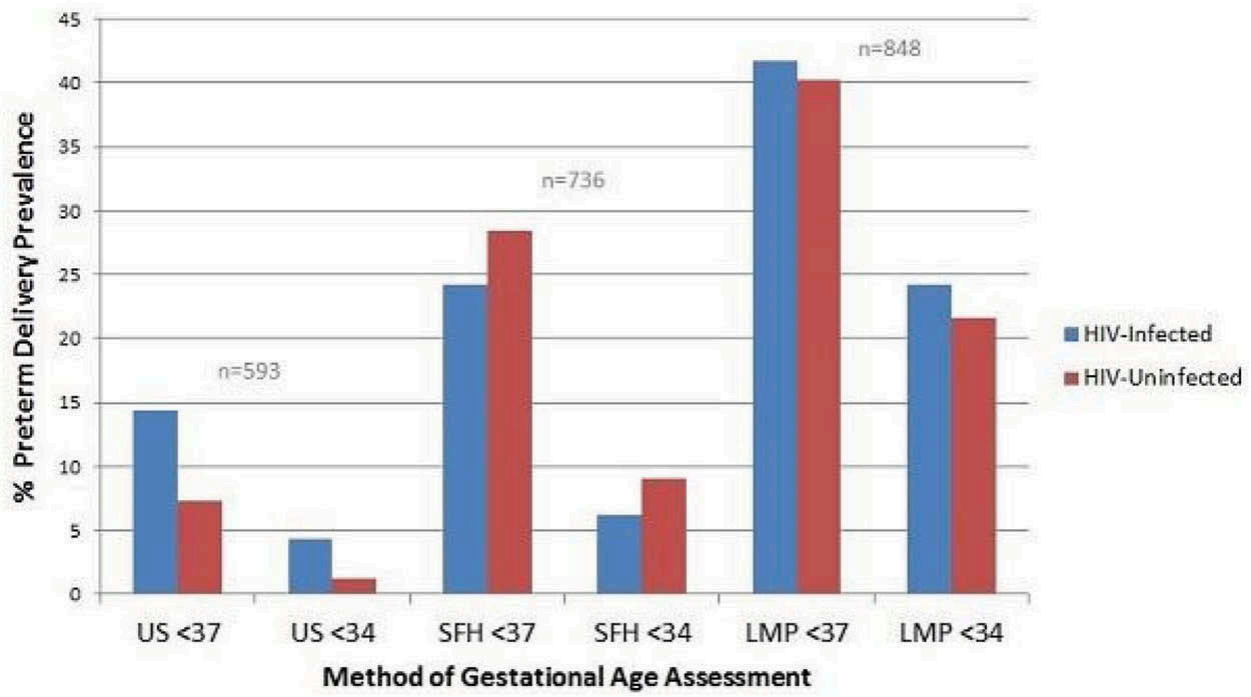
Prevalence of preterm (<37) and very preterm (<34) deliveries by HIV status.

## MOPDC0102

### Pregnancy outcomes and infant survival in the era of universal HAART in Africa: the POISE study

S Dadabhai ^1^; L Gadama^2^; R Chamanga^2^; R Kawalazira^2^; C Katumbi^2^; D Dula^2^; I Singini^2,3^; L Degnan^1^; M Kamanga^2^; B Lau^4^ and TE Taha^4^

^1^Johns Hopkins Bloomberg School of Public Health, Epidemiology, Blantyre, Malawi. ^2^College of Medicine-Johns Hopkins Research Project, Blantyre, Malawi. ^3^University of Cape Town, Biostatistics, Cape Town, South Africa. ^4^Johns Hopkins Bloomberg School of Public Health, Epidemiology, Baltimore, USA

Presenting author email: sufia@jhu.edu

**Background**: In the era of universal HAART, concerns remain that triple therapy may increase adverse pregnancy outcomes. We compared low birth weight (LBW), preterm birth (PTB) and survival in infants born to ART-experienced women and to uninfected women in Blantyre, Malawi, where first-line ART includes tenofovir, lamivudine and efavirenz.

**Methods**: We enrolled HIV-infected and HIV-uninfected women and their babies at delivery into a one-year prospective study at four health facilities. Eligibility included confirmed HIV status, consent, singleton births, CD4 >350 cells/ml^3^ and no stage 3/4 HIV. We documented sociodemographic data, clinical and reproductive history, maternal and infant survival, birth weight and gestational age using Ballard score. We applied logistic regression to measure the association between HIV and LBW and PTB. Odds ratios and 95% confidence intervals (CIs) are presented.

**Results**: We enrolled 459 HIV-uninfected and 335 HIV-infected women on ART from January to December 2016; 1.4% were virally suppressed at baseline (<40 copies per/ml). Rates of LBW among HIV-infected and HIV-uninfected women were 4.2% and 3.4%, respectively; *p* = 0.69; 11.9% of HIV-infected women and 12.0% of HIV-uninfected women delivered PTB; *p* = 0.99. In multivariate analyses for LBW and PTB (Table 1), there was no association between HIV status and adverse outcomes. Among all women, having more than one pregnancy (odds ratio (OR) = 0.36, 95% CI (0.18, 0.73)) or more than one birth (OR = 0.39, 95% CI (0.19, 0.82)) was protective against LBW. PTB was 8.9% among women starting ART before or during first trimester; 15.5% among those starting in second trimester; and 17.9% among women starting in third trimester (*p* = 0.06 for trend). Two infants died: one HIV exposed and one HIV unexposed. No maternal deaths were observed.

**Abstract MOPDC0102–Table 1.** Adverse pregnancy outcomes by HIV status.

		Low birth weight		Gestational age	
Baseline characteristics		OR (95% CI)	Adjusted OR (95% CI)	OR (95% CI)	Adjusted OR (95% CI)
HIV status (reference = HIV negative, *n* = 408)	HIV positive (*n* = 310)	1.23 (0.57, 2.66)	2.08 (0.88, 4.90)	0.99 (0.63, 1.57)	1.18 (0.69, 1.98)
Maternal age	Per year of age	0.96 (0.89, 1.03)	0.95 (0.88, 1.03)	0.98 (0.95, 1.02)	0.99 (0.94, 1.03)
Body mass index at delivery (kg/m^2^) (reference = 18.5–24.9, *n* = 426)	<18.5 (*n* = 15)	1.83 (0.23, 14.78)	2.12 (0.25, 17.83)	1.92 (0.52, 7.05)	2.26 (0.59, 8.63)
	≥25 (*n* = 267)	1.00 (0.45, 2.25)	0.99 (0.43, 2.32)	1.05 (0.65, 1.68)	1.11 (0.67, 1.82)
Estimated work load during pregnant (reference = in house only, *n* = 569)	In house + outdoor (*n* = 43)	0.63 (0.83, 4.79)	0.71 (0.09, 5.58)	1.10 (0.42, 2.91)	1.33 (0.49, 3.63)
	Moderate to heavy (*n* = 92)	0.27 (0.04, 2.00)	0.24 (0.03, 1.84)	0.62 (0.28, 1.40)	0.54 (0.22, 1.29)
Electricity at home (reference = yes, *n* = 337)	No (*n* = 382)	1.29 (0.59, 2.82)	1.33 (0.59, 2.99)	1.13 (0.72, 1.78)	1.30 (0.80, 2.12)
Haemoglobin (reference = ≥10 mmHg, *n* = 522)	<10 mmHg (*n* = 151)	2.28 (0.68, 7.71)	2.81 (0.81, 9.75)	1.20 (0.67, 2.14)	1.33 (0.72, 2.46)

**Conclusions**: It is reassuring to observe that adverse outcomes were not different between healthy HIV-infected women and HIV-uninfected women. It appears that near-universal ART can eliminate mother-to-child transmission of HIV without significant negative impact on other pregnancy outcomes.

## MOPDC0103

### ARV drug concentrations in breastmilk, viral load and HIV transmission to the infant

N Davis ^1^; A Corbett^2^; J Kaullen^2^; J Nelson^2^; C Chasela^3^; D Sichali^4^; M Hudgens^2^; W Miller^5^; D Jamieson^1^; A Kourtis^1^; BAN study team

^1^Centers for Disease Control and Prevention, Division of Reproductive Health, Atlanta, USA. ^2^University of North Carolina at Chapel Hill, Chapel Hill, USA. ^3^University of Witwatersrand, Johannesburg, South Africa. ^4^UNC Project Malawi, Lilongwe, Malawi. ^5^Ohio State University, Columbus, USA

Presenting author email: dwg4@cdc.gov

**Background**: Concentration of antiretroviral (ARV) drug found in plasma, as well as the amount of drug excreted into breastmilk, may affect the rate at which ARVs suppress viral replication, and/or the duration of viral suppression, affecting HIV viral load and potentially HIV transmission from mother to infant.

**Methods**: A case cohort study was conducted using data from the Breastfeeding, Antiretrovirals and Nutrition study to (1) examine correlation between plasma and breastmilk ARV drug concentration, (2) estimate associations between ARV drug concentration and HIV viral load and (3) compare time to breastmilk HIV transmission by plasma drug concentration status. We included mothers randomized to 28 weeks of postpartum maternal ARVs or infant nevirapine who had ≥1 plasma or breastmilk (maternal ARV arm only) specimen available 2–24 weeks postpartum. Among these, we included all mothers who transmitted HIV to their infants between 2 and 28 weeks and 15% of mothers who did not (*n* = 27 and 227, respectively). Plasma and breastmilk drug concentrations for nevirapine, nelfinavir and lopinavir were dichotomized using the median effective concentration (EC_50_) as a cutoff (>EC50 vs. ≤EC50). Plasma and breastmilk viral load were dichotomized as detectable (plasma: >40 copies/ml, breastmilk: >56 copies/ml) or undetectable. Spearman correlation coefficients were used to assess correlation between plasma and breastmilk ARV concentration. Associations between drug concentration and viral load cutoffs were assessed using mixed-effects models. Cox models were used to estimate the association between plasma drug concentration and breastmilk HIV transmission between 2 and 28 weeks.

**Results**: All ARV compounds exhibited substantial correlations between maternal plasma and breastmilk concentrations (rho: 0.85–0.98, *p*-value <0.0001). Having plasma drug concentration above the EC_50_ was associated with lower odds of having detectable HIV RNA (plasma odds ratio (OR) 0.69, 95% confidence interval (CI) 0.49–0.98; breastmilk OR 0.23, 95% CI 0.15–0.37) and a reduced rate of breastmilk HIV transmission (hazard ratio 0.42, 95% CI 0.18–0.98). Having breastmilk drug concentration above the EC_50_ was also associated with lower odds of having detectable HIV RNA (plasma OR 0.65, 95% CI 0.47–0.89; breastmilk OR 0.44, 95% CI 0.31–0.62).

**Conclusions**: Ensuring adequate drug concentration in both plasma and breastmilk is important for reaching and maintaining viral suppression and preventing breastmilk HIV transmission.

## MOPDC0104

### Stillbirth in HIV-infected women delivering in UK/Ireland between 2007 and 2015

G Favarato; H Bailey; H Peters; K Francis; A Horn; R Sconza; P Tookey; C Thorne; National Study of HIV in Pregnancy and Childhood (NSHPC)

UCL Great Ormond Street Institute of Child Health, Faculty of Population Health Sciences, London, UK

Presenting author email: graziella.favarato@ucl.ac.uk

**Background**: Stillbirth (SB) has multifactorial and incompletely understood causes. We aimed to assess the SB rate and associated risk factors in HIV-infected women delivering in UK/Ireland between 2007 and 2015.

**Methods**: We analysed data from singleton deliveries and defined an SB as a baby delivered at ≥24 gestational weeks (GW) showing no signs of life. We performed multivariable logistic regression of SB risk factors, adjusted for maternal age and country of origin (grouped, see Table 1), year, injecting drug user history, parity, first antenatal CD4 count ≤350 cells/µl, antenatal ART regimen, late antenatal care start (≥13 GW) and newborn gender with random effect for repeated pregnancies in the same mother.

**Results**: There were 10,157 pregnancies (in 7951 mothers) and 87 (0.9%) SB; MTCT was reported in 41 (0.4%) cases. Compared to live births (LB), SB were more likely to be male (58.7% vs. 50.6%), delivered pre-term (median 33.5 GW vs. 39 GW) and be SGA (55.2% vs. 20.4%); 7/87 (8.1%) SB had congenital abnormalities versus 295/10,070 (2.9%) LB. Compared to mothers delivering an LB, those delivering an SB were more likely to be primiparous (46.5% vs. 32.7%), older (56.3% vs. 47.2% of age ≥33 years), from eastern Africa (47.1% vs. 41.4%), more likely to book antenatal care at ≥13 GW (93.1% vs. 86.8%), have first antenatal CD4 count ≤350 cells/µl (50.8% vs. 34.5%) and more likely to receive no antenatal ART (5.8% vs. 1.6%). Multivariate analysis suggested that significant risk factors associated with SB were antenatal CD4 count ≤350 cells/µl and delivering a male newborn; women whose country of origin was not Europe or Africa were also at higher risk (Table 1).

**Abstract MOPDC0104–Table 1.** Adjusted OR (95% CI) for stillbirth.

Maternal age (per one-year increase)	1.04 (0.98, 1.10)
Area of origin: Europe	1.00
Eastern Africa	1.63 (0.55, 4.80)
Western Africa	1.35 (0.40, 4.57)
Middle/South Africa	2.24 (0.68, 7.39)
Other	4.33 (1.31, 14.26)
CD4 cells/µl: ≤350	1.00
>350	1.96 (1.09, 3.53)
Newborn: Female	1.00
Male	1.95 (1.06, 3.58)

**Conclusions**: SB rate in HIV-infected women in UK/Ireland was 0.9% in 2007–2015, around twice that in the general population (<0.5%). Further research is needed to understand circumstances around SB in this population in order to identify possible interventions.

## MOPDC0105

### Usefulness of HBV rapid tests to identify pregnant women at high risk of HBV mother-to-child transmission: the pilot ANRS 12328 study in Cambodia

O Segeral ^1^; D N’Diaye^2^; S Prak^3^; W Khamduang^4^; J Nouhin^3^; M Ek^5^; K Chim^5^; S Hout^6^; A-M Roque-Afonso^7^; P Piola^2^; N Ngo-Giang-Huong^4^ and F Rouet^3^

^1^ANRS /University of Health Sciences, Phnom Penh, Cambodia. ^2^Public Health and Epidemiology Unit, Pasteur Institute in Cambodia, Phnom Penh, Cambodia. ^3^HIV/Hepatitis Unit, Pasteur Institute in Cambodia, Phnom Penh, Cambodia. ^4^Faculty of Associated Medical Sciences; Institut de Recherche pour le Développement (IRD) UMI 174/ PHPT, Chaing Mai, Thailand. ^5^Maternity, Calmette Hospital, Phnom Penh, Cambodia. ^6^Clinical Biology Laboratory, Calmette Hospital, Phnom Penh, Cambodia. ^7^Virologie Hôpitaux Universitaires Paris Sud, Villejuif – INSERM U785 – Centre National de Référence des hépatites A & E, Villejuif, France

Presenting author email: oliseg@hotmail.com

**Background**: Mother-to-child transmission (MTCT) of HBV is mainly associated with high maternal HBV DNA viral load (VL) levels. International guidelines recommend the use of antiviral drugs (such as tenofovir (TDF)) during pregnancy if maternal VL is >200,000 IU/mL. In Southeast Asia (SEA), many pregnant women are not screened for hepatitis B surface antigen (HBsAg) and VL testing is not accessible for those who are HBsAg-positive. Here, we report among pregnant women from Cambodia, the performance of a serial algorithm using two HBV rapid diagnostic tests (RDTs), in which samples reactive for HBsAg were further tested for hepatitis B e antigen (HBeAg), as a surrogate marker for HBV replication.

**Methods**: In 2015, we prospectively collected plasma samples of 250 pregnant women from the Calmette Hospital (Phnom Penh), including women with a known positive HBsAg status. All specimens were initially tested with the SD BIOLINE HBsAg RDT (Standard Diagnostics), and results were compared to the Murex HBsAg ELISA v3.0 (Diasorin) (gold standard). HBeAg status was then blindly assessed among all ELISA HBsAg-positive samples using the SD BIOLINE HBeAg RDT, and results were compared to HBV DNA levels (PUMA HBV kit, Omunis) (gold standard). Analysis was done according to thresholds of 200,000 and 2,000,000 IU/mL.

**Results**: Overall, 128 pregnant women tested positive for HBsAg with ELISA (51.2%). The sensitivity and specificity of the HBsAg RDT, compared to ELISA, were 99.2% (95% confidence intervals, 95.7–99.9) and 100% (97.0–100), respectively. Among the 128 ELISA HBsAg-positive samples, 29 (23%) tested positive with the HBeAg RDT, 34 (26%) showed HBV viremia >200,000 IU/mL and 29 (23%) >2,000,000 IU/mL. The sensitivity and specificity of the HBeAg RDT in detecting HBV replication were 76.5% (60.0–87.6) and 96.8% (91.0–98.9) for VL >200,000 IU/mL and 89.7% (73.6–96.4) and 97.0% (93.6–100.0) for VL >2,000,000 IU/mL.

**Conclusions**: Our results strongly suggest that a combination of HBsAg and HBeAg RDTs in a serial algorithm is an efficient strategy to identify highly viremic HBV-infected pregnant women in need of TDF to prevent HBV MTCT. RDTs-based strategies can significantly improve HBV screening coverage among pregnant women, notably in SEA where HBV is highly prevalent.

## MOPDD0101

### HIV treatment and care services for adolescents: a situational analysis of 218 facilities in 23 sub-Saharan African countries

D Mark^1,2^; A Armstrong^3^; C Andrade^1^; M Penazzato^4^; L Hatane ^1^; L Taing^1^; T Runciman^1^; N Sugandhi^5^ and J Ferguson^6^

^1^PATA, Cape Town, South Africa. ^2^University of Cape Town, Psychology, Cape Town, South Africa. ^3^Consultant, London, UK. ^4^HIV Department, World Health Organisation, Geneva, Switzerland. ^5^ICAP at Colombia University, New York, USA. ^6^Africa Centre for Population Health, Health Adolescents & Young Adults Research Unit, Mtubatuba, South Africa

Presenting author email: luann@teampata.org

**Background**: In 2013, 2.1 million adolescents (age 10–19 years) were living with HIV globally. The extent to which health facilities provide appropriate treatment and care was unknown. To support understanding of service availability in 2014, Paediatric-Adolescent Treatment Africa (PATA), an NGO supporting a network of health facilities across sub-Saharan Africa, undertook a facility-level situational analysis of adolescent HIV treatment and care services in 23 countries.

**Methods**: Two hundred and eighteen facilities, responsible for an estimated 80,072 HIV-infected adolescents in care, were surveyed. Sixty per cent of the sample were from PATA’s network, with the remaining gathered via local NGO partners and snowball sampling. Data were analysed using descriptive statistics and coding to describe central tendencies and identify themes.

**Results**: Respondents represented three sub-regions: West and Central Africa (*n* = 59; 27%), East Africa (*n* = 77, 35%) and Southern Africa (*n* = 82, 38%). Half (50%) of the facilities were in urban areas, 17% peri-urban and 33% rural settings. Insufficient data disaggregation and outcomes monitoring were critical issues. A quarter of facilities did not have a working definition of adolescence. Facilities reported non-adherence as their key challenge in adolescent service provision, but had insufficient protocols for determining and managing poor adherence and loss to follow-up. Adherence counselling focused on implications of non-adherence rather than its drivers. Facilities recommended peer support as an effective adherence and retention intervention, yet not all offered these services. Almost two-thirds reported attending to adolescents with adults and/or children, and half had no transitioning protocols. Of those with transitioning protocols, 21% moved pregnant adolescents into adult services earlier than their peers. There was limited sexual and reproductive health integration, with 63% of facilities offering these services within their HIV programmes and 46% catering to the special needs of HIV-infected pregnant adolescents.

**Conclusions**: Results indicate that providers are challenged by adolescent adherence and reflect an insufficiently targeted approach for adolescents. Guidance on standard definitions for adherence, retention and counselling approaches is needed. Peer support may create an enabling environment and sensitize personnel. Service delivery gaps should be addressed, with standardized transition and quality counselling. Integrated, comprehensive sexual reproductive health services are needed, with support for pregnant adolescents.

## MOPDD0102

### Factors associated with viral suppression among adolescents living with HIV in Cambodia

S Yi^1,2^; C Ngin^1^; S Tuot^1^; P Chhoun^1^; K Pal^1^; S Sau^3^; K Chhim ^1^; SC Choub^1^; G Mburu^4^ and P Ly^3^

^1^KHANA Center for Population Health Research, Phnom Penh, Cambodia. ^2^Touro University California, Center for Global Health Research, Vallejo, USA. ^3^National Center for HIV/AIDS, Dermatology and STD, Phnom Penh, Cambodia. ^4^Lancaster University, Division of Health Research, Phnom Penh, UK

Presenting author email: ckolab@khana.org.kh

**Background**: Adolescents living with HIV on antiretroviral therapy (ART) have poorer rates of treatment adherence and viral suppression as well as higher mortality rates compared to their adult counterparts. This study investigated factors associated with viral suppression among adolescents living with HIV in Cambodia.

**Methods**: A cross-sectional study was conducted in August 2016, among adolescents living with HIV aged 15–17 years randomly selected from 11 ART clinics in the capital city and 10 provinces, utilizing a structured questionnaire. The most recent viral load test result was retrieved from medical records obtained from the ART clinics. Adolescents were categorized as having viral suppression if the viral load count was <1000 copies/ml. Multivariate logistic regression analysis was performed.

**Results**: This study included 328 adolescents with a mean age of 15.9 years (SD = 0.8); of whom, 48.5% were female. Mean duration on ART was 97 months (SD = 40.2). Proportion of adolescents with viral load suppression was 76.8%. In bivariate analyses, viral suppression was significantly associated with older age, duration on ART, higher CD4 count, better family socio-economic status, living with parents, parental education, having parents as main caregivers, no experience of negative attitude from healthcare providers, being aware that they were receiving ART, knowing that HIV is transmitted through unprotected sex with people living with HIV, understanding that there is no cure for AIDS, receiving treatment from a paediatric clinic and type of ART (first or second line). After adjustment, viral suppression remained significantly associated with longer duration on ART, higher CD4 count, receiving treatment from a paediatric clinic, being aware that they were receiving ART and better HIV-related knowledge including knowing that HIV is transmitted through unprotected sex with people living with HIV and understanding that there is no cure for AIDS.

**Conclusions**: Viral load suppression rates among adolescents living with HIV in this study were considerably high, but fall short of the global target of 90% viral suppression among people living with HIV on ART. Our findings indicate the need to strengthen treatment literacy and understanding of HIV among adolescents as they prepare for transition from paediatric to adult HIV care.

## MOPDD0103

### Evaluating the implementation and impact of the adolescent package of care at health facilities in former Nyanza province, Kenya

M Mburu ^1^; P Ongwen^1^; M Guze^2^; N Okoko^1^; SB Shade^3^; C Blat^2^; J Kadima^1^; JP Otieno^1^; N Tuma^1^; W Muriu^4^; EA Bukusi^1^; CR Cohen^1^ and HT Wolf^5^

^1^Centre for Microbiology Research (CMR), Kenya Medical Research Institute (KEMRI), Nairobi, Kenya. ^2^University of California San Francisco, Obstetrics, Gynecology & Reproductive Sciences, San Francisco, USA. ^3^Department of Medicine, University of California, Prevention Sciences, San Francisco, USA. ^4^University of Nairobi, School of Mathematics, Nairobi, Kenya. ^5^Georgetown University, Pediatrics, Washington, USA

Presenting author email: megmburu@gmail.com

**Background**: Youth represent 40% of new HIV infections worldwide including in sub-Saharan Africa. Young people respond better to HIV and sexual health services tailored for their developmental stage. In 2015, Kenya’s National AIDS and STD Control Program (NASCOP) introduced an adolescent package of care (APOC) to provide standardized adolescent services to HIV-infected adolescents. We conducted this evaluation to assess the impact of APOC on visit adherence, family planning (FP) uptake and viral load (VL) suppression.

**Methods**: Beginning in November 2015, staff at Family AIDS Care & Education Services (FACES)-supported sites were trained in APOC, which included utilizing adolescent checklists and adolescent-tailored services, that is, support groups, home re-fills, adolescent clinic days. At 16 sites utilizing electronic medical records, data for adolescents (9–19 years) were extracted on demographic characteristics, clinic appointments, FP and VL from November 2015 to December 2016. At these same sites, chart audits were conducted on a random sample of encounters per site every quarter to assess APOC checklist utilization (0–10) as a proxy for APOC implementation. Generalized estimating equations for logistic regression, accounting for repeated measures, were used to assess effect of checklist utilization on outcomes from November 2015 to December 2016.

**Results**: We assessed visit adherence and FP uptake in 19,301 encounters for 2739 HIV-infected adolescents and 1372 VL tests for 1305 adolescents. Median age was 13 years (IQR 11, 17). Females comprised 60% (*n* = 1646) and 61% (*n* = 794) of the clinical encounters and VL tests, respectively. FP uptake (aOR = 1.50; 95% CI: 1.19–1.89) and VL suppression (aOR = 1.54; 95% CI: 1.09–2.20) increased over time, as did APOC checklist utilization (aOR = 2.18; 95% CI: 1.54–3.08). Increased APOC checklist utilization was not associated with change in visit adherence and was inversely associated with FP uptake (aOR = 0.93; 95% CI: 0.89–0.98) and VL suppression (aOR = 0.87; 95% CI: 0.89–0.95) over time.

**Conclusions**: We observed increased FP uptake and VL suppression during scale-up of the APOC. However, increased utilization of the APOC checklist was not associated with these improved outcomes. The checklist is only one element of APOC. Further investigation of additional elements is needed to assess full implementation and impact of APOC on adolescent outcomes.

## MOPDD0104

### Economic context and HIV vulnerability in adolescents and young adults living in urban slums in Kenya: a qualitative analysis based on scarcity theory

L Jennings ^1^; M Muthai^2^; S Linnemayr^3^; A Trujillo^1^; M Mak’anyengo^4^; B Montgomery^5^ and D Kerrigan^6^

^1^Department of International Health, Johns Hopkins Bloomberg School of Public Health, Baltimore, USA. ^2^Department of Psychiatry, University of Nairobi, College of Health Sciences, Nairobi, Kenya. ^3^RAND Corporation, Santa Monica, USA. ^4^National Health and Development Organization (NAHEDO), Kenyatta National Hospital, Nairobi, Kenya. ^5^Univertiy of Arkansas for Medical Sciences, Fay W. Boozman College of Public Health, Health Behavior and Health Education, Little Rock, USA. ^6^Department of Health, Johns Hopkins Bloomberg School of Public Health, Behavior, and Society, Baltimore, USA

Presenting author email: ljennin6@jhu.edu

**Background**: Urban slum adolescents and young adults have disproportionately high rates of HIV compared to rural and non-slum urban youth. Yet, few studies have examined youth’s perceptions of the economic drivers of HIV. This study applied principles from traditional and behavioural economics, in particular the theory of scarcity as defined by Mullainathan and Shafir in 2013, in understanding the duality that impoverished youth face in making sexual decisions both in response to direct money shortages and under the cognitive load of financial distress.

**Methods**: Twenty focus group discussions were conducted with 120 adolescents, aged 15–17, and young adults, aged 18–22, living in one of two urban slums in Nairobi, Kenya. Using a semi-structured discussion guide, we asked youth to describe how their economic scarcity, in the form of financial, material, and physical deprivation, contributed to sexual risk behaviours and influenced their capacity to prevent HIV acquisition. All discussions were conducted in Kiswahili and translated and transcribed into English. Data were then coded and analysed using interpretive phenomenology.

**Results**: Results indicated that slum youth made many sexual decisions considered rational from a traditional economics perspective, such as acquiring more sex when resources were available, maximizing wealth through sex, being price sensitive to costs of condoms or testing services and taking more risks when protected from adverse sexual consequences. Youth’s engagement in sexual risk behaviours was also motivated by scarcity phenomena explained by behavioural economics, such as compensating for sex lost during scarce periods (i.e., risk-seeking), valuing economic gains over HIV risks (i.e., tunnelling, bandwidth tax), and transacting sex as an investment strategy (i.e. internal referencing). When scarcity was alleviated, young women additionally described reducing the number of sex partners to account for non-economic preferences (i.e. slack). These findings further revealed two implications for prevention interventions relating to gender-specific economic interests and reduced perceived costs of HIV infection.

**Conclusions**: Scarcity theory draws attention to the role of financial insecurity in altering how individuals and couples approach sexual decision-making. Combination prevention interventions, including biomedical technologies relying on adherence, should not ignore traditional and behavioural economic drivers of sexual decisions in urban poor settings.

## MOPDD0105

### The effectiveness and cost-effectiveness of community-based support for adolescents receiving antiretroviral treatment in South Africa

G Fatti^1^; N Shaikh^1^; O Oyebanji^1^; F Chirowa^2^; D Jackson^3,4^; A Goga^5,6^; T Magubu^7^; JB Nachega^8,9,10^; B Eley^11^ and A Grimwood ^1^

^1^Kheth’Impilo, Cape Town, South Africa. ^2^Kheth’Impilo, Harare, Zimbabwe. ^3^UNICEF, New York, USA. ^4^University of the Western Cape, School of Public Health, Cape Town, South Africa. ^5^South African Medical Research Council, Health Systems Research Unit, Pretoria, South Africa. ^6^Department of Paediatrics, University of Pretoria, Pretoria, South Africa. ^7^University of Zimbabwe, Harare, Zimbabwe. ^8^Departments of Epidemiology and International Health, Johns Hopkins University Bloomberg School of Public Health, Baltimore, USA. ^9^Departments of Epidemiology, Infectious Diseases and Microbiology, University of Pittsburgh Graduate School of Public Health, Pittsburgh, USA. ^10^Department of Medicine and Centre for Infectious Diseases, Stellenbosch University, Cape Town, South Africa. ^11^Department of Paediatrics and Child Health, University of Cape Town, Cape Town, South Africa

Presenting author email: ashraf.grimwood@khethimpilo.org

**Background**: Adolescents receiving antiretroviral treatment (ART) in sub-Saharan Africa are at particular risk of suboptimal adherence, inadequate viral suppression and high loss to follow-up (LTFU). Sub-Saharan Africa also has critical shortages of professional healthcare workers. Community-based support (CBS) programs are task-shifting interventions to address these shortages. Effectiveness and cost-effectiveness data of interventions improving ART outcomes amongst adolescents are very limited. We measured the effectiveness and cost-effectiveness of a large CBS programme in South Africa, the country having the highest HIV burden globally.

**Methods**: A cohort study included ART-naïve adolescents and youth (ages 10–24 years) who initiated ART at 47 facilities. CBS workers conducted regular home visits providing ART-related and adherence education, psychosocial support, symptom screening for opportunistic infections and traced patients defaulting clinic appointments. Outcomes were compared between adolescents who received CBS plus standard clinic-based care versus adolescents who received standard care only. Cumulative incidences of LTFU, mortality, CD4 count increases, viral suppression and pharmacy refill data using the medication possession ratio (MPR) were analysed using multivariable competing-risks regression, generalized estimating equations and multilevel mixed-effects models over five years of treatment. Costs of CBS were compiled and incremental cost-effectiveness ratios (annual cost per additional patient retained in care for patients receiving CBS versus not receiving CBS) were calculated.

**Results**: Of 6706 patients included, 2100 (31.3%) received CBS, 5523 (82.4%) were female and 1810 (27.0%) were aged 10–19 years. LTFU was 36% lower amongst adolescents who received CBS; being 29.9% versus 39.0% amongst adolescents with and without CBS after five years, respectively; adjusted subhazard ratio (asHR) = 0.64 (95% CI: 0.55–0.76); *p* < 0.0001). Mortality was 32% lower amongst adolescents with CBS, asHR = 0.68 (95% CI: 0.53–0.88; *p* = 0.004). Virological suppression (adjusted risk ratio = 1.04 [95% CI: 0.94–1.16]), annual CD4 count increases (coefficient = 11.7 [95% CI: −13.0 to 36.6; *p* = 0.35]) and mean MPR (81.9% vs. 82.7%; *p* = 0.16) were not significantly different. The average cost of CBS was US$49.54/patient/year. The cost per additional patient retained due to CBS was US$828 and US$594 after one and two years, respectively.

**Conclusions**: CBS for adolescents receiving ART is a low-cost intervention associated with substantially reduced LTFU and mortality and can be scaled-up in low-income countries.

## MOPDD0106

### HIV risk behaviour, risk perception and experiences in accessing HIV and sexual reproductive health (SRH) services among adolescent key populations in Kenya: a situational analysis

A Ikahu ^1^; R Kimathi^1^; M Kiragu^1^; S Njenga^1^; M Mireku^1^; M Muthare^1^; G Ombui^1^; J Kyongo^1^; R Karuga^1^; E Gitau^2^; U Gilbert^2^; H Musyoki^3^; L Digolo^1^; W Mukoma^1^ and L Otiso^1^

^1^LVCT Health, Nairobi, Kenya. ^2^UNICEF, Nairobi, Kenya. ^3^NASCOP, Nairobi, Kenya

Presenting author email: aikahu@lvcthealth.org

**Background**: Men who have sex with men, sex workers and people who inject drugs are considered key populations (KPs) due to their high risk of HIV infection and transmission. Adolescent KPs (less than 19 years) are at higher risk of HIV than their older counterparts yet programmes have not prioritized them. Legal barriers affect their access to sexual reproductive health (SRH) and HIV services. A study was conducted to identify risk perceptions and experiences that heighten risk of HIV among adolescents aged 10–19 years who engage in either sex work, same sex relationships or intravenous drug use and their uptake of SRH services.

**Methods**: A qualitative exploratory study was conducted between October 2015 and April 2016 in Nairobi, Mombasa and Kisumu Counties. Nine (9) focus group discussions and 18 in-depth interviews were conducted with 37 adolescent girls reporting sex work, 36 adolescent boys in same sex relationships and 35 adolescents involved in intravenous drug use. The participants were purposively selected from various KP community-based organizations. Data were coded thematically and analysed using Nvivo 10. Ethical approval was obtained from AMREF ESRC.

**Results**: 108 adolescent KPs (51 female and 57 male) ranging from 10 to 19 years were interviewed. They reported similar experiences that placed them at heightened HIV risk; being forced to have “condomless” sex for more money, selling sex to sustain themselves and their dependents (22 of the 37 adolescent FSWs had children), sexual abuse and physical abuse from clients and police. Self-perceptions of HIV risk were mixed; those practicing sex work had multiple sexual partners perceived themselves to be at high risk while those having one partner perceived themselves at low risk. All groups preferred accessing SRH services in private unlike public facilities due to stigma and discrimination.

**Conclusions**: The findings demonstrate the challenges faced by the often forgotten adolescent KPs highlighting the need for adolescent KP friendly policies, services and structural interventions to address poverty, legal barriers to service access, violence, stigma and discrimination. HIV prevention interventions should address knowledge of and self-perception of HIV risk to prevent HIV acquisition and transmission.

## TUPDA0101

### Combined IL-21 and IFNα treatment limits residual inflammation and delays viral rebound in SIV-infected macaques

L Micci^1^; J Harper^1^; S Paganini^1^; C King^1^; E Ryan^1^; F Villinger^2^; J Lifson^3^ and M Paiardini ^4,5^

^1^Emory University, Yerkes National Primate Research Center, Atlanta, USA. ^2^University of Louisiana at Lafayette, New Iberia Research Center, New Iberia, USA. ^3^Leidos Biomedical Research, Frederick, USA. ^4^Department of Medicine, Emory University School of Medicine, Atlanta, USA. ^5^Department of Microbiology and Immunology, Yerkes National Primate Research Center, Emory University, Atlanta, USA

Presenting author email: lmicci@emory.edu

**Background**: Although antiretroviral therapy (ART) suppresses HIV replication, immune dysfunctions and chronic inflammation critically contribute to non-AIDS-related morbidity and mortality in infected subjects. Furthermore, inflammation facilitates HIV persistence during ART. We previously demonstrated that addition of Interleukin (IL)-21, an immunomodulatory cytokine, reduces chronic inflammation and HIV persistence in ART-treated, SIV-infected rhesus macaques (RMs). This study sought to combine the anti-inflammatory functions of IL-21 with the antiviral properties of IFNa to reinvigorate antiviral responses. We hypothesize an impact on viral rebound following ART treatment interruption (ATI).

**Methods**: 15 RMs were infected with SIV_mac239_ IV. RMs started a triple formulation of TDF, FTC and Dolutegravir (DTG) day 35 post-infection and continued for at least 12 months. Eight RMs received Macaquized (M)-IL-21-IgFc (100 mg/kg, SC, once weekly for four weeks) at initiation and mid-way thru ART. Additionally, this group received M-IFNa-IgFc (500,000 IU, SC, once weekly for five weeks) prior to ART interruption. Upon ART discontinuation, the eight IL-21-treated RMs received PEGylated-IFNa-2a (PEGASYS), 7 mg/kg, SC, once weekly for seven weeks; while the remaining seven RMs were ART-treated controls. Blood (PB), lymph nodes (LN) and colorectal (RB) biopsies were longitudinally collected to assess the effects of IL-21 and IFNa on inflammation, T-cell subsets and viral persistence.

**Results**: ART fully suppressed plasma viremia (<30 RNA copies/mL) in all RMs. During ART, IL-21 reduced levels of activated (HLA-DR^+^CD38^+^) and proliferating (Ki-67^+^) T cells in PB, RB and LN in comparison to ART-only controls (*p* < 0.01). Levels of inflammation remained significantly lower also during and after addition of IFNa (*p* < 0.01). Upon ART interruption, IL-21/IFNa-treated RMs exhibited delayed viral rebound with a median of 21 days as compared to 9 days in the controls (*p* = 0.0009). Moreover, IL-21/IFNa-treated RMs maintained reduced viremia in comparison to controls up to 45 days after ATI (*p* = 0.0004).

**Conclusions**: These data support the safety of a combined IL-21 and IFNa treatment for HIV infection. While IL-21-treatment effectively reduces inflammation, addition of IFNa prior- and after-ART discontinuation resulted in a prolonged and more effective control of viral rebound. The synergy of such therapeutics may promote reinvigoration of host responses toward reduction of latent HIV reservoirs.

## TUPDA0102

### Interleukin (IL)-27 induces HIV resistance in T cells and dendritic cells via different mechanisms: identification of YB-1 and ANKRD22 as novel host dependency factors

D Poudyal^1^; B Sowrirajan^1^; Q Chen^1^; J Adelsberger^2^; M Bosche^1^; J Yang^1^; HC Lane^3^ and T Imamichi ^1^

^1^Leidos Biomedical Research, Inc., Frederick National Laboratory for Cancer Research, Laboratory of Human Retrovirology and Immunoinformatics, Frederick, USA. ^2^Leidos Biomedical Research, Inc., Frederick National Laboratory for Cancer Research, Frederick, USA. ^3^NIH, NIAID, Bethesda, USA

Presenting author email: timamichi@mail.nih.gov

**Background**: We have reported that IL-27 inhibits HIV replication in macrophages, T cells and dendritic cells (DC) and proposed that IL-27 is a potent novel anti-viral cytokine. We have also demonstrated that IL-27 induces HIV resistance in macrophages via downregulating SPTBN1 (Spectrin-beta chain brain 1) expression; however, the mechanism whereby IL-27 induces HIV resistance in T cells or DC has not been described.

**Methods**: T cells and monocytes were isolated from healthy donor PBMCs. T cells were stimulated with PHA. Monocytes were differentiated to DC in the presence of IL-4 and granulocyte macrophage colony-stimulating factor (GM-CSF) with or without IL-27. HIV replication was monitored using a commercial p24 ELISA kit. CD4 and chemokine receptor expression were analysed by FACS. Post-translational modification (PTM) of proteins were analysed using 2-Dimentional Difference in Gel analysis (2D-DIGE) and Western blotting. Genes of interest were knocked down using siRNA.

**Results**: IL-27-treated PHA-stimulated T cells (27-T) and IL-27-induced DC (27-DC) resisted HIV infection by 70% and >90%, respectively, without significant change in the expression of CD4, CCR5 or CXCR4. To define the mechanism of these anti-HIV effects, microarray and Western blotting were performed. In 27-T cells, four genes were upregulated by >3-fold and no genes were significantly downregulated by >2-fold in microarray analysis of ~20,000 genes. 2D-DIGE revealed that the amounts of PTM Y box binding protein 1 (YB1: a Y-box transcription factor) was diminished in 27-T by 60%. Knockdown of YB-1 in control T cells led to 70% resistance to HIV infection. In 27-DC, over 100 genes were upregulated or downregulated by >2-fold. A series of qPCR and Western blot analyses confirmed that the expression of ANKRD22 (ankyrin repeat domain 22) was consistently induced in 27-DC. Knock down of ANKRD22 reversed resistance to HIV infection in 27-DC. These results indicated that IL-27-induced HIV resistance in T cells and DCs by a decrease in PTM-YB1 and an increase in ANKRD22, respectively.

**Conclusions**: PTM-YB-1 and ANKRD22 were identified as novel host dependency factors in T cells and DC, respectively. Taken together with our previous report, these data demonstrate that IL-27 induces HIV resistance in macrophages, T cells and DC in cell-type-dependent manners.

## TUPDA0103

### Increased effector cytotoxic lymphocytes in lymph nodes of hetIL-15 treated macaques suggest potential to disrupt SIV/HIV reservoirs

A Valentin^1^; E Moysi^1^; DC Watson^1^; C Petrovas^2^; C Bergamaschi^1^; BK Felber^1^ and GN Pavlakis ^1^

^1^National Cancer Institute, Frederick, USA. ^2^VRC, NIAID, Bethesda, USA

Presenting author email: george.pavlakis@nih.gov

**Background**: Heterodimeric interleukin-15 (hetIL-15) activates and expands cytotoxic T and NK cells, which suggests that the cytokine could be useful for the treatment of malignancies and HIV infection. Based on these properties, hetIL-15 is currently evaluated in humans for the treatment of cancer. We also study the effects of hetIL-15 in infected macaques to evaluate its use in HIV infection.

**Methods**: Twelve rhesus macaques received six subcutaneous injections of hetIL-15 over two weeks using increasing doses of cytokine (step-dosing). At the end of the treatment, the animals were sacrificed and the hetIL-15 effects on different lymphocyte populations isolated from tissues collected at necropsy were monitored by multi-parametric flow cytometry and quantitative multiplexed confocal microscopy (Histo-cytometry).

**Results**: This protocol was safe in rhesus macaques and resulted in systemic expansion (Ki67+) of CD8 + T lymphocytes and NK cells with higher granzyme B content. These expanded cell populations were found in both effector sites, such as liver, vagina and rectum, and secondary lymphoid tissues. Among the gut-resident CD8 + T lymphocytes, we found a gradient, based on anatomical location, of the IL-15-induced T-cell proliferation, which follows the proliferation pattern found in untreated animals. Importantly, a significant increase in cytotoxic effector memory CD8 + T cells was found in lymph nodes from all hetIL-15-treated macaques, and this increase was bigger than that in SIV-infected animals. CM9 tetramer staining demonstrated that the increase of CD8+ effector T cells in lymphoid organs included actively proliferating SIV-specific T cells with higher granzyme content. Imaging analysis by Histo-cytometry revealed that these effector CD8 + T cells infiltrated the B-cell follicles where chronically infected follicular helper CD4 + T cells are located.

**Conclusions**: Step-dose administration of hetIL-15 is a well-tolerated regimen that results in systemic activation and expansion of cytotoxic leukocytes that infiltrate areas where chronic HIV-infected cells reside. These results suggest that hetIL-15 could be useful in disrupting or eliminating long-term viral reservoirs in HIV-1-infected individuals, thus contributing to a functional cure of the infection. Work assessing the long-term impact of hetIL-15 on the size of the viral reservoir is currently ongoing.

## TUPDA0104

### The human IL-15 superagonist ALT-803 activates NK and memory T cells, reactivates latent SIV and drives SIV-specific CD8 + T cells into B-cell follicles

G Webb^1^; S Li^2^; J Greene^3^; J Reed^1^; J Stanton^1^; A Legasse^1^; B Park^1^; M Axthelm^1^; E Jeng^4^; H Wong^4^; J Whitney^5^; B Jones^6^; D Nixon^6^; P Skinner^2^ and J Sacha ^1^

^1^Oregon Health and Science University, Beaverton, USA. ^2^University of Minnesota, St. Paul, USA. ^3^Oregon Health and Science University, Beaverton, USA. ^4^Altor Bioscience Corporation, Miramar, USA. ^5^Harvard Medical School, Boston, USA. ^6^The George Washington University, Washington, USA

Presenting author email: sacha@ohsu.edu

**Background**: There is an urgent need to develop alternate approaches to activate and clear the latent HIV reservoir that do not negatively impact immune function and are independent of viral sequence. IL-15 is a key cytokine for homeostatic maintenance, proliferation and expansion of memory CD4 + T cells, the primary HIV cellular reservoir. Here, we explored the human IL-15 superagonist, ALT-803, as an immunostimulatory molecule and potential latency reversing agent (LRA) in cART-suppressed SIV-infected macaques.

**Methods**: SIV-naïve and SIV-infected macaques were administered ALT-803 IV and subsequently monitored for NK and T-cell proliferation. SIV-infected macaques treated with ALT-803 were assessed for intrafollicular migration via *in situ* staining of lymph nodes with MHC class I tetramers. ALT-803 was tested as an LRA *in vitro* with primary CD4 + T cells from cART-suppressed macaques, and *in vivo* in SIV-infected, cART-suppressed macaques.

**Results**: ALT-803 activated and induced robust proliferation in NK cells and in both effector and central memory T cells. ALT-803 redirected activated cells to secondary lymphoid tissues, a known anatomical location of the viral reservoir, and *in situ* with MHC class I tetramer staining showed increased migration into B-cell follicular sanctuaries. ALT-803 did not affect viral loads in macaques with uncontrolled SIV infection; instead, ALT-803 potentiated low-level viral replication in elite controllers. In experiments using CD4 + T cell from cART-suppressed macaques, ALT-803 induced robust viral replication *in vitro*. When administered to macaques with cART suppression of plasma viremia, ALT-803 treatment resulted in plasma viral “blips” and unlike previous reports of other common g-chain cytokines like IL-7, ALT-803 did not cause an increase in the size of the latent viral reservoir.

**Conclusions**: ALT-803, an IL-15 superagonist, is well tolerated in SIV-infected, cART-suppressed macaques and induces virus reactivation both *in vitro* and *in vivo*. In addition to reactivating quiescent virus, ALT-803 potently activates NK and memory CD8 + T cells, which traffic to lymph nodes, specifically entering B-cell follicles where latently infected CD4 + T follicular helper cells reside. The ability of ALT-803 to potentially mediate both the “shock” and “kill” make it an appealing candidate for further studies aimed at durable cART-free HIV remission.

## TUPDA0105

### Tissue factor pathway inhibitor Ixolaris improves disease outcome in progressive SIVsab-infected pigtail macaques

T He ^1,2^; M Schechter^3^; B Andrade^4,5,6^; GH Richter^1^; B Policicchio^1,7^; K Raehtz^1,8^; E Brocca-Cofano^1,2^; C Apetrei^1,8^; R Tracy^9^; R Ribeiro^10^; I Francischetti^11^; I Sereti^12^ and I Pandrea^1,2^

^1^University of Pittsburgh School of Medicine, Center for Vaccine Research, Pittsburgh, USA. ^2^University of Pittsburgh School of Medicine, Pathology, Pittsburgh, USA. ^3^Frederick National Laboratory for Cancer Research, Clinical Research Directorate/Clinical Monitoring Research Program, Leidos Biomedical Research, Inc., Frederick, USA. ^4^National Institutes of Health, National Institute of Allergy and Infectious Diseases, Immunobiology Section, Laboratory of Parasitic Diseases, Bethesda, USA. ^5^Instituto Gonçalo Moniz, Fundação Oswaldo Cruz, Salvador, Brazil. ^6^Instituto Brasileiro para a Investigação da Tuberculose, Fundação José Silveira, Multinational Organization Network Sponsoring Translational and Epidemiological Research (MONSTER) Initiative, Salvador, Brazil. ^7^Department of Infectious Diseases and Microbiology, University of Pittsburgh Graduate School of Public Health, Pittsburgh, USA. ^8^Department of Microbiology and Molecular Genetics, University of Pittsburgh School of Medicine, Pittsburgh, USA. ^9^Department of Pathology & Laboratory Medicine, The Robert Larner, M.D. College of Medicine University of Vermont, Burlington, USA. ^10^Los Alamos National Laboratory, Theoretical Biology and Biophysics, Theoretical Division, Los Alamos, USA. ^11^National Institutes of Health, National Institute of Allergy and Infectious Diseases, Laboratory of Malaria and Vector Research, Bethesda, USA. ^12^National Institutes of Health, National Institute of Allergy and Infectious Diseases, Laboratory of Immunoregulation, HIV Pathogenesis Section, Bethesda, USA

**Background**: Despite viral suppression with antiretroviral therapy, HIV-infected subjects face high risk of non-AIDS comorbidities, which is often associated with elevated immune activation and inflammation (IA/INFL) and hypercoagulability. Tissue factor (TF) may bridge IA/INFL and hypercoagulation via protease-activated receptor signalling. We hypothesized that a TF pathway inhibitor can reduce the hypercoagulation and IA/INFL associated with HIV/SIV infection, and thus improve the disease outcome.

**Methods**: We compared the dynamics of TF expression on monocyte isolated from progressive (pigtail macaques, PTMs) versus non-progressive (African green monkeys, AGMs) models of SIVsab infection to establish its role in SIV pathogenesis. We tested the specificity and potency of a new TF inhibitor, Ixolaris, *ex vivo* on non-human primate (NHP) plasma and monocytes stimulated with LPS. Ixolaris was administered to five SIVsab-infected PTMs, with three SIV-infected, untreated PTMs as controls. IA/INFL markers (CD38/HLA-DR and proinflammatory cytokines) and hypercoagulation markers (D-dimer) were monitored throughout Ixolaris administration in treated and control PTMs.

**Results**: Monocyte TF expression increased postinfection in PTMs while it remained at baseline levels in chronically infected AGMs, confirming the role of TF in exacerbating SIV pathogenesis. Ixolaris specifically inhibited the extrinsic coagulation pathway and strongly inhibited TF activity by monocytes stimulated with LPS. *In vivo* administration of Ixolaris resulted in significantly lower inflammation (IL-17) and immune activation (HLA-DR^+^- and CD38^+^-expressing CD4^+^ and CD8^+^ T cells) during early chronic infection in treated SIVsab-infected PTMs compared to controls. Ixolaris also reduced hypercoagulation levels (D-dimer) in acutely SIVsab-infected PTMs. While viral loads and CD4 counts were minimally impacted by treatment, Ixolaris improved PTM survival, with no PTMs developing disease during the first 100 days postinfection, which is rarely seen in untreated SIVsab-infected PTMs.

**Conclusions**: Our results support a role of TF pathway in promoting disease progression and cardiovascular comorbidities in SIV-infected NHPs. *In vivo* TF inhibition by Ixolaris resulted in reduced IA/INFL and hypercoagulation in SIVsab-infected PTMs independent of CD4 counts and plasma viremia and improved the outcome of the SIVsab infection. Therefore, targeting TF pathway in HIV-infected subjects may represent an effective approach to prevent the deleterious consequences of HIV infection.

## TUPDA0106

### Engineering HIV immunity with a chimeric antigen receptor in the non-human primate model

A Zhen^1^; C Peterson^2^; M Carrillo^1^; V Rezek^1^; M Kamata^1^; J Zack^1^; H-P Kiem^2^ and S Kitchen ^1^

^1^David Geffen School of Medicine at UCLA, UCLA AIDS Institute, Los Angeles, USA. ^2^Fred Hutchinson Cancer Research Center, Seattle, USA

Presenting author email: skitchen@ucla.edu

**Background**: HIV-1-specific cytotoxic T lymphocytes are crucial for the elimination of HIV-infected cells. We have previously demonstrated using humanized mice that hematopoietic stem cells (HSCs) modified with a protective CD4 chimeric antigen receptor (CD4CAR) successfully differentiated into CD4CAR expressing T cells that significantly suppressed HIV replication. These results demonstrated the feasibility of engineering HIV-specific T-cell immunity with a HSC-based approach.

**Methods**: We tested the safety and feasibility of engineering T-cell immunity via HSC in a non-human primate (NHP) model of SHIV infection. We utilized CD4CAR vectors that also carry an anti-HIV protective gene (C46) that would inhibit infection. Two pigtailed macaques (*Macaca nemestrina)* were transplanted with C46CD4CAR-modified autologous HSC, and two were transplanted with HSC modified with control vector C46CD4CARdeltaZeta that lacks the signalling Zeta chain. After hematopoietic recovery, the animals were challenged with SHIV and went through combined anti-retroviral drug treatment and withdrawal. Animals were monitored for viral load, CAR cell detection and immune function for over a year.

**Results**: We determined that engraftment of pigtailed macaques with C46CD4CAR-modified HSCs was safe and the animals had normal transplant recovery. We observed long-term engraftment and stable production of C46CD4CAR expressing cells without any significant toxicities and found that C46CD4CAR-modified HSCs could differentiate into multiple hematopoietic lineages. Following challenge of the animals with SHIV, we observed significant expansion of C46CD4CAR expressing cells after infection and wide distribution of CAR-expressing cells in multiple lymphoid tissues. Importantly, we found lower viral loads in lymphoid tissues in C46CD4CAR-containing animals than in control animals, suggesting viral suppression by C46CD4CAR-expressing cells.

**Conclusions**: This study in NHPs demonstrates the safety and feasibility of a HSC-based therapy utilizing a HIV-specific chimeric antigen receptor for treating chronic HIV infection. These results set the stage for future investigational new drug (IND) development to eradicate viral infection and provide more effective immune surveillance of HIV.

## TUPDB0101

### Dynamics of HIV-1 DNA in children over long-term sustained viral suppression: impact of the time of infection at viral control on reservoir size

M Moragas ^1^; M Distefano^1^; D Mecikovsky^2^; S Arazi Caillaud^2^; R Bologna^2^; P Aulicino^1^ and A Mangano^1^

^1^Hospital de Pediatría “Prof. Dr. Juan P. Garrahan”, Laboratorio de Biología Celular y Retrovirus-CONICET, Unidad de Virología y Epidemiología Molecular, Ciudad de Buenos Aires, Argentina. ^2^Hospital de Pediatría “Prof. Dr. Juan P. Garrahan”, Servicio de Epidemiología e Infectología, Ciudad de Buenos Aires, Argentina

Presenting author email: matias.moragas@gmail.com

**Background**: The dynamics of HIV-1 DNA reservoir in perinatally infected children throughout long-term sustained viral suppression (VS) – and how they are affected by the time of infection at VS – have not been defined. Improved understanding of HIV-1 dynamics is necessary for therapeutic interventions that aim to achieve sustained antiretroviral therapy-free HIV-1 remission.

**Methods**: This study included 37 perinatally HIV-1 infected children on suppressive antiretroviral therapy. Children were grouped according to the timing of antiretroviral therapy (ART) initiation (≤0.5 or >0.5 years) and the time to achieve VS (≤1.5, between >1.5 and 4, and >4 years). Decay of cell-associated HIV-1 DNA (CA-HIV-1 DNA) level -quantified by semi-nested real time PCR- and 2-long terminal repeats (2-LTR) circles frequency -detected by hemi-nested real time PCR- were analysed over 4 years of VS using piecewise linear mixed-effects model with two splines and logistic regression, respectively.

**Results**: CA-HIV-1 DNA in peripheral blood mononuclear cells (PBMC) had a high decay during the first two years of VS [−0.26 (95% CI: −0.43 to −0.09) log_10_ copies per one million (cpm) PBMC/year], followed by a slow decay and reaching a plateau between 2 and 4 years of VS [−0.06 (95% CI: −0.15 to 0.55) log_10_ cpm PBMC/year]. The level of CA-HIV-1 DNA in PBMCs highly correlated with those estimated in CD4^+^ T cells (*r* = 0.97, *p* < 0.0001) and whole blood (*r* = 0.98, *p* < 0.0001). The initial decay according to time of infection at VS was −0.51 (95% CI: −0.94 to −0.07), −0.35 (95% CI: −0.83 to 0.14) and −0.21 (95% CI: −0.39 to −0.02) log_10_ cpm PBMC/year for children who achieved VS by 1.5, >1.5–4 and >4 years of infection, respectively. The frequency of 2-LTR circles decayed significantly, from 82.9% at pre-VS to 37.5% and 28.1% at two and four years of VS, respectively (*p* = 0.0009).

**Conclusions**: A marked decay of HIV-1 DNA occurs during the first two years of viral suppression – where the earlier the time of infection at viral suppression, the higher the rate of decay – and seems to set HIV-1 reservoir size. After two years, HIV-1 DNA decreases slowly and independently of the time to achieve effective viral control.

## TUPDB0102

### Impact of T-cell homeostasis and thymic output on the seeding of the HIV reservoir in infants

M Massanella ^1,2^; J Ananworanich^3,4^; L Leyre^1,2^; T Jupimai^5^; P Sawangsinth^5^; M de Souza^3^; P Suntarattiwong^6^; P Kosalarksa^7^; T Puthanakit^8,9^; N Chomont^1,2^; HIVNAT209 and HIVNAT194 study groups

^1^Centre de Recherche du CHUM, CR-CHUM, Montréal, Canada. ^2^Department of Microbiology, Infectiology and Immunology, Université de Montréal, Montréal, Canada. ^3^SEARCH, Thai Red Cross AIDS research Centre, Bangkok, Thailand. ^4^Henry M. Jackson Foundation for the Advancement of Military Medicine, Bethesda, USA. ^5^HIV Netherlands Australia Thailand (HIV-NAT) Research Collaboration, Thai Red Cross AIDS Research Center, Bangkok, Thailand. ^6^Queen Sirikit National Institute of Child Health, Bangkok, Thailand. ^7^Department of Pediatrics, Faculty of Medicine, Khon Kaen University, Khon Kaen, Thailand. ^8^Chulalongkorn University, Research Unit in Pediatric Infectious Diseases and Vaccines, Bangkok, Thailand. ^9^Department of Pediatrics, Faculty of Medicine, Chulalongkorn University, Bangkok, Thailand

Presenting author email: marta.massanella.luna@umontreal.ca

**Background**: Early antiretroviral therapy (ART) limits the size of the HIV reservoir and immune activation levels in adults; however paediatric data are limited. We evaluated the impact of immune parameters on the size of the HIV reservoir in early treated vertically infected infants.

**Methods**: Virologically non-suppressed HIV-infected Thai infants (*n* = 12, <2 months old) and fully suppressed children (for >1 year) who started ART within the first six months of life (*n* = 57, median 4 years old) were included. We quantified total HIV DNA in CD4 T cells by RT-PCR. Immune activation and proliferation markers in CD4 and CD8 naïve, stem cell, central, transitional and effector memory T cells as well as frequencies of effector, recent thymic emigrants (RTE) and T-regulatory (Treg) cells were assessed by flow cytometry.

**Results**: High frequencies of naïve CD4^+^ T cells were observed in non-suppressed and ART-treated children (median 78 and 64, respectively). Interestingly, the frequency of RTE was significantly increased in ART-treated children despite their older age (median 81 vs. 89, *p* = 0.009), suggesting that HIV replication in non-suppressed infants may limit thymic production. Frequencies of T cells undergoing proliferation (Ki67^+^) were significantly lower in CD4^+^ (median 4.8 vs. 3.6, *p* = 0.04) and CD8^+^ (median 29 vs. 4.6, *p* < 0.0001) T cells from ART-treated children. Similarly, frequencies of activated CD8^+^ T cells (HLA-DR^+^CD38^+^) were significantly lower in ART-treated children (median 29 vs. 4.6, *p* < 0.0001). High frequencies of Treg were observed in both groups (>5.1% of CD4^+^ T cells) with no significant differences. In non-suppressed infants, the frequency of cells harbouring HIV DNA was negatively correlated with the frequency of RTE (*r* = −0.9, *p* = 0.01) and positively correlated with the frequency of memory Treg cells (CD45RA^−^CD27^−^CD25^bright^FOXP3^+^, *r* = 0.7, *p* = 0.04). Similarly, HIV DNA levels in ART-treated children negatively correlated with RTE (*r* = −0.3, *p* = 0.02) and positively correlated with Treg (*r* = 0.3, *p* = 0.04).

**Conclusions**: The high frequencies of RTE resulting from enhanced thymic production in paediatric population may limit the establishment and persistence of the HIV reservoir. In contrast, the positive association between the frequency of HIV-infected cells and Treg suggests that this subset may play a prominent role in the establishment of the HIV latent reservoir during infancy.

## TUPDB0103

### Prolonged HIV-1 remission and viral rebound in an individual treated during hyperacute infection

T Henrich ^1^; H Hatano^1^; A Hill^2^; O Bacon^1^; M Kearney^3^; J Blankson^4^; S Cohen^1^; M Abdel Mohsen^1^; R Fromentin^5^; J Spindler^3^; K Metcalf-Pate^6^; R Siliciano^6^; D Richman^7^; N Chomont^5^; J Siliciano^6^; J Mellors^8^; T Liegler^1^ and S Deeks^1^

^1^University of California, San Francisco, San Francisco, USA. ^2^Harvard University, Cambridge, USA. ^3^National Cancer Institute, Frederick, USA. ^4^Johns Hopkins School of Medicine, Baltimore, USA. ^5^University of Montreal, Montreal, Canada. ^6^Johns Hopkins University, Baltimore, USA. ^7^University of California, San Diego, San Diego, USA. ^8^University of Pittsburgh, Pittsburgh, USA

Presenting author email: timothy.henrich@ucsf.edu

**Background**: It is unknown if extremely early initiation of ART may be curative. We describe an individual who started ART an estimated 10 days after infection with plasma HIV RNA of 220 copies/ml. After extensive blood and tissue sampling, he underwent an analytical treatment interruption (ATI) following 34 months of ART.

**Methods**: Colorectal and lymph node tissues, bone marrow, cerebral spinal fluid (CSF), plasma and large numbers of PBMC obtained longitudinally were studied for HIV persistence in several laboratories using molecular and culture-based detection methods including a humanized mouse outgrowth assay.

**Results**: The individual initiated PrEP (TDF/FTC) during very early Fiebig stage I (HIV-1 RNA+ EIA-) followed by ART intensification (TDF/FTC/r/DRV/RAL) 8 days later. HIV RNA levels were 120 and <40 copies/mL, 7 and 22 days after PrEP initiation, respectively, followed by no detectable level. Low-level cell-associated HIV RNA (3.2 copies/million CD4+ T cells) was detected 32 days after infection. Over the following two years, no further HIV could be detected, despite massive sampling from ileum, rectum, lymph nodes, bone marrow, CSF, CD4 + T-cell subsets and plasma. 530 million CD4 + T cells were also assayed in a humanized mouse outgrowth assay. One mouse (53 million input cells) experienced very low-level viremia (201 copies/mL) after 5.5 weeks, but sequence confirmation was unsuccessful. The participant stopped ART and remained aviremic for 7.4 months, rebounding with HIV RNA of 36 copies/mL that rose to 59,805 copies/mL 6 days later. ART was restarted promptly. Rebound plasma HIV sequences were identical to those obtained during acute infection by single-genome sequencing. Mathematical modelling predicted that the latent reservoir size was approximately 200 cells prior to ATI and that only around 1% of individuals with a similar HIV burden may achieve lifelong ART-free remission.

**Conclusions**: We report HIV relapse despite initiation of ART at one of the earliest stages of acute HIV infection possible. Near complete or complete loss of detectable HIV in blood and tissues did not lead to indefinite ART-free HIV remission. However, the small numbers of latently infected cells in individuals treated during hyperacute infection may be associated with prolonged ART-free remission.

## TUPDB0104

### HIV RNA persists in rectal tissue despite rapid virologic suppression in blood plasma with dolutegravir-based combination antiretroviral therapy in treatment-naïve patients

C Lahiri ^1^; N Brown^1^; H Chien^1^; A Vunnava^1^; K Ryan^2^; E Acosta^2^; A Sheth^1^; J Ingersoll^3^ and I Ofotokun^1^

^1^Department of Medicine, Emory University School of Medicine, Atlanta, USA. ^2^Department of Pharmacology and Toxicology, University of Alabama Birmingham, Birmingham, USA. ^3^Emory University, Virology and Molecular Biomarkers Core, Atlanta, USA

Presenting author email: cdelill@emory.edu

**Background**: Despite virologic suppression in blood plasma (BP) with combination antiretroviral therapy (cART), HIV eradication remains elusive, largely due to incomplete suppression in reservoir sites including gut-associated lymphoid tissue. We compared HIV RNA dynamics within BP and rectal tissue (RT) following initiation of cART with integrase strand transfer inhibitor dolutegravir (DTG).

**Methods**: Treatment-naïve HIV-positive volunteers began DTG-based (50 mg daily) cART and serial sampling of BP and RT through 84 days as per Figure 1. HIV RNA and DTG concentrations were quantified using Abbott Real-Time HIV-1 assays and tandem mass spectroscopy, respectively, in both compartments. Median DTG concentrations were compared between undetectable and detectable RT HIV RNA groups using Mann–Witney non-parametric tests.

**Results**: Eight participants were enrolled: 4 (50%) females, 6 (75%) black, median age 39.2 years (IQR 32.9–52.7). Median baseline CD4 and HIV RNA were 208 cells/mm^3^ (IQR 104–320) and 24,847 copies/mL (IQR 6237–50,269), respectively. All participants (100%) had undetectable BP HIV RNA (<40 copies/mL) by Day 42; only three achieved RT viral suppression at any time (Figure 1). DTG quantitation was performed on 22 paired BP and RT steady-state samples collected Days 7 through 84. The undetectable RT RNA group had higher median DTG RT concentrations than those with detectable RNA: 1340 ng/g (IQR 683–2100) versus 624 ng/g (IQR 377–939), *p* = 0.03. There were no significant differences in BP DTG or RT:BP DTG ratios between undetectable and detectable groups: median 2810 ng/mL (IQR 1720–4140) versus 1400 ng/mL (IQR 687–3250) and median 0.45 (IQR 0.30–0.55) versus 0.40 (IQR 0.28–0.69), respectively.

**Abstract TUPDB0104–Figure 1.**
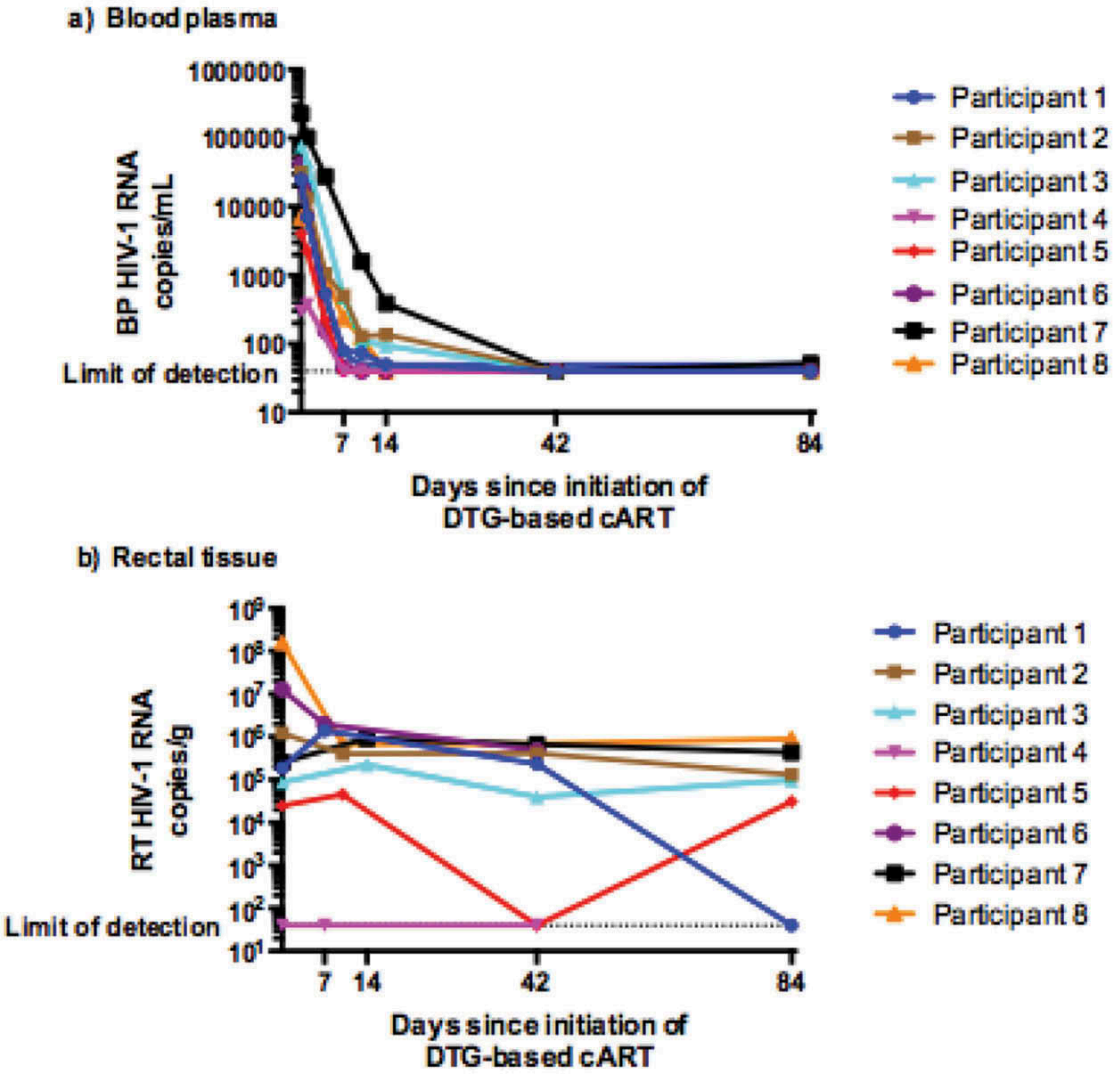
HIV RNA over time following initiation of DTG-based cART.

**Conclusions**: Despite rapid virologic suppression in BP, HIV RNA persisted in RT for the majority of participants. Along with larger studies examining DTG compartmental pharmacokinetic/pharmacodynamic relationships, assessment of the infective potential of HIV RNA recovered from RT are warranted, as this may have implications for ongoing rectal transmission notwithstanding plasma virologic suppression.

## TUPDB0105

### Viral rebound in semen after treatment interruption in a HIV therapeutic vaccine double-blind trial (VRI02/ANRS149-LIGHT)

J Ghosn ^1^; R Palich^2^; A Chaillon^3,4^; V Boilet^5,6^; M-L Néré^2^; P Delobel^7^; F Lutch^8^; O Bouchaud^9^; J-M Molina^10^; M-L Chaix^2,4^; C Delaugerre^2,4^; V Rieux^4,11^; R Thiebaut^5,6^; Y Lévy^4,12,13^ and J-D Lelièvre^4,12,13^

^1^Hotel-Dieu University Hospital Center, University of Paris Descartes, Therapeutic, Immunology and Infectious Disease, Paris, France. ^2^Saint-Louis University Hospital Center, University of Paris Diderot, Virology, U941, Paris, France. ^3^University of California San Diego, Medicine, San Diego, USA. ^4^Vaccine Research Institute – VRI, Paris, France. ^5^INSERM U1219, ISPED, Bordeaux Population Health, Bordeaux, France. ^6^Vaccine Research Institute – VRI, CMG, Bordeaux, France. ^7^Toulouse-Purpan University Hospital Center, Infectious Diseases, Toulouse, France. ^8^Saint-Etienne University Hospital Center, Infectious Diseases, Saint-Etienne, France. ^9^Avicenne University Hospital Center, Infectious Diseases, Paris, France. ^10^Saint-Louis University Hospital Center, University of Paris Diderot, Infectious Diseases, U941, Paris, France. ^11^French National Agency for Research on AIDS and Viral Hepatitis (ANRS), Paris, France. ^12^INSERM U955, IMRB, Equipe 16, Créteil, France. ^13^Henri Mondor University Hospital Center, Créteil, France

Presenting author email: jade.ghosn@aphp.fr

**Background**: Antiretroviral treatment interruption (ATI) leads to HIV replication rebound in both blood and semen; however dynamic of HIV-RNA rebound in semen is poorly known. We compared HIV-RNA timing of and level of rebound in blood and seminal plasmas (BP-SP) and characterized the HIV rebounding populations in both compartments after ATI in HIV-1-infected patients enrolled in the therapeutic vaccine trial VRI02/ANRS149-LIGHT.

**Methods**: Ten male from the VRI02/ANRS149-LIGHT trial with CD4 cells ≥600/mm^3^ and HIV-RNA <50 copies/ml under treatment for at least 18 months were studied. ATI occurred from week (W)36 to W48. Paired blood and semen samples were collected at W32 (before ATI), W36, W38, W40, W42, W44 and W48 following ATI, for HIV-RNA and DNA quantification. Ultradeep sequencing (UDS, 454/Roche) of partial *env* (C2/V3) HIV-DNA and -RNA was performed in 5 out of 10 participants in PBMC before ATI and in plasma/semen during ATI. Sequenced reads were quality filtered and assembled using an in-house data processing pipeline.

**Results**: Patients had sustained suppressed blood viral load for a median of 44 months (range: 28–152) before ATI. HIV-RNA rebounded in blood and semen in all patients after ATI (median 5.12 log_10_ cp/ml (range: 4.61–6.35) and 4.26 log_10_ cp/ml (range: 3.20–4.67), respectively). BP HIV-RNA rebounded as soon as W38 in 8/10 patients, and SP HIV-RNA between W38 and W40 in 8/10 patients. HIV-DNA median level was 2.97 log_10_ cp/10^6^ PBMC (range: 1.61–3.26) at W32 and 3.30 log_10_ cp/10^6^ PBMC (range: 2.50–3.67) at W44. Non-sperm cells HIV-DNA was detected in at least one sample in 6/10 patients. Phylogenetic analysis of UDS revealed that (1) rebounding HIV-RNA population in BP and in SP originated from PBMC HIV-DNA at the time of ATI and (2) intermingled HIV-RNA populations in BP and in SP with no evidence of compartmentalization.

**Conclusions**: The rapid and intense HIV-RNA rebound observed very early both in blood and semen after ATI emphasizes the need for targeted prevention strategies to reduce the risk of sexual transmission during ATI. PBMC HIV-DNA is the major contributor for HIV-RNA rebound in both compartments, even after several years of sustained suppressed viral replication.

## TUPDC0101

### Transgender women willingness to use PrEP in north-eastern Brazil

F Soares^1^; I Dourado ^2^; L Magno^3^; LA Vasconcelos da Silva^4^; A Nunn^5^; PopTrans Study Group

^1^Federal University of Bahia, Institute of Collective Health, Salvador, Brazil. ^2^Federal University of Bahia, Health Collective Institute, Salvador, Brazil. ^3^State University of Bahia, Nursing, Salvador, Brazil. ^4^Federal University of Bahia, Institute of Humanities, Arts and Sciences Professor Milton Santos, Salvador, Brazil. ^5^Brown University, School of Public Health, Providence, USA

Presenting author email: ines.dourado@gmail.com

**Background**: PrEP can dramatically reduce HIV acquisition risks, particularly for transgender women and other sexual and gender minorities. However, access to PrEP in developing countries remains very limited. Brazil has one of the largest and oldest HIV treatment programmes in the world and will soon integrate PrEP in the national public health system. We explored PrEP willingness among transgender women.

**Methods**: We recruited 127 transgender women using *Respondent Driven Sampling* in Salvador, Brazil’s third largest – and one of its lowest-income and highest afro-descendants- cities. Participants were interviewed about PrEP. Latent class analyses were used to identify those willing to take PrEP from the following list of variables: 1 – disposition to use PrEP; 2 – willingness to use PrEP if available in the public health system; 3 – willingness to use PrEP even if had to pay; 4 – interest in using PrEP even if it is not 100% effective; 5 – being less afraid of contracting HIV if using PrEP; 6 – willingness to take one pill a day; 7 – knowledge about PrEP. Entropy was 0.992 indicating good distinction of classes.

**Results**: Only 18.4% of women knew about PrEP. However, upon becoming aware, willingness to use PrEP was reassuring. Two latent classes were identified: Class 1-willingness to use PrEP (91.3%); Class 2- non willingness to use PrEP (8.6%). Most participants noted that PrEP was an important HIV prevention tool for transgender women. Correlates of Latent Class 2 were: Socio-behavioural factors including not being black, having a monthly income greater than R$ 900.00 and regular use of condoms in sexual intercourse. When asked about implementation strategies, participants suggested integrating conversations about PrEP into a suite of HIV prevention services, addressing health system services that address the broader social and structural factors influencing transgender health risks.

**Conclusions**: PrEP willingness was very high as 91% of transgender women wanted it in Northeast Brazil. While access to PrEP is still limited, uptake among transgender women will likely be high when Brazil releases PrEP. However, it is important to take into account socio-behavioural factors and combination prevention as those regular users of condom may not see and additional benefit of PrEP.

## TUPDC0102

### How are transwomen acquiring HIV? Insights from phylogenetic transmission clusters

H-HM Truong ^1^; K O’Keefe^2^; S Pipkin^2^; T Liegler^1^; S Scheer^2^ and W Mc Farland^2^

^1^Department of Medicine, University of California, San Francisco, San Francisco, USA. ^2^Department of Public Health, San Francisco, USA

**Background**: Worldwide, HIV prevalence among transwomen is 50 times higher than in the general adult population. In many surveillance systems and surveys, transwomen and their male sexual partners are classified as “men who have sex with men” (MSM), irrespective of gender identity and sexual orientation. Little is known about how transwomen acquire HIV, which may be due in part to this misclassification as “MSM”. We sought insights on sexual and use of non-sterile needle networks as potential sources of HIV among transwomen by examining phylogenetic transmission clusters. We also assessed a new transmission risk paradigm that re-classifies males closely linked to transwomen as non-MSM.

**Methods**: San Francisco residents diagnosed with HIV (2000–2015), in care at public facilities and with available viral pol sequences were included in the analysis. Transmission clusters with ≥2 cases were identified by bootstrap values ≥90% and mean pairwise genetic distances ≤0.025%.

**Results**: Transwomen were 275 of 5200 cases with viral sequences; 86 transwomen were in 70 clusters; 44 (51%) had injection risk. Many clusters with transwomen contained MSM-persons who inject drugs (MSM-PWID) (47% of clusters) and non-MSM PWID (26%); whereas MSM were in 54% of clusters and heterosexual men in 1%. After re-classification, the profile of clusters shifted: 16% of clusters contained MSM-PWID, 57% had non-MSM PWID, 16% had MSM and 47% had heterosexual men. Similarly, among 130 clusters containing cis women, 27% had MSM-PWID, 41% had non-MSM PWID, 28% had MSM and 58% had heterosexual men.

**Conclusions**: Applying the new paradigm for classifying the transmission risk of transwomen and their partners suggests that transwomen may stand apart from the MSM epidemic. The risk profile of transwomen’s transmission clusters is highly sensitive to whether or not male partners are classified as MSM. Under the new paradigm, transmission clusters containing transwomen closely resemble clusters containing cis women, with a strong presence of PWID and heterosexual men. There is increasing recognition that transwomen should be considered by their gender identity for health services and research. The same consideration perhaps should apply to male sexual partners of transwomen. Examining transmission clusters may bring new insights to the applicability of MSM-focused research to transwomen and their partners.

## TUPDC0103

### Discrepancy between risky sexual behaviour and perceived HIV risk among transgender women in community-based test and treat cohorts in Thailand

AR Ramautarsing ^1^; T Nakpor^2^; R Janamuaysook^1^; S Pengnonyang^1^; J Jantarapakde^1^; D Trachunthong^1^; T Sungsing^1^; P Rodbamrung^1^; P Mingkwanrungruangkit^1^; W Sirisakyot^1^; S Tongmuang^1^; S Jarupittaya^1^; N Mahachokchai^2^; P Yokchawee^2^; S Samalu^3^; M Uthaikorn^3^; W Champa^4^; P Patpeerapong^5^; S Mills^6^; S Chareonying^6^; P Phanuphak^7^; M Cassell^8^; R Vannakit^8^ and N Phanuphak^1^

^1^Prevention Department, Thai Red Cross AIDS Research Center, Bangkok, Thailand. ^2^Rainbow Sky Association of Thailand, Bangkok, Thailand. ^3^The Service Workers In Group Foundation, Bangkok, Thailand. ^4^Caremat Organisation, Chiang Mai, Thailand. ^5^Mplus Foundation, Chiang Mai, Thailand. ^6^FHI 360 and USAID LINKAGES Project, Bangkok, Thailand. ^7^Thai Red Cross AIDS Research Center, Bangkok, Thailand. ^8^Office of Public Health, U.S. Agency for International Development Regional Development Mission Asia, Bangkok, Thailand

Presenting author email: reshmie@trcarc.org

**Background**: Transgender women (TG) are a high-burden population for HIV. Globally, their risk of HIV is 49 times higher compared to the general population, indicating that HIV prevention and care services for TG are critical and urgent. To understand the facilitators and barriers for TG to access HIV testing and antiretroviral therapy (ART) at community-based clinics, we studied socio-demographic characteristics, behavioural risk and knowledge and attitudes towards HIV and ART.

**Methods**: Thai TG aged ≥18 years were recruited from six community-based clinics in Thailand. Demographic characteristics, behavioural risk, HIV knowledge and attitudes towards ART were assessed using questionnaires. Trained community health-workers provided same-day result HIV testing and sexually transmitted infection (STI) screening, as well as CD4 testing and linkage to care for HIV-infected participants.

**Results**: Of 734 TG participants enrolled, 56.1% were between 18 and 25 years old. Only 15.7% had a college degree or higher, and 36.8% earned less than 280 USD monthly. Prevalence of any STI was 32% (syphilis 3.5%, chlamydia 23.3%, gonorrhoea 14.6%). Nobody (0.0%) reported a negative attitude towards people with HIV, 42.0% of participants knew that ART can reduce HIV transmission, and 65.4% thought all people with HIV should use ART. Almost 90% said they would start ART immediately if they were diagnosed with HIV. Multiple sexual partners in the last six months was reported by 53.5%. While 77.4% of TG reported unprotected sex in the last six months and 91.1% identified proper condom use as a manner of decreasing HIV-risk, only 17% perceived their HIV risk as high. Almost half (45.0%) had never tested for HIV. HIV prevalence was 9.0%, and among 66 HIV-infected participants, median (IQR) CD4 count was 406 (306–562), 84% initiated ART within two weeks of diagnosis.

**Conclusions**: Among these young Thai TG presenting for HIV-testing at community-based clinics, the prevalence of HIV and STIs was high. Attitudes towards HIV and ART were positive, and ART uptake was high. However, there was a remarkable discrepancy between risky sexual behaviour and perceived HIV risk. Our findings are crucial to informing HIV education and prevention programs targeting TG in Thailand.

## TUPDC0104

### Engaging transgender women of colour in HIV prevention: findings from mixed methods community-based research

T Poteat ^1^; M Malik^1^; A Wirtz^1^ and E Cooney^2^

^1^Johns Hopkins School of Public Health, Epidemiology, Baltimore, USA. ^2^Johns Hopkins Bloomberg School of Public Health, International Health, Baltimore, USA

Presenting author email: tpoteat123@gmail.com

**Background**: Transgender women (TW) across the globe are highly vulnerable to HIV. TW of colour (TWOC) in the United States are particularly burdened, with an estimated HIV prevalence over 50% in some studies. Effective HIV interventions are needed to prevent HIV acquisition and onward transmission and to improve health outcomes. We used mixed-methods approaches to understand how to develop and implement accessible, acceptable, and effective HIV interventions for TWOC.

**Methods**: Qualitative, in-depth key informant (KI) interviews (*n* = 20) were conducted with TW and health and social service providers. Interviewer-administered surveys and rapid HIV tests were implemented among TWOC (*n* = 46) in Baltimore, USA.

**Results**: Among TWOC, HIV prevalence was 48%, with 18% unaware of their HIV status and 33% who had not been tested for HIV in the last 12 months. History of sex work (78%) and condomless anal intercourse (47%) were high. Most participants had heard of PrEP (89%); of those, 78% knew where to get it and 23% had ever taken it. Of those who had ever taken PrEP (*n* = 9), 67% had done so in the prior 12 months. Despite low uptake, 81% of HIV-negative TWOC stated they would take PrEP if it was made available to them. Interviews elicited diverse reflections on how to better engage TWOC in HIV programmes. KIs emphasized the importance of embedding HIV services within social service programmes that are responsive to community needs (e.g., job readiness, mental health support, housing), to improve access and acceptability of HIV programmes for TWOC. KIs also recommended regular staff training in transgender competent care (e.g., using correct gender pronouns), recognizing that experiencing discrimination in health venues deters TWOC from future care seeking. Other suggestions included: offering services where TWOC regularly visit, hiring TWOC to lead programs, and doing tailored outreach and advertising.

**Conclusions**: Acceptable high-impact HIV prevention and care interventions for TWOC are urgently needed as evidenced by an elevated HIV prevalence and low PrEP uptake. Noting the disconnect between willingness and uptake of PrEP among TWOC, HIV prevention programmes could better bridge this gap by incorporating strategies voiced by TW and responding to identified access barriers.

## TUPDC0105

### Factors associated with HIV infection among transgender women in Cambodia: results from a national integrated biological and behavioural survey

S Yi ^1,2^; S Chhim^3^; P Chhoun^1^; S Tuot^1^; P Mun^4^; K Pal^1^ and C Ngin^1^

^1^KHANA Center for Population Health Research, Phnom Penh, Cambodia. ^2^Touro University California, Center for Global Health Research, Vallejo, USA. ^3^FHI 360 Cambodia, Phnom Penh, Cambodia. ^4^National Center for HIV/AIDS, Dermatology and STD, Phnom Penh, Cambodia

Presenting author email: siyan@doctor.com

**Background**: Transgender women are at high risk for HIV infection, and little is known about the burden of HIV infection and its related factors in this population worldwide. This study was conducted to examine factors associated with HIV infection among transgender women in Cambodia.

**Methods**: This cross-sectional study was conducted between December 2015 and February 2016 in the capital city of Phnom Penh and 12 HIV high-burden provinces. Respondent driven sampling was used to recruit sexually active transgender women aged 18 and over. A structured questionnaire was used for a behavioural survey, and rapid finger-prick HIV testing was performed using Determine™ antibody test. Multivariate logistic regression analysis was conducted to identify factors associated with HIV infection using STATA.

**Results**: A total of 1375 transgender women participated in the study with a mean age of 25.9 years (SD = 7.1). The overall HIV prevalence among this population was 5.9%. In multivariate logistic regression, participants living in urban areas were twice as likely to be HIV infected as those living in rural areas. Participants with primary education were 1.7 times as likely to be infected compared to those with high school education. HIV infection increased with age; compared to those aged 18–24, the odds of being HIV infected were twice among transgender women aged 25–34 and 2.8 times higher among those aged ≥35. Self-injection of gender affirming hormones was associated with a fourfold increase in the odds of HIV infection. A history of genital sores over the previous 12 months increased the odds of HIV infection by threefold. Transgender women with stronger feminine identity dressing up as a woman all the time were twice as likely to be HIV infected compared to those who did not dress up as a woman all the time. Having never used online services developed for transgender women was also associated with higher odds of being HIV infected.

**Conclusions**: Transgender women in Cambodia are at high risk of HIV. To achieve the goal of eliminating HIV in the country, effective combination prevention strategies focusing on the above risk factors among transgender women are urgently needed.

## TUPDC0106

### Prevalence and correlates of police harassment among transgender women in Jamaica: implications for HIV prevention and care

CH Logie ^1^; A Lacombe-Duncan^1^; K Levermore^2^; N Jones^2^; A Neil^2^; T Ellis^2^; Y Wang^1^ and PA Newman^1^

^1^University of Toronto, Toronto, Canada. ^2^Jamaica AIDS Support for Life, Kingston, Jamaica

Presenting author email: carmen.logie@utoronto.ca

**Background**: Criminalization of homosexuality limits access to HIV prevention and care. Little is known about police harassment targeting transgender women in contexts where homosexuality is criminalized, such as Jamaica. We examined prevalence of police harassment and its association with HIV infection and risk factors among transgender women in Jamaica.

**Methods**: We implemented a cross-sectional survey with transgender women in Kingston, Ocho Rios and surrounding areas in Jamaica using chain referral sampling. Unadjusted and adjusted logistic regression analyses were conducted to identify health (e.g. HIV status), intrapersonal (e.g. sex work), interpersonal (e.g. social support) and structural (e.g. transphobic violence) factors associated with ever experiencing police harassment perceived to be due to transgender identity.

**Results**: Participant (*n* = 137) mean age was 24.0 years (SD: 4.5; range 18–44); two-thirds (*n* = 92; 67.2%) lived in Kingston, and half (*n* = 71; 51.8%) reported sex work involvement. Three-quarters (*n* = 103; 75.7%) had received an HIV test; of these, one-quarter (*n* = 26; 25.2%) were HIV positive. Almost half (43.8%; *n* = 60) reported experiencing police harassment due to their transgender identity. Of participants with complete data (*n* = 127), 16.5% (*n* = 21) reported a history of incarceration due to transgender identity. Of those, 71.4% (*n* = 15) reported being incarcerated one to three times and 28.6% (*n* = 6) were incarcerated four to six times. In unadjusted analyses, police harassment was associated with sociodemographic (<high school education vs. ≥high school [OR: 3.04, 95% CI: 1.1–8.4]), health (HIV-positive serostatus [OR: 2.44, 95% CI: 1.01–5.86], depression [OR: 1.23, 95% CI: 1.01–1.50]), intrapersonal (sex work [OR: 2.61, 95% CI: 1.30–5.25]), interpersonal (higher need for social support [OR: 1.09, 95% CI: 1.03–1.15]) and structural (transphobic physical violence [OR: 6.97, 95% CI: 3.14–15.51]). In adjusted multivariable analyses, HIV positive serostatus (AOR: 2.96, 95% CI: 1.04, 8.43) and transphobic physical violence (OR: 6.06, 95% CI: 2.53, 14.55) maintained strong associations with experiences of police harassment.

**Conclusions**: Nearly half of transgender women in this Jamaican study reported police harassment, and this was associated with HIV-positive serostatus and physical violence. Criminalization and violence are structural drivers of HIV, constraining access to the HIV care continuum. Human rights for transgender women are central to HIV prevention and care in Jamaica.

## TUPDD0101

### Poor retention and care-related sex disparities among youth living with HIV in rural Mozambique

A Ahonkhai^1,2^; M Aliyu^1,3^; C Audet^1,3^; M Simmons^1^; G Claquin ^4^; S Vermund^1,2,5^ and W Wester^1,2^

^1^Vanderbilt University Medical Center, Vanderbilt Institute for Global Health, Nashville, USA. ^2^Vanderbilt University Medical Center, Division of Infectious Disease, Nashville, USA. ^3^Department of Health Policy, Vanderbilt University School of Medicine, Nashville, USA. ^4^Friends in Global Health, Maputo and Quelimane, Mozambique. ^5^Vanderbilt University Medical Center, Division of Pediatrics, Nashville, USA

Presenting author email: gael.claquin@fgh.org.mz

**Background**: Despite a 30% decline in HIV-related deaths over the past decade, HIV remains still the leading cause of death among African adolescents. Our objective was to summarize performance along the continuum of HIV care and identify predictors of loss to follow-up (LTFU) among youth enrolled in HIV care in rural Mozambique.

**Methods**: We analysed a retrospective cohort of youth (15–24 years) accessing care at one of 89 PEPFAR-supported clinics in Mozambique between 2012 and 2015. We determined the proportion of patients pre-antiretroviral therapy (ART) LTFU at 6 months, cumulative incidence of post-ART LTFU and mortality one-year post-initiation. We defined LTFU as being >60 days late for the last scheduled visit (pre-ART), or ART pick-up (post-ART). We used logistic and multivariable Cox regression models to identify predictors of pre- and post-ART LTFU, respectively, in the year after enrolment.

**Results**: Of 23,322 patients in the cohort, 19,287 (83%) were female. Primary referral source was prevention of mother-to-child transmission clinics for females (49%, *n* = 8639), and voluntary counselling and testing sites for males (65%, *n* = 2314). Females enrolled in care at earlier HIV disease stage (median CD4 469 vs. 363 cells/mm^3^, *p* < 0.001) and initiated ART more expeditiously than males (median 6 [IQR 0–129] vs. 35 [IQR 2–180] days, *p* < 0.001). Pre-ART LTFU at 1 year was 36% overall, and lower for females vs. males (33% vs. 56%, OR = 0.28; 95%CI:0.24–0.33). The cumulative incidence of post-ART LTFU was 36% overall (95%CI:35–36%), with small differences by sex (F:M 35% vs. 37%, aHR 0.66 95%CI:0.58–0.75). Adjusted one-year mortality rate for ART patients retained in care was 12.6% (95%CI:6–22%).

**Conclusions**: In the cohort of youth enrolled in care four-fifths were female. Females were enrolled in care earlier in their disease course, initiated on ART more quickly, and less likely to experience pre-ART LTFU than young males. After ART initiation, one-third of all patients were LTFU, and mortality rates were high. While outcomes were poor overall in this setting, young women may require enhanced prevention efforts, while young men need targeted testing and treatment interventions.

## TUPDD0102

### Gender age considerations and likelihood of viral load suppression at sub-national level in five counties, Kenya

S Muga ^1^; J Onyalo^1^; M Obuya^1^; P Njoka^1^; R Kithuka^1^; C Komen^1^; L Muyumbu^1^; G Obanyi^1^; E Ashiono^1^; C Muturi^1^; R Odhiambo^1^ and P Mwarogo^2^

^1^FHI360, USAID-APHIAPlus Nuru ya Bonde, Nakuru, Kenya. ^2^FHI360, Country Office, Nairobi, Kenya

Presenting author email: smuga@fhi360.org

**Background**: Sub-Saharan Africa accounts for 66% of new HIV infections globally. Program responses need to focus on sub-national contexts if epidemic control is to be achieved. The objective of this paper is to describe the likelihood of viral suppression at sub-national level, considering age and gender in five counties in Kenya.

**Methods**: This retrospective correlational survey reviewed the significance of age and gender on the likelihood of viral load suppression among clients on antiretroviral therapy (ART) who had accessed at least one viral load test in the period October 2015 to September 2016. ART was initiated as per the national guidelines in Kenya. Raw viral load data was assessed from the national viral load database for 139 facilities in five counties (Baringo, Kajiado, Laikipia, Nakuru and Narok) and 38,113 records were analysed. The survey used logistic regression to assess relationships between gender, age and viral load suppression (<1000 copies/ml), with *p* < 0.05.

**Results**: Overall, females were 16% more like to be virally suppressed compared to males. Females aged 1–9 years,10–14 years, 20–24 years were (62%, *p* = 0.00), (26%, *p* = 0.024), (79%, *p* = 0.001) respectively more likely to be virally suppressed than males. There was no significant difference in the likelihood of viral suppression between females and males for clients under one-year-old, (*p* = 0.230), 15–19 years (*p* = 0.254), and in 25 years or older (*p* = 0.079).

In Laikipia, males aged 10–14 years were 25% less likely to be virally suppressed. In Nakuru and Narok, males aged between one and nine years were 53% less likely to be virally suppressed. Further, in Nakuru those aged 20–24 years were 48% less likely to virally suppressed compared to female counterparts.

In Kajiado, Baringo and Laikipia counties, the likelihood of clients being virally suppressed increased with age. While in Narok and Nakuru counties, it was not dependent on increase in age.

**Conclusions**: The likelihood of achieving viral suppression seemed to agree with literature that female clients are likely to be suppressed. However, there are sub-national differences in the suppression rates. There are also differences in suppression rates through age groups in the counties. This information can be used for further research and effective programming.

## TUPDD0103

### An exploratory study to determine the survival period to switching for adult ART patients (15+ years) in Swaziland using routinely collected data

NH Nxumalo^1^; T Motsa^2^; F Shabalala^3^; M Shongwe ^3^; A Wagner^4^; M Malik^5^; K Matshotyana^1^ and K Payne^5^

^1^Management Sciences for Health, Mbabane, Swaziland. ^2^Strategic Information Department, Ministry of Health, Mbabane, Swaziland. ^3^University of Swaziland, Faculty of Health Sciences, Mbabane, Swaziland. ^4^Harvard Medical School, Harvard Pilgrim Healthcare Institute, Boston, USA. ^5^Management Sciences for Health, Arlington, USA

**Background**: It has been observed over the past five years that there is limited use of data stored in electronic data systems for making targeted programmatic decisions and conducting operational research. To demonstrate how routine data stored in these electronic systems can be used to inform HIV programming, SIAPS worked with the Ministry of Health (MoH) to conduct a study to determine the survival period to switching for adult patients (15+ years) on antiretroviral therapy (ART).

**Methods**: This was a retrospective cohort design study. The study population consisted of 117,586 adult (≥15 years) ART patient records identified as active between years 2010 to 2015 in the national ART database. The survival rates from time of ART initiation to time of regimen switch were evaluated according to gender and age using Kaplan–Meier models, that is, outcome variable was switching (binary event) and explanatory variables were WHO staging I; II; III; IV, Age categories: 15–24; 25–34; 35–44; and 45 years and above, and Sex: Male; Female.

**Results**: 3.6% (*n* = 4266) of the studied ART patients were found to have switched at some point during the course of treatment. Median survival time for all ART patients switching from first- to second-line regimen was found to be 607 days (95%CI: 601–613) days or 19 months. Patients aged 45 years and older at ART initiation remained on the first-line regimen for longer periods than other age groups at 93.5%. Survival times for males were less than those of females. Only 83% of patients initiated at WHO stage IV remained on first-line regimen by end of a five-year follow-up period.

**Conclusions**: There were significant differences in survival periods with men exhibiting a poorer survival period. Also, for the 18–25-year age group, we found that women actively switch regimen more often than men. Further studies would be required to understand the factors contributing to these findings. This can inform policies in HIV programming that target the unique needs of males and female clients.

## TUPDD0104

### Tanzanian men more successful than women in referring sexual partners to HIV testing via partner notification

K Curran ^1^; M Plotkin^2^; C Kahabuka^3^; A Christensen^3^; R Kisendi^4^; W Maokola^4^; M Betron^5^; K Grabbe^5^; M Drake^3^; E Mlanga^6^ and V Wong^7^

^1^Jhpiego, HIV and Infectious Diseases, Washington, USA. ^2^Jhpiego, Chapel Hill, USA. ^3^Jhpiego, Dar es Salaam, Tanzania, United Republic of. ^4^National AIDS Control Programme, Ministry of Health, Dar es Salaam, Tanzania, United Republic of. ^5^Jhpiego, Washington, USA. ^6^USAID, Dar es Salaam, Tanzania, United Republic of. ^7^USAID, Washington, USA

Presenting author email: kelly.curran@jhpiego.org

**Background**: Partner notification (PN) is effective at increasing uptake of HIV testing services (HTS) and identifying previously undiagnosed individuals. 2016 Guidance from WHO recommends inclusion of PN into HTS programmes. PN and referral to HTS can be conducted by the index client (passive notification) or with provider-assisted strategies. Passive PN involves HIV status disclosure and is impacted by socio-cultural dynamics surrounding sex and gender. This abstract describes the success of male versus female index clients in listing and referring sexual partners for testing in Tanzania.

**Methods**: A cross-sectional observational study was conducted in three hospitals in Njombe region, Tanzania, from June to September 2015. Individuals newly diagnosed with HIV in VCT or PITC were offered their choice of passive or provider-assisted referral for partner HIV testing. Odds ratios were calculated for likelihood of male and female index clients to successfully refer partner(s) to HTS or list multiple partners, and in-depth interviews were conducted with 40 index clients and partners.

**Results**: Almost all (91.6%) of the 390 (183 males and 207 females) index clients chose passive rather than provider-assisted referral. Of the 439 listed partners, 249 (56.7%) were successfully referred to HTS (63.4% of female partners; 49.8% of male partners). Male index clients were 2.2 (1.4–3.5) (*p* < 0.001) times more likely than female index clients to successfully refer at least one partner, and 6.2 (2.7–14.1) (*p* < 0.001) times more likely to list more than one partner. In qualitative analysis, both gender-specific (feeling undervalued if the relationship had not produced children; lacking contact info for commercial sex partners) and non-gender-specific (difficulty communicating with past partners) challenges were described.

**Conclusions**: PN has been shown to be effective and is being scaled up in multiple African countries, but little discussion has addressed gender and PN. In this study, men were more likely to list multiple partners and to successfully refer at least one partner to HTS. This strength could be built upon in programmatic approaches which target men for PNS. Women’s more limited ability to successfully refer their partner(s) could signal a need for different approaches to support women in the PN process.

## TUPDD0105

### Male engagement strategies effective in improving Option B+ retention in rural Mozambique

C Audet ^1^; E Graves^2^; M Bravo^3^; MFS Alvim^3^; A Green^2^ and YM Chire^4^

^1^Vanderbilt University, Health Policy, Nashville, USA. ^2^Vanderbilt University Medical Center, Nashville, USA. ^3^Friends in Global Health, Maputo, Mozambique. ^4^Friends in Global Health, Quelimane, Mozambique

Presenting author email: carolyn.m.audet@vanderbilt.edu

**Background**: Retention in antiretroviral therapy services initiated during antenatal care (ANC) in Mozambique is less than 30% at one year. To nurture supportive prevention of mother-to-child transmission (PMTCT) services we introduced a Male Engagement Strategy (MES) that involved partnering with Traditional Birth Attendants and training a new cadre of male-to-male community health agents, “Male Champions.” Together they counselled expectant couples to change community norms around male engagement in spousal/partner pregnancies and uptake of HIV services.

**Methods**: We conducted a retrospective analysis of women (>15 years) enrolled in HIV care and treatment through PMTCT services at 112 sites in rural Zambézia province, Mozambique. We compared clinical retention rates among sites receiving MES versus. those receiving standard of care (SOC) using chi-squared tests, Wilcoxon rank sum tests, and Cox regression models. In addition, we assessed the effect of MES on retention by implementation time using a Cox regression model.

**Results**: Six thousand five hundred women were enrolled in PMTCT care at MoH-run clinics receiving Friends in Global Health technical support from January 2015 to November 2016. Median age was 24 years (IQR: 20–29), 84% were married or living with a partner, median CD4 cell count was 463 cells/mm^3^, and 51% were enrolled in sites supported by MES. Cumulative incidence of ART loss to follow-up (LTFU) at six months was 38.1% (36.4%, 39.8%) among those enrolled at MES sites vs. 43.3% among those who received SOC (*p* = 0.001). Controlling for clinical (e.g. CD4 cell count) and social (e.g. education, marital status) characteristics, those who attended MES clinics had a 33% lower risk of being LTFU at six months versus those receiving SOC (*p* < 0.001). Longer duration of MES exposure at a clinic was associated with increased retention: covariate-adjusted hazard ratios for late ART pick-up decreased from 0.75 (0.65, 0.86) at 12 months to 0.47 (0.38, 0.58) at 36 months.

**Conclusions**: Programmes designed to encourage PMTCT services should include community and clinic-based interventions targeting male involvement in ANC and HIV services to improve maternal retention. Successful programs should see continuous improvement in clinical outcomes as activities become more socially acceptable and better integrated into clinical services.

## TUPDD0106

### Using traditional techniques to increase uptake of male circumcision and HIV testing and counselling services of males ages 15–29 through Lihawu Male Mentoring Camp

R Britch; T Churchyard and M Sibanda

Kwakha Indvodza, Mbabane, Swaziland

Presenting author email: britch.ryan@gmail.com

**Background**: Swaziland’s HIV prevalence remains the world’s highest at 26%. However, Swazi men often report fear and suspicion towards VMMC, mostly caused by solely biomedical approaches. In response, KI’s innovative project, Lihawu Male Mentoring Camp (LMMC), offers 15–29-year-old men a comprehensive package of adolescent male mentoring and health services, sensitization and interventions, with the following objectives:

- To increase number of circumcisions in the pivot age.
- To create a conducive space for VMMC clients to engage with mentors and peers in a fun way, clarifying myths and misconceptions about male health issues, masculinity and MC.
- To increase adherence to post-operative MC complementary care, conduct and lifestyle choices.

**Methods**: LMMC is a three-day residential camp of activities aimed at age pivot adolescents, combining behaviour change tenants of traditional Bantu initiation rites of passage with clinical best practice in VMMC.

Activities include challenges, games and cultural observances as well as sensitization on masculinity and gender awareness, goal setting, HIV and male health issues and services.

At the end of the camp, participants are offered a comprehensive package of male health services including HTC and VMMC.

**Results**: Increased VMMC (86%) and HTC (87%) uptake amongst pivot-age clients (national av. 19%) exposing them to a package of interventions and life skills marking a transition from childhood to adulthood. Pre- and post-camp surveys show a dramatic increase in gender equitable beliefs and acceptance of gender deviance, as well as increases in post-circumcision care and conduct, and in condom usage and efficacy knowledge.

**Abstract TUPDD0106–Table 1.** Increases of VMMC and HTC.

Health service	HIV status knowledge on entry	HIV status knowledge on exit
**HTC**	93/352 (26%)	307/352 (87%)
	No. of clinically eligible clients	No. of Circumcisions performed
**VMMC**	318/352	275/318 (86%)

**Abstract TUPDD0106–Figure 1.**
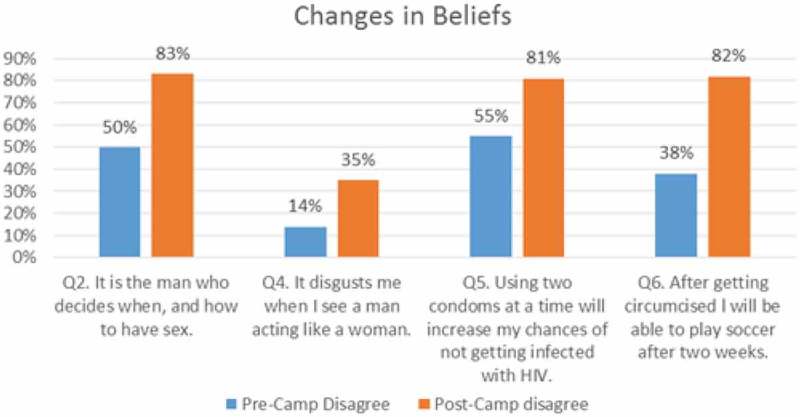
Changes in beliefs.

**Conclusions**: Contrary to common beliefs, traditional approaches may be utilized as effective methods to sensitize men on positive masculinity, SRH issues, and the benefits of VMMC, as well as dramatically increase uptake in prevention services.

## WEPDB0101

### Immediate vs. delayed oral etoposide (ET) among HIV-infected individuals with limited-stage KS initiating ART: A5264/AMC-067 study

MC Hosseinipour ^1,2^; M Kang^3^; SE Krown^4^; A Bukuru^5^; T Umbleja^3^; J Martin^6^; J Orem^7^; C Godfrey^8^; B Hoagland^9^; N Mwelase^10^; D Langat^11^; M Nyirenda^12^; J MacRae^13^; M Borok-Williams^14^; W Samaneka^14^; A Moses^1,2^; O Martinez-Maza^15^; R Ambinder^16^; D Dittmer^2^; M Nokta^17^; T Campbell^18^; A5264/AMC-067 REACT-KS team

^1^UNC Project, Lilongwe, Malawi. ^2^University of North Carolina School of Medicine, Chapel Hill, USA. ^3^SDAC/Harvard School of Public Health, Center for Biostatistics in AIDS Research, Boston, USA. ^4^AIDS Malignancy Consortium, New York, USA. ^5^Joint Clinical Research Center (JCRC), Kampala, Uganda. ^6^University of California, Center for AIDS Prevention Studies, San Francisco, USA. ^7^Uganda Cancer Institute, Kampala, Uganda. ^8^HIV Research Branch, TRP, DAIDS, NIAID, NIH, Rockville, USA. ^9^INI Evandro Chagas/FIOCRUZ, Rio de Janiero, Brazil. ^10^University of Witwatersrand, Johannesburg, South Africa. ^11^Kenya Medical Research Institute/Walter Reed Project, Nairobi, Kenya. ^12^Johns Hopkins Project, University of Malawi College of Medicine, Blantyre, Malawi. ^13^IMPACTA Peru, Lima, Peru. ^14^University of Zimbabwe, Harare, Zimbabwe. ^15^University of California, Los Angeles, USA. ^16^Johns Hopkins University, Baltimore, USA. ^17^National Cancer Institute, Bethesda, USA. ^18^University of Colorado Denver, Aurora, USA

Presenting author email: minach@med.unc.edu

**Background**: Limited-stage KS often responds to ART alone; the role for adjuvant chemotherapy is unclear. We assessed the impact of immediate vs. delayed oral etoposide (ET) among HIV-infected individuals with limited-stage KS initiating ART.

**Methods**: ART-naïve, HIV-1-infected adults with limited-stage KS (stage T0 and T1 [minimal oral KS and/or asymptomatic edema]) were randomized to ART (TDF/FTC/EFV) alone (Arm A) versus ART plus up to eight cycles of oral ET (Arm B) and followed for 96 weeks. Participants with KS progression on ART alone received ET as part of Arm A strategy. Participants who received non-study chemotherapy after ET continued follow-up. Primary outcome was categorical (van Elteren test stratified by CD4): Failure (composite of KS progression, initiation of non-study chemotherapy, lost-to-follow-up, death), Stable and Response (partial or complete) at 48 weeks compared to baseline. Sensitivity analysis excluded receipt of non-study chemotherapy in Failure. Secondary outcomes included times to initial KS progression, suspected KS-IRIS and KS response (Gray’s tests).

**Results**: Study terminated early for futility after DSMB interim review. Of 190 participants (A = 94, B = 96); male: 71%; African: 93%; mean age: 35 years; T1:61%. Failure (53.8% vs. 56.6%), Stable (16.3% vs. 10.8%) and Response (30% vs 32.5%) did not differ between arms (A vs. B) among those with Week 48 data potential (*N* = 163, *p* = 0.91). Failure (48.8% vs. 38.6%), Stable (16.3% vs. 16.9%) and Response (35.0% vs. 44.6%) were also not different in sensitivity analysis (*p* = 0.17). Times to KS progression (*p* = 0.021), KS-IRIS (*p* = 0.003) and KS response (*p* = 0.003) favoured Arm B (Figure 1). Mortality and adverse events were similar.

**Abstract WEPDB0101–Figure 1.**
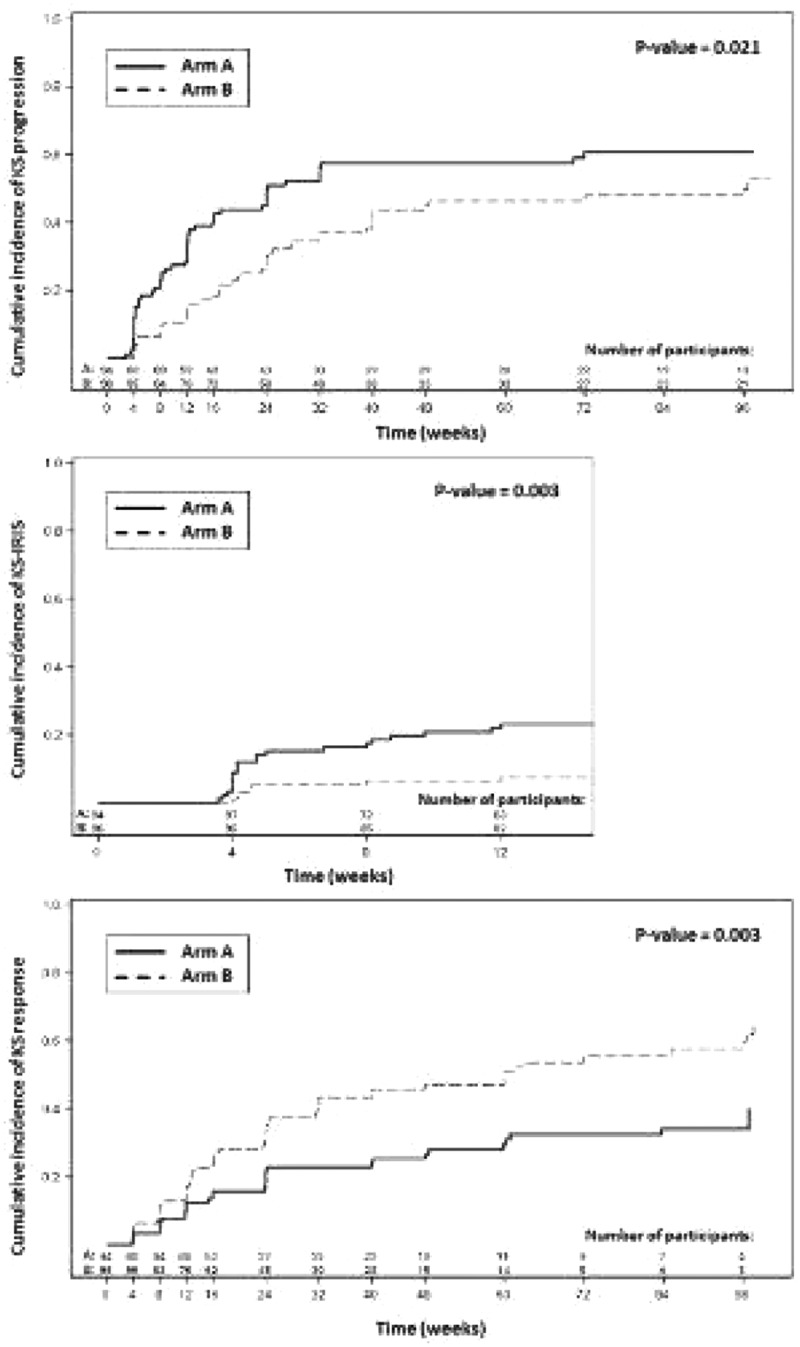
Time to event analysis

**Conclusions**: Immediate ET showed no benefit compared to delayed ET by the primary endpoint. Pre-specified secondary analyses showed shortened time to KS response, reduced KS-IRIS incidence and increased time to KS progression with immediate ET, but no effect on mortality or need for additional, non-ET chemotherapy.

## WEPDB0102

### Implementing CRAG screening in HIV patients initiating ART in rural HIV clinics with regular absence of CD4 testing services in rural Tanzania

G Mbwanji; Diana Faini; A Nyuri; Andrew Katende; Aneth Kalinjuma; Maja Weisser; David Boulware and Emili Letang

Ifakara Health Institute, HIV/AIDS, Morogoro, Tanzania, United Republic of

Presenting author email: gmbwanji@ihi.or.tz

**Background**: The World Health Organization (WHO) recommends screening for cryptococcal antigen (CRAG) in blood of HIV-infected antiretroviral therapy (ART)-naïve patients with CD4 <100 cells/µL. CRAG+ persons who receive ART but not antifungal therapy are at a high risk of death. However, absence of reliable or prompt CD4 testing services in rural clinics jeopardizes implementation of CRAG screening.

**Methods**: We implemented CRAG screening in all primary health HIV clinics in the Kilombero district, southern Tanzania. Point-of-care CRAG lateral flow assay testing was recommended for all ART-naïve HIV-infected persons with criteria for ART initiation or with headache for >5 days. All CRAG+ persons were transported to the Referral Hospital in Ifakara for a meningitis diagnostic workup and antifungal therapy. Patient transport costs, antifungals, and incentives to clinicians were provided.

**Results**: From November 2015 to November 2016, 723 ART-naïve patients were tested for CRAG in 8 HIV clinics. Of these, 45 (6.2%) were CRAG+, and 26 (58%) were diagnosed at peripheral clinics and referred to Referral Hospital for evaluation. The median age of the CRAG+ patients was 35 years (interquartile range [IQR], 21–55), and 60% (27/45) were women. Lumbar punctures were performed in 41 consenting (91%) patients, and 51% (21/41) of patients were CRAG+ in cerebrospinal fluid (CSF). Among these 21 CSF CRAG+ persons, 3 were asymptomatic (7% of overall CRAG+ persons).

**Conclusions**: Our CRAG screening algorithm tailored for rural HIV clinics was effective in maximizing cryptococcal detection in advanced HIV patients at a district level in the absence of regular CD4 testing. The high CRAG prevalence found highlights the importance in the absence of CD4 testing of extending CRAG screening to all HIV-infected persons enrolling in care in order to reduce early mortality.

## WEPDB0103

### High mortality despite high-dose oral fluconazole (1600 mg) and flucytosine, and serial lumbar punctures, for HIV-associated cryptococcal meningitis: ANRS 12257 study in Burundi and Ivory Coast

A Chabrol ^1^; A Doumbia^2^; R Landman^3,4,5^; A Fontanet^6^; SP Eholie^7^; T Niyongabo^8^; L Nizigama^8^; D Laureillard^9^; B Sylla^4^; H Menan^10^; C Padoin^11^; S Brun^12^; C Alloui^13^; S Gibowski^14^; A Kakou^2^; O Bouchaud^15^; ANRS 12257 Study Group

^1^Centre Hospitalier Sud Francilien, Infectious Diseases, Corbeil Essonnes, France. ^2^CHU Treichville, Infectious Diseases, Abidjan, Cote D’Ivoire. ^3^Hôpital Bichat, Infectious Diseases, Paris, France. ^4^Institut de Médecine et d’Epidémiologie Appliquée, Paris, France. ^5^INSERM, IAME, UMR 1137, Paris, France. ^6^Institut Pasteur, Emerging Diseases Epidemiology Unit, Paris, France. ^7^CHU Treichville, Infectious Diseases, Abidjan, France. ^8^CHU Kamenge, Infectious Diseases, Bujumbura, Burundi. ^9^CHU Caremeau, Infectious Diseases, Nîmes, France. ^10^CHU Treichville, Parasitology and Mycology, Abidjan, Cote D’Ivoire. ^11^CHU Avicenne, Pharmacology, Bobigny, France. ^12^CHU Avicenne, Parasitology and Mycology, Bobigny, France. ^13^CHU Avicenne, Virology, Bobigny, France. ^14^ANRS, Clinical Research Safety Office, Paris, France. ^15^CHU Avicenne, Infectious Diseases, Bobigny, France

Presenting author email: amelie.chabrol@chsf.fr

**Background**: Mortality from HIV-associated cryptococcal meningitis (CM) remains unacceptably high in low-income countries, where applicable and effective antifungal strategies are needed. In a context where Amphotericine B (AmB) is unavailable, in hospitals lacking intensive care units, we evaluated prospectively the safety and efficacy of an oral combination of fluconazole 1600 mg and flucytosine associated with serial lumbar punctures (SLP) in HIV- associated CM.

**Methods**: Eligible HIV-infected patients presenting a first episode of CM were enrolled in a one-arm open-label clinical trial in Burundi and Ivory Coast from 2012 to 2015. After inclusion, patients received fluconazole 1600 mg per day in combination with flucytosine 100 mg/kg per day for 2 weeks, followed by fluconazole alone, 800 mg per day for 8 weeks and then 200 mg until the end of follow-up (24 weeks). Intracranial pressure was treated with SLP, according to IDSA recommendations. The primary endpoint was 10-week mortality, expected at 35% ±15% precision. Secondary endpoints were 2-week and 24-week mortality, early fungicidal activity (EFA) determined by serial quantitative cerebrospinal fluid (CSF) cultures, and safety.

**Results**: Forty-one (22F/19M) patients were included, 59% being antiretroviral therapy (ART)-naive; 14 (34%) had reduced level of consciousness and 24 (59%) had elevated intracranial pressure at presentation. Overall 10-week mortality was 48.8% (95% CI = 32.9–64.9); 2-week and 24-week mortality were 26.8% (14.2–42.9) and 58.5% (42.1–73.7), respectively. Mean EFA was −0.27 +/- 0.20 log CFU/ml per day, and 16 patients had sterile CSF after two weeks of treatment. The treatment appeared to be well tolerated with no study drug discontinuation. For naive patients, ART was started at a median of 28 days, with no cryptococcal immune reconstitution inflammatory syndrome observed.

**Conclusions**: Mortality with high dose oral fluconazole and flucytosine associated with SLP was at the upper range of expected values. Oral treatment seems to be less effective than AmB-based combinations, probably due to lower fungicidal activity. Nevertheless, in low-income countries where AmB is not available, this combination appears to be a well-tolerated therapeutic option.

## WEPDB0104

### Comparison of various anal intraepithelial neoplasia screening strategies including standard anoscopy, anal cytology and HPV genotyping in HIV-positive men who have sex with men

P Boucheron^1^; S Pernot^2^; H Peré^3^; ML Lucas^4^; D Veyer^3^; N Fatallah^5^; V De Parades^5^; J Pavie^4^; J Netter^2^; L Collas^4^; J Taieb^2^; S Grabar^1^ and L Weiss ^4^

^1^Groupe hospitalier Hôtel Dieu (AP-HP), Paris Descartes Sorbonne Cité University, Biostatistics and Epidemiology Unit, France, France. ^2^Hôpital Européen Georges Pompidou, Paris Descartes Sorbonne Cité University, Hépato-gastroentérologie et oncologie digestive, Paris, France. ^3^Hôpital Européen Georges Pompidou, Paris Descartes Sorbonne Cité University, Biology, Paris, France. ^4^Hôpital Européen Georges Pompidou, Paris Descartes Sorbonne Cité University, Immunology, Paris, France. ^5^Groupe Hospitalier Saint-Joseph, Proctology, Paris, France

Presenting author email: laurence.weiss@aphp.fr

**Background**: There is no international consensus on anal cancer screening strategy. Guidelines range from digital anorectal examination (DARE) including standard anoscopy (SA) alone (France) to DARE combined with anal cytology (Pap) (IDSA) +/– HPV genotyping in HIV-positive men who have sex with men (HIV+ MSM), to detect high grade intraepithelial neoplasia (HGAIN). This study aimed at comparing various HGAIN screening strategies yields based on Pap, SA and HPV genotyping alone or in combination in HIV+ MSM.

**Methods**: Pap, SA and HPV genotyping were performed systematically on consecutive HIV+ MSM attending for the first time a cancer screening consultation between January 2012 and August 2016 in a French hospital. High-resolution anoscopy (HRA) was performed in case of HPV16 positivity or abnormal cytology (ASCUS, LSIL, HSIL). Targeted biopsies were performed when dysplasia was suspected. Screening yield was defined as the number of patients with HGAIN relative to the total number of patients screened. Each strategy was compared with the complete strategy.

**Results**: 212 patients (median age 51 (IQR:45–57), HIV-RNA <20 in 84% of patients, median CD4: 682/mm^3^ (IQR:491–890)) were screened. The most frequent HPV genotypes were high risk HPV: HPV52 (24.5%), HPV16 (18.9%), HPV53 (18.4%), HPV31 (15.6%) and HPV68 (15.6%). 86 out of 212 (40.6%) patients had at least one positive screening test (Pap+: 62/212(29.2%), HPV16+: 40/212 (18.9%), SA = dysplasia: 19/212 (9.0%)). Screening strategies yields to detect HGAIN compared with the complete strategy and Pap alone are presented here.

**Abstract WEPDB0104–Table 1.** 

Anal cancer screening strategy (*N* = 212)	Number of HRA performed	Number of biopsies performed	HGAIN at histology: *N*(%)	Strategy vs. complete strategy : P(Fisher)	Strategy vs. Pap alone : P(Fisher)
**SA**	0	19	7 (3.3%)	<0.001	0.02
**HPV16 genotyping**	39	26	14 (6.6%)	<0.05	0.47
**Pap**	59	40	19 (9.0%)	0.27	
**SA + HPV16 genotyping**	33	40	19 (9.0%)	0.27	1.00
**Pap + HPV16 genotyping**	75	48	23(10.8%)	0.65	0.63
**SA + Pap**	51	52	24 (11.3%)	0.77	0.52
**SA + Pap + HPV16 genotyping**	67	59	27 (12.7%)		0.27

**Conclusions**: Pap alone or combinations of two screening tests yielded to similar rates of HGAIN detection than the complete strategy. Compared to Pap alone, Pap + HPV16 slightly improved the number of HGAIN detected but not significantly. Given the limited number of clinicians trained in HRA and the perspective of self-sampling, Pap +/– HPV16 screening might be the best strategy to increase screening acceptance and to identify HGAIN in HIV+ MSM.

## WEPDB0105

### Human Papillomavirus infection and cervical lesions in HIV-1-infected women on antiretroviral treatment in Thailand

T Delory^1,2,3^; N Ngo-Giang-Huong^1,2,4^; S Rangdaeng^5^; N Chotivanich^6^; A Limtrakul^7^; C Putiyanun^8^; P Suriyachai^9^; W Matanasarawut^10^; T Jarupanich^11^; P Liampongsabuddhi^12^; I Heard^13^; G Jourdain^1,2,4^; M Lallemant^1,2,4^; S Le Cœur ^1,2,14^; PapilloV study group

^1^Institut de Recherche pour le Developpement (IRD), UMI 174-PHPT, Chiang Mai, Thailand. ^2^Faculty of Associated Medical Sciences, Chiang Mai University, Chiang Mai, Thailand. ^3^AP-HP, Service de Maladies Infectieuses et Tropicales, Paris, France. ^4^Harvard T H Chan School of Public Health, Boston, USA. ^5^Department of Pathology, Chiang Mai University, Faculty of Medicine, Chiang Mai, Thailand. ^6^Chonburi Hospital, Ministry of Public Health, Chonburi, Thailand. ^7^Nakornping Hospital, Ministry of Public Health, Chiang Mai, Thailand. ^8^Chiang Kham Hospital, Ministry of Public Health, Chiang Kham, Thailand. ^9^Phayao Hospital, Ministry of Public Health, Phayao, Thailand. ^10^Lamphun Hospital, Ministry of Public Health, Lamphun, Thailand. ^11^Hat Yai Hospital, Ministry of Public Health, Hat Yai, Thailand. ^12^Lampang Hospital, Ministry of Public Health, Lampang, Thailand. ^13^HPV National Reference Center, Pasteur Institute, Paris, France. ^14^Institut National d’Etudes Demographiques (INED), Mortality Health and Epidemiology Unit, Paris, France

**Background**: Rates of Human Papillomavirus (HPV) infections and cervical lesions are both increased in HIV-infected women. The objectives of the study were to estimate the prevalence and factors associated with Human Papillomavirus (HPV) infection, HPV genotypes and cytological/histological high-grade (HSIL+/CIN2+) lesions in HIV-1 infected women receiving combination antiretroviral therapy (cART).

**Methods**: We conducted a cross-sectional study (PapilloV study, NCT01792973) within a prospective cohort (the PHPT cohort) of HIV-infected women on cART in 24 hospitals accross Thailand. Cervical specimens were collected for cytology and HPV genotyping (Papillocheck®). Any women with High-Risk-HPV (HR-HPV), and/or potentially HR-HPV (pHR-HPV) and/or ASC-US or higher (ASC-US+) lesions were referred for colposcopy. Factors associated with HR-HPV infection and with HSIL+/CIN2+ lesions were investigated using mixed effects logistic regression models.

**Results**: 829 women were enrolled: median age 40.4 years, on cART (613 on NNRTI-based regimen and 213 on PI-based regimen) for a median of 6.9 years, median CD4 cell-count 536 cells/mm3, and 788 (96%) with HIV-viral load <50 copies/mL, 196 (24%) were CDC-stage C and 125 (18%) had history of virologic failure. Of 214 (26%) infected with HPV: 159 (19%) had ≥1 HR-HPV, of whom 38 (5%) HPV52, 22 (3%) HPV16, 9 (1%) HPV18; 21 (3%) had pHR-HPV, 34 (4%) low-risk HPV infection, and 56 (26%) had multiple genotypes. Younger age, low CD4 cell-counts and low education were independently associated with HR-HPV infection. 72 women (9%) had ASCUS+ and 28 (3%) HSIL+/CIN2+ lesions. HR-HPV infection was independently associated with HSIL+/CIN2+ lesions.

**Conclusions**: The prevalence of HPV infection and of cervical lesions was low. CD4 cell count was inversely associated with the presence of HR-HPV infection, indicating the need for closer gynecological follow-up in case of immunological failure.

## WEPDB0106

### Screening for tuberculosis with Xpert MTB/RIF versus fluorescent microscopy among people newly diagnosed with HIV in rural Malawi: a cluster-randomized trial

LG Ngwira ^1^; M Khundi^2^; GL Barnes^3^; A Nkhoma^2^; M Murowa^4^; S Cohn^3^; L Moulton^3,5^; RE Chaisson^5,6,7^; EL Corbett^8,9^; DW Dowdy^5,6,7^; CHEPETSA Study Group

^1^Malawi Liverpool Wellcome Trust, Clinical Research Department, Blantyre, Malawi. ^2^Malawi Liverpool Wellcome Trust, Clinical Research, Blantyre, Malawi. ^3^Johns Hopkins School of Medicine, Center for TB Research, Baltimore, USA. ^4^Malawi Ministry of Health, Thyolo, Malawi. ^5^Department of International Health, Johns Hopkins Bloomberg School of Public Health, Baltimore, USA. ^6^Center for TB Research, Johns Hopkins School of Medicine, Baltimore, USA. ^7^Department of Epidemiology, Johns Hopkins Bloomberg School of Public Health, Baltimore, USA. ^8^Malawi Liverpool Wellcome Trust, Clinical Research Programme, Blantyre, Malawi. ^9^Clinical Research Department, London School of Hygiene and Tropical Medicine, London, UK

**Background**: Tuberculosis (TB) is the leading cause of HIV-associated death. Xpert MTB/RIF (Xpert) has greater diagnostic sensitivity than microscopy but also higher costs and infrastructural requirements. Sensitive TB screening following HIV diagnosis could potentially reduce mortality.

**Methods**: Cluster-randomized trial of point-of-care TB screening using Xpert versus LED fluorescent smear microscopy (LED FM) across 12 primary care clinics in Thyolo District, Malawi (ClinicalTrials.gov NCT01450085). Randomization was constrained (1:1), with a primary outcome of 12-month all-cause mortality. Participants newly diagnosed with HIV underwent TB symptom screening followed by Xpert or LED FM if symptomatic and isoniazid preventive therapy if asymptomatic. Analyses used *t*-tests of log-transformed cluster-level rates, and Poisson regression.

**Results**: Of 1842 participants recruited (Figure 1), 24/1001 (2.4%) participants in the Xpert arm and 10/841 (1.2%) in the LED FM arm had TB diagnosed at entry. The primary outcome was similar across arms (overall mortality 6.45 per 100 person-years with Xpert vs. 7.80 with LED FM, rate ratio 0.83, 95% CI: 0.63–1.09; *p* = 0.14; see Figure 2). However, a pre-specified secondary analysis among people with WHO stage 3 or 4 disease (*n* = 463) showed significantly lower mortality in the Xpert arm (15.9 vs. 34.8 per 100 person-years, rate ratio 0.46, 95% CI: 0.22–0.93; *p* = 0.03). Unadjusted and adjusted results were similar.

**Conclusions**: Screening rural adult Malawians recently diagnosed with HIV for tuberculosis using point-of-care Xpert MTB/RIF increased baseline diagnoses of TB and halved mortality among individuals with stage 3 or 4 disease but did not significantly affect all-cause mortality overall.

**Abstract WEPDB0106–Figure 1.**
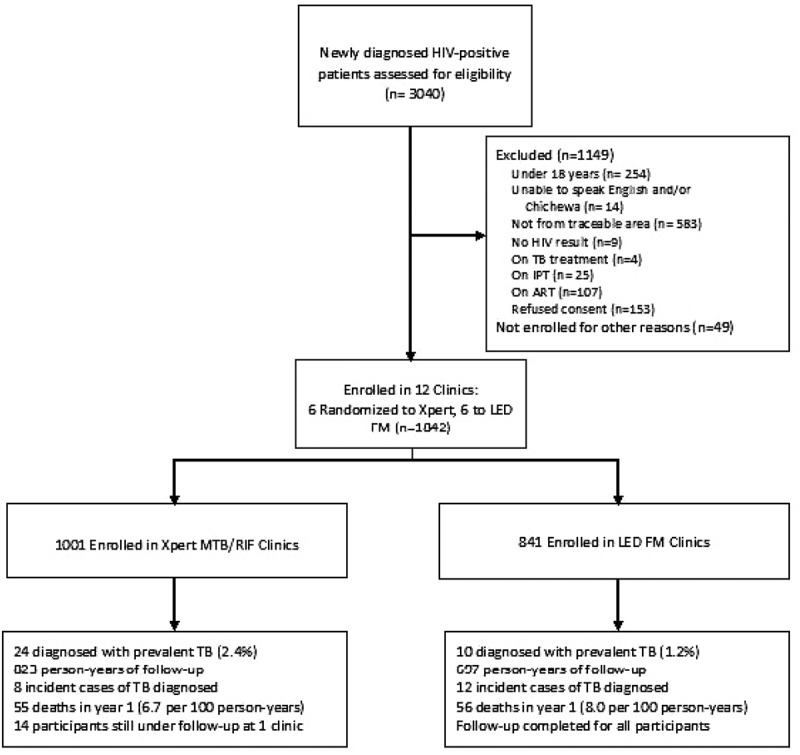
Study profile.

**Abstract WEPDB0106–Figure 2.**
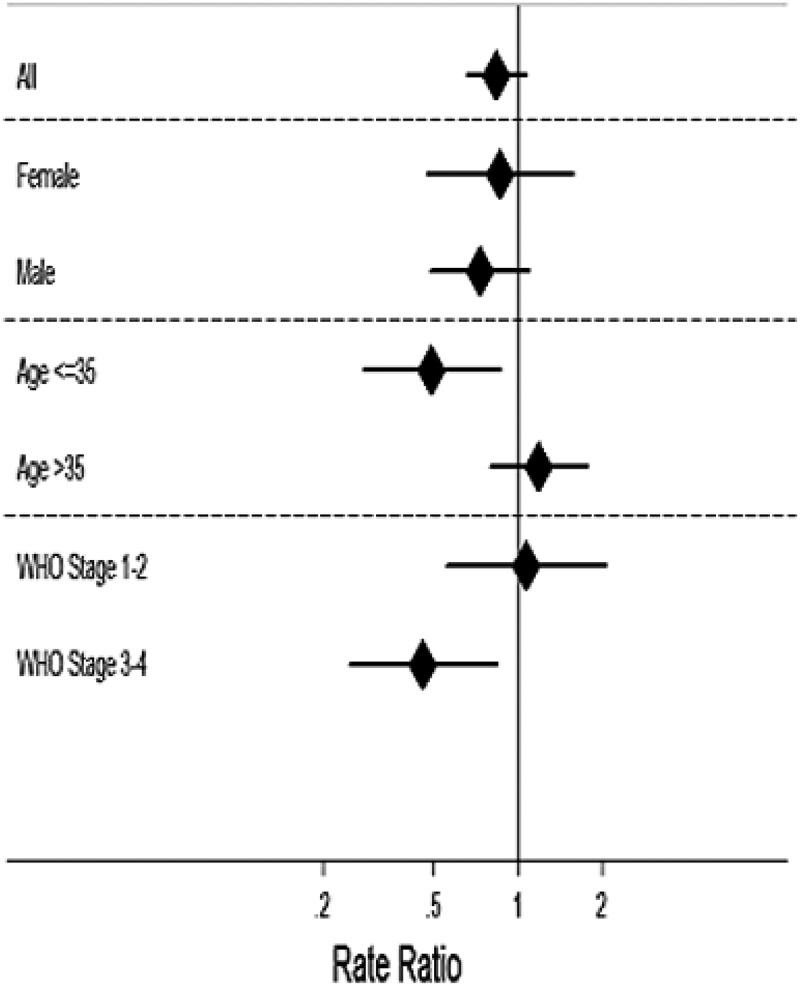
RR (Xpert v LED FM) for all-cause mortality.

## WEPDC0101

### STI co-infections at HIV diagnosis in France

F Lot; J Pillonel and F Cazein

Santé Publique France, Saint-Maurice, France

Presenting author email: florence.lot@santepubliquefrance.fr

**Background**: Sexual risk behaviours expose people to HIV infection but also to other sexually transmitted infections (STI). In the context of an increase of STI in France, the aim of our work was to analyse the frequency of STI in people newly diagnosed for HIV between 2012 and 2015.

**Methods**: Since 2012, mandatory HIV surveillance system in France has collected information on bacterial STI (syphilis, gonorrhea, chlamydia trachomatis infection or lymphogranuloma venereum-LGV). These infections had to be reported if they were concurrently diagnosed at the time of HIV diagnosis or diagnosed in the last 12 months before HIV diagnosis (STI/HIV co-infections).

**Results**: Information on STI infection was available for 9.207 HIV diagnoses in adults during the period 2012–2015 (52% of all diagnoses). STI/HIV co-infection was globally 14.2% (1.310/9.207), but was more frequent in men having sex with men (MSM) (25.4%) than in heterosexuals born in France (9.0%: 11.1% in men and 6.0% in women) or abroad (3.4%: 5.0% in men and 2.3% in women) and in injecting drug users (6.7%). STI/HIV co-infection was more frequent when HIV infection was diagnosed during acute illness.

STI/HIV co-infection has increased overtime (from 12.9% in 2012 to 16.9% in 2015), but this increase was observed only in MSM (from 22.0% to 30.0%).

Among STI, the frequency of syphilis, gonorrhea, chlamydia and LGV were respectively 74.3%, 15.8%, 15.0% and 1.7%. Chlamydia was the only STI more frequent in heterosexuals compared to MSM.

**Conclusions**: STI/HIV co-infections affect almost one third of MSM newly diagnosed with HIV in 2015, and most commonly syphilis/HIV. These results highlight the importance to combine HIV testing to other STI, and to offer an HIV test to patients presenting with a STI.

## WEPDC0102

### Acquisition of sexually transmitted infections among women using a variety of contraceptive options: a prospective study among high-risk African women

FK Matovu ^1,2^; E Brown^3^; A Mishra^4^; G Nair^5^; T Palanee-Phillips^6^; N Mgodi^7^; C Nakabiito^8^; N Chakhtoura^9^; S Hillier^10^ and J Baeten^4^

^1^Makerere University-Johns Hopkins University Research Collaboration, Kampala, Uganda. ^2^Makere University School of Public Health, Kampala, Uganda. ^3^SCHARP-FHCRC, Seattle, USA. ^4^University of Washington, Seattle, USA. ^5^Emavundleni Research Centre, Cape Town, South Africa. ^6^Wits RHI, Johannesburg, South Africa. ^7^UZ-UCSF, Harare, Zimbabwe. ^8^MUJHU Research Collaboration, Kampala, Uganda. ^9^NICHD/ NIH, Bethesda, Uganda. ^10^Magee-Womens Research Institute, Pittsburgh, USA

Presenting author email: fmatovu@mujhu.org

**Background**: In many African settings, women concurrently face high risk of HIV-1, STIs and unintended pregnancies. Few studies have evaluated STI risk among users of hormonal implants and intrauterine devices (IUDs) although these long-acting reversible methods are being promoted widely because of their contraceptive benefits. Within a prospective study of women at risk for HIV, we compared the risk of STI acquisition among women using different contraceptive methods.

**Methods**: MTN-020/ASPIRE was a randomized trial of the dapivirine vaginal ring for HIV-1 prevention that enrolled 2629 women from Malawi, South Africa, Uganda and Zimbabwe; all were required to use contraception at study entry. Analysis was restricted to 2264 women (50.2% from South Africa) who used DMPA (*n* = 1147), implants (*n* = 692), NET-EN (*n* = 438) or IUD (*n* = 541) at any point during follow-up. Screening and treatment for Chlamydia trachomatis, Neisseria gonorrhoeae and Trichomonas vaginalis occurred at baseline, semi-annually and when clinically indicated.

**Results**: Over 3440 person-years of follow-up, 408 cases of C.trachomatis (incidence 11.86/100 person-years), 196 of N.gonorrhoeae (5.70/100 person-years) and 213 cases of T.vaginalis (6.19/100 person-years) were detected. The incidence of C.trachomatis and N.gonorrhoeae were not significantly different across contraceptive methods (Table 1), although DMPA and implant users had lower incidence than IUD users. The incidence of T.vaginalis was significantly lower for DMPA, implant and NET-EN users, compared with IUD users. Findings were consistent across South Africa and non-South Africa sites.

**Abstract WEPDC0102–Table 1.** Incidence of STIs by contraceptive method.

**Conclusions**: Among African women at high risk for HIV-1, we found that risk of cervical infections (N.gonorrhoeae and C.trachomatis) did not differ across contraceptive methods. Significantly lower rates of T.vaginalis among progestin based methods compared to IUD users was seen, likely due to hypoestrogenic states which may not be conducive for persistence of T.vaginalis. Results are reassuring and lend support to current WHO guidance that women should have a wide range of contraceptive options.

## WEPDC0103

### Differences in biological and behavioral HIV risk before, during and after PrEP use among a national sample of gay and bisexual men in the United States

J Parsons ^1^; HJ Rendina^1^; T Whitfield^1^ and C Grov^2^

^1^Hunter College-CUNY, Psychology, New York, USA. ^2^CUNY School of Public Health, New York, USA

Presenting author email: jeffrey.parsons@hunter.cuny.edu

**Background**: Some have expressed concern that gay and bisexual men (GBM) who use pre-exposure prophylaxis (PrEP) will engage in more condomless anal sex (CAS) and acquire/transmit STIs more frequently while on PrEP. Others, however, argue that increases in STIs among men on PrEP result from required STI screening and testing. There has been little longitudinal data to support either conclusion.

**Methods**: Data were collected from *One Thousand Strong*, a longitudinal study of 1071 HIV-negative GBM from across the US. Participants were tested for urethral and rectal gonorrhea and chlamydia and asked to report on their PrEP use every 12 months.

**Results**: In cross-sectional between-group analyses, the 823 PrEP-naive men had a significantly lower STI infection rate (4.2%) than the 77 men currently (10.4%) or 17 men formerly (11.8%) on PrEP, X^2^(2) = 7.72, *p* = 0.02; men currently on PrEP also reported significantly more acts of CAS, H(2) = 37.73, *p* < 0.001. Within-person longitudinal analyses of the 181 men who reported PrEP use indicated slight but non-significant increases in the odds of an STI diagnosis while on PrEP (OR = 1.25, *p* = 0.55) and after discontinuing PrEP (OR = 1.43, *p* = 0.53) in comparison to pre-uptake of PrEP. We also saw no significant changes in CAS while on PrEP (OR = 1.09, *p* = 0.76) or after PrEP discontinuation (OR = 0.48, *p* = 0.10) compared to pre-uptake levels.

**Conclusions**: Our findings failed to support the notion that GBM experience an increase in CAS and STIs while on PrEP. Although PrEP-naive GBM have fewer STIs and report less CAS than current and former PrEP users, these data provide support for the notion that the highest risk GBM are the ones who initiate PrEP use, and their risk behaviours do not change substantially as a result. It is worth noting that most of the men on PrEP in this sample are early adopters, and further research is needed to determine whether behavioral differences may emerge in larger samples of men who may engaging in lower levels of HIV risk behaviour at the time of PrEP initiation.

## WEPDC0104

### Partner notification of sexually transmitted infections among MSM on PrEP: a sub-study of the ANRS-IPERGAY trial

M Suzan-Monti ^1,2,3^; L Cotte^4^; L Fressard^1,2,3^; E Cua^5^; C Capitant^6^; L Meyer^6^; J-M Molina^7^ and B Spire^1,2,3^

^1^INSERM UMR912 – SESSTIM, Marseille, France. ^2^Aix Marseille Université, UMR_S 912, IRD, Marseille, France. ^3^ORS PACA, Observatoire Régional de la Santé Provence-Alpes-Côte d’Azur, Marseille, France. ^4^Hôpital de la Croix Rousse, Centre Hospitalier et Universitaire de Lyon, Lyon, France. ^5^Hôpital de l’Archet, Centre Hospitalier de Nice, Département de Maladies Infectieuses, Nice, France. ^6^INSERM SC10, Villejuif, France. ^7^Hôpital Saint Louis AP-HP, Service de Maladies Infectieuses, Paris, France

Presenting author email: marie.suzan@inserm.fr

**Background**: Sexual partners of people with HIV or other sexually transmitted infections (STI) are at high risk of infection. Partner notification (PN) is a useful public health approach to enhance both targeted testing of those at very high risk, and linkage to care for undiagnosed HIV-positive /STI-positive individuals. Despite WHO recommendations, PN is implemented differently worldwide. In France, there are no specific guidelines, and information about PN practices is scarce. We used the ANRS-IPERGAY PrEP prevention trial to investigate PN in HIV-negative men who have sex with men (MSM), who reported STI.

**Methods**: The present sub-study was conducted among 275 participants who completed a specific online PN questionnaire, during the open-label extension study of the ANRS-IPERGAY trial, between April and June 2016. Socio-demographic data were collected at inclusion. Data about their most recent sexual encounter and about preventive behaviours were collected at the follow-up visit prior to filling out the PN questionnaire to define variables to be used as proxies of at-risk practices. Chi-2 or exact Fisher tests were used to select variables eligible for multiple logistic regression analysis.

**Results**: Among the 275 participants, 250 reported at least one previous STI. Among the latter, 172 (68.8%) had informed their partner(s) of their most recent STI. Of these, 138 (80.2%) had notified their occasional partners and 83 (48.3%) their main partner. There was no significant socio-demographic difference between MSM who notified their partner(s) and those who did not. Multiple logistic regression showed that MSM were less likely to notify their main partner when their most recent sexual encounter was through unprotected anal sex with an occasional partner (aOR[95% CI] 0.31[0.14;0.68], *p* = 0.03). Older MSM were less likely to inform occasional partners (aOR[95% CI] 0.44[0.21;0.94], *p* = 0.03), while those participating in chemsex at their most recent sexual encounter were more likely to inform their occasional partners (aOR[95% CI] 2.56[1.07;6.09] *p* = 0.03).

**Conclusions**: Unprotected sexual relationships with people other than main partners, and recreational drug use were identified, respectively, as a socio-behavioural barrier to and motivator of PN among a sample of high-risk MSM. These results provide a first insight into the process of PN and might fuel reflection about PN in France.

## WEPDC0105

### Predictors of genital ulcerations in HIV-serodiscordant couples, Lusaka, Zambia

K Wall ^1^; W Kilembe^2^; B Vwalika^2^; L Haddad^3^; S Lakhi^2^; R Chavuma^2^; K Naw Htee^3^; I Brill^4^; C Vwalika^2^; L Mwananyanda^2^; E Chomba^2^; J Mulenga^2^; A Tichacek^3^ and S Allen^3^

^1^Emory University, Epidemiology, Atlanta, USA. ^2^Rwanda Zambia HIV Research Group, Lusaka, Zambia. ^3^Rwanda Zambia Emory HIV Research Group, Atlanta, USA. ^4^Department of Medicine, University of Alabama at Birmingham, Birmingham, USA

Presenting author email: kristin.wall@gmail.com

**Background**: Genital ulcers are known risk factors for HIV transmission, and reduction of genital ulcers could reduce HIV incidence. However, little is known about risk factors for ulcers, limiting their early identification and treatment.

**Methods**: HIV-serodiscordant heterosexual couples (M+F–, M–F+) were followed with censoring at antiretroviral treatment uptake or HIV transmission (1994–2012). Exposures (demographic, clinical, laboratory) were measured every 3 months. Anderson–Gill survival models evaluated associations between exposures measured during the visit prior to the time-to-undiagnosed genital ulcer outcome (defined as incident syphilis diagnosis via rapid plasma regain test or active ulcer on genital exam).

**Results**: We followed 1393 M+F– couples for 2756 couple-years and 1656 M–F+ couples for 3216 couple-years. The proportion of intervals positive for ulcers were 13.7% for HIV-positive men, 5.6% for HIV– men, 8.5% for HIV+ women, and 4.4% for HIV- women. Risk for genital ulcer for HIV- women was associated (*p* < 0.05) with bilateral inguinal adenopathy (BIA) (adjusted hazard ratio, aHR = 1.9), genital inflammation (GI) (aHR = 1.5–1.9), man’s non-STI GI (aHR = 2.9) and increasing number of previous pregnancies (aHR = 1.1). Risk for genital ulcer for HIV+ women was associated with BIA (aHR = 1.5), GI (aHR-1.5–2.0), man’s non-STI GI (aHR = 2.0), HIV stage III-IV versus I (aHR = 1.5) and being pregnant (aHR = 0.7). Risk for genital ulcer for HIV- men was associated with man’s BIA (aHR = 1.8) and STI GI (aHR = 2.9), woman’s ulcer (aHR = 1.7) and non-STI GI aHR = 1.4), and being uncircumcised (aHR = 1.7); being uncircumcised with foreskin smegma was independently predictive (aHR = 3.2). Risk for genital ulcer for HIV+ men was associated with man’s STI GI (aHR = 2.8), HSV-2-positivity (aHR = 2.5) and HIV stage III-IV versus I (aHR = 1.7); being uncircumcised with foreskin smegma was independently predictive (aHR = 2.4).

**Conclusions**: BIA and GI may be early indicators or risk factors for genital ulceration; importantly, partners’ non-STI GI is also a strong risk factor, and screening of both partners for BIA and GI is indicated. Uncircumcised men with foreskin smegma were at increased risk for genital ulceration. Interestingly, HSV-2-positivity was only predictive of genital ulcer for HIV+ men. Targeted screening among HIV+ individuals with more advanced stage of disease may be worthwhile.

## WEPDD0101

### Community-based testing strategies among sex workers in the transport corridor in Mozambique

E Simons^1^; T Ellman ^2^; R Giuliani^3^; C Bimansha^1^; L O’Connell^1^; E Venables^2^; H Jassitene^1^; C das Dores TP Mosse Lázaro^4^ and M Jose Simango^4^

^1^Medecins Sans Frontieres, Tete, Mozambique. ^2^Medecins Sans Frontieres, Southern Africa Medical Unit, Cape Town, South Africa. ^3^Medecins Sans Frontieres, Maputo, Mozambique. ^4^Ministry of Health, Tete, Mozambique

Presenting author email: tom.ellman@joburg.msf.org

**Background**: The MSF Corridor project aims to implement a comprehensive intervention for sex workers (SW) along the transport corridor in Mozambique and Malawi. The community-based model incorporates outreach services, HIV testing and counseling, condom distribution, retesting for HIV-negative SWs and access to STI and HIV care. Sex worker peer educators (SWPE) play an important role in supporting outreach activities, health education and linkage to care. This analysis describes testing, retesting and seroconversion among SWs in Tete and Sofala, Mozambique and explores SWPE perspectives on their role.

**Methods**: Retrospective analysis of routinely collected data included SWs enrolled in the outreach programme between January 2014 and June 2015. The proportion HIV-positive among SWs who initially tested between January 2014 and June 2015 and the proportion of those initially negative who retested within 6 months were assessed. Seroconversion was determined among those who retested within 6 months. Participant and non-participant observations were conducted during SWPE outreach activities in four project sites, along with nine in-depth interviews and two focus group discussions.

**Results**: 1810 female SWs enrolled, with a median age at first contact of 28 years [23–32]. Among 1207 SWs tested, HIV positivity at initial test was 44%. Overall HIV positivity rate, including 371 additional SWs who self-reported positive, was 57%; 32%, 42%, 61% and 78% among SWs <18, 18–24, 25–34 and ≥35 years, respectively. 42% of SWs initially HIV-negative retested within 6 months and 14 (5%) seroconverted (median time: 114 days). SWPEs described their ability to reach out to their peers, to engage new and ‘informal’ SWs with healthcare services, including HIV testing. Challenges included experiencing prejudice and undervaluation by non-SW colleagues.

**Conclusions**: Despite stigma and mobility challenges, most SWs contacted agreed to test. Among those negative, almost half retested within 6 months. However, retention for retesting remains a major challenge. HIV prevalence and apparent incidence demonstrate the extreme risk among this group and importance of community strategies to access testing, treatment and prevention, including PrEP. SWPEs have a key role in developing trust among their peers and supported uptake of testing and re-testing. Greater efforts are needed to develop their role in SW programmes.

## WEPDD0102

### Views on HIV self-test kit distribution strategies targeting female sex workers: qualitative findings from Zimbabwe

M Tumushime ^1^; N Ruhode^1^; EL Sibanda^1^; M Mutseta^2^; C Watadzaushe^1^; S Gudukeya^2^; M Mapingure^2^; KE Hatzold^2^; M Taegtmeyer^3^; E Corbett^4^; FM Cowan^1,3^ and S Napierala Mavedzenge^5^

^1^Centre for Sexual Health and HIV/AIDS Research (CeSHHAR), Harare, Zimbabwe. ^2^Population Services International (PSI) Zimbabwe, Harare, Zimbabwe. ^3^Liverpool School of Tropical Medicine, Liverpool, UK. ^4^London School of Hygiene and Tropical Medicine, London, UK. ^5^RTI International, San Francisco, USA

Presenting author email: mary@ceshhar.co.zw

**Background**: HIV self-testing (HIVST) may be a suitable strategy to increase HIV testing uptake and frequency among female sex workers (FSW). Optimal ways of distributing test kits to FSW are unclear. We qualitatively explored views on HIVST and distribution methods, amongst FSW and potential self-test kit distributors.

**Methods**: Focus group discussions (FGD) were held among FSW and peer educators (PE), condom-promoting hairdressers and community female condom sales agents (‘Care Promoters’), ≥18 years. Discussions were transcribed and analysed thematically.

**Results**: From September 2016 to January 2017, 15 FGD were conducted across Zimbabwe with 7–10 participants each: 6 each among FSW (*n* = 54) and PE (*n* = 55); 2 among hairdressers (*n* = 16); and 1 among Care Promoters (*n* = 7).

Though knowledge of HIVST was limited, FSW felt it provides increased privacy and convenience. Most were against PE and hairdressers distributing kits, preferring healthcare workers from dedicated FSW clinics to do so and provide HIVST information. Preference for on-site self-testing at these clinics was expressed. Provision of HIVST vouchers for distribution to other FSW was suggested; some PE agreed, proposing they do pre-test HIV counselling alongside.

PE reported HIVST may empower FSW and provide opportunities to test clients/partners. Most were interested in distributing self-test kits if trained, though some preferred clinic distribution. Like FSW, they felt hairdressers should not be distributors.

Hairdressers showed willingness to distribute kits to FSW even at their households; conversely, FSW and PE views were mixed regarding door-to-door distribution, partly due to low prospects of linkage to post-test services. Some thought Care Promoters were better positioned as they already distribute condoms to FSW. Hairdressers expressed a need to be incentivized, seeing self-test kit distribution as an opportunity for additional income.

Care Promoters felt HIVST may increase testing among FSW. They expressed willingness to distribute kits, and like FSW, proposed a voucher system, redeemable at clinics.

**Conclusions**: Though all potential distributors demonstrated willingness, FSW and PE preferred HIVST distribution through FSW clinics where support and post-test services are easily accessible. Distribution of HIVST vouchers also emerged as a potential strategy. These findings will inform scale-up of HIVST distribution targeting FSWs in Zimbabwe.

## WEPDD0103

### Feasibility and acceptability of home-based HIV testing among refugees: a pilot study in Nakivale Refugee Settlement in southwestern Uganda

K O’Laughlin ^1,2,3^; W He^4^; K Greenwald^3^; J Kasozi^5^; Y Chang^3,4^; E Mulogo^6^; Z Faustin^7^; P Njogu^8^; R Walensky^2,3,9^ and I Bassett^2,3,9^

^1^Department of Emergency Medicine, Brigham and Women’s Hospital, Boston, USA. ^2^Massachusetts General Hospital, Medical Practice Evaluation Center, Boston, USA. ^3^Harvard Medical School, Boston, USA. ^4^Massachusetts General Hospital, Division of General Medicine, Boston, USA. ^5^United Nations High Commissioner for Refugees, Kampala, Uganda. ^6^Mbarara University of Science and Technology, Mbarara, Uganda. ^7^Bugema University, Kampala, Uganda. ^8^United Nations High Commissioner for Refugees, Nairobi, Kenya. ^9^Harvard University Center for AIDS Research, Boston, USA

Presenting author email: kolaughlin@bwh.harvard.edu

**Background**: Home-based HIV testing may help reach refugees who face obstacles accessing testing in sub-Saharan Africa. We conducted a pilot study to determine the acceptability and feasibility of home-based HIV screening in Nakivale Refugee Settlement.

**Methods**: From February to March 2014, we visited homes up to 3 times in 3 geographic zones within Nakivale. We enrolled adults 18 years who spoke English, Kinyarwanda, Runyankore or Kiswahili and surveyed them about their country of origin, years in the settlement and reasons for testing acceptance/refusal. We used the proportion of eligible participants present to demonstrate feasibility. The primary outcome was participation in HIV testing and receipt of result. We used logistic regression models with the generalized estimating equation to correlate willingness to test with gender and number of eligible individuals present in the household at time of consent, while taking into account clustering within households.

**Results**: Of 319 homes visited with 566 eligible individuals reported living in these homes, 292 homes (92%) had 507 (90%) individuals present; 353 (70%) present at visit one, 127 (25%) additional people at visit two and 27 (5%) additional people at visit three. Home-based HIV testing participants totaled 378 (75%); all received their results and 7 (1.9%) had new HIV diagnoses. Of participants, 134 (35%) were from the Democratic Republic of the Congo, 129 (34%) from Rwanda, 91 (24%) from Burundi and 23 (6%) from Uganda. Participants were predominantly refugees (93%) and female (56%), with a median age of 30 (IQR 24–40) and a median time of 6 years (IQR 3–8) in Nakivale. Willingness to participate was positively associated with the number of participants at home at time of consent (OR 1.59 [1.17–2.14], *p* = 0.003). Testing was not associated with gender (OR = 1.00 [0.71–1.41], *p* = 0.99). The most common reason for testing was: “to know if I am HIV-infected” (91%).

**Conclusions**: In this home-based HIV testing pilot study, the majority of eligible individuals (75%) participated in HIV testing and received their results. Most participants were reached by the second visit and were more likely to participate when others were present at the time of consent. Home-based HIV testing is feasible and acceptable in a refugee setting.

## WEPDD0104

### Sex, test and treat: implementing an incentivized community-driven intervention to promote the uptake of HIV testing services among clients of sex workers

TN Flavien ^1^; F Ghislaine^1^; N Denise^2^; G Honorat^1^; S Billong^3^; JB Elat M^3^; D Levitt^4^ and S Baral^5^

^1^CARE, CHAMP, Yaounde, Cameroon. ^2^Horizons Femmes, Yaounde, Cameroon. ^3^National Aids Control Committee, Yaounde, Cameroon. ^4^CARE International, Washington, USA. ^5^Department of Epidemiology, Johns Hopkins Bloomberg School of Public Health, Center for Public Health & Human Rights, Baltimore, USA

Presenting author email: flavienndonko@gmail.com

**Background**: Female sex workers (FSW) and their clients face elevated risks of acquisition and transmission of HIV and other sexually transmitted infections globally. In Cameroon, HIV prevalence among FSWs is estimated at 36%, and HIV testing uptake among clients of FSW is suboptimal. To increase uptake of HIV testing services (HTS) among FSW clients, the USAID-funded *Continuum of HIV/AIDS prevention, care and treatment for Most-at-risk Populations* (CHAMP) programme developed a community-led referral recruitment approach. FSWs were provided a small incentive to promote HTS to their clients, who could access the services directly at, or near sex work hotspots.

**Methods**: FSW at high-volume hotspots were given coupons with unique identifier codes (UICs) and basic training to refer clients to an HTS counsellor (and lab technician) located in a room nearby. FSW received USD$1 for each client who agreed to take a test. Each client who accessed HTS was assigned a UIC. Quantitative client data were collected via the CommCare digital platform and analysed for the period September 2015 through December 2016. In addition, the team collected qualitative data via in-depth interviews with 30 clients who attended HTS.

**Results**: Prior to the implementation of this innovation, HTS uptake averaged less than 100 FSW clients per month. Recruitment and referral through FSW increased HTS uptake among clients dramatically (1071 clients of FSW were tested in just one month, with a yield of 4.8% living with HIV). Most clients who agreed to be tested noted the discrete environment and time saved as reasons for testing, as compared to mass screening campaigns. FSW expressed satisfaction and pride serving as community mobilizers.

**Abstract WEPDD0104–Figure 1.**
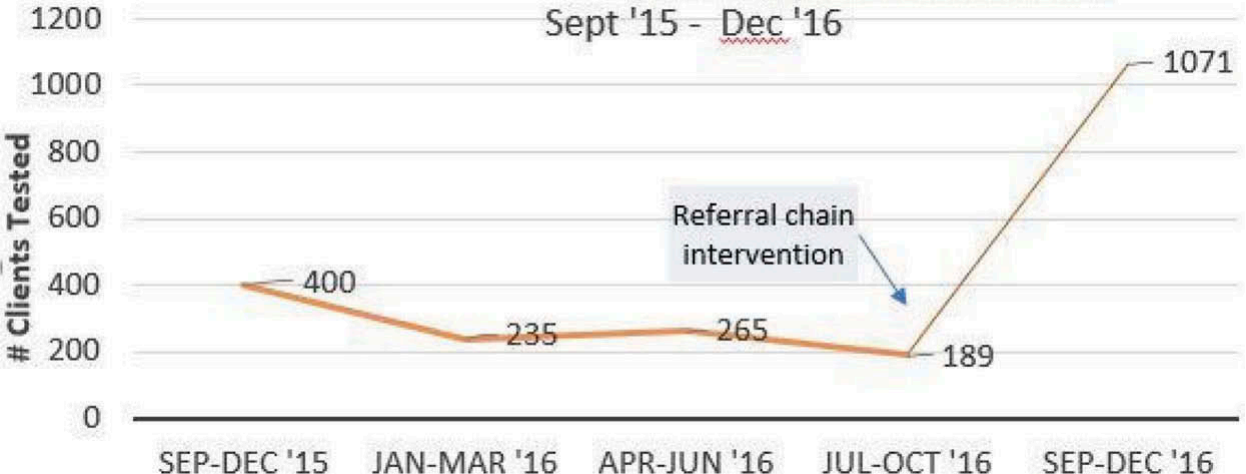
Clients of FSW – HTC uptake – CHAMP Cameroon.

**Conclusions**: Incentivized, FSW community-led referral recruitment approaches may have strong potential to identify clients more likely to be living with HIV, effectively recruit them into the services network, and concurrently engender building of social capital among FSW.

## WEPDD0105

### Implementing test and start programme in a rural conflict affected area of south Sudan: the experience of Médecins Sans Frontières

MC Ferreyra Arellano ^1^; B Oulo^2^; E Grandio^2^ and V Achut^3^

^1^Medical Department, Medecins Sans Frontieres, Barcelona, Spain. ^2^Medecins Sans Frontieres, Juba, South Sudan. ^3^Ministry of health South Sudan, HIV/AIDS/STI, Juba, South Sudan

Presenting author email: cecilia.ferreyra@barcelona.msf.org

**Background**: Community-based HIV counselling and testing (CB-HCT) and early initiation of antiretroviral therapy (ART) reduce HIV transmission and mortality. Access to HIV care in settings with low ART coverage and/or affected by conflict is low; innovative strategies are needed to increase HIV care and ensure continuation of ART in case of instability. A pilot test and start project was implemented in rural areas of Yambio South Sudan, a chronically conflict-affected area aiming to determine feasibility and acceptability of this intervention.

**Methods**: Data from July 2015 to December 2016 was analysed. The project involved five mobile teams offering HCT and same-day ART initiation at community level. Contingency plan included delivery of key messages on ‘what to do in case of conflict’ during counselling sessions and coordination with community health workers (CHWs) to distribute ‘run-away bags’ with 3 months of ART. Several episodes of acute instability occurred during this period which needed to activate the plan to ensure that patients would not interrupt their treatment.

**Results**: During this period, 13,872 people were tested; 442 (3.2%) was found to be HIV positive and 344 (77.8%) started on ART. 224 (54.4%) where women with a mean age of 33 years, 207 (60.2%) had CD4 count below 500 cells/µl. By December 2016, 67 (19.5%) patients were loss of follow-up, 8 (2.3 %) died. Retention in care at 6 and 12 months of follow-up was, respectively, 291(84%) and 277(81%) patients. 114 patients with available viral load results (85.7%) had VL less than 1000 copies/ml after 6 months of ART. At 17 months, 251 (73%) patients are still under follow-up and on ART.

**Conclusions**: Our programme shows a high level of acceptance to HCT and early ART initiation despite rural context and security situation. Early results show retention in care and virological suppression outcomes comparable with HIV programmes at clinic level and without security issues. We believe this strategy could be extrapolated to other contexts with low access to ART and instability.

## WEPDX0101

### Phylogenetic, epidemiological and virological insights on the rise of large cluster outbreaks fuelling the HIV-1 epidemic among men having sex with men within Quebec

B Brenner ^1^; R-I Ibanescu^2^; M Roger^3^; M Oliveira^4^; I Hardy^3^; M Wainberg^5^; Montreal PHI and SPOT cohort study groups

^1^McGill University, Medicine & McGill AIDS Centre, Montreal, Canada. ^2^Lady Davis Instutute, Montreal, Canada. ^3^Universite de Montreal, Montreal, Canada. ^4^Lady Davis Institue, Montreal, Canada. ^5^McGill University, Montreal, Canada

Presenting author email: bluma.brenner@mcgill.ca

**Background**: HIV-1 epidemics remain uncontrolled among Men having Sex with Men (MSM) within Quebec in the era of treatment-as-prevention. Phylogenetics infer two patterns of HIV-1 spread among MSM, each contributing to half of the epidemic. While the majority (95%) of HIV-1 strains lead to self-limiting clusters (size 1–4), 32 viruses contributed to micro-epidemics (cluster size 20–140) disproportionately rising from 13%, 25% and 42% of diagnoses in 2004–2007, 2008–2011 and 2012–2015, respectively. Here, phylogenetic, epidemiological and virological data deduced factors favouring the selective advantage of large cluster variants.

**Methods**: Population-level phylogenetics across the RT/protease domain deduced temporal dynamics of HIV-1 clustering. Phylogenetics across the viral integrase and V3 loop was performed on representative clusters. Epidemiological data from the SPOT rapid testing site and Montreal primary HIV cohort deduced epidemiological and behavioural risk effects implicated in clustering. Primary HIV-1 strains from MSM associated with large 20+ clusters or singleton/small clusters (cluster size 1–4) were grown in cell culture under dolutegravir (DTG), elvitegravir (EVG) and/or lamivudine (3TC) pressures. Sanger and deep sequencing assessed HIV-1 genotypic changes under drug pressure.

**Results**: Phylodynamics charted the introduction and spread of 32 large clusters (median cluster size 20–140), over median 2-year periods. Clusters were concentrated in Montreal with several clusters in Quebec City and Sherbrooke. Three large clusters (cluster size 38, 43, 45), with median transmission dates of June 2010, February 2012 and October 2013, shared a common integrase. Large cluster strains (*n* = 11) were resilient showing accelerated acquisition of resistance within 5–8 weeks to DTG, EVG and 3TC compared to small cluster strains where resistance arose after 20 weeks. Several large clusters displayed dual X4/R5 tropism. Large clusters were arising in younger persons with 29%, 35% and 41% under 30 years of age over the 2004–2007, 2008–2011 and 2012–2015 periods. Only 48% of infections within large clusters were first genotyped in primary/recent infection. HIV-1 testing habits remain poor and significantly better for persons reporting multiple anonymous partnerships than those reporting low-risk behaviour.

**Conclusions**: HIV-1 continues to spread among MSM with an alarming shift towards large cluster outbreaks, emphasizing the need for improved prevention paradigms.

## WEPDX0102

### Transmission cluster-specific pattern of adaptive evolution of the HIV-1 envelope gp120 protein sequence in a Japanese MSM population

T Shiino ^1^; M Matsuda^2^; A Hachiya^2^; W Sugiura^3^; Y Yokomaku^2^; Y Iwatani^2^; K Yoshimura^4^; The Japanese Drug Resistance HIV-1 Surveillance Network

^1^National Institute of Infectious Diseases, Infectious Disease Surveillance Center, Shinjuku-ku, Japan. ^2^Nagoya Medical Center, Clinical Research Center, Nagoya, Japan. ^3^Gla, Shinjuku-ku, Japan. ^4^National Institute of Infectious Diseases, AIDS Research Center, Shinjuku-ku, Japan

Presenting author email: tshiino@nih.go.jp

**Background**: HIV-1 envelope protein (Env) is the target of neutralizing antibodies (NAbs), which shows a potential in the treatment and prevention of HIV infection. To combat against the rapid evolution of the Env gene during the HIV transmission chain, development of various broad NAbs corresponding to the genetic diversity is required. Recently, we have identified unique transmission clusters (TCs) of subtype B circulating in Japan based on their protease-RT gene sequences. In this study, we analysed adaptive evolution of the Env sequences in the TCs to further characterize the immune escape of the Env gene within a TC.

**Methods**: We determined 2673 C2-V5 sequences of Env gp120 from 712 individuals who underwent tropism testing between 2011 and 2015 at Nagoya Medical Center, the second largest medical facility participating in the Japanese Drug Resistance HIV-1 Surveillance Network. The TC of each sequence was identified by searching our TC database, and was confirmed by inferring the phylogenies of gp120 sequences. Neutral evolution of each region within each TC was tested by Kumar’s Z-test for the null hypothesis of strict neutrality. Codon-by-codon neutral selection was analysed by maximum-likelihood computations of synonymous and non-synonymous substitutions per site using the HyPhy software package.

**Results**: We identified 89 TCs, 77 of which belong to subtype B associated with the MSM population. The nucleotide diversity of the C3 region was greater than that of the V3 region and was similar to that of the V4 region. Regions with greater diversity exhibited significantly positive selection during the evolution of subtypes, whereas the directions of selection during the evolution of TCs were much more variable. Furthermore, different amino acid sites in the C3 region were under positive selection during the evolution of each TC. Viruses circulating in each TC of gp120 have a distinct substitution pattern to confer escape from NAbs.

**Conclusions**: Our study indicates that a prevention programme targeting a key population using an effective broad NAb may not work efficiently for another patient group. Identification and characterization of the HIV transmission network is thus crucial to choose the most appropriate NAb for an antibody-mediated prevention strategy targeting a local key population.

## WEPDX0103

### Analysis of US HIV sequence data indicates that recent and rapid HIV transmission is focused among young Hispanic/Latino men who have sex with men

AM Oster ^1^; AM France^1^; N Panneer^1^; JO Wertheim^2^; MC Ocfemia^1^; S Dasgupta^1^; AL Hernandez^1^

^1^Centers for Disease Control and Prevention, Division of HIV/AIDS Prevention, Atlanta, USA. ^2^Department of Medicine, University of California, San Diego, San Diego, USA

Presenting author email: aoster@cdc.gov

**Background**: Although the estimated rate of HIV transmission in the United States is approximately 4 transmission events per 100 person-years, the rate of transmission in some risk networks is likely much higher. Identifying these networks can provide critical data for focusing efforts on populations in need of the most intensive prevention interventions. To describe the leading edge of HIV transmission, we identified molecular clusters with recent and rapid growth, determined the transmission rate for these clusters and described the persons involved in rapid transmission.

**Methods**: We analysed baseline partial HIV-1 polymerase sequences reported to the National HIV Surveillance System through December 2015 by 27 participating jurisdictions for persons with HIV diagnosed during 2013–2015. We calculated genetic distance for each pair of sequences. Using a pairwise threshold of 0.005 substitutions/site, we inferred clusters and identified rapidly growing clusters (those with ≥5 diagnoses during 2015). We used node ages determined through molecular clock phylogenetic analysis to calculate HIV transmission rates for these rapidly growing clusters and compared persons in these clusters to other persons with sequences included in the analysis, accounting for correlation between cases in the same cluster.

**Results**: Sequences were analysed for 30,323 persons; 13 rapidly growing clusters were identified. These clusters had a transmission rate of 34/100 person-years. Compared with the 30,127 persons not in these clusters, the 196 persons in these 13 clusters were disproportionately men who have sex with men (MSM) (94% vs. 62%, *p* < 0.0001), aged <30 years (68% vs. 41%, *p* < 0.0001), Hispanic/Latino (49% vs. 28%, *p* < 0.0001), and, specifically, young Hispanic/Latino MSM (32% vs. 9%, *p* < 0.0001). The clusters included high levels of transmitted drug resistance (43% vs. 20%, *p* = 0.0006).

**Conclusions**: This approach identified a small number of clusters with a transmission rate more than 8 times that of previous national estimates. These findings highlight the extent of rapid transmission among young Hispanic/Latino MSM, suggesting the need for prevention efforts that focus on this population. Identifying clusters of active transmission can help programmes effectively direct limited public health resources.

## WEPDX0104

### Phylodynamic insights into HIV epidemic dynamics within Canada

J Joy ^1^; R Liang^1^; T Nguyen^1^; R McCloskey^1^; B Brenner^2^; T Lynch^3^; J Gill^4^; J Buller^5^; Z Brumme^6^; A Burchell^7^; S Rourke^8^; M Loutfy^9^; J Raboud^7^; C Cooper^10^; D Kelly^11^; C Tsoukas^12^; N Machouf^13^; M Klein^12^; A Wong^14^; P Levett^15^; S Hosein^16^; M Wainberg^2^; P Sandstrom^17^; J Montaner^1^; R Hogg^1^; A Poon^18^; PR Harrigan^1^; CANOC Collaboration

^1^BC Centre for Excellence in HIV/AIDS, Vancouver, Canada. ^2^McGill University, Montreal, Canada. ^3^Department of Pathology and Laboratory Medicine, University of Calgary, Calgary, Canada. ^4^University of Calgary, Microbiology, Immunology and Infectious Diseases, Calgary, Canada. ^5^University of Manitoba, Cadham Provincial Laboratory, Winnipeg, Canada. ^6^Simon Fraser University, Faculty of Health Sciences, Burnaby, Canada. ^7^University of Toronto, Dalla Lana School of Public Health, Toronto, Canada. ^8^University of Toronto, Psychiatry, Toronto, Canada. ^9^Women’s College Research Institute, Toronto, Canada. ^10^Department of Medicine, University of Ottawa, Ottawa, Canada. ^11^Memorial University, St. Johns, Canada. ^12^McGill University Health Centre, Montreal, Canada. ^13^Clinique Medicale l’Actuel, Montreal, Canada. ^14^University of Saskatchewan, Regina, Canada. ^15^University of Regina, Regina, Canada. ^16^CATIE, Science and Medicine, Toronto, Canada. ^17^Public Health Agency of Canada, Winnipeg, Canada. ^18^Department of Pathology and Laboratory Medicine, University of Western Ontario, London, Canada

Presenting author email: jjoy@cfenet.ubc.ca

**Background**: Despite the importance of HIV, the early history, geographic dissemination and dynamics of the virus within populations across Canada remain unclear. Epidemiological processes stamp measurable signatures on HIV genomes sampled at different places and times. Using statistical phylodynamic approaches applied to HIV sequences sampled from across Canada, we test hypotheses concerning past and present HIV epidemic dynamics.

**Methods**: We compiled 51,493 doubly anonymized HIV pol sequences from 20,000+ patients annotated with clinical and socio-demographic parameters. Data were available from 5 Canadian provinces: British Columbia, Alberta, Saskatchewan, Ontario and Quebec. Analyses were restricted to the first sample collected from each patient and drug resistance codons were censored from the alignment. Phylogenetic trees were inferred using FastTree2. Phylogenetic clusters of five or more participants were identified using a tip-to-tip distance cutoff <0.02 substitutions per site. Diversification rate and phylogeographic analyses were conducted in R and BEAST, respectively.

**Results**: We observed variation among provinces in the proportion of non-subtype B infections, with the Prairies displaying significantly greater numbers of non-B infections (*p* < 0.05). We recovered 285 clusters of size ≥5 (Figure 1). Cluster size was associated with proportion of people who inject drugs (*p* < 0.004). Most provinces contain large, primarily province specific, clusters dominated by transmission through injection drug use. Some between-province clustering is observed (*n* = 55 clusters including 3 or more provinces). Association of clusters with more than one province was associated with proportion MSM risk factor (*p* < 0.05). Consistent with other evidence, the Prairies had the highest rates of HIV diversification.

**Conclusions**: Secondary analysis of genotypic resistance data provides useful epidemiological inferences on a national scale. Different circumstances permitted establishment, dissemination and growth of the HIV epidemic in Canada at different times within component subpopulations. Our results emphasize the varied challenges facing different regions of Canada in controlling the HIV epidemic in the future.

**Abstract WEPDX0104–Figure 1.**
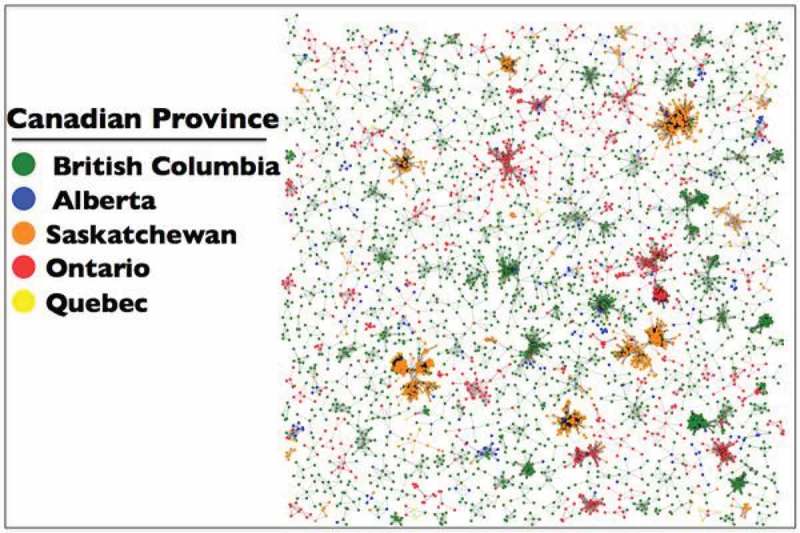
Phylogenetic clusters of HIV in Canada.

## WEPDX0105

### Phylogenetics between and within hosts along the genome reveals transmission, dual infections, recombination and contamination

CM Wymant ^1,2^; M Hall^1,2^; F Blanquart^2^; O Ratmann^2^ and C Fraser^1,2^

^1^Nuffield Department of Medicine, University of Oxford, Big Data Institute, Oxford, UK. ^2^Imperial College London, Infectious Disease Epidemiology, London, UK

**Background**: Next-generation sequencing (NGS) has transformed genomics for many pathogens, increasing the availability of sequence data for studying evolution, epidemiology, vaccines and therapeutic design. However, on the comprehensive Los Alamos National Laboratory database, more than 90% of HIV sequences were generated with the older method of Sanger sequencing. Widespread adoption of NGS may have been hindered by the technical difficulty of reconstructing and interpreting quasispecies data from reads (short fragments of DNA), given the high diversity of HIV between and within hosts.

**Methods**: Our public computer program ‘shiver’ reconstructs whole HIV genomes using two commands, by mapping (aligning) reads to a reference genome constructed specifically for each sample to maximize accuracy, then finding the consensus of the reads. Our program ‘phyloscanner’ interprets mapped reads with a single command. This extracts all patients’ reads in a sliding window of the genome, processes and aligns them, constructs a phylogeny with RAxML, analyses the diversity within and between hosts, performs ancestral host-state reconstruction and produces a per-patient summary. Contamination is detected by comparing reads between patients and by finding phylogenetic outliers. We used these two programs on new whole-genome NGS data for 24 seroconverters from European and African cohorts: 5 each from subtypes A1, B, C and D, and 1 each from 01_AE, 01_AG, F1 and G.

**Abstract WEPDX0105–Figure 1.**
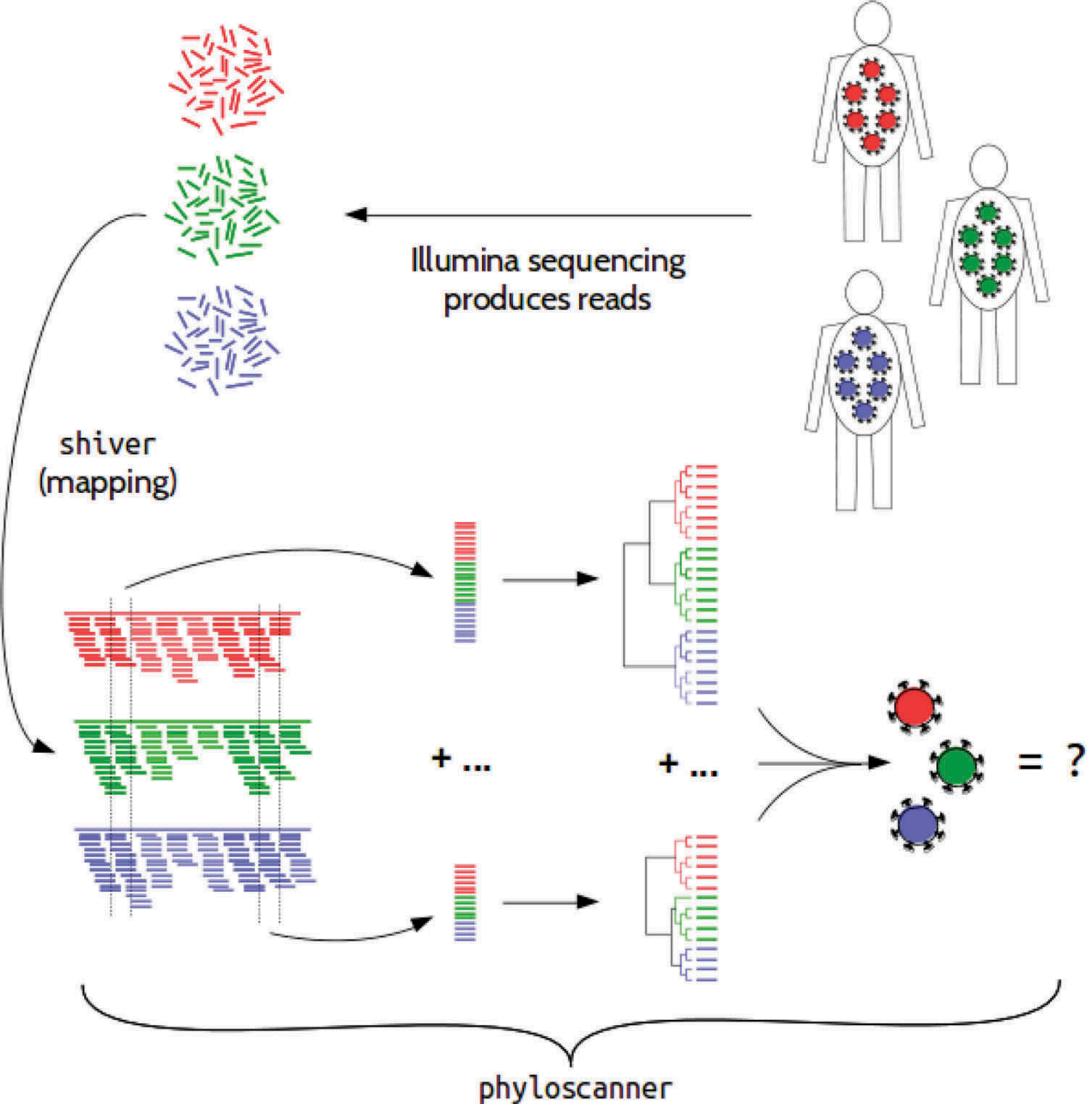
Understanding NGS data for HIV.

**Results**: In our data, we found donor-recipient pairs with the direction of transmission suggested by the host-state reconstruction, identifying ancestry between quasispecies. (Unsampled intermediate patients are always possible.) Variability across the genome highlighted the importance of whole genomes and many genomic windows for detecting transmission from sequence data. Dually infected individuals had two distinct phylogenetic clusters of reads. Phylogenetic intermediates showed where these two strains recombined in the host.

**Conclusions**: Raw NGS data for HIV can be analysed easily and powerfully with our public tools phyloscanner and shiver.

## WEPDX0106

### Deep analysis of HIV transmission chains: input of ultra-deep sequencing

E Todesco ^1,2^; M Wirden^1,2^; S Lambert^1,2^; A Simon^3^; C Soulié^1^; C Katlama^1,4^; V Calvez^1,2^; A-G Marcelin^1,2^ and S Hué^5^

^1^Sorbonne Universités, UPMC Univ Paris 06, INSERM, Institut Pierre Louis d’épidémiologie et de Santé Publique (IPLESP UMRS 1136), F75013, Paris, France. ^2^Department of Virology, Hôpital Pitié-Salpêtrière, AP-HP, F75013, Paris, France. ^3^Department of Internal Medicine, Hôpital Pitié-Salpêtrière, AP-HP, Paris, France. ^4^Department of Infectious Diseases, Hôpital Pitié-Salpêtrière, AP-HP, Paris, France. ^5^Department of Infectious Disease Epidemiology, London School of Hygiene and Tropical Medicine, London, UK

Presenting author email: eve.todesco@aphp.fr

**Background**: Many studies have shown clustering in recently HIV-1 infected Men having Sex with Men (MSM) but few data are available on the link with transmitted drug resistance (TDR). Moreover, the added value of Ultra Deep Sequencing (UDS) over Sanger sequencing (SS) for phylogenetic HIV transmission studies needs to be determined. We explored the epidemiological linkage between HIV-infected MSM using both sequencing approaches, and evaluated its impact on TDR detection.

**Methods**: Reverse transcriptase, protease and integrase sequences were obtained by SS and UDS from 70 HIV-1 infected, treatment-naive MSM diagnosed between January 2012 and July 2013 in Paris. Maximum likelihood phylogenies were estimated using FastTree and RAxML, with SH-like tests and 1000 bootstrap replicates, respectively, with both data sets. Transmission events were identified as clades with branch support ≥70% and intra-clade genetic difference <2.5%. TDR mutations were recognized from the consensus list of TDR surveillance.

**Results**: Median time between the HIV-1 diagnosis and date of the sample used for genotypic analysis was 12 days; 5 diagnosis occurred during the acute infection stage.

SS and UDS data concurred in the identification of 7 transmission pairs and 1 cluster of 3 patients. With UDS, direct linkage and direction of transmission was unambiguously inferred in 3/8 and 1/8 clusters, respectively. Sequences from the putative recipient were polyphyletic for 4/8 clusters, suggesting multiple founder viruses and excluding unobserved, intermediary links. By SS, the prevalence of TDR mutations was 5.7% in the unlinked patients and 0.0% in the linked patients (13.2% and 35.3% by UDS, respectively). Minority-resistant variants were not shared among the transmissions chains.

**Conclusions**: While SS and UDS identified the same transmission chains, UDS provided extra information on founder viruses, linkage and levels of TDR. No mutation within the clusters was associated with reduced efficiency of PrEP, even by UDS. Moreover, no sharing of minority-resistant variants was observed among the chains of transmission. These results highlight the benefits of UDS data in the phylogenetic identification of transmission chains, allowing the inference of direct linkage and multiplicity of founder viruses in the recipients, and potentially of direction of transmission.

## LATE BREAKER ORAL ABSTRACTS

## MOAA0106LB

### Enrichment of the HIV reservoir in CD32+ CD4 T cells occurs early and is closely associated with immune checkpoint receptor expression

GE Martin ^1^; M Pace^1,2^; JP Thornhill^1,3^; C Phetsouphanh^1^; E Hopkins^1^; J Meyerowitz^1^; N Nwokolo^4^; J Fox^5^; C Willberg^1,6^; P Klenerman^1,6^; S Fidler^3^; J Frater^1,2,6^; CHERUB Investigators

^1^Nuffield Department of Medicine, University of Oxford, Oxford, UK. ^2^Oxford Martin School, Oxford, UK. ^3^Division of Medicine, Wright Fleming Institute, Imperial College, London, UK. ^4^Chelsea and Westminster Hospital, London, UK. ^5^Department of Genitourinary Medicine and Infectious Disease, Guys and St Thomas’ NHS Trust, London, UK. ^6^National Institute of Health Research Biomedical Research Centre, Oxford, UK

Presenting author email: genevieve.martin@ndm.ox.ac.uk

**Background**: CD32 has been identified as a marker of a substantially enriched HIV reservoir. Here, we explore the relationship of CD32 expression on CD4 T cells with other correlates of reservoir size including time to viral rebound after treatment interruption.

**Methods**: CD32 expression was measured by flow cytometry on PBMCs (*n* = 39) and tonsillar tissue (*n* = 1) from individuals who initiated ART during primary HIV infection (PHI), and uninfected controls (*n* = 10). Co-expression with immune checkpoint receptors (ICRs), lineage, memory and T follicular helper (Tfh) markers was measured. HIV DNA was quantified in bulk and sorted CD32+ and CD32– populations.

**Results**: One-year post-ART initiation, the frequency of CD32+ CD4 T cells was 1.5% (range 0.2–6.4), and did not differ from controls. CD32+ CD4 T cells were found predominantly within differentiated memory subsets (transitional, effector memory and T_EMRA_) compared with CD32- CD4 T cells (all *p* < 0.001) for HIV+ (*n* = 20) and controls. CD32+ CD4 T cells were highly enriched for HIV DNA compared with CD32– cells (average 103-fold, *n* = 6, *p* = 0.03), although CD32 percentage did not correlate with reservoir size (*n* = 29). In a subset of individuals (*n* = 19) who interrupted ART after 48 weeks, CD32+ CD4% did not predict viral load rebound, although all three individuals with persistently undetectable viraemia had CD32+ CD4% below the median.

CD32+ CD4 T cells from blood had higher expression of PD-1, Tim-3 and TIGIT (all *p* < 0.0001) and a higher density of CD2 (*p* = 0.001) than CD32- cells in HIV+ participants (*n* = 20) and controls. Tonsil CD32+ CD4 T cells (*n* = 1) showed a similar pattern of memory distribution and ICR expression as the periphery. Although tonsillar CD32+ CD4 T cells had higher individual expression of Bcl-6, ICOS and CXCR5 than CD32- cells, the co-expression pattern was not consistent with a Tfh phenotype.

**Conclusions**: We confirm the role of CD32 as a marker of the HIV reservoir, and show that this may occur early during PHI on more differentiated CD4 T cells and is highly co-expressed with ICRs. That expression is similar between HIV+ and HIV– individuals and suggests that preferential infection or survival of CD32+ cells, rather than CD32 up-regulation, is responsible for the observed enrichment.

## MOAB0105LB

### A Phase 3 randomized controlled clinical trial of bictegravir in a fixed dose combination, B/F/TAF, vs ABC/DTG/3TC in treatment-naive adults at Week 48

J Gallant ^1^; A Lazzarin^2^; A Mills^3^; C Orkin^4^; D Podzamczer^5^; P Tebas^6^; P-M Girard^7^; I Brar^8^; E Daar^9^; D Wohl^10^; J Rockstroh^11^; X Wei^12^; K White^12^; H Martin^12^; E Quirk^12^ and A Cheng^12^

^1^Southwest CARE Center, Santa Fe, USA. ^2^IRCCS Ospedale San Raffaele SrL, Milan, Italy. ^3^Mills Clinical Research, Los Angeles, USA. ^4^Barts Health NHS Trust, Royal London Hospital, Ambrose King Centre, London, UK. ^5^Hospital Universitari de Bellvitge, Barcelona, Spain. ^6^University of Pennsylvania, Philadelphia, USA. ^7^Hopital Saint Antoine, Paris, France. ^8^Henry Ford Hospital, Detroit, USA. ^9^Los Angeles Biomedical Research Institute at Harbor-UCLA Medical Center, Torrance, USA. ^10^University of North Carolina at Chapel Hill, Chapel Hill, USA. ^11^University Hospital Bonn, Bonn, Germany. ^12^Gilead Sciences Inc., Foster City, USA

Presenting author email: jgallant@southwestcare.org

**Background**: Integrase strand transfer inhibitors (INSTIs) are recommended as first-line antiretroviral therapy in combination with 2 nucleoside reverse transcriptase inhibitors. Bictegravir (B), a novel, potent INSTI with a high in vitro barrier to resistance and low potential for drug interactions, has been coformulated with emtricitabine (F) and tenofovir alafenamide (TAF) as a fixed-dose combination (B/F/TAF). We report results from a blinded Phase 3 study comparing B/F/TAF to coformulated abacavir, dolutegravir and lamivudine (ABC/DTG/3TC).

**Methods**: HIV-infected, treatment-naive, HLA-B*5701-negative, HBV-uninfected adults with estimated glomerular filtration rate (eGFR) ≥50 mL/min were randomized 1:1 to receive blinded treatment with fixed-dose combination B/F/TAF (50/200/25mg) or ABC/DTG/3TC (600/50/300mg) with matching placebos once daily. The primary endpoint was proportion of participants with HIV-1 RNA (VL) <50 c/mL at W48 (FDA snapshot). Noninferiority was assessed through 95.002% confidence intervals (CI) (12% margin). Secondary endpoints were safety (adverse events (AEs) and laboratory abnormalities) and pre-defined analyses of changes from baseline in bone mineral density (BMD) and measures of renal function, including eGFR and proteinuria.

**Results**: 629 participants were randomized and treated (314 B/F/TAF, 315 ABC/DTG/3TC): 10% women, 36% Black, 16% VL >100,000 c/mL, 11% CD4 <200 cells/mL. Median baseline characteristics: age 32 yrs, CD4 count 444 cells/µL and VL 4.47 log10 c/mL. At W48, B/F/TAF was non-inferior to ABC/DTG/3TC, with 92.4% on B/F/TAF and 93.0% on ABC/DTG/3TC achieving HIV-1 RNA <50 c/mL (difference −0.6%; 95.002% CI −4.8% to 3.6%, *p* = 0.78). No resistance mutations emerged in either group. Comparing B/F/TAF to ABC/DTG/3TC throughout, the most common AEs were diarrhoea (13%, 13%), headache (11%, 14%) and nausea (10%, 23%). Few participants (0 vs. 4 (1%)) had any AEs leading to premature study drug discontinuation. At W48, mean percentage changes from baseline in BMD were −0.83% versus −0.60% (*p* = 0.39) (lumbar spine) and −0.78% versus −1.02% (*p* = 0.23) (total hip). No differences between treatments were noted in changes from baseline for eGFR and proteinuria at W48.

**Conclusions**: At W48, B/F/TAF achieved virologic suppression in 92.4% of treatment-naive adults and was noninferior to ABC/DTG/3TC, with no emergent resistance. B/F/TAF was safe and well tolerated with less nausea than ABC/DTG/3TC. Bone and renal safety profiles were similar between groups.

## MOAB0106LB

### Dual therapy with darunavir/ritonavir plus lamivudine for HIV-1 treatment initiation: Week 24 results of the randomized ANDES study

O Sued^1^; MI Figueroa^1^; A Gun^2^; W Belloso^3^; D Cecchini^4^; G Lopardo^5^; D Pryluka^6^; MJ Rolon^1^; V Fink^1^; S Perez Lloret^7^ and P Cahn ^1^

^1^Fundacion Huesped, Clinical Research, Buenos Aires, Argentina. ^2^Fundacion Huesped, Laboratory research, Buenos Aires, Argentina. ^3^Hospital Italiano, Clinical Research, Buenos Aires, Argentina. ^4^Hospital Cosme Argerich, Infectious Diseases Unit, Buenos Aires, Argentina. ^5^Centro de Estudios Infectologicos SA (CTD Stamboulian), Buenos Aires, Argentina. ^6^Consultorio Infectológico Dr. Pryluka, Buenos Aires, Argentina. ^7^Institute of Cardiology Research, University of Buenos Aires, National Research Council (CONICET-ININCA), Buenos Aires, Argentina

Presenting author email: omar.sued@huesped.org.ar

**Background**: Following the results of the GARDEL trial, dual therapy (DT) has been explored in different studies. Generic fixed dose combinations (FDC) of Darunavir/ritonavir (DRV/RTV) 800/100mg and Tenofovir/Lamivudine(TDF/3TC) are available in Argentina. This study compares DRV/rtv plus 3TC to standard-of care triple therapy (TT) based on these same drugs plus Tenofovir (TDF).

**Methods**: ANDES is a randomized, open-label, Phase IV study, designed to assess the antiviral efficacy, safety and tolerability of DT with DRV/rtv (800/100mg) FDC, plus 3TC (300mg), compared to TT with DRV/RTV (800/100mg) plus TDF/3TC (300/300mg) FDC in treatment-naive HIV-1-infected patients. Primary endpoint: proportion of patients with viral load (pVL) <50 copies/mL at Week 48. Preplanned analyses at Week 24, measured by the proportion of patients with pVL <400 copies/mL (ITT-exposed analysis, FDA snapshot algorithm) are reported. ClinicalTrials.gov Identifier:NCT02770508.

**Results**: Out of 182 patients screened, 145 were randomized to receive: DT (*n*:75) or TT (*n*:70). Screening failure rate was 20%. At baseline: 91% were male; median age 30 years; CDC stage A: 92%; 24% had pVL >100,000 copies/mL. At Week 24, 94.7% (*n*:71) of patients receiving DT and 97.1% (*n*:68) receiving TT were responders (pVL <400 copies/mL), difference −2.5% (95% CI: −7.9 to 2.9) Patients with baseline pVL >100,000 copies/mL (*n*:35) showed 100% response in both arms. One patient had virological failure at W24 due to non-compliance (control arm). Mean CD4+ increases were similar in both arms (DT = 206 cells/mm^3^; TT = 204 cells/mm^3^).Sixty-seven Grade 2–3 possible/probable related adverse events (AEs) were reported in 51 patients (36%), most frequent were gastrointestinal (22%) and rash (14%). AE incidence was similar in both arms; one patient was discontinued due to a drug-related Grade 3 adverse event (rash).

**Conclusions**: A generic combination of DRV/RTV in fixed-dose plus 3TC showed non-inferiority to a generic triple drug regimen of DRV/RTV plus TDF/3TC at 24 weeks. These results, if confirmed at Week 48, may provide further evidence about the potential efficacy of dual therapy based on 3TC and a drug with a high genetic barrier.

## MOAD0106LB

### Retention in community versus clinic-based adherence clubs for stable ART patients in South Africa: 24 month final outcomes from a randomized controlled trial

C Hanrahan ^1^; S Schwartz^1^; V Keyser^2^; M Mudavanhu^2^; N West^1^; L Mutunga^2^; J Steingo^2^; J Bassett^2^ and A Van Rie^3^

^1^Johns Hopkins Bloomberg School of Public Health, Epidemiology, Baltimore, USA. ^2^Witkoppen Health and Welfare Centre, Johannesburg, South Africa. ^3^University of Antwerp, Antwerp, Belgium

Presenting author email: chanrah1@jhu.edu

**Background**: Adherence clubs, where groups of 25–30 patients stable on antiretroviral therapy (ART) meet for counselling and medication pick-up, are an innovative model to retain patients in care and facilitate task-shifting. Adherence clubs can be organized at a clinic or community venue. We performed a randomized controlled trial to compare club retention between community and clinic-based adherence clubs.

**Methods**: Stable patients with undetectable viral load at Witkoppen Clinic in Johannesburg, South Africa, were randomized to a clinic- or community-based adherence club. Clubs were held every other month. All club participants received annual viral load monitoring and medical exam at the clinic. Patients were referred back to standard clinic-based care if they missed a club visit without ART pickup within 5 days, had two consecutive late ART pickups, developed a comorbidity requiring closer monitoring or had viral rebound. We assessed the proportion referred back to routine care by 24 months following randomization.

**Results**: From February 2014 to May 2015, we randomized 775 adults into 12 pairs of clubs – 376 (49%) clinic-based, and 399 (51%) community-based. Characteristics were similar by arm: 65% female, 89% on fixed-dose combination ART and median CD4 count of 506 cells/mm^3^. The proportion referred back to standard clinic-based care was greater among community-based (47%, *n* = 191) compared to clinic-based clubs (37%, *n* = 140, *p* = 0.003) (Figure 1).

**Abstract:**
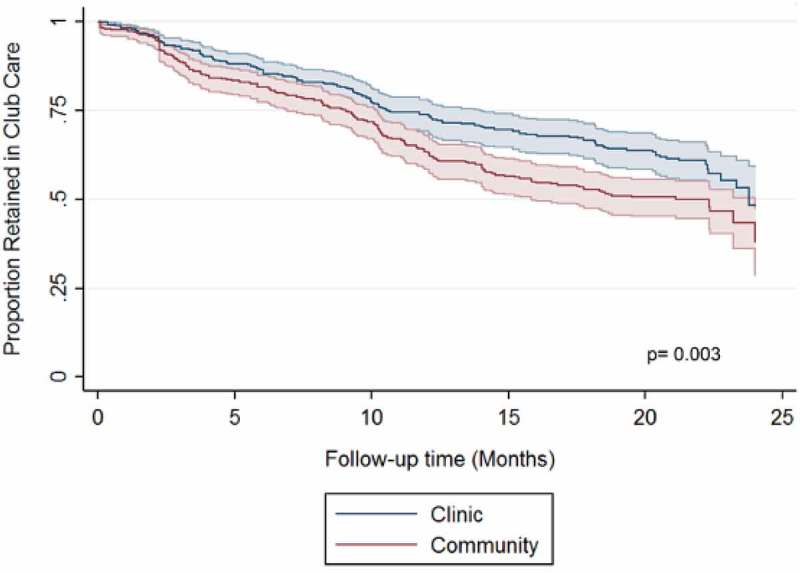
MOAD0106LB–Figure 1.

Adjusted for age, gender, employment and baseline CD4 count, community-based club participants had an increased risk of loss from club (aHR 1.43, 95% CI:1.15–1.79, *p* = 0.001). Main reasons for return to clinic-based care were missing ART pickup (59%, *n* = 198) or pregnancy (11%, *n* = 36), and were similar by arm. Among those referred to standard care, 63% and 80% made a visit within 60 and 90 days, respectively, of their last club visit.

**Conclusions**: By two years, drop-out from adherence club participation was high (43%) and higher among community-based compared to clinic-based clubs.

## MOAX0201LB

### A randomized controlled trial for the treatment of HIV-associated cryptococcal meningitis in Africa: oral fluconazole plus flucytosine or one week amphotericin-based therapy versus two weeks amphotericin-based therapy. The ACTA Trial

S Molloy ^1^; C Kanyama^2^; R Heyderman^3,4,5^; A Loyse^1^; C Kouanfack^6^; D Chanda^7^; S Mfinanga^8^; E Temfack^9,10^; S Lakhi^11^; S Lesikari^8^; A Chan^12^; N Stone^1,7^; N Kalata^4,5^; N Karunaharan^1,7^; K Gaskell^4,5^; M Peirse^4,5^; J Ellis^4,5^; C Chawinga^2^; S Lontsi^6^; J-G Ndong^6^; P Bright^7,12^; D Lupiya^12^; T Chen^13^; J Bradley^14^; J Adams^1^; C van der Horst^2,15^; JJ van Oosterhout^12^; V Sini^6^; YN Mapoure^9^; P Mwaba^7^; T Bicanic^1^; D Lalloo^13^; D Wang^13^; M Hosseinipour^2,15^; O Lortholary^10,16^; S Jaffar^13^; T Harrison^1^; ACTA Trial Study Team

^1^St George’s University of London, Centre for Global Health, Institute for Infection and Immunity, London, UK. ^2^UNC Project, Kamuzu Central Hospital, Lilongwe, Malawi. ^3^University College London, London, UK. ^4^Malawi-Liverpool-Wellcome Trust Clinical Research Programme, Blantyre, Malawi. ^5^College of Medicine, Queen Elizabeth Hospital, Blantyre, Malawi. ^6^Hopital Central Yaounde/Site ANRS Cameroun, Yaounde, Cameroon. ^7^Institute for Medical Research and Training, University Teaching Hospital, Lusaka, Zambia. ^8^National Institute Medical Research, Muhimbili Medical Research Centre, Dar Es Salaam, Tanzania, United Republic of. ^9^Douala General Hospital, Douala, Cameroon. ^10^Paris Descartes University/Institut Pasteur, Paris, France. ^11^University Teaching Hospital, Lusaka, Zambia. ^12^Dignitas International, Zomba Hospital, Zomba, Malawi. ^13^Liverpool School of Tropical Medicine, Liverpool, UK. ^14^London School of Hygiene and Tropical Medicine, London, UK. ^15^University of North Carolina, Chapel Hill, USA. ^16^Necker Pasteur Center for Infectious Diseases and Tropical Medicine, Paris, France

Presenting author email: smolloy@sgul.ac.uk

**Background**: Cryptococcal meningitis (CM) accounts for 10–20% of HIV-related deaths and >100,000 deaths/year. Amphotericin (AmB) plus flucytosine for 2 weeks is considered the gold standard but is unavailable in resource-limited settings where fluconazole treatment predominates.

**Methods**: Based on Phase II studies, we tested, against 2 weeks AmB-based treatment, 2 new strategies, which could be sustainable in Africa, and more effective than fluconazole: optimized oral therapy of high dose fluconazole plus flucytosine, and short (1 week) induction with AmB-based treatment. In the AmB arms, we compared fluconazole and flucytosine as adjunctive treatments.

Between 2013 and 2016, 721 participants from 9 centres in Malawi, Zambia, Cameroon and Tanzania with first-episode CM were randomized to:

**Oral** (238): fluconazole (1200mg/day) plus flucytosine (100mg/kg/day) for 2 weeks.

**1-week** (240): AmB (1mg/kg/d), plus fluconazole (1200mg/day), or flucytosine (100mg/kg/day) (ratio 1:1), for 7 days. Days 8–14, fluconazole 1200mg/day.

**2-weeks** (243): AmB (1mg/kg/d) plus fluconazole (1200mg/day), or flucytosine (100mg/kg/day) (ratio 1:1), for 14 days.

After 2 weeks, all received standard fluconazole consolidation. ART was started, or restarted, at 4 weeks, and patients followed-up to 10 weeks.

**Results**: Only 4 participants were lost-to-follow-up. Mortality at 2 and 10 weeks for oral, 1-week and 2-weeks was 18%, 22%, 21%, and 35%, 36%, 40%, respectively. The upper 1-sided 95% CI limits for the difference in mortality comparing oral and 1-week against 2 weeks AmB-based treatment (primary endpoint) were 3.0% and 6.8%, below the pre-specified 10% non-inferiority margin. Hazard ratios (95% CI) were 0.82 (0.54–1.25) and 1.01 (0.68–1.51) at 2, and 0.83 (0.61–1.13) and 0.89 (0.66–1.21) at 10 weeks, for oral and 1-week versus 2-weeks, respectively. As adjunctive treatment with AmB, flucytosine was superior to fluconazole (HR(95% CI): 1.62(1.19–2.20) *p* = 0.002). One week AmB plus flucytosine had the lowest 10-week mortality (24%), significantly lower than all other AmB arms (HR(95%CI): 0.56(0.35–0.91) comparing 1-week with 2-weeks AmB plus flucytosine). Side effects were more frequent with 2 weeks AmB than with 1 week AmB, or oral therapy.

**Conclusions**: One week AmB plus flucytosine and the oral combination provide safe, effective and sustainable induction therapy in resource-limited settings. Flucytosine should be made widely available for treatment of cryptococcosis.

## MOAX0202LB

### Dolutegravir/tenofovir/emtricitabine (DTG/TDF/FTC) started in pregnancy is as safe as efavirenz/tenofovir/emtricitabine (EFV/TDF/FTC) in nationwide birth outcomes surveillance in Botswana

R Zash ^1,2,3^; D Jacobson^4^; G Mayondi^3^; M Diseko^3^; J Makhema^3^; M Mmalane^3^; T Gaolathe^3^; C Petlo^5^; L Holmes^6^; M Essex^2,3^; S Lockman^2,3,7^ and R Shapiro^2,3^

^1^Beth Israel Deaconess Medical Center, Division of Infectious Diseases, Boston, USA. ^2^Harvard TH Chan School of Public Health, Boston, USA. ^3^Botswana Harvard AIDS Institute Partnership, Gaborone, Botswana. ^4^Harvard TH Chan School of Public Health, Center for Biostatistics in AIDS Research, Boston, USA. ^5^Ministry of Health, Gaborone, Botswana. ^6^Massachusetts General Hospital for Children, Boston, USA. ^7^Brigham and Women’s Hospital, Boston, USA

Presenting author email: rzash@bidmc.harvard.edu

**Background**: Global rollout of DTG-based antiretroviral therapy (ART) has been hampered by lack of safety data in pregnancy.

**Methods**: We captured birth outcome data at 8 government hospitals throughout Botswana (~45% of all deliveries) starting August 2014. In 2016, Botswana changed first-line ART from EFV/TDF/FTC to DTG/TDF/FTC, including for pregnant women. This analysis included women initiating either EFV/TDF/FTC (delivered August 2014–August 2016) or DTG/TDF/FTC (delivered November 2016–April 2017) during singleton pregnancy. Outcomes included combined endpoints of any adverse outcome (stillbirth, preterm birth (<37 weeks), small for gestational age (SGA) (<10th% weight-for-gestational age), or neonatal death (<28 days)) and severe adverse outcomes (stillbirth, neonatal death, very preterm birth (<32 weeks) and very SGA (<3rd% weight-for-gestational age)). We fit log-binomial regression models, controlling for maternal age, gravidity and education, to estimate adjusted risk ratios (aRRs). Congenital abnormalities were detected by maternity nurse surface exam.

**Results**: Maternal characteristics were similar for women starting DTG/TDF/FTC (*N* = 845) or EFV/TDF/FTC (*N* = 4593), including age, education, occupation, parity, alcohol/tobacco use, history of adverse birth outcome, delivery site, gestational age at presentation for antenatal care and CD4 cell count. ART was initiated at median (IQR) 19 (15, 25) weeks gestation for DTG/TDF/FTC and 21 (16, 27) for EFV/TDF/FTC. There were no significant differences in stillbirth, neonatal death, preterm or very preterm birth, SGA or very SGA by regimen (Table 1). Comparisons of any adverse birth outcome (aRR 1.0, 95% CI 0.9,1.1) and severe outcomes (aRR 1.0, 95% CI 0.8,1.2) were similar by regimen. Among 512 first-trimester ART initiations (116 DTG/TDF/FTC, 396 EFV/TDF/FTC), one major congenital abnormality was identified (skeletal dysplasia in an EFV-exposed infant).

**Conclusions**: Adverse birth outcomes were similar for DTG-based ART and EFV-based ART when started during pregnancy. Further studies are needed to determine the safety of DTG exposure from conception.

**Abstract MOAX0202LB–Table 1.** Birth outcomes by regimen started in pregnancy.

	Tenofovir/emtricitabine/ DOLUTEGRAVIR (DTG/TDF/FTC) *N* = 845	Tenofovir/emtricitabine/ EFAVIRENZ (EFV/TDF/FTC) *N* = 4593	Adjusted relative risk (95% CI) DTG/TDF/FTC vs. EFV/TDF/FTC
Any adverse outcome	291 (34.4%)	1606 (35.0%)	1.0 (0.9,1.1)
Severe adverse outcome	92 (10.9%)	519 (11.3%)	1.0 (0.8,1.2)
Stillbirth	18 (2.1%)	105 (2.3%)	0.9 (0.6,1.5)
Neonatal death (<28 days)	11 (1.3%)	60 (1.3%)	1.0 (0.5,1.9)
Preterm birth (<37 weeks)	149 (17.8%)	844 (18.5%)	1.0 (0.8,1.1)
Very preterm birth (<32 weeks)	35 (4.2%)	160 (3.5%)	1.2 (0.8,1.7)
Small for gestational age (<10th %tile weight for gestational age)	156 (18.7%)	838 (18.5%)	1.0 (0.9,1.2)
Very small for gestational age (<3rd %tile weight for gestational age)	51 (6.1%)	302 (6.7%)	0.9 (0.7,1.2)

## MOAX0203LB

### Weekly oral MK-8591 protects male rhesus macaques against repeated low-dose intrarectal challenge with SHIVC109P3

M Markowitz ^1^; A Gettie^1^; L St Bernard^1^; H Mohri^1^; CD Andrews^1^; A Horowitz^1^; J Blanchard^2^; B Grasperge^2^; L Sun^3^; KL Filigrove^3^; DJ Hazuda^3^ and JA Grobler^3^

^1^Aaron Diamond AIDS Research Center, Rockefeller University, New York, USA. ^2^Tulane National Primate Research Center, Covington, USA. ^3^Merck Sharp and Dhome, Kenilworth, USA

Presenting author email: mmarkowitz@adarc.org

**Background**: MK-8591 (4’-Ethynyl-2-Fluoro-2’-Deoxyadenosine; EFdA) is a potent and long-acting nucleoside RT translocation inhibitor (NRTTI) in clinical development for the treatment of HIV-1 infection. Single 10 mg doses of MK-8591 resulted in suppression of viremia in HIV-1-infected patients for at least 10 days as have weekly doses of 3.9 mg/kg or greater in SIV-infected rhesus macaques (RM).

**Methods**: Two groups of 8 male RM were given 5 mL/kg of 10% Tween80 with (treated) or without (placebo) 3.9 mg/kg MK-8591 by oral gavage on day 0, day 7 and weekly thereafter for a maximum of 14 doses or until SHIV infection was confirmed. All animals were challenged intrarectally (IR) with 1 ml of 50 TCID_50_ of SHIVC109P3, a viral stock derived from the third passage in RM of the molecular clone SHIVC109F.PB4, which contains an HIV Env initially derived from a newly HIV-infected Zambian. Challenges occurred on day 6 and weekly thereafter for a maximum of 12 challenges or until infection was confirmed. Prior to weekly challenge, blood was drawn to determine infection status and drug levels. Infection was confirmed by real-time RT PCR amplification of viral *gag* sequences in plasma on two consecutive samples. Proviral DNA was measured by PCR and virus-specific antibody responses were assessed. Intracellular levels of MK-8591-triphosphate (TP) were measured by LC/MS/MS.

**Results**: All placebo animals became infected after 1–4 challenges (median 1, mean 2). All treated animals remained uninfected after 12 challenges and were followed through Week 24 without evidence of infection as determined by the absence of plasma viremia, proviral DNA and seroconversion. MK-8591-treated macaques had a 41.5-fold lower risk of infection (95% C.I 7.3, 237.9) compared with placebo macaques (*p* < 0.0001, log-rank test). Mean trough concentrations of the active MK-8591-TP at the time of challenge were 4.07mM (range: 2.26–5.17) and compare favourably with the level achieved by a weekly oral dose of 10mg in HIV-1-infected humans.

**Conclusions**: MK-8591 is a potent NRTTI that completely protected against repeated low-dose IR challenge in this SHIVC109P3/RM model with intracellular active drug concentrations readily achieved in humans. These results support the potential use of MK-8591 for HIV prophylaxis.

## MOAX0204LB

### Substantial progress in confronting the HIV epidemic in Swaziland: first evidence of national impact

R Nkambule ^1^, H Nuwagaba-Biribonwoha^2^, Z Mnisi^1^, TT Ao^3^, C Ginindza^4^, YT Duong^5^, H Patel^6^, S Saito^5^, NM Philip^5^, K Brown^6^, C Draghi^7^, AC Voetsch^6^, K Mabuza^8^, A Zwane^4^, R Sahabo^2^, V Okello^1^, T Dobbs^6^, B Parekh^6^, C Ryan^3^, J Justman^5^

^1^Ministry of Health – Swaziland, Mbabane, Swaziland, ^2^ICAP at the Columbia University, Mailman School of Public Health, Mbabane, Swaziland, ^3^U.S. Centers for Disease Control and Prevention/U.S. President’s Emergency Fund for AIDS Relief (PEPFAR), Mbabane, Swaziland, ^4^Central Statistics Office, Mbabane, Swaziland, ^5^ICAP at the Columbia University, Mailman School of Public Health, New York, United States, ^6^U.S. Centers for Disease Control and Prevention, Center for Global Health, Division of Global HIV/AIDS, Atlanta, United States, ^7^Association of Schools and Programs of Public Health, Washington, United States, ^8^National Emergency Response Council on HIV/AIDS (NERCHA), Mbabane, Swaziland

Presenting author email: nkambulere@gov.sz

**Background**: Swaziland has the highest national HIV incidence and prevalence in the world. In response, the Swazi government extensively scaled up national HIV prevention and treatment services. The 2016–2017 Population HIV Impact Assessment (PHIA) Swaziland HIV Incidence Measurement Survey (SHIMS2) provides the first measure of the impact of national HIV programmes’ scale up on the epidemic’s trajectory since the previous SHIMS1 survey conducted in 2011.

**Methods**: A nationally representative sample of individual ≥15 years (y) underwent household-based, rapid HIV testing from August 2016 to March 2017. All HIV-positive samples were tested for HIV RNA and limiting antigen (LAg) Avidity. WHO criteria for HIV incidence estimates were used (LAg ≤ 1.5 ODn and HIV RNA >1000 copies/ml). Viral load (VL) suppression (VLS) was defined as <1000 copies/ml. Weighted measures of national HIV incidence, HIV prevalence and population VL (among all HIV positive, regardless of HIV knowledge or ART use) were compared with SHIMS1 results among adults 18–49 year.

**Abstract MOAX0204LB–Table 1.** HIV incidence, prevalence and viral load suppression among adult 18–49 years in SHIMS2, 2016–17 and SHIMS1, 2011.

**Results**: A total of 10,934 participants ≥15 years were tested, 3003 tested HIV positive, with HIV prevalence (95% Confidence Interval) of 27.0% [25.7, 28.3], and HIV incidence of 1.36% [0.92, 1.81]. Among adults, 18–49 years incidence was 1.39% [0.83, 1.94], a 44% decrease from the 2011 incidence estimate of 2.48% [1.96, 3.00]. Adult HIV incidence was higher among women (1.95%) [1.04, 2.84] than men (0.86%) [0.23, 1.48], with 38% and 53% decreases in women and men, respectively, from 2011. VLS among all HIV-positive participants was 73.1% [71.3, 75.0]. Among HIV-positive adults of 18–49 years, VLS was 71.3% [69.0, 73.5], a twofold increase from the 2011 VLS of 34.8% [33.4, 36.2].

**Conclusions**: Since 2011, VLS prevalence in Swaziland has doubled and national HIV incidence has decreased by nearly half. These remarkable findings in a high prevalence setting provide the first direct measure of the national impact of expanded HIV prevention and treatment programmes. Sustaining these achievements will be paramount to Swaziland’s success in curbing its severe HIV epidemic.

## MOAX0205LB

### Safety and efficacy of long-acting CAB and RPV as two drug IM maintenance therapy: LATTE-2 week 96 results

J Eron ^1^; D Margolis^2^; J Gonzalez-Garcia^3^; H-J Stellbrink^4^; Y Yazdanpanah^5^; D Podzamczer^6^; T Lutz^7^; JB Angel^8^; GJ Richmond^9^; B Clotet^10^; F Gutierrez^11^; L Sloan^12^; KC Sutton^2^; D Dorey^13^; KY Smith^2^; PE Williams^14^ and WR Spreen^2^

^1^University of North Carolina at Chapel Hill, Division of Infectious Diseases, Chapel Hill, USA. ^2^ViiV Healthcare, Research Triangle Park, USA. ^3^Hospital Universitario La Paz/IdiPAZ, Madrid, Spain. ^4^ICH-Hamburg, Hamburg, Germany. ^5^Hôpital Bichat Claude Bernard, Paris, France. ^6^Ciudad Sanitaria y Universitaria de Bellvitge, Barcelona, Spain. ^7^Infektiologikum, Frankfurt, Germany. ^8^The Ottawa Hospital, Ottawa, Canada. ^9^Broward General Medical Center, Fort Lauderdale, USA. ^10^Hospital Germans Trias i Pujol, UAB, UVIC-UCC, Badalona, Spain. ^11^Hospital General de Elche & Universidad Miguel Hernández, Alicante, Spain. ^12^North Texas Infectious Disease Consultants, Dallas, USA. ^13^GlaxoSmithKline, Missisauga, Canada. ^14^Janssen Research and Development, Beerse, Belgium

Presenting author email: joseph_eron@med.unc.edu

**Background**: Long-acting (LA) injectable nanosuspensions of cabotegravir (CAB) and rilpivirine (RPV) are being developed. At the LATTE-2 W32 primary endpoint, response rates were statistically comparable between injectable every 4 weeks (Q4W), injectable every 8 weeks (Q8W) LA arms and daily oral CAB 30 mg + ABC/3TC (PO) dosing.

**Methods**: Phase 2b, multicentre, parallel group, open-label study in ART-naive HIV infected adults. Patients with plasma HIV-1 RNA <50 c/mL during the 20-week induction period on once daily oral CAB + ABC/3TC were randomized 2:2:1 to IM CAB LA + RPV LA Q4W, Q8W or PO in the Maintenance Period (MP). Evaluations included; antiviral activity <50 c/mL (FDA snapshot analysis), protocol-defined virologic failure (PDVF)and safety at the pre-specified W96 secondary endpoint in MP (ITT-maintenance exposed (ME)).

**Results**: 309 patients were enrolled (ITT-exposed): 91% male, 20% non-white and 19% >100,000 c/mL HIV-1 RNA. 286 patients were randomized into the MP. At W96, 94% (Q8W), 87% (Q4W) and 84% (PO) remained suppressed (ITT-ME). Three ME patients had PDVF through W96; two Q8W (one at W4 and one at W48 with NNRTI/INI mutations) and one PO at W8. SAEs occurred in 10% (Q8W), 10% (Q4W) and 13% (PO) patients, and none were drug related. Excluding injection site reactions (ISRs), 2% (Q8W), 4% (Q4W) and 2% (PO) reported drug-related AEs ≥Grade 3. Only two patients had ISRs leading to discontinuation through W96. Majority of ISRs were mild/moderate pain and discomfort with <1% of ISRs was classified as severe. Emergent lab abnormalities ≥Grade 3 occurred in 19% (Q8W), 29% (Q4W) and 21% (PO).

**Conclusions**: LA injectable 2-drug therapy given either Q8W or Q4W IM demonstrated high rates of virologic response and was well tolerated through 96 weeks. Difference in virologic success between Q8W and Q4W is primarily due to non-virologic reasons. Phase 3 studies are evaluating Q4W dosing.

**Abstract:** MOAX0205LB–Table 1.

## MOAX0105LB

### HIV self-testing among female sex workers in Zambia: a randomized controlled trial

M Chanda^1^; K Ortblad^2^; M Mwale^1^; S Chongo^1^; C Kanchele^1^; N Kamungoma^1^; A Fullem^3^; C Dunn^3^; L Barresi^2^; G Harling^2^; T Bärnighausen^2,4,5^ and C Oldenburg ^6^

^1^John Snow, Inc, Lusaka, Zambia. ^2^Harvard T.H. Chan School of Public Health, Boston, USA. ^3^John Snow, Inc, Boston, USA. ^4^University of Heidelberg, Heidelberg, Germany. ^5^Africa Health Research Institute, Somkhele, South Africa. ^6^University of California, San Francisco, F.I. Proctor Foundation, San Francisco, USA

Presenting author email: catherine.oldenburg@ucsf.edu

**Background**: HIV self-tests (HIVST) may help increase HIV testing coverage to meet the 90-90-90 target in female sex worker (FSW) populations. We report the results of a randomized controlled trial of HIVST among FSW in Zambia.

**Methods**: Trained peer educators in Kapiri, Chirundu and Livingstone, Zambia each recruited 6 FSW participants. Peer educator-FSW groups were randomized to: (1) *direct* distribution of an oral HIVST from the peer educator, (2) distribution of a *coupon* for an oral HIVST available from a health clinic/pharmacy or (3) referral to *standard* HIV testing. HIVST-arm participants received one HIVST at baseline and another three months later. Participants completed baseline, month-1 and month-4 questionnaires.

**Results**: 965 participants were enrolled between September and October 2016; 20% had never tested for HIV. 98.3% of direct distribution arm participants reported using their HIVST at month-1, compared to 86.3% in the coupon arm (*p *= 0.001); this difference had disappeared by month-4. There was no significant difference in reported past-month testing for HIV at month-1 or month-4, although rates were highest for the direct arm at both time points. At month-1, 94.9%, 84.4% and 88.5% of direct-, coupon- and standard-arm participants reported testing in the past month (*p *= 0.10 direct vs. standard, *p *= 0.29 coupon vs. standard). At month-4, past-month testing coverage was 84.1%, 79.8% and 75.1% (*p *= 0.11 direct vs. standard, *p *= 0.42 coupon vs. standard).

Of 144 participants reporting a positive HIV test at month-1, 51.0% and 52.8% in direct- and coupon-arm participants reported linking to care, compared to 74.6% in the standard arm (*p *= 0.07 direct, *p *= 0.12 coupon). At month-4, of 235 participants reporting a positive test, 71.6%, 76.6% and 85.7% of direct-, coupon- and standard-arm participants reported linking (*p *= 0.13 direct, *p *= 0.17 coupon). Three cases of HIVST-related intimate-partner violence (IPV) were reported, despite 60% of participants reporting IPV in the previous year.

**Conclusions**: HIVST provision via peer educators to Zambian FSW led to high test uptake and rapid linkage to care, including amongst those who had never previously tested, without a significant increase in IPV. HIVST should be considered as part of an intervention package to maximize HIV protection for FSW populations.

## MOAX0106LB

### Performance and usability of INSTI, a blood-based rapid HIV self-test for qualitative detection of HIV antibodies in intended-use populations in Kenya

M Mwau; L Achieng and P Bwana

Kenya Medical Research Institute, Nairobi, Kenya

Presenting author email: mmwau@kemri.org

**Background**: HIV self-testing is rapidly gaining acceptance as an effective method to reach undiagnosed individuals in sub-Saharan Africa; however, there is very little documented data on performance of blood-based self-tests in diverse intended-use populations. The self-testing concept was approved in Kenya in 2015, but no products were available.

**Methods**: The Kenya Medical Research Institute (KEMRI) completed a study of the blood-based 60-second INSTI HIV Self-Test to measure its performance, usability and readability in 688 consenting adults with broad demographic diversity, from Matayos, Bumutiru, Khunyangu, Aterait and Asinge villages in Busia County, Western Kenya. All subjects participated in the performance study, comparing INSTI results to fourth-generation EIA results from venous blood collected from each subject. Portions of the study subjects also participated in qualitative usability and readability studies to assess label comprehension, ease of use and result interpretation. The study was conducted between 22 March and 11 April 2017, under ethics approval by the KEMRI Ethical Review Committee.

**Results**: Compared to the bioelisa HIV-1+2 Ag/Ab (Biokit S.A., Barcelona, Spain) EIA test, the specificity of the INSTI HIV self-test was 99.26% and sensitivity was 98.51%. Negative predictive value was 98.89% and positive predictive value was 99.00% for the study population. From the 350 subjects in the usability study, 98.00% found the test instructions easy to follow; 99.71% successfully added the blood droplet into INSTI bottle 1; 97.71% indicated willingness to use the test again; and 98.29% would recommend the kit to a partner. For the 91 subjects in the readability study, 100% correctly interpreted the positive, negative and invalid results, while 65.93% were unsure how to interpret the weak positive result.

**Conclusions**: INSTI is unique for its use of a “hanging” fingerstick blood drop, without the need for a collection device. This first field study of such a fingerstick blood-based self-test provides strong evidence that the INSTI HIV Self-Test is accurate, acceptable and easy to use by self-testers with diverse backgrounds in sub-Saharan Africa. Modifications to the kit instructions to include a visual of a weak positive result would provide a more consistent interpretation.

## TUAA0106LB

### Efficacy of epithelial stem cell-based AIDS vaccine to induce mucosal immune responses offering protection against SIV challenge in macaques

M-CE Gauduin ^1^; R White^1^; J Garcia^1^; P Kozlowski^2^; P Blancou^3^ and P Frost^4^

^1^Texas Biomedical Research Institute, Virology and Immunology, San Antonio, USA. ^2^Louisiana State University Health Sciences Center, New Orleans, USA. ^3^University of Nice-Sophia Antipolis, Valbonne, France. ^4^Southwest National Research Center, San Antonio, USA

Presenting author email: mcgauduin@txbiomed.org

**Background**: A key obstacle limiting the development of an effective AIDS vaccine is the inability to deliver antigen for a sufficient period of time resulting in weak and transient protection. HIV transmission occurs predominantly across mucosal surfaces; therefore, an ideal vaccine strategy would be to target HIV at mucosal entry sites to prevent infection.

**Methods**: We developed a SIV single cycle vaccine under the control of the involucrin promoter (pINV-SIVsc), which was tested for its ability to drive SIV expression in terminally differentiated epithelial cells, induce mucosal immune response and offer better protection against SIV challenge. A total of 20 naive young Rhesus macaques were selected (10/20 expressed MHC class I Mamu-A*01 allele). The pINV-SIVsc vaccine was administrated intravaginal (*n* = 12) at Week 0. Animals were monitored overtime for specific immune responses in blood and various tissues (*n* = 4). Eight animals were challenged at Week 12 (*n* = 4) or at Week 24 (*n* = 4) using repeated pathogenic SIVmac239 and monitored for specific immune responses in blood and various tissues. Eight additional animals were infected with repeated SIVmac239, and served as unvaccinated Controls. Complementary approaches were used to characterize SIV-specific immune responses in blood, vaginal secretions, LN and vaginal biopsies collected at various times.

**Results**: This vaccine induced strong mucosal IgA and IgG responses and specific T cells expressing a4ß7 homing to the mucosa. Repeated challenges revealed significant delay and lower viremia with 3–4 logs-reduction at peak, 4–5 logs-reduction at set-point and undetectable viremia by Week 10–14 post-SIV in vaccinated females compared to Controls. Following challenge, we demonstrated a positive correlation between the generation of mucosal and systemic T cell responses and control of viremia, an inverse association between viremia and post-infection vaginal IgA/IgG responses.

**Conclusions**: We have obtained evidence, within the limitation of the small animals’ number studied, that macaques vaccinated with pINV-SIVsc can generate strong mucosal SIV-specific T cell responses and local antibody responses (IgA/IgG). We demonstrated the efficacy of an epithelial stem-cell-based SIV vaccine to serve as antigen delivery system suggesting an important role in protection against mucosal infection.

## TUAB0104LB

### Fixed dose combination of doravirine/lamivudine/TDF is non-inferior to efavirenz/emtricitabine/TDF in treatment-naive adults with HIV-1 infection: Week 48 results of the Phase 3 DRIVE-AHEAD study

KE Squires ^1^; J-M Molina^2^; PE Sax^3^; W-W Wong^4^; C Orkin^5^; O Sussmann^6^; D Tutu^7^; L Lupinacci^8^; A Rodgers^8^; X Xu^8^; G Lin^8^; S Kumar^8^; B-Y Nguyen^8^; GJ Hanna^8^; C Hwang^8^; E Martin^8^; for the DRIVE-AHEAD Study Group

^1^Thomas Jefferson University Hospital, Philadelphia, USA. ^2^University of Paris Diderot, Hôpital Saint-Louis, Paris, France. ^3^Brigham and Women’s Hospital, Harvard Medical School, Boston, USA. ^4^Taipei Veterans General Hospital, Taipei, Japan. ^5^Royal London Hospital, London, UK. ^6^Asistencia Cientifica de Alta Complejidad SAS, Bogota, Colombia. ^7^Desmond Tutu HIV Foundation, Cape Town, South Africa. ^8^Merck & Co., Inc., Kenilworth, USA

Presenting author email: kathleen.squires@jefferson.edu

**Background**: Doravirine (DOR) is a novel non-nucleoside reverse transcriptase inhibitor (NNRTI) with once-daily (QD) dosing. In the Phase 3 DRIVE-FORWARD study in HIV-1 treatment-naive adults receiving 2 NRTIs, DOR 100mg QD was non-inferior to darunavir+ritonavir on efficacy, and demonstrated a more favourable lipid profile.

**Methods**: DRIVE-AHEAD compared doravirine with efavirenz (EFV) in an ongoing Phase 3, multicentre, double-blind, non-inferiority trial. Eligible participants were antiretroviral treatment-naive adults with HIV-1 infection and pre-treatment HIV-1 RNA ≥1000 c/mL. Participants were randomized (1:1) to a once-daily fixed-dose regimen of DOR 100mg, lamivudine 300mg and tenofovir disoproxil fumarate 300mg (DOR/3TC/TDF) or EFV 600mg, emtricitabine 200mg and TDF300 mg (EFV/FTC/TDF) for up to 96 weeks. Randomization was stratified by screening HIV-1 RNA (≤/>100,000 c/mL) and hepatitis B/C co-infection (yes/no). Primary efficacy endpoint was percentage of participants with HIV-1 RNA <50 c/mL at Week 48 (FDA Snapshot approach). Predefined non-inferiority margin was 10%. Primary safety endpoint was percentage of participants with pre-specified neuropsychiatric adverse events (dizziness, sleep disorders/disturbances, altered sensorium).

**Results**: Of 734 participants randomized, 728 received study drug (364 in each treatment group) and were included in the analyses (mean age 33 years, 85% male, 48% white). At Week 48, HIV-1 RNA <50 c/mL was achieved by 84.3% (307/364) of DOR/3TC/TDF recipients and 80.8% (294/364) of EFV/FTC/TDF recipients (difference 3.5%, 95%CI (−2.0, 9.0)). The incidence of dizziness, sleep disorders/disturbances and altered sensorium (Table 1) was lower in DOR/3TC/TDF recipients than in EFV/FTC/TDF recipients (*p* < 0.001, *p* < 0.001 and *p* = 0.033, respectively). Fasting LDL-C and non-HDL-C (Table 1) were reduced by DOR/3TC/TDF and increased by EFV/FTC/TDF (both *p* < 0.0001).

**Conclusions**: In HIV-1 treatment-naive adults, the efficacy of DOR/3TC/TDF at Week 48 was non-inferior to EFV/FTC/TDF and similar regardless of baseline HIV-1 RNA. DOR/3TC/TDF was generally safe and well tolerated, with significantly fewer neuropsychiatric events than EFV/FTC/TDF and a favourable lipid profile.

**Abstract TUAB0104LB–Table 1.** Week 48 efficacy and safety outcomes.

## TUAB0105LB

### Superior efficacy of dolutegravir (DTG) plus 2 nucleoside reverse transcriptase inhibitors (NRTIs) compared with lopinavir/ritonavir (LPV/RTV) plus 2 NRTIs in second-line treatment: interim data from the DAWNING study

M Aboud ^1^; R Kaplan^2^; J Lombaard^3^; F Zhang^4^; J Hidalgo^5^; E Mamedova^6^; M Losso^7^; P Chetchotisakd^8^; J Sievers^1^; D Brown^9^; J Hopking^10^; M Underwood^11^; MC Nascimento^1^; M Gartland^11^ and K Smith^11^

^1^ViiV Healthcare, Brentford, UK. ^2^Desmond Tutu HIV Foundation, Cape Town, South Africa. ^3^Josha Research, Bloemfontein, South Africa. ^4^Beijing Ditan Hospital, Beijing, China. ^5^VÍA LIBRE, Lima, Peru. ^6^Kiev AIDS Centre, Kiev, Ukraine. ^7^Hospital J M Ramos Mejía, Buenos Aires, Argentina. ^8^Srinagarind Hospital, Khon Kaen University, Khon Kaen, Thailand. ^9^ViiV Healthcare, Abbotsford, Australia. ^10^GlaxoSmithKline, Stockley Park, UK. ^11^ViiV Healthcare, Research Triangle Park, USA

**Background**: DAWNING is a non-inferiority study conducted to compare a protease inhibitor-sparing regimen of DTG+2NRTIs with a current WHO-recommended regimen of LPV/RTV+2NRTIs in HIV-1 infected subjects failing first-line therapy of a non-nucleoside reverse transcriptase inhibitor (NNRTI) + 2 NRTIs (ClinicalTrials.gov: NCT02227238). An Independent Data Monitoring Committee (IDMC) performed periodic reviews of data to protect the ethical and safety interests of subjects.

**Methods**: Adult subjects failing first-line therapy, with HIV-1 RNA ≥400 copies(c)/mL, were randomized (1:1, stratified by Baseline plasma HIV-1 RNA and number of fully active background NRTIs) to 52 weeks of open-label treatment with DTG or LPV/RTV combined with an investigator-selected dual NRTI background, including at least one fully active NRTI. An IDMC review was performed, which included data from 98% (612/627 randomized) of subjects through 24 weeks on therapy.

**Results**: At Week 24, 78% of subjects on DTG versus 69% on LPV/RTV achieved HIV-1 RNA <50 c/mL (adjusted difference 9.6%, 95% CI: 2.7% to 16.4%, *p* = 0.006 for superiority). The difference was primarily driven by lower rates of Snapshot virologic non-response in the DTG group. The safety profile of DTG+2NRTIs was favourable compared to LPV/RTV+2NRTIs with more drug-related adverse events (AEs) reported in the LPV/RTV group, mainly due to higher rates of gastrointestinal disorders.

Following review of Week 24 data and large subsets of data from Weeks 36 and 48, the IDMC recommended discontinuation of the LPV/RTV arm due to persistent differences in rates of Snapshot virologic non-response and protocol-defined virologic failure (PDVF) favouring the DTG arm.

**Abstract TUAB0105LB–Table 1.** Week 24 outcomes.

Week 24 outcomes	DTG (*N* = 307)	LPV/RTV (*N* = 305)
Snapshot virologic success	240 (78%)	210 (69%)
Snapshot virologic non-response	36 (12%)	64 (21%)
Data in window not <50 c/mL	33 (11%)	59 (19%)
Discontinued for other reason while not <50 c/mL or change in ART	3(1%)	5 (2%)
Snapshot no virologic data	31 (10%)	31 (10%)
Discontinued due to AE or death	5 (2%)	14 (5%)
Discontinued for other reason or missing data during window but on study	26 (8%)	17 (6%)
PDVF	5/312 (2%)	12/312 (4%)
Drug-related AEs	45/314 (14%)	107/310 (35%)

**Conclusions**: The IDMC recommended discontinuation of the LPV/RTV arm due to superior efficacy of DTG+2NRTIs and the potential to harm subjects on LPV/RTV based on available data. Final Week 24 results of this study will be presented. DAWNING provides important information to help guide second-line treatment decisions in resource-limited settings.

## TUAB0106LB

### HIV-specific broadly neutralizing monoclonal antibody, VRC01, minimally impacts time to viral rebound following treatment interruption in virologically suppressed, HIV-infected participants who initiated antiretroviral therapy during acute HIV infection

TA Crowell ^1,2^; DJ Colby^3^; S Pinyakorn^1,2^; J Intasan^3^; K Benjapornpong^3^; K Tanjnareel^3^; N Chomont^4^; L Trautmann^1,2^; S Tovanabutra^1,2^; S Krebs^1,2^; D Bolton^1,2^; A McDermott^5^; R Bailer^5^; N Doria-Rose^5^; B Patel^6^; RJ Gorelick^7^; BA Fullmer^7^; P Visudhiphan^8^; RJ O’Connell^1,8^; R Tressler^6,9^; J Mascola^5^; NL Michael^1^; ML Robb^1,2^; N Phanuphak^3^; J Ananworanich^1,2,3^; RV397 and RV254/SEARCH010 Study Groups

^1^U.S. Military HIV Research Program, Walter Reed Army Institute of Research, Silver Spring, USA. ^2^Henry M. Jackson Foundation for the Advancement of Military Medicine, Bethesda, USA. ^3^SEARCH, The Thai Red Cross AIDS Research Centre, Bangkok, Thailand. ^4^Centre de Recherche du CHUM and Department of Microbiology, Infectiology and Immunology, Université de Montréal, Quebec, Canada. ^5^Vaccine Research Center, National Institute of Allergy and Infectious Diseases, National Institutes of Health, Bethesda, USA. ^6^Division of AIDS, National Institute of Allergy and Infectious Diseases, National Institutes of Health, Bethesda, USA. ^7^AIDS and Cancer Virus Program, Leidos Biomedical Research, Inc., Frederick National Laboratory for Cancer Research, Frederick, USA. ^8^Armed Forces Research Institute of Medical Sciences, Bangkok, Thailand. ^9^Columbus Technologies & Services, Inc., El Segundo, USA

Presenting author email: tcrowell@hivresearch.org

**Background**: We present interim, blinded data exploring kinetics of viral load (VL) rebound in a randomized, placebo-controlled trial of VRC01 following analytic treatment interruption (ATI) in adults who initiated antiretroviral therapy (ART) during acute HIV infection (AHI). Study arms will be unblinded in June 2017.

**Methods**: Virologically suppressed adults who initiated ART during AHI underwent ATI and randomization (3:1) to receive VRC01 40mg/kg or placebo intravenously every three weeks for up to 24 weeks. If virologically suppressed at 24 weeks, observation continued of all therapies. Participants were monitored every 3–7 days for VL rebound and resumed ART for confirmed VL >1000 copies/ml or CD4 <350 cells/mm^3^.

**Results**: Twenty-three Thai males were enrolled. Four received no study product and one experienced Grade II generalized urticaria during the first infusion, terminating study participation without ATI. These analyses include 18 participants who initiated ART during Fiebig I (*n* = 1), II (*n* = 10) or III (*n* = 7); underwent randomization and ATI; and met a study endpoint (Table 1 and Figure 1). As of 9 May 9 2017, one participant remained off ART with an undetectable VL for 32 weeks. All other participants experienced VL rebound, restarted ART and re-achieved virologic suppression. Ten participants had detectable VL via single copy assay (range 0.44–2.1 copies/mL) at median 10 (range 7–29) days prior to rebound >20 copies/mL. There were no serious adverse events.

**Abstract TUAB0106LB–Table 1.** Participant characteristics and key results.

Baseline participant characteristics (*n* = 18)	Median	Range
Age (years)	28	21–50
Duration of ART (years)	3.0	2.3–6.6
CD4 Count (cells/mm^3^)	716	402–1032
Key results (*n* = 17; excluding one participant without VL rebound)		
Time to rebound viral load >20 copies/mL (days from ATI)	21	9–65
First detectable viral load (copies/mL)	447	21–7395
Highest viral load (copies/mL)	3845	1401–31,807
Time to viral load <20 copies/mL (days from ART resumption)	21	6–34

**Abstract TUAB0106LB–Figure 1.**
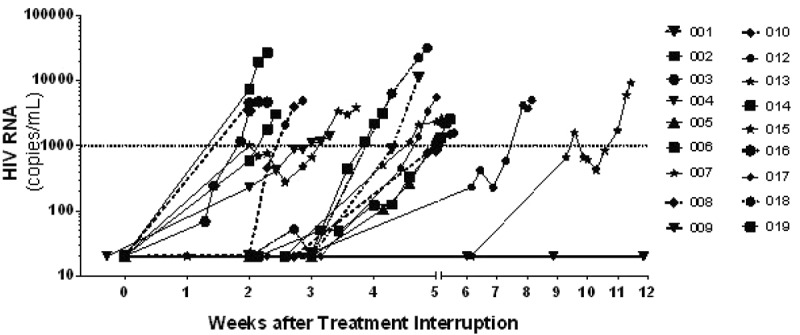
Clinical viral load assessments.

**Conclusions**: Participants who initiated ART during AHI and received VRC01 or placebo during ATI mostly experienced rapid VL rebound. Early ART alone or with VRC01 during ATI appears insufficient to delay time to VL rebound in most individuals.

## TUAC0406LB

### Increasing knowledge of HIV status among men: a cluster-randomized trial of community-based distribution of oral HIV self-test kits nested in four HPTN 071 communities in Zambia

H Ayles ^1,2^; S Floyd^3^; C Mulubwa^2^; B Hensen^1^; A Schaap^2,3^; M Phiri^2^; B Chiti^2^; K Shanaube^2^; M Simwinga^2^; V Bond^2,4^; S Fidler^5^; R Hayes^3^; A Mwinga^2^; on behalf of the HPTN 071 study team

^1^Department of Clinical Research, London School of Hygiene & Tropical Medicine, London, UK. ^2^Zambart, Lusaka, Zambia. ^3^Department of Infectious Disease Epidemiology, London School of Hygiene & Tropical Medicine, London, UK. ^4^Department of Global Health and Development, London School of Hygiene & Tropical Medicine, London, UK. ^5^Imperial College London, HIV Clinical Trials Unit, London, UK

Presenting author email: helen@zambart.org.zm

**Background**: HPTN071 (PopART) is a community-randomized trial evaluating the impact of a combination HIV-prevention intervention on HIV incidence. Overall, this intervention has reached the first UNAIDS 90, yet men and younger adults still have lesser knowledge of HIV status. We nested a cluster-randomized trial of oral HIV self-testing (HIVST) in addition to rapid finger-prick HIV testing (HIVFP) offered door to door by lay counsellors (CHiPs) within HPTN071 to evaluate the impact on knowledge of HIV status.

**Methods**: Four of the Zambian HPTN071 intervention communities were randomized by CHiP zones, with an average of 471 households per zone. In HIVST zones (*n* = 33/*N* = 66), individuals aged >16 years who did not self-report being HIV-positive, were offered a choice of HIVST or HIVFP. Secondary distribution of HIVST was offered for absent partners. A population-average logistic regression model was used to estimate the effect of the HIVST intervention on knowledge of HIV status (definition: self-report HIV-positive or accepted HIV-testing services), using total population enumerated as the denominator, adjusting for community, sex and age, and accounting for clustering by zone.

**Results**: Between 1 February and 30 April 2017, 63.3% (8139/12,852) of adults enumerated in the HIVST arm knew their HIV status compared to 61.3% (8203/13,383) in the non-HIVST arm, adjusted OR 1.25 (95% CI 1.01–1.56, *p* = 0.04). Women had high knowledge of HIV status (71.1% in both HIVST and non-HIVST, adjOR 1.03, 95% CI 0.85–1.25, *p* = 0.74). Among men, knowledge of HIV status was 55.0% in HIVST compared to 50.2% in non-HIVST (adjOR 1.30, 95% CI 1.07–1.60, *p* = 0.01), with strong evidence that the effect of the HIVST intervention was different for men and women (*p* = 0.004; Table 1).

**Abstract TUAC0406LB–Table 1.** Knowledge of HIV status in non-HIVST and HIVST zones.

Characteristic	Enumerated in non-HIVST zones	Knows HIV-status in non-HIVST zones	Percent (%)	Enumerated in HIVST zones	Knows HIV-status in HIVST zones	Percent (%)	Odds ratio	95% Confidence interval	*p*-Value
Total	13,383	8203	61.3	12,852	8139	63.3	1.25	1.01–1.56	0.04
Sex: male	6311	3171	50.2	6200	3412	55.0	1.30	1.07–1.60	0.01
Sex: female	7072	5031	71.1	6652	4727	71.1	1.03	0.85–1.25	0.74
Age (years): 16–29	6841	4541	66.4	6565	4508	68.7	1.24	1.01–1.53	0.04
Age (years): 30 and above	6542	3662	56.0	6287	3631	57.8	1.20	0.97–1.48	0.10
Community 1	3670	1971	53.7	3585	1933	53.9	1.01	0.79–1.29	0.93
Community 2	1567	715	45.6	1650	992	60.1	1.99	1.39–2.85	<0.001
Community 3	4150	2477	59.7	4604	2784	60.5	1.17	0.88–1.55	0.29
Community 4	3996	3040	76.1	3013	2430	80.7	1.45	0.68–3.10	0.34

**Conclusions**: Introducing HIVST for 3 months in communities already exposed to door-to-door HIV-testing services for 3 years increased the proportion of the population who knew their HIV status. This effect was seen most markedly in men.

## TUAC0106LB

### Safety, tolerability and pharmacokinetics of long-acting injectable cabotegravir in low-risk HIV-uninfected women and men: HPTN 077

R Landovitz ^1^; S Li^2^; B Grinsztejn^3^; H Dawood^4^; A Liu^5^; M Magnus^6^; M Hosseinipour^7^; R Panchia^8^; L Cottle^2^; G Chau^2^; P Richardson^9^; M Marzinke^9^; C Hendrix^9^; B Tolley^10^; A Adeyeye^11^; D Burns^11^; A Rinehart^12^; D Margolis^12^; M McCauley^13^ and J Eron^14^

^1^University of California, Los Angeles, Center for Clinical AIDS Research & Education, Los Angeles, USA. ^2^SCHARP, Seattle, USA. ^3^Instituto de Pesquisa Clinica Evandro Chagas-Fiocruz, Rio de Janeiro, Brazil. ^4^CAPRISA, University of Kwazulu Natal, Vulindlela, South Africa. ^5^San Francisco Department of Public Health, San Francisco, USA. ^6^George Washington University, Washington, USA. ^7^UNC-Malawi Project, Lilongwe, Malawi. ^8^Chris Hani Baragwanath Hospital, PHRU, Johannesburg, South Africa. ^9^Johns Hopkins University, Baltimore, USA. ^10^FHI360, Durham, USA. ^11^Division of AIDS/NIAID/NIH, Bethesda, USA. ^12^ViiV Healthcare, Durham, USA. ^13^FHI360, Washington, USA. ^14^University of North Carolina, Chapel Hill, USA

Presenting author email: rlandovitz@mednet.ucla.edu

**Background**: Cabotegravir (CAB) is a novel strand-transfer integrase inhibitor, available as a long-acting injectable nanosuspension, under development for HIV treatment and prevention.

**Methods**: HPTN 077 is a Phase 2a, randomized double-blind placebo-controlled study of CAB at two doses. Participants were low-risk HIV-uninfected individuals at eight sites globally, randomized (3:1) to daily oral CAB 30mg (or placebo (PBO)) for four weeks (W), followed by CAB (or PBO) 800mg IM at W5, 17, and 29 (Cohort 1(C1)) or 600mg IM at W5, 9, 17, 25, and 33 (Cohort 2(C2)).

**Results**: 110 participants enrolled in C1, 89 in C2. Median age 31 years (IQR: 24,40), BMI 27 (IQR: 23,32), 66% female, 41% Black, 27% White, 24% Latino, 8% mixed/other. Overall, 94% completed the oral phase, 89% received at least one injection and 75% completed all injections, which did not differ by arm, cohort or sex (Table 1). Over 41 W, injection site pain and injection site reactions (ISR) were more common in CAB versus PBO. No other differences were found in safety or tolerability. ISR led to injection discontinuation in 2/134 (1.5%).

**Abstract TUAC0106LB–Table 1.** Outcomes of study PPTs by cohort/sex.

One seizure (participant with previous seizures) and one seroconversion (48W post last injection) occurred in CAB participants; CAB levels at the time of events were 278 ng/mL and <25 ng/mL (LLQ), respectively. C2 dosing consistently achieved plasma trough targets; C1 dosing did not (Figure 1).

**Abstract TUAC0106LB–Figure 1.**
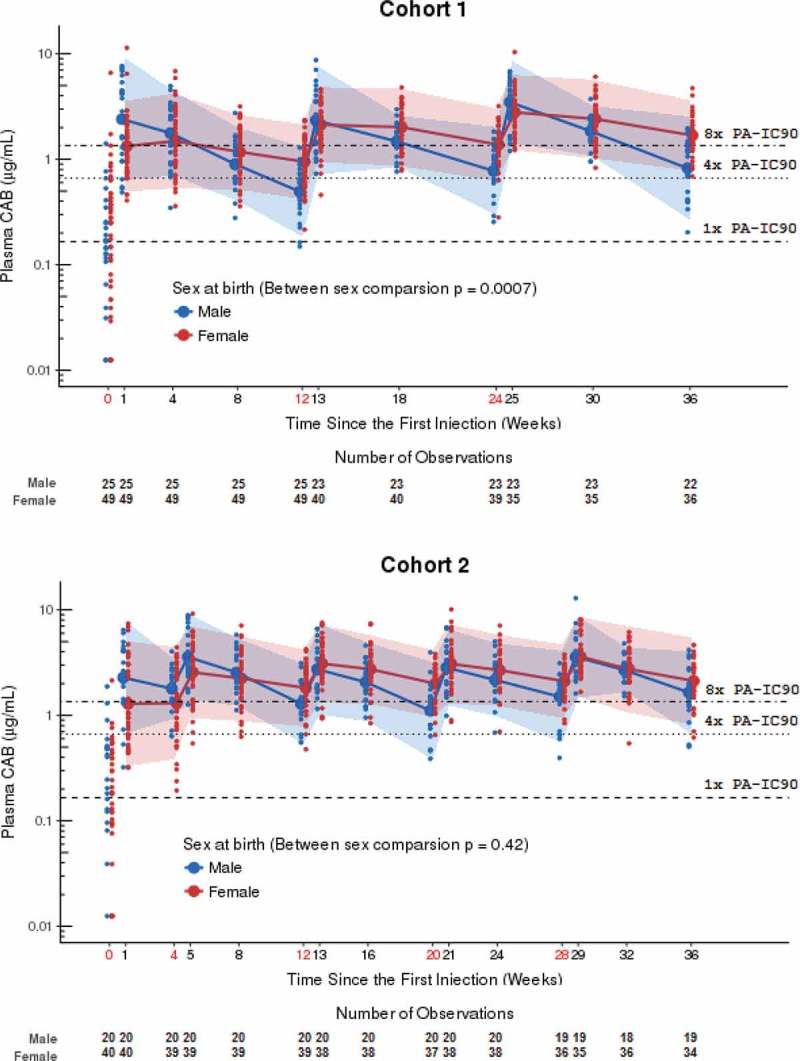
Geometric mean conc and 90% pred interval.

**Conclusions**: CAB was well tolerated among low-risk HIV-uninfected men and women. Pharmacokinetics support the development of CAB for HIV prevention using 600mg IM every 8 weeks with a 4-week loading dose for all sexes.

## TUAC0506LB

### HIV treatment prevents HIV transmission in male serodiscordant couples in Australia, Thailand and Brazil

B Bavinton^1^; B Grinsztejn^2^; N Phanuphak^3^; F Jin^1^; I Zablotska^1^; G Prestage^1^; A Kelleher^1^; A Pinto^1^; D Cooper^1^; A Grulich ^1^; The Opposites Attract Study Group

^1^University of New South Wales, Kirby Institute, Sydney, Australia. ^2^Instituto de Pesquisa Clínica Evandro Chagas, Rio de Janeiro, Brazil. ^3^Thai Red Cross, AIDS Research Centre, Bangkok, Thailand

Presenting author email: agrulich@kirby.unsw.edu.au

**Background**: Prospective data on the association of HIV transmission and undetectable viral load (UVL) in homosexual male HIV-serodiscordant couples (HM-SDC) are extremely limited. We report the final results from the Opposites Attract cohort study of HM-SDC in Australia, Bangkok and Rio de Janeiro.

**Methods**: HM-SDC were recruited through clinics. Information on sexual behaviours was collected at each visit from the HIV-negative partner (HNP). HNPs were tested at baseline and follow-up for HIV antibodies/sexually transmitted infections (STIs), and positive partners (HPPs) for HIV viral load/STIs. Phylogenetic analysis of *pol* and *env* genes was performed to identify linked HIV transmissions within couples based on genetic distance and monophyletic grouping. Incidence was calculated per couple-year of follow-up (CYFU), and stratified by pre-exposure prophylaxis (PrEP) use and by whether different forms of condomless anal intercourse (CLAI) were reported. UVL was defined as <200 copies/mL. One-sided upper 95% confidence limits (UCL) were calculated.

**Results**: By end 2016, 358 HM-SDC were enrolled: 157, 105 and 96 from Australia, Thailand and Brazil, respectively. There were 591 CYFU in 343 couples with at least one follow-up visit of whom 57.4% reported anal sex with outside partners during any point in follow-up. At baseline, 79.9% of HPPs were on anti-retroviral therapy (ART) and 77.9% had UVL; STI prevalence was 14.3%/11.7% in HPPs/HNPs, respectively. There were 318 CYFU in periods where CLAI was reported with a total of 16,889 acts of CLAI. There were 3 new HIV infections but no linked transmissions. The overall UCL of the transmission rate when CLAI was reported was 1.16/100 CYFU, and it was 1.56/100 CYFU when there was UVL.

**Abstract TUAC0506LB–Table 1.** HIV incidence by category of CLAI.

	Linked trans-missions (n)	Couple-years of follow up (CYFU)	CLAI acts (*n*)	Incidence per 100 CYFU (95% CI)
Overall	0	591.2	16,889	0 (0–0.62)
Any CLAI	0	318.0	16,889	0 (0–1.16)
Any CLAI, no daily PrEP	0	241.3	12,928	0 (0–1.53)
Insertive CLAI	0	210.0	8389	0 (0–1.76)
Receptive CLAI	0	132.1	4569	0 (0–2.79)
UVL (VL <200)	0	236.2	12,638	0 (0–1.56)
VL >200	0	5.17	290	0 (0–71.4)
STI diagnosed	0	23.2	1007	0 (0–15.9)
First 6 months ART	0	10.0	341	0 (0–36.9)

**Conclusions**: There were no linked HIV transmissions in almost 600 CYFU involving close to 17,000 acts of CLAI in HM-SDC. Our results provide strong support for the hypothesis that undetectable viral load prevents HIV transmission in homosexual men.

## TUAC0206LB

### Safety and acceptability trial of the dapivirine vaginal ring in US adolescents

K Bunge ^1^; L Levy^2^; D Szydlo^3^; J Zhang^3^; A Gaur^4^; D Reirden^5^; K Mayer^6^; D Futterman^7^; C Hoesley^8^; S Hillier^9^; M Marzinke^10^; C Dezzutti^9^; C Wilson^8^; L Soto-Torres^11^; B Kapogiannis^12^; A Nel^13^; K Squires^14^; MTN-023/IPM 030 Protocol Team

^1^Department of Obstetrics, Gynecology, and Reproductive Sciences, University of Pittsburgh, Pittsburgh, USA. ^2^FHI360, Washington, USA. ^3^FHCRC – SCHARP, Seattle, USA. ^4^St. Jude Children’s Research Hospital, Memphis, USA. ^5^Children’s Hospital Colorado, Denver, USA. ^6^Fenway Health/The Fenway Institute, Boston, USA. ^7^Children’s Hospital at Montefiore Medical Center, New York, USA. ^8^University of Alabama, Birmingham, USA. ^9^Magee Womens Research Institute, Pittsburgh, USA. ^10^Johns Hopkins University School of Medicine, Baltimore, USA. ^11^National Institue of Allergy and Infectious Disease, Divison of AIDS, Bethesda, USA. ^12^National Institute of Child Health and Human Development, Bethesda, USA. ^13^International Partnership for Microbicides, Western Cape, South Africa. ^14^Thomas Jefferson University, Philadelphia, USA

Presenting author email: kbunge@mail.magee.edu

**Background**: Young women aged 15–25 years are disproportionately affected by the HIV epidemic. Two Phase 3 trials of a 25mg dapivirine vaginal ring demonstrated HIV-1 risk reduction in adult women over 21, but not in those aged 18–21. Lack of protection was correlated with low adherence.

**Methods**: A Phase 2a randomized, double-blind, placebo-controlled trial of the dapivirine ring was conducted in sexually active females, aged 15–17. Participants were randomized 3:1 to a dapivirine or placebo ring to be inserted monthly for 6 months. Safety endpoints included Grade 2 product-related adverse events (AE) and Grade 3 and higher AEs. Adherence to ring use was assessed through self-report, plasma dapivirine concentrations and residual levels in used rings. A plasma dapivirine concentration >95 pg/mL was used to define short-term adherence (hours); a dapivirine residual level <23.5 mg was used to define long-term adherence (monthly). Acceptability was assessed through computer-assisted self-interviews.

**Results**: Ninety six participants were enrolled across six US sites. The mean age was 16.3 years; 59% were black and 34% white. Adherence to study visits was 97%. There were no differences in safety outcomes between treatment arms. By self-report, 42% (95% CI 32, 52) of participants reported that they never removed the ring except to replace it monthly. In the dapivirine group, drug levels indicated adherence in 87% of plasma samples and 95% of rings. Participants noted no discomfort due to the ring at 87% of visits and “liking” the ring at 93% of visits. The most frequently cited concern (28%) involved their primary sex partner feeling the ring during sex.

**Abstract TUAC0206LB–Figure 1.**
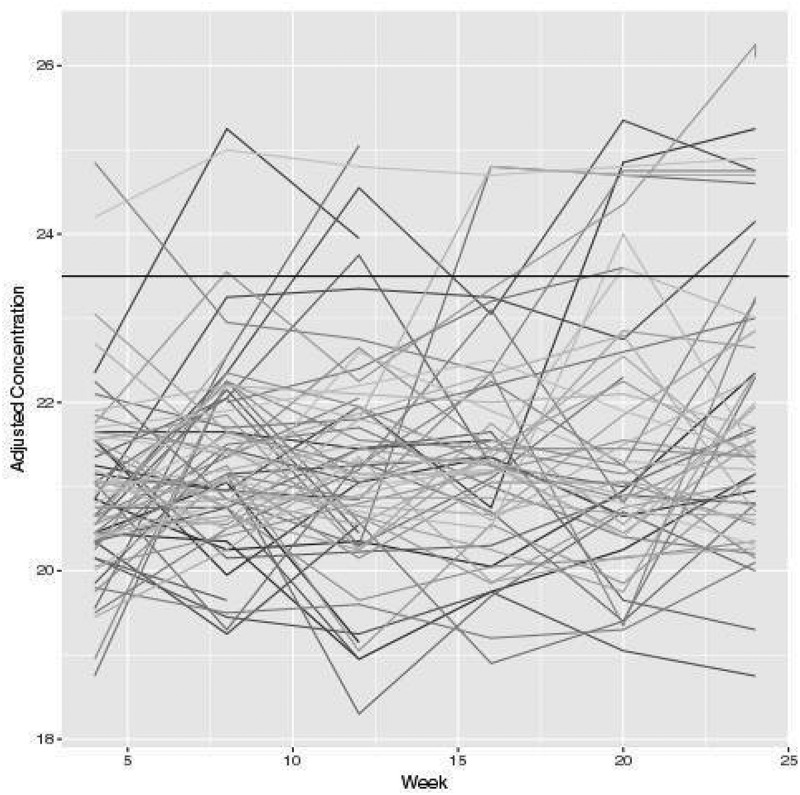
Spaghetti plot of residual dapivirine concentration.

**Conclusions**: The dapivirine vaginal ring, a promising microbicide approach, is safe and acceptable in this population. By self-report and objective measures, adherence was high. Discussing potential partner concerns with participants prior to ring use may positively influence adherence.

## TUAC0207LB

### Pluspills: an open-label, safety and feasibility study of oral pre-exposure prophylaxis (PrEP) in 15–19-year-old adolescents in two sites in South Africa

K Gill ^1^; J Dietrich^2^; G Gray^2^; T Pidwell^1^; F Kayamba^1^; T Bennie^1^; L Myer^3^; L Johnson^3^; H Spiegel^4^; C Slack^5^; V Elharrar^4^; A Strode^5^; J Rooney^6^ and L-G Bekker^1,3,7^

^1^Desmond Tutu HIV Foundation, Cape Town, South Africa. ^2^Perinatal HIV Research Unit, Johannesburg, South Africa. ^3^University of Cape Town, Cape Town, South Africa. ^4^NIH, Baltimore, USA. ^5^University of Kwazulu Natal, Pietermaritzburg, South Africa. ^6^Gilead Sciences, California, USA. ^7^Desmond Tutu HIV Centre, Cape Town, South Africa

Presenting author email: katherine.gill@hiv-research.org.za

**Background**: South African adolescents are at risk for HIV acquisition. PrEP is licensed and being offered to key populations, but not yet to adolescents. This open-label 12-month PrEP study examined uptake, safety and adherence to PrEP and assessed sexual risk behaviour among adolescents in Soweto and Cape Town, South Africa.

**Methods**: Sexually active, healthy, HIV negative, adolescents (15–19 years) participated in a study of Tenofovir/Emtricitabine PrEP. Participants were asked to take daily PrEP for at least 3 months, were seen monthly, after which they could opt-out of PrEP if preferred. At subsequent 3-monthly follow-up visits, participants could decide to stay on or off PrEP as per preference. Laboratory and clinical safety monitoring occurred at each visit, including HIV and pregnancy testing. Plasma and dried blood spots (DBS) were serially collected for tenofovir (TFV) and tenofovir diphosphate (TFV-DP) levels at every PrEP refill visit. Plasma TFV levels were offered at each visit as part of adherence counselling.

**Results**: 244 individuals were screened and 148 were enrolled (median age 18.67% F). 3(1%) had undiagnosed HIV infection and 9 (6%) were pregnant at screening. STI diagnosis at baseline was high (40%) and remained high throughout. 26 (18%) participants opted out of PrEP at 12 weeks. Thereafter, PrEP opt-out (and opt-in) at months 6 and 9 included 41% and 43% (5% and 7%) of the cohort, respectively. PrEP was safe and reasonably well tolerated. Plasma TFV levels were detectable in 57% of participants at Week 12, 38% at Week 24 and 38% at study end. One HIV seroconversion occurred on study (0.76/100 person-years) in a 19-year-old woman who had stopped PrEP 24 weeks prior to diagnosis.

**Conclusions**: Pluspills enrolled a cohort of self-selected adolescents at high risk of HIV acquisition and offered an opportunity to engage on ethical norms for adolescent research. PrEP was safe and tolerable in those who persisted. However, PrEP usage decreased and adherence diminished over time when visits became less frequent. STI diagnoses remained constant and HIV incidence was low. SA adolescents need access to PrEP with tailored adherence support and potentially augmented visit schedules.

## TUAD0206LB

### Willingness and ability of OST clients to pay for some OST services in Odesa region in the light of the Ukraine crisis

L Khomych ^1^; O Postnov^2^; N Avaliani^3^; O Iaremenko^3^ and M Duda^3^

^1^Deloitte Consulting Overseas Projects, LLC, USAID Project HIV Reforn in Action, Kyiv, Ukraine. ^2^Ukrainian Research Antiplague Institute named after Mechnikov, Odesa, Ukraine. ^3^Deloitte Consulting Overseas Projects, LLC, USAID Project HIV Reform in Action, Kyiv, Ukraine

Presenting author email: lkhomych@hss-share.net.ua

**Background**: Odesa region is one of the highest burden of HIV in Ukraine. HIV epidemic is mainly concentrated among key populations (PWIDs, CSWs and their partners, MSMs). The international donors’ support of OST programmes for PWIDs are currently in the process of financing reduction. The economic crisis in Ukraine complicated the nationwide state financing of OST programmes in Ukraine. The implementation of the alternative financing models such as co-payment is optimal way to secure critical for PWIDs services. The aim of the study is to assess the willingness and ability of current OST clients to pay for some OST services.

**Methods**: Cross-sectional study was conducted in Odesa city in 2016 among current OST clients in Odesa city. Random sampling strategy was used for respondent recruitment. Structured questionnaire was used for data collection. The study protocol received an approval of IRB at Ukrainian Institute on Public Health Policy. Bivariate analysis was used to estimate correlates of clients’ willingness to pay for some OST services.

**Results**: A total of 161 clients of OST programmes (125 (77.6%) males and 36 (22.4%) females) were interviewed. The average age of the respondents was 41.98 years (SD = 8.0). 66.5% of interviewed OST clients in Odesa city had a secondary education and 14.9% had a higher education. Half of the respondents (49.7%) had a single marital status. 74% of respondents uptake OST programme for more than 12 months. Overall, 89% of respondents (*n* = 143) demonstrate willingness to pay for some OST services in Odesa. The willingness to co-pay for some OST services is higher among clients listed “possibility to uptake additional medical care at OST site” as personal benefit of OST participation. The willingness is also correlated with income per family member (*p* = 0.009), self-assessment of financial wellbeing (*p* = 0.008) and perceived social support (*p* = 0.014).

**Conclusions**: Clients of OST programme in Odesa region demonstrated willingness and ability to pay for some OST services despite an economic crisis in the country. Their willingness depends on socio-economic status of PWID and his/her family. These important correlates of willingness and ability to pay should be considered for design and implementing OST co-payment service delivery model in Odesa region.

## TUAD0106LB

### Index partner testing and targeted case finding in northern Haiti

VA Francois

JHPIEGO, HIV TB, Cap Haitian, Haiti

Presenting author email: valerie.francois@jhpiego.org

**Background**: In Haiti, national guidelines recommend HIV testing for partners of people living with HIV (PLHIV), but uptake and documentation have been low. While some healthcare providers recommend partner testing, it is not prioritized and delivery is inconsistent. HIV-positive women may be reluctant to notify their partners for fear of abuse or abandonment, and additional strategies are needed to increase case finding, particularly among men.

**Methods**: Jhpiego-Haiti, through the USAID-funded Maternal and Child Survival Program, and in collaboration with the National AIDS Control Program (PNLS), attempted to strengthen index partner testing by introducing training and documentation at 20 health facilities in northern Haiti. The 2-day training, conducted in October 2016, addressed targeted case finding, and strategies for supporting index clients living with HIV to refer their partners for testing. Counselling and communication skills were reinforced, and data collection and reporting tools were presented. Providers offered counselling on partner testing to all newly diagnosed HIV-positive clients, and submitted monthly reports on key indicators for partner testing.

**Results**: From October 2016 to March 2017, a total of 593 index clients tested HIV-positive in 20 health facilities in northern Haiti, 63% of whom were women. 519 index clients (88%) accepted counselling for partner testing, and 112 (22%) of those agreed to refer their partners. Only 61 partners attended the clinic for HIV testing, 30 (49%) of whom were HIV-positive, and 31 of whom were in discordant couples. The primary reason partners were not referred is because index clients were afraid to disclose their status for fear of their partner’s reaction and stigmatization.

**Conclusions**: Training providers, equipping them with monitoring tools, and supporting them to counsel newly diagnosed PLHIV on partner testing can lead to the identification of HIV-positive partners and discordant couples. Further efforts to strengthen this targeted testing approach, including ongoing counselling and support for index patients to disclose, are needed to achieve the UNAIDS 90-90-90 targets in Haiti.

## WEAA0106LB

### Identification of a new factor involved in DNA methylation-mediated repression of latent HIV-1

S Bouchat^1^; R Verdikt^1^; N Delacourt^1^; C Vanhulle^1^; B Van Driessche^1^; G Darcis^1,2,3^; A Pasternak^3^; V Avettand-Fenoel^4^; V Ledouce^5^; M Bendoumou^1^; C Schwartz^6^; S De Wit^7^; B Berkhout^3^; V Gautier^5^; C Rouzioux^4^; O Rohr^6,8^ and C Van Lint ^1^

^1^Université Libre de Bruxelles (ULB), Service of Molecular Virology, Département de Biologie Moléculaire (DBM), Brussels, Belgium. ^2^Université de Liège, Service des Maladies Infectieuses, CHU de Liège, Domaine Universitaire du Sart-Tilman, B35, Liège, Belgium. ^3^Laboratory of Experimental Virology, Department of Medical Microbiology, Academic Medical Center of the University of Amsterdam, University of Amsterdam, Amsterdam, Netherlands. ^4^Université Paris-Descartes, AP-HP, Hôpital Necker-Enfants Malades, Service de Virologie, Paris, France. ^5^University College Dublin (UCD), UCD Centre for Research in Infectious Diseases (CRID), School of Medicine, College of Health and Agricultural Sciences, Dublin, Ireland. ^6^University of Strasbourg, Institut de Parasitologie et de Pathologie Tropicale, EA7292, Strasbourg, France. ^7^Université Libre de Bruxelles (ULB), Service of Infectious Diseases, CHU Saint-Pierre, Brussels, Belgium. ^8^University of Strasbourg, Institut Universitaire de Technologie Louis Pasteur de Schiltigheim, Schiltigheim, France

Presenting author email: cvlint@ulb.ac.be

**Background**: DNA methylation is an epigenetic mechanism of HIV-1 latency. The methylation profile of the latent viral 5’LTR is heterogeneous in latency model cell lines and in patient cells in which it increases progressively during cART. Moreover, we previously reported that the DNA methylation inhibitor decitabine (5-aza-2’deoxycytidine) induces different levels of HIV-1 reactivation in latently-infected T cell lines and *ex vivo* patient cell cultures. However, the mechanism of DNA methylation-mediated HIV-1 silencing remains unclear.

**Methods**: Sodium bisulphite sequencing, EMSAs, ChIP-qPCR assays, RNA interference, GFP fluorescence FACS, p24 ELISA experiments and purification of primary cells from HIV-positive patient blood.

**Results**: To explore this mechanism, we took advantage of two latently-infected J-Lat cell lines (the 8.4 and 15.4 clones) representing distinct integration sites and showed that these two cell lines exhibited similar levels of 5’LTR CpG methylation in basal conditions but different DNA demethylation extents in response to decitabine. Demethylation at CpG dinucleotides following decitabine-induced reactivation of HIV-1 production occurred at specific and reproducible CpG positions that differed depending on the two J-Lat cell lines studied. Interestingly, a site comprising one of this hotspot for decitabine-induced demethylation was shown to bind UHRF1 (Ubiquitin-like PHD and ring finger domain-containing protein 1), only in one of the J-Lat cell line, whereas DNMTs were recruited to both J-Lat cell lines. Treatment with decitabine caused a decreased *in vivo* UHRF-1 recruitment to the 5’LTR. UHRF1 knockdown using RNA interference or pharmacological approaches showed increased levels of HIV-1 transcription and production that were accompanied by an increased recruitment of RNA polymerase II to the 5’LTR. We are currently studying UHRF1 functional role in latently infected primary cells from aviremic cART-treated HIV-positive patients.

**Conclusions**: We have identified UHRF1 as a factor recruited to the HIV-1 5’LTR in a methylation- and integration-dependent manner during latency and which plays a functional role in DNA methylation-mediated repression of HIV-1 gene expression. UHRF1 is known to regulate and maintain heterochromatic equilibrium *via* its combined action on both DNA methylation and histone modifications. This factor has not previously been identified as a regulator of HIV latency and might thus constitute a new therapeutic target for HIV cure strategies.

## WEAB0106LB

### Zoledronic acid is superior to TDF-switching for increasing bone mineral density in HIV-infected adults with osteopenia: a randomized trial

J Hoy^1,2^; R Richardson^3^; PR Ebeling^1^; J Rojas^4^; N Pocock^5^; S Kerr^3^; E Martinez^4^; A Carr ^6^; Zoledronate or Switch Tenofovir (ZeST) Study Group

^1^Monash University, Melbourne, Australia. ^2^The Alfred Hospital, Melbourne, Australia. ^3^St Vincent’s Hospital, Centre for Applied Medical Research, Sydney, Australia. ^4^Hospital Clínic, University of Barcelona, Infectious Diseases Unit, Barcelona, Spain. ^5^St Vincent’s Hospital, Sydney, Nuclear Medicine, Sydney, Australia. ^6^St Vincent’s Hospital, Sydney, HIV, Immunology and Infectious Diseases Unit, Sydney, Australia

Presenting author email: andrew.carr@svha.org.au

**Background**: Tenofovir disoproxil fumarate (TDF) reduces bone mineral density (BMD) and probably increases fracture risk in HIV-infected adults. Proven strategies for improving BMD in HIV-infected adults on TDF are TDF switching or bisphosphonate therapy; which strategy is superior is unknown.

**Methods**: We randomized virologically suppressed, HIV-infected adults on TDF with low BMD (T score <−1.0 at hip or spine by dual-energy x-ray absorptiometry (DXA)) to either switch TDF or to continue TDF and receive intravenous zoledronic acid (ZOL) 5mg every 12 months. Calcium (all patients) and vitamin D (if serum 25OH vitamin D was <50 nmol/L) were supplemented. The primary study outcome was change in lumbar (L1-L4) spine BMD at 24 months by intention-to-treat analysis. Secondary outcomes included femoral neck and total hip BMD, fractures, safety and virological failure (confirmed viral load ≥400 cp/mL).

**Results**: We randomized 87 patients (44 to TDF switch and 43 to ZOL): mean age 50 years (SD 11), 96% men, mean TDF duration 5.9 years (SD 3.1), 22% on a boosted PI, mean spine and hip T scores −1.6 and −1.3, respectively. TDF switches were mostly to abacavir (62%) or raltegravir (19%). Adherence to each strategy was high: four switch patients (10%) recommenced TDF at a median of 9 months; 98% of ZOL doses were administered. Mean spine BMD changes at 24 months were 7.4% (SD 4.3%) with ZOL versus 2.9% (4.5%) with TDF-switching (mean difference 4.4%, 95% CI 2.6–6.3; *p* < 0.001). Mean left femoral neck BMD changes were 4.1% (3.8%) and 2.1% (4.6%), respectively (mean difference 2.0%, 95% CI 0.2–3.8; *p* = 0.03). Mean left total hip BMD changes were 4.6% (2.6%) and 2.6% (4.0%), respectively (mean difference 1.9%, 95% CI 0.5–3.4; *p* = 0.009). There was 1 fracture in the ZOL group (1 patient) and 7 separate fracture events in the TDF switch group (4 patients). Serious adverse events occurred in 9 (19%) ZOL patients and 6 (14%) TDF-switch patients; none was related to study drugs or procedures. Virological failure occurred in 1 TDF-switch patient and in no ZOL patient.

**Conclusions**: ZOL is more effective than TDF switching at increasing BMD in osteopenic adults on TDF, and may result in fewer fractures.

## WEAC0106LB

### Pre-exposure prophylaxis (PrEP) among men who have sex with men (MSM) in the Netherlands: motives to choose for, switch to or stop with daily or event-driven PrEP

H Zimmermann ^1^; S Eekman^1^; R Achterbergh^1,2^; M Prins^1,3^; M Schim van der Loeff^1^; H de Vries^2,3^; E Hoornenborg^1,2^; U Davidovich^1,3^; HIV Transmission Elimination Amsterdam (H-Team)

^1^Public Health Service Amsterdam, Infectious Diseases – Research & Prevention, Amsterdam, Netherlands. ^2^Public Health Service Amsterdam, Infectious Diseases – STI Outpatient Clinic, Amsterdam, Netherlands. ^3^Academic Medical Center of Amsterdam, Amsterdam, Netherlands

Presenting author email: hzimmermann@ggd.amsterdam.nl

**Background**: Proper implementation of pre-exposure prophylaxis (PrEP) among men who have sex with men (MSM) requires a clear understanding of the reasons why MSM choose one PrEP-dosing regimen over another in real-life settings. Therefore, we aimed to gain insight into the motives for choosing or switching between daily and event-driven PrEP or (temporarily) stopping PrEP use.

**Methods**: We used data from the Amsterdam PrEP (AmPrEP) demonstration study (June 2015–February 2017), where both daily and event-driven PrEP (dPrEP and edPrEP, respectively) are offered. MSM’s motives to choose a regimen were measured at baseline among 376 participants of whom 273 chose dPrEP and 103 chose edPrEP. Motives to switch or stop were recorded at every 3-monthly follow-up visit. Standardized closed- and open-end items were used. Open answers were coded and analysed following qualitative research methods.

**Results**: Among the reasons to use dPrEP were the convenience of daily routine (*n* = 133), perceived higher dPrEP efficacy (*n* = 34) and fear of side effects relating to edPrEP re-initiation (*n* = 5). Perceived toxicity and burden of daily medication were reasons to choose edPrEP (*n* = 38). Infection risk was also considered: dPrEP was preferred for unplanned and/or frequent sexual risk behaviour (*n* = 79), while edPrEP was chosen when risk was more predictable (*n* = 57). Some chose for, or switched to, edPrEP to inhibit sexual risk behaviour (*n* = 4), while others chose for, or switched to, dPrEP to gain more sexual freedom (*n* = 17). Other reasons to switch to edPrEP included experiencing side effects (*n* = 14), having less sex than anticipated (*n* = 20), experimenting with another regimen (*n* = 2) and receiving negative reactions from the environment (*n* = 1). Doubts about edPrEP’s safety (*n* = 2), inability to plan sex (*n* = 13) and desire for more structure (*n* = 9) were motivators to switch to dPrEP. Motives to temporarily stop dPrEP (*n* = 99) were situational (e.g. medical issues or vacations). Changed life circumstances (*n* = 2) and reduced sexual risk (*n* = 6) were motives to completely stop with PrEP use (*n* = 12).

**Conclusions**: A great variety of personal and contextual factors determine the choices for PrEP regimens, related switches and stops. In order to successfully support future PrEP users, a tailored approach, addressing choices for PrEP regimens as a continuum of flexible and changeable choices, is essential.

## WEAD0206LB

### Cost-effectiveness of routine viral load monitoring in low- and middle-income countries: a systematic review

RV Barnabas ^1^; P Revill^2^; N Tan^1^ and A Phillips^3^

^1^University of Washington, Seattle, USA. ^2^University of York, York, UK. ^3^University College London, London, UK

Presenting author email: rbarnaba@uw.edu

**Background**: Routine viral load (VL) monitoring for HIV-1 management of persons on ART has been recommended by the WHO to identify treatment failure. However, VL testing represents a substantial cost in resource-constrained healthcare systems. The central challenge is whether and how VL monitoring may be delivered such that it maximizes health gains across the population for the costs incurred. We hypothesized that key efficiency assumptions about programme design and cost drive the cost-effectiveness of programmes with viral load monitoring.

**Methods**: We conducted a systematic review of studies on the cost-effectiveness of viral load monitoring in low- and middle-income countries (LMIC). We followed the Cochrane Collaboration guidelines and the PRISMA reporting guidelines.

**Results**: We identified 18 studies that evaluated the cost-effectiveness of viral load monitoring in HIV treatment programmes. Overall, we identified three key factors that make it more likely for viral load monitoring to be cost-effective: (1) Use of lower cost, robust approaches to viral load monitoring; (2) Ensuring the pathway to health attainment is established and that viral load results are acted upon; (3) Viral load result is used to simplify HIV care in patients with viral suppression (i.e. differentiated care, with fewer clinic visits and longer prescriptions); viral load monitoring in differentiated care programmes provides evidence that reduced clinical engagement, where appropriate, is not impacting health outcomes.

**Conclusions**: The cost-effectiveness of viral load monitoring critically depends on the context. To achieve this goal of being cost effective, viral load monitoring will need to facilitate scale up of differentiated care approaches to HIV treatment – introducing viral load monitoring without differentiated care is unlikely to be cost-effective in most settings and results in lost opportunity for health gains through an alternative use of limited resources. As countries scale up differentiated care programmes, data on clinical outcomes and cost are essential to evaluate the ongoing cost-effectiveness of viral load monitoring as utilized in practice.

## WEAD0106LB

### The cost-effectiveness of integrating maternal ART into maternal & child health (MCH) services during the postpartum period in South Africa

C Dugdale ^1,2,3^; T Phillips^4,5^; L Myer^4,5^; EP Hyle^1,2,6^; K Brittain^4,5^; KA Freedberg^1,2,6^; L Cunnama^7^; R Walensky^1,2,6^; A Zerbe^8^; EJ Abrams^8,9^; A Ciaranello^1,2^; the MCH-ART Trial Team

^1^Medical Practice Evaluation Center, Massachusetts General Hospital, Boston, USA. ^2^Department of Medicine, Division of Infectious Diseases, Massachusetts General Hospital, Boston, USA. ^3^Department of Medicine, Division of Infectious Diseases, Brigham & Women’s Hospital, Boston, USA. ^4^Division of Epidemiology and Biostatistics, School of Public Health & Family Medicine, University of Cape Town, Cape Town, South Africa. ^5^Centre for Infectious Diseases Epidemiology & Research, School of Public Health & Family Medicine, University of Cape Town, Cape Town, South Africa. ^6^Division of General Internal Medicine, Massachusetts General Hospital, Boston, USA. ^7^Health Economics Unit, School of Public Health and Family Medicine, University of Cape Town, Cape Town, South Africa. ^8^ICAP at Columbia, Mailman School of Public Health, Columbia University, New York, USA. ^9^College of Physicians & Surgeons, Columbia University, New York, USA

**Background**: Despite improved PMTCT services in South Africa, poor retention in care and low maternal ART adherence after delivery increase risk of postnatal MTCT and adverse maternal outcomes. We projected the clinical and economic impact of the MCH-ART trial, which evaluated integrated, co-located maternal and paediatric care throughout breastfeeding in Cape Town.

**Methods**: Using the CEPAC model, we simulated HIV-infected women initiating ART during pregnancy and their infants (mean maternal age: 28.6 y, median CD4: 354 cells/µL). We compared two strategies: routine care (referral to local clinics after delivery for separate adult ART and well-baby care) and integrated maternal/paediatric care in the MCH service through weaning (median: 8 m, then referral to local clinics). Trial-derived inputs included: primary trial outcomes of 12-month maternal retention (routine: 71%, integrated: 81%) and virologic suppression (routine: 56%, integrated: 77%); breastfeeding duration (routine: 6 m, integrated: 8 m); and infant HIV testing uptake at 6–10 w (routine: 78%, integrated 82%). We assumed an intervention cost of $200/mother–infant pair and equal monthly LTFU and ART failure rates in both strategies after 12 months. Model outcomes included MTCT rates, maternal and paediatric life expectancy (LE), and lifetime HIV-related per-person costs (2014 US$). We calculated incremental cost-effectiveness ratios (ICERs) from discounted (3%/year) maternal + paediatric LE and costs, defining “cost-effective” as an ICER < $6500/life-year saved (South Africa 2014 *per-capita* GDP).

**Results**: Compared to routine care, integrated care was projected to decrease maternal 1-year mortality (1.7% vs. 1.5%), increase maternal LE (27.0 y vs. 28.6 y, undiscounted) and result in an ICER of $940/LYS. Modelled paediatric outcomes were similar between arms. The intervention remained cost-effective with lower intervention efficacy (50%: Table 1), higher intervention costs (up to $4650) and longer breastfeeding duration (12 m).

**Conclusions**: Integrated maternal-paediatric care, co-located in MCH services through the end of breastfeeding, is a cost-effective strategy to optimize postpartum maternal and infant outcomes in South Africa.

**Abstract WEAD0106LB–Table 1.** Result of clinical and economic analysis of integrated postpartum maternal-infant care intervention in South Africa.

## LATE BREAKER POSTER DISCUSSION ABSTRACTS

## MOPDC0106LB

### Breast milk dapivirine pharmacokinetics and estimated infant exposure during dapivirine intravaginal ring use among lactating women

L Noguchi ^1,2^; R Beigi^3^; J Biggio^4^; M Marzinke^1^; C Kelly^5^; R Scheckter^6^; K Bunge^3^; J Piper^7^; S Hillier^3^; A Nel^8^ and C Hendrix^1^

^1^Johns Hopkins University, Baltimore, USA. ^2^MCSP, Washington, USA. ^3^Magee-Womens Hospital/University of Pittsburgh, Pittsburgh, USA. ^4^Ochsner Health System, New Orleans, USA. ^5^SCHARP, Seattle, USA. ^6^FHI 360, Durham, USA. ^7^DAIDS/NIAID/US NIH, Rockville, USA. ^8^International Partnership for Microbicides, Silver Spring, USA

Presenting author email: lnoguch1@jhu.edu

**Background**: The 25mg dapivirine (DPV) vaginal ring (VR) can reduce women’s risk of acquiring HIV infection. Most studies of HIV prevention products exclude lactating women, despite global recommendations for breastfeeding and continued risk for HIV acquisition during lactation. MTN-029/IPM 039 was a Phase I, open-label study of pharmacokinetics; safety and tolerability, and adherence associated with DPV VR use.

**Methods**: Between January 2016 and March 2017, 16 healthy, HIV-uninfected women aged 18 or older were enrolled in Pittsburgh and Birmingham, USA. Eligible women had weaned infants from breastfeeding before enrolment, but were able to pump breast milk. Participants were instructed to wear the VR continuously for 14 days. Milk and blood plasma samples were collected (pre-insertion, Hour 3, Hour 6, Hour 24, Day 7, Day 14 after ring placement, and two days after ring removal) and analysed for DPV using validated ultra-performance liquid chromatography-tandem mass spectrometry assays, with lower limits of quantification of 10 pg/mL and 20 pg/mL for milk and plasma, respectively. We estimated infant DPV intake assuming 150 mL/kg/day milk ingestion. Adverse events (AEs) were collected at all participant contacts.

**Results**: All participants had detectable DPV in milk and plasma. Median DPV concentrations in milk and plasma rose gradually to relatively steady concentrations on Day 7 and Day 14, followed by falling concentrations after ring removal to approximately 40% of Day 14 concentrations by Day 16. Median (interquartile range) peak concentration for milk and plasma were 676 pg/mL (443, 924.5) and 327 pg/mL (274.5, 378), respectively. Time-adjusted median milk/plasma ratio was 1.70 (1.38, 1.86). Estimated daily infant exposure was 68.0 ng/kg/day (53.0, 85.1). Estimated terminal concentration half-life after ring removal was 39.0 hours (27.1, 53.4) and 35.2 hours (29.8, 46.4) for milk and plasma, respectively. Six of 16 (38%) participants experienced eight total AEs, most of which were mild.

**Conclusions**: In this first study of DPV exposure during lactation, DPV VR use was associated with low levels of detectable DPV in milk and plasma, very low estimated levels of infant exposure and a favourable safety profile. Future DPV VR studies should evaluate longer periods of use among breastfeeding mother–infant pairs.

## TUPDB0201LB

### Phase 3 randomized, controlled clinical trial of bictegravir coformulated with FTC/TAF in a fixed-dose combination (B/F/TAF) versus dolutegravir (DTG) + F/TAF in treatment-naive HIV-1 positive adults: Week 48 results

PE Sax ^1^; A Pozniak^2^; J Arribas^3^; E Koenig^4^; E Dejesus^5^; H-J Stellbrink^6^; A Antinori^7^; K Workowski^8^; J Slim^9^; J Reynes^10^; W Garner^11^; D Sengupta^11^; H Martin^11^; E Quirk^11^ and A Cheng^11^

^1^Brigham and Women’s Hospital, Boston, USA. ^2^Chelsea & Westminster Hospital, London, UK. ^3^Hospital Universitario La Paz, Mardid, Spain. ^4^Instituto Dominicano de Estudios Virologicos IDEV, Santo Domingo, Dominican Republic. ^5^Orlando Immunology Center, Orlando, USA. ^6^ICH Study Center, Hamburg, Germany. ^7^Hospital Clinico Universitario de Santiago, Santiago De Compostela, Spain. ^8^Emory University, Atlanta, USA. ^9^Saint Michael’s Medical Center, Newark, USA. ^10^CHU Gui De Chauliac, Montpellier, France. ^11^Gilead Sciences Inc., Foster City, USA

Presenting author email: psax@bwh.harvard.edu

**Background**: In a Phase 2 study, bictegravir (BIC, B), a novel, potent integrase strand transfer inhibitor (INSTI) with a high barrier to resistance, was directly compared with dolutegravir (DTG), each given with the recommended N(t)RTI combination of emtricitabine and tenofovir alafenamide (F/TAF) in treatment-naive, HIV-infected adults. Both treatments demonstrated high efficacy and were well tolerated through Week (W) 48. We now report results from a Phase 3 study of this comparison of BIC and DTG, each with F/TAF, utilizing a single-pill co-formulation of B/F/TAF.

**Methods**: Treatment-naive, HIV-infected adults with estimated glomerular filtration rate (eGFR) ≥30 mL/min were randomized 1:1 to receive blinded treatment with fixed dose combination B/F/TAF (50/200/25 mg) or DTG (50 mg) + F/TAF (200/25 mg) with matching placebos once daily through W48. Chronic hepatitis B and/or C infection was allowed. The primary endpoint was the proportion of participants with HIV-1 RNA <50 copies/mL (c/mL) at W48 (FDA snapshot). Non-inferiority was assessed through 95.002% confidence intervals (CI) using a margin of 12%. Secondary endpoints were safety measures (adverse events (AEs) and laboratory results).

**Results**: 645 participants were randomized and treated (320 B/F/TAF, 325 DTG + F/TAF): 12% women, 31% Black, 19% viral load (VL) >100,000 c/mL, 12% CD4 <200 cells/µL, median age 34 years, CD4 count 440 cells/µL, and VL 4.44 log10 c/mL. At W48, B/F/TAF was non-inferior to DTG + F/TAF, with 89.4% on B/F/TAF and 92.9% on DTG + F/TAF achieving HIV-1 RNA <50 c/mL (difference −3.5%; 95.002%CI −7.9% to 1.0%, *p* = 0.12). At W48, proportion of participants with HIV-1 RNA ≥50 c/mL was <1% in each arm. No study subject in either treatment arm developed resistance to any of the study drugs. The most common AEs were headache (13% B/F/TAF, 12% DTG + F/TAF) and diarrhoea (12% for both). Few participants (5 (2%), 1 (<1%)) had AEs leading to premature study discontinuation. Lipid changes were not significantly different between study arms. No renal discontinuations and no cases of proximal renal tubulopathy were reported.

**Conclusions**: After 48 weeks, B/F/TAF achieved virologic suppression in 89.4% of treatment-naive adults and was non-inferior to DTG + F/TAF. B/F/TAF was safe and well tolerated.

## TUPDB0202LB

### Single doses as low as 0.5 mg of the novel NRTTI MK-8591 suppress HIV for at least seven days

RP Matthews ^1^; D Schürmann^2^; DJ Rudd^1^; V Levine^1^; S Fox-Bosetti^1^; S Zhang^1^; M Robberechts^1^; A Huser^2^; DJ Hazuda^1^; M Iwamoto^1^ and JA Grobler^1^

^1^Merck & Co., Inc., Kenilworth, USA. ^2^Charité Universitätsmedizin Berlin, Research Hospital, Berlin, Germany

Presenting author email: randolph.matthews@merck.com

**Background**: MK-8591 is a nucleoside reverse transcriptase translocation inhibitor (NRTTI) in early clinical development for the treatment of HIV-1 infection. MK-8591-triphosphate (MK-8591-TP), the active phosphorylated anabolite of MK-8591, exhibits a half-life of ~150–160 hrs in human PBMCs. Here, we describe antiviral efficacy and tolerability of single doses of 0.5 mg to 30 mg MK-8591 over 7 to 10 days in HIV-1 infected subjects in an ongoing Phase 1b monotherapy proof-of-concept efficacy study.

**Methods**: In an open-label study in HIV-1-infected subjects naive to antiretroviral treatment (ART), subjects are being administered a single dose of MK-8591 across a range of doses. Blood samples are being collected for viral load (VL), MK-8591 PK and MK-8591-TP PK at pre-specified time points up to 7 to 10 days post-dose. Following completion of Day 7 or Day 10 procedures, subjects are being offered standard of care ART. Safety, PK and VL data from the doses of 0.5 mg, 1 mg, 2 mg, 10 mg and 30 mg (*N* = 6/panel) are available.

**Results**: Single doses of MK-8591 across the entire tested range were associated with a rapid and robust reduction in VL. At 168 hours postdose, a mean (95% CI) placebo-corrected VL reduction of 1.18 log10 (0.95, 1.46) was observed for 0.5mg. For the 30mg dose, mean VL continued to decline through Day 10 with a mean placebo-corrected reduction of 1.57 log10 (1.34, 1.85), with no evidence of recrudescence at any dose. In samples with sufficient VL for testing (14/24), no common mutant strains, including M184V/I, were detected. All doses were generally well tolerated, with a limited number of mild/moderate adverse experiences reported. MK-8591 plasma and MK-8591-TP PBMC PK were similar to previously observed data in healthy subjects.

**Conclusions**: MK-8591 suppressed HIV replication for at least seven days when administered as a single dose as low as 0.5mg. The antiviral potency, human pharmacokinetics (PK) and physical properties of MK-8591 have the potential to open new paradigms for extended duration HIV treatment and prophylaxis approaches.

## TUPDB0203LB

### Pharmacokinetics, pharmacodynamics and pharmacogenomics of efavirenz 400mg once-daily during pregnancy and postpartum

M Lamorde ^1^; X Wang^2^; M Neary^3^; E Bisdomini^4^; S Nakalema^1^; P Byakika^1^; J Mukonzo^1^; W Khan^4^; A Owen^3^; M McClure^2^ and M Boffito^2,4^

^1^IDI, Kampala, Uganda. ^2^Imperial College, London, UK. ^3^University of Liverpool, Liverpool, UK. ^4^SSCR, Chelsea and Westminster Hospital, London, UK

Presenting author email: marta.boffito@nhs.net

**Background**: Antiretroviral dose reductions may compensate for the finite global manufacturing capacity and allow access programmes to reach larger numbers of HIV-infected patients. The ENCORE-1 study showed that efavirenz 400mg (EFV400) is non-inferior to the standard adult dose. WHO clinical guidelines now recommend EFV400 as an alternative first-line agent, however with a disclaimer that no data on EFV400 during the third trimester of pregnancy (TT) exist. This study investigated the pharmacokinetics (PK), efficacy and *CYP2B6* pharmacogenetics of EFV400 in women living with HIV (WLWH) during TT and post-partum (PP) with a view to removing the disclaimer and allowing wider EFV400 use in first-line.

**Methods**: Open-label, multicentre study (UK and Uganda) in WLWH receiving tenofovir disoproxil fumarate (TDF), emtricitabine (FTC) and EFV 600mg with an undetectable viral load (VL), who switched to TDF/FTC/EFV400 was performed. Weekly therapeutic drug monitoring (TDM), steady-state PK profiles during TT and PP, safety, virologic efficacy and polymorphisms in CYP2B6 (516C>T; 938T>C) were evaluated.

**Results**: 22 WLWH of African origin were enrolled, baseline median (range) age and CD4 were 30 (18–41) years and 548 (190–882) cells/mm^3^, respectively. All had VL <50 copies/mL at baseline, which was maintained throughout the study (only 2 blips were observed but confirmed <50, when repeated). None of the children were HIV-infected. No WLWH were excluded from the study because of low EFV400 TDM results (<800 ng/mL in >3 consecutive visits). Geometric mean (GM) ratios (TT/PP; 90% confidence intervals) of EFV400 Cmax, AUC and C_24h_ were 0.86 (0.68–1.09), 0.74 (0.59–0.94), 0.62 (0.47–0.83). GM C_24h_ was 1256 ng/mL (coefficient of variation 79%). 20/22 WLWH were carriers of the CYP2B6 516G allele and only 2 were slow metabolizers. EFV400 was well tolerated during pregnancy with no Grade 3 or 4 laboratory abnormalities.

**Conclusions**: Cmax, AUC and C_24h_ in TT were 14%, 26% and 38% lower compared to PP but within ranges of those measured for EFV600 during TT by Schalkwijk et al. (2016) and those measured in ARV-naive patients on EFV400 in ENCORE-1 (Dickinson et al. 2015). All subjects maintained a VL <50, suggesting that EFV400 can be used in pregnant WLWH.

## TUPDB0204LB

### Universal sputum testing versus symptom-based testing for tuberculosis (TB) in HIV-infected pregnant women: a cluster-randomized implementation trial in South Africa

N Martinson ^1^; K Motihaoleng^1^; E Variava^2^; G Barnes^3^; P Abraham^1^; L Lebina^1^; S Cohn^3^; L Moulton^4^; N Salazar-Austin^3^ and R Chaisson^3^

^1^Perinatal HIV Research Unit, University of the Witwatersrand, Soweto, South Africa. ^2^Klerksdorp Tshepong Hospital Complex, Internal Medicine, Klerksdorp, South Africa. ^3^Johns Hopkins University, Center for TB Research, Baltimore, USA. ^4^Bloomberg School of Public Health, Johns Hopkins University, International Health, Baltimore, USA

Presenting author email: martinson@phru.co.za

**Background**: TB in HIV-infected pregnant women is a leading cause of maternal and infant morbidity and mortality. Currently-recommended symptom-based screening of HIV-infected pregnant women may be insensitive.

**Methods**: We conducted a cluster-randomized trial to compare universal sputum TB testing of HIV-infected pregnant women against standard symptom-based testing. Sixteen public-sector antenatal clinics in two health districts were assigned to either strategy by constrained randomization. HIV-infected pregnant women without currently diagnosed TB were eligible. In universal testing clinics (UC), all HIV-positive pregnant women were asked to produce a sputum sample. In symptom clinics (SC), only those with WHO criteria for TB testing (cough, fever, night sweats or weight loss) were asked to produce sputum. Sputa were tested by Xpert MTB/RIF and midway through the study liquid MGIT culture was added. Women and infants were followed through 2 months postpartum. Cluster-adjusted results are shown.

**Results**: From May 2015 through March 2017, 937 and 1095 HIV-infected pregnant women were enrolled in the UCs and SCs, respectively. Median age was 30 years, median gestational age 24 months, 11% had prior TB, 90% were on ART, with no significant differences by arm. At baseline, 17% of UC women and 22% of SC women had >1 TB symptom (*p* = 0.40).

In UCs and SCs, respectively, 35 and 4 women were diagnosed with TB during pregnancy (UC prevalence = 3.7%, SC 0.37%, adjusted *p* = 0.01). Two months post-partum, infant mortality in UCs was 0.9% versus 2.1% in SCs (adjusted *p* = 0.06). Miscarriages and stillbirths were similar in both arms and two women died in the SCs.

MGIT culture identified more TB than Xpert: 25/487 samples (5%) were MGIT+ versus 19/1400 (1.4%) Xpert+ (*p* < 0.05). 440 samples were tested with both assays, 4 were Xpert+/MGIT+, 412 negative for both, 3 Xpert+/MGIT- and 21 Xpert-/MGIT+.

**Conclusions**: Universal TB screening of all HIV-infected pregnant women increased case detection 10-fold and was associated with reduced early infant mortality. MGIT identified more TB than Xpert in women whose pregnancy may mask TB symptoms. Our data support sputum testing all HIV-infected pregnant women for TB in high burden areas such as South Africa. Cost-effectiveness studies of universal testing are needed.

## TUPDB0205LB

### Sub-study 202094 of SWORD 1 & SWORD 2: switch from TDF containing regimen to DTG+RPV improves bone mineral density and bone turnover markers over 48 weeks

G McComsey ^1^; J Gonzalez-Garcia^2^; S Lupo^3^; J De Wet^4^; D Parks^5^; L Kahl^6^; B Wynne^7^; M Gartland^8^; K Angelis^9^; M Cupo^10^; K Vandermeulen^11^ and M Aboud^6^

^1^Case Western Reserve University, UH Clinical Research Center, Cleveland, USA. ^2^Hospital Universitario la Paz. IdiPAZ, Madrid, Spain. ^3^CAICI Instituto Centralizado, de Asistencia e Investigación Clínica Integral, Santa Fe, Argentina. ^4^Spectrum Health, Vancouver, Canada. ^5^Southampton Clinical Research Group, St. Louis, USA. ^6^ViiV Healthcare, Brentford, UK. ^7^ViiV Healthcare, Collegeville, USA. ^8^ViiV Healthcare, Research Triangle Park, USA. ^9^GlaxoSmithKline, Uxbridge, UK. ^10^GlaxoSmithKline, Collegeville, USA. ^11^Janssen, Beerse, Belgium

**Background**: Loss of bone mineral density (BMD) has been attributed to traditional risk factors for osteoporosis, HIV infection and antiretroviral therapy (ART), in particular tenofovir (TDF). 202094, a sub-study of the international, multicentre SWORD 1 & 2 studies, evaluated change in BMD (by DEXA) following switch from a triple ART regimen containing TDF to the 2-drug regimen (2DR) dolutegravir (DTG) + rilpivirine (RPV). Week 48 SWORD data demonstrated maintenance of suppression with DTG+RPV and non-inferiority to continuing current ART (CAR).

**Methods**: Subjects were HIV-infected adults, with HIV-1 RNA <50 c/mL and who received ART containing TDF both for ≥6 months prior to randomization to DTG+RPV or CAR on Day 1 through Week 48 in SWORD-1/2. Hip and lumbar spine BMD were measured by DEXA scans which were centrally read. The primary endpoint was the percentage change in total hip BMD. Secondary endpoints included change in lumbar spine BMD and bone turnover markers. The ANCOVA BMD analysis of the intent-to-treat population adjusted for baseline parameters (see Table 1).

**Results**: The results at Week 48 are shown in Table 1. DTG + RPV patients had an increase from Baseline to Week 48 in hip (1.34%) and spine BMD (1.46%) which differed statistically significantly (*p *= 0.014, *p *= 0.039, respectively) from CAR patients (Table 1). Consistent with this, DTG + RPV patients had a decrease from Baseline to Week 48 in bone-specific alkaline phosphatase, procollagen type 1-N-propeptide, osteocalcin and Type I Collagen C-Telopeptide bone turnover markers which differed statistically significantly from the CAR group (*p* range from <0.001 to 0.029 across markers).

**Abstract TUPDB0205LB–Table 1.** 202094 Week 48 results: ITT-exposed population.

Treatment assignment in parent SWORD study	DTG + RPV	CAR	*p*-Value
Evaluable subjects at Baseline and Week 48^a^	46	35	
**Primary endpoint: total Hip^b^ BMD**			
Mean adjusted percentage change from Baseline to Week 48 (95% CI)^c^	1.34 (0.68, 2.01)	0.05 (−0.71, 0.82)	
Difference in adjusted percentage change from Baseline to Week 48 between treatment groups (95% CI) and *p*-value^c^	1.29 (0.27, 2.31)		*p* = 0.014
**Secondary endpoint: lumbar spine^d^ BMD**			
Mean adjusted percentage change from Baseline to Week 48 (95% CI)^c^	1.46 (0.65, 2.28)	0.15 (−0.79, 1.09)	
Difference in adjusted percentage change from Baseline to Week 48 between treatment groups (95% CI) and *p*-value^c^	1.32 (0.07, 2.57)		*p* = 0.039

^a^Subjects having evaluable BMD data at both Baseline and Week 48 had their DEXA scans performed within the predefined time period for the study visit. ^b^Total hip includes the femoral neck, trochanter and intertrochanter areas. ^c^BMD is expressed as areal density. Estimates and associated *p*-values are from an ANCOVA model adjusted for Baseline BMD value, age at study entry and Baseline BMI. ^d^Lumbar spine includes the first lumbar vertebra (L1) to the fourth lumbar vertebra (L4).

**Conclusions**: Switch to the 2 drug-regimen of DTG+RPV is associated with significant improvement in bone mineral density and markers of bone health compared to continuation of a TDF-based 3 drug regimen, and provides a robust option for preserving bone health while continuing suppressive HIV treatment.

## TUPDB0206LB

### Earlier treatment and lower mortality in infants initiating antiretroviral therapy at <12 weeks of age in South Africa: The International epidemiologic Databases to Evaluate AIDS Southern Africa (IeDEA-SA) collaboration

V Iyun ^1^; K Technau^2^; B Eley^3^; H Rabie^4^; A Boulle^5,6^; G Fatti^7^; F Tanser^8,9^; R Wood^10^; L Fairlie^11^ and M-A Davies^1^

^1^University of Cape Town, Centre for Infectious Disease Epidemiology and Research, Cape Town, South Africa. ^2^University of the Witwatersrand, Rahima Moosa Mother and Child Hopsital Hospital, Johannesburg, South Africa. ^3^Red Cross War Memorial Children’s Hospital and Department of Paediatrics, University of Cape Town, Cape Town, South Africa. ^4^Tygerberg Academic Hospital and Department of Paediatrics, University of Stellenbosch, Stellenbosch, South Africa. ^5^Centre for Infectious Disease Epidemiology and Research, School of Public Health and Family Medicine, University of Cape Town, Cape Town, South Africa. ^6^Western Cape Department of Health, Khayelitsha ART Programme and Department of Health Impact Assessment, Cape Town, South Africa. ^7^Kheth’impilo, Cape Town, South Africa. ^8^Africa Centre for Population Health, Durban, South Africa. ^9^School of Nursing and Public Health, University of Kwazulu Natal, Durban, South Africa. ^10^University of Cape Town, Gugulethu ART Programme and Desmond Tutu HIV Centre, Cape Town, South Africa. ^11^University of the Witwatersrand, Wits Reproductive Health and HIV Research Institute, Johannesburg, South Africa

Presenting author email: toyiniyun@gmail.com

**Background**: The context of HIV prevention and treatment for children in South Africa has significantly improved and there is a recent shift towards birth early infant diagnosis and early infant antiretroviral therapy (ART). We describe the characteristic and outcomes of children initiating ART in the context of changing paediatric HIV testing and treatment guidelines in South Africa.

**Methods**: A retrospective cohort analysis was conducted using data from the IeDEA-SA collaboration. HIV-infected children initiating combination ART at <3 months old, from 2006 to 2016 were included. We described changes in characteristics of children starting ART and mortality, loss to follow-up (LTFU) and transfer out by 12 months on ART.

**Results**: Among 1380 infants, the median age at ART initiation was 62 days (interquartile range (IQR) 37–79); median time on ART was 13.6 months (IQR 4.0–34.5). The median age at ART start decreased from 67 days (IQR 53–81) in 2006–2009 to 48 days (IQR 9–74) in 2013+ (*p* < 0.001). There was a decline in median log viral load at ART initiation from 5.9 (IQR 5.4–6.4) in 2006–2009 to 5.4 (IQR 3.9–6.3) in 2013+ (*p* < 0.001). The median absolute CD4 count (cells/µL) increased progressively from 888 (IQR 380–1703) in 2006–2009 to 1526 (IQR 659–2231) in 2013+ (*p* < 0.001). Among infants with data on WHO disease staging (*n* = 865), 84% (*n* = 299) started ART with WHO disease stage 3/4 in 2006–2009 compared to 39% (*n* = 279) in 2013+ (*p* < 0.001). After 1 year on ART, 11% (median age 68 days (IQR 55–75)) and 4% (median age 60 days (IQR 25–83)) of children died in 2006–2009 and 2013+, respectively (*p* < 0.001). LTFU increased from 7% in 2006–2009 to 21% in 2013+ (*p* = 0.002) and transfer out declined from 20% in 2006–2009 to 12% in 2013+ (*p* < 0.001).

**Conclusions**: Children are starting ART earlier, with less progressive disease and associated declines in mortality; however, mortality and LTFU in infants starting ART remains unacceptably high. In view of the scale up of birth PCR testing in South Africa, a significant proportion of children still start ART with advanced disease, highlighting the need for a focused approach to early infant HIV testing and follow-up on ART.

## TUPDB0106LB

### Viral and host characteristics of a child with perinatal HIV-1 following a prolonged period after ART cessation in the CHER trial

A Violari ^1^; M Cotton^2^; L Kuhn^3^; D Schramm^4^; M Paximadis^4^; S Loubser^4^; S Shalekoff^4^; B da Costa Dias^4^; K Otwombe^1^; A Liberty^1^; J McIntyre^5,6^; A Babiker^7^; D Gibb^7^ and C Tiemessen^4^

^1^University of the Witwatersrand, Perinatal HIV Research Unit, Faculty of Health Sciences, Johannesburg, South Africa. ^2^Family Clinical Research Unit, Department of Paediatrics and Child Health, Stellenbosch University, Stellenbosch, South Africa. ^3^Department of Epidemiology, Mailman School of Public Health, Columbia University, New York, USA. ^4^University of the Witwatersrand, Centre for HIV and STIs, National Institute for Communicable Diseases and Faculty of Health Sciences, Johannesburg, South Africa. ^5^Anova Health Institute, Johannesburg, South Africa. ^6^Univerity of Cape Town, School of Public Health & Family Medicine, Cape Town, South Africa. ^7^University College London, Medical Research Council, Clinical Trials Unit, London, UK

Presenting author email: violari@mweb.co.za

**Background**: In the 6-year CHER trial (2005–2011), HIV-infected infants were randomized to deferred antiretroviral therapy (ART) or early limited ART for 40 (ART-40W) or 96 (ART-96W) weeks; ART reinitiation was based on CD4 and clinical criteria. We describe a child, randomized to ART-40W in 2008, who on long term follow-up, maintains an undetectable viral load after 8.5 years off-ART.

**Methods**: Studies conducted to describe immunological and viral characteristics included: ultrasensitive qualitative nested and quantitative semi-nested PCR assay to assess HIV DNA reservoir; co-culture of CD4 cells with MOLT-4/CCR5 and CD8-depleted phytohaemagglutinin-activated lymphocytes to detect replication-competent virus.

**Results**: HIV diagnosis was confirmed by HIV-DNA PCR+ at age 32 days, and on Days 39 and 60, VL was >750,000 and 151,000 copies/ml, respectively. ART started at age 8.7 weeks and was interrupted at 40 weeks post randomization. On ART, VL declined to <50 copies/ml at Week 24 and was <20 copies/ml post-interruption. During later follow-up, 6-monthly VL were also <20 copies/ml. At age 9.5 years, the child was clinically asymptomatic with CD4 802 cells/µl. Qualitative DNA PCR was negative. HIV-antibody by ELISA was negative but was weakly reactive to Gag p40 and p24 on Western blot; a weak Gag-specific CD4 T-cell response was detected by whole blood intracellular cytokine assay. Proviral DNA was positive by ultrasensitive nested (int) PCR and HIV DNA reservoir size was estimated at 2.2 copies/10^6^ PBMCs by semi-nested quantitative (RT) assay. DNA sequencing of Gag confirmed subtype-C virus. No replication-competent virus was detected in culture supernatants by Day 22 using p24 ELISA and ultrasensitive nested RT-PCR. All HLA loci were heterozygous (A*30:02:01/66:01; B*08:01:01/44:03:01; C*04:01:01/07:01:01; DRB1*12:01:01/13:02:01; DPB1*01:01:01/18:01; B1*05:01:01/06:09:01). The KIRAA1 genotype included both full-length and truncated KIR2DS4. Immunophenotyping showed few CCR5-expressing CD4 T-cells (6.6%), low CCR5 density, low immune activation (HLA-DR, TIGIT), high PD-1expression and high percentage of naive CD8 T-cells.

**Conclusions**: To our knowledge, this is the first case of sustained virological control from a randomized trial of ART interruption following early treatment of infants. Further investigation may expand our understanding of how the immune system controls HIV replication and inform future research strategies for ART interruption after early ART.

## TUPDC0107LB

### High level of retention and adherence at Week 48 for MSM and TGW enrolled in the PrEP Brasil demonstration study

B Grinsztejn ^1^; B Hoagland^1^; R Moreira^1^; E Kallas^2^; J Madruga^2^; I Leite^3^; R de Boni^1^; P Anderson^4^; A Liu^5^; P Luz^1^; V Veloso^1^; PrEP Brasil Study Group

^1^Fundacao Oswaldo Cruz, Instituto Nacional de Infectologia Evandro Chagas, Rio de Janeiro, Brazil. ^2^Universidade de Sao Paulo, Faculdade de Medicina, Sao Paulo, Brazil. ^3^Fundacao Oswaldo Cruz, Escola Nacional de Saude Publica, Rio de Janeiro, Brazil. ^4^University of Colorado Anschutz Medical Campus, Denver, USA. ^5^San Francisco Department of Public Health, Bridge HIV, San Francisco, USA

Presenting author email: gbeatriz@ini.fiocruz.br

**Background**: PrEP Brasil is a demonstration study to assess feasibility of daily oral tenofovir-disoproxil-fumarate plus emtricitabine (TDF/FTC) provided at no cost to high-risk men who have sex with men (MSM) and transgender women (TGW) within the Brazilian public health system. We report Week 48 PrEP retention, adherence to daily pill use, trends in sexual behaviour and incidence of HIV and sexually transmitted infections.

**Methods**: PrEP Brasil was initiated on April 2014; participants were followed for 48 weeks. Adherence was evaluated based on tenofovir diphosphate (TFV-DP) concentrations in dried blood spot samples. Logistic regression models were used to quantify the association of socio-demographic, behavioural and clinical variables with high levels of adherence (≥4 doses/week).

**Results**: 450 participants initiated PrEP, of which 376 (83%) were retained through 48 weeks. At Week 48, 73.7% (277/376) had protective levels consistent with ≥4 doses/week. Higher odds of achieving protective levels was observed among participants from São Paulo (adjusted odds ratio (aOR) 2.01, 95% confidence interval (95% CI) 1.16–3.47), as well as among those who reported sex with HIV-positive partners (aOR 1.77, 95% CI 1.03–3.04), and those who had protective levels of TFV-DP at Week 4 (aOR 3.26, 95% CI 1.87–5.68). The prevalence of rectal chlamydia and rectal gonorrrhea ranged from 8.0% and 4.9% at enrolment to 7.7% and 3.7% at Week 48, respectively (*p* = 0.88 and *p* = 0.42). Syphilis incidence was 9.0/100PY (95% CI 6.5–12.5). The mean number of sexual partners slightly decreased from 11.0 to 8.6 (*p* = 0.20) whereas the proportion of participants engaging in condomless receptive anal sex slightly increased from 44.7% to 47.8% (*p* = 0.37). Two individuals seroconverted during follow-up (HIV incidence 0.51 (95% CI 0.13–2.06) infections/100PY); both had undetectable TFV-DP levels at seroconversion.

**Conclusions**: Our results show high levels of retention and adherence to PrEP corroborating PrEP’s feasibility in a real-life setting of a middle-income country. Moreover, sexual behaviour and STI incidence remained stable over time, suggesting a lack of risk compensation in this population.

## WEPDC0106LB

### Are associations between HIV and HPV transmission due to behavioural confounding factors or biological effects?

C van Schalkwyk ^1,2^; J Moodley^3^; A Welte^2^ and L Johnson^1^

^1^University of Cape Town, Centre for Infectious Disease Epidemiology and Research, Cape Town, South Africa. ^2^University of Stellenbosch, DST/NRF Centre of Excellence in Epidemiological Modelling and Analysis, Stellenbosch, South Africa. ^3^University of Cape Town, Cape Town, South Africa

Presenting author email: carivs@sun.ac.za

**Background**: Epidemiological studies suggest twofold to fivefold unadjusted increased risk of newly detecting HPV infection for HIV-infected individuals and of HIV acquisition following HPV detection. Meta-analyses of the latter association, using estimates adjusted for behavioural risk factors, estimate approximately twofold increased risk. We conducted a mathematical modelling study to assess whether confounding behavioural factors and network effects are sufficient to explain associations between HIV and HPV infection status, without biological interactions.

**Methods**: MicroCOSM, a dynamic individual-based network model, was used to simulate epidemics of HIV and 13 oncogenic HPV types. Heterogeneity in sexual risk behaviour was represented by distinguishing relationship types and allowing for variation between individuals in their rate of partner acquisition and propensity for concurrent partnerships. No biological effects were assumed to modify transmission parameters of HIV in the presence of HPV infection and vice versa. Bayesian methods were used to calibrate the model to South African HIV and type-specific HPV prevalence data. Medians of the posterior distributions of the model parameters were used to simulate cohorts with biannual HIV and HPV testing from 2010 to 2015. Cox proportional hazards models were used to estimate hazard ratios for each of 100 simulated cohorts and mean hazard ratios were calculated.

**Results**: The 2010 mean HIV prevalence and oncogenic HPV prevalence among adults aged 15–49 in the 100 cohorts are 20.1% (95% CI 18.7–21.4%) and 38.9% (95% CI 36.5–41.2%), respectively. The modelled mean unadjusted hazard ratio of HIV acquisition following detection of an oncogenic HPV type is 3.2 (95% CI 2.6–3.8). The mean unadjusted hazard ratio for the effect of HIV on newly detected HPV is 3.7 (95% CI 3.4–4.1). Model findings are similar for different years of study enrolment and frequency of testing.

**Conclusions**: Model results are comparable to unadjusted results from observational studies, even when no biological effects are included. This suggests that observed associations between HPV and HIV transmission could be attributed to confounding by behavioural factors and network-level effects, implying that primary prevention of HPV (for example by vaccination) may not play a significant role in HIV prevention.

## WEPDD0106LB

### Accelerating progress towards the first 90 among men: a trial of the peer-based distribution of HIV self-test kits in Bulisa, Uganda

M Nanfuka ^1^; A Choko^2,3^; J Birungi^1^; G Taasi^4^; P Kisembo^1^; A Juliet^1^ and S Helleringer^5^

^1^The AIDS Support Organisation, Kampala, Uganda. ^2^Malawi-Liverpool Wellcome Trust Clinical Research Program, Blantrye, Malawi. ^3^London School of Hygiene and Tropical Medicine, Department of Infectious Diseases Epidemiology, London, UK. ^4^Ministry of Health, Kampala, Uganda. ^5^Bloomberg School of Public Health, John Hopkins University, Baltimore, USA

Presenting author email: nanfukam@tasouganda.org

**Background**: Too few men living with HIV are aware of their status, particularly in fishing communities around the great lakes of Africa. HIV self-testing (HIVST) addresses barriers preventing men from testing. But current approaches to distributing HIVST kits (e.g., through health facilities) only reach a subset of the men requiring HIV testing. We conducted a pilot trial of the distribution of HIVST kits through peer networks of fishermen.

**Methods**: We recruited seed participants among male patients of a TASO-supported facility. We introduced them to HIVST, and asked if they would distribute HIVST kits to peers who have not recently been tested for HIV. If so, we provided a package containing up to 5 HIVST kits (OraQuick), instructions and scripts addressing questions their peers may ask about HIVST. Recruited peers were referred to the study using a coupon with a unique number, and were asked to return the HIVST kit (used or unused). We conducted audio computer-assisted self-interviews with seeds and recruits to measure (a) the occurrence of adverse events (e.g., coercion) and (b) the uptake of HIVST kits. The accuracy of HIVST was measured against a confirmatory HIV test conducted by a health worker.

**Results**: A total of 19 seeds offered an HIVST kit to 115 men, and 95 (82.6%) accepted the offer. Among those, 29 had never been tested (25.8%), and 42 (44.2%) had tested more than a year ago. According to confirmatory testing, HIV prevalence was 4.3% among recruits (4/94). Compared to this standard, the sensitivity of HIVST was 100%. Three men living with HIV learned of their infection through HIVST (yield = 1 new diagnosis per 6.3 seeds). The specificity of HIVST was 93.3% (88/94). The 6 false positives were due to a faint grey line appearing on the test location of the OraQuick kit. No recruit reported coercion, but one seed experienced hostility from family members of a recruit.

**Conclusions**: A novel network-based distribution model of HIVST had high uptake and yield among men in this pilot study. It could constitute a crucial tool in reaching the 90-90-90 targets in under-served fishing communities in Uganda and elsewhere.

